# Profiling of basal and ligand-dependent GPCR activities by means of a polyvalent cell-based high-throughput platform

**DOI:** 10.1038/s41467-023-39132-x

**Published:** 2023-07-05

**Authors:** Manel Zeghal, Geneviève Laroche, Julia Douglas Freitas, Rebecca Wang, Patrick M. Giguère

**Affiliations:** 1 Department of Biochemistry, Microbiology and Immunology, University of Ottawa, Ottawa, ON K1H8M5 Canada; 2 Brain and Mind Research Institute, University of Ottawa, Ottawa, ON K1H8M5 Canada

**Keywords:** Biological models, High-throughput screening, G protein-coupled receptors, Permeation and transport

## Abstract

Representing the most attractive and successful druggable receptors of the proteome, GPCRs regulate a myriad of physiological and pathophysiological functions. Although over half of present pharmaceuticals target GPCRs, the advancement of drug discovery is hampered by a lack of adequate screening tools, the majority of which are limited to probing agonist-induced G-protein and β-arrestin-2-mediated events as a measure of receptor activation. Here, we develop Tango-Trio, a comprehensive cell-based high-throughput platform comprising cumate-inducible expression of transducers, capable of the parallelized profiling of both basal and agonist-dependent GPCR activities. We capture the functional diversity of GPCRs, reporting β-arrestin-1/2 couplings, selectivities, and receptor internalization signatures across the GPCRome. Moreover, we present the construction of cumate-induced basal activation curves at approximately 200 receptors, including over 50 orphans. Overall, Tango-Trio’s robustness is well-suited for the functional characterization and screening of GPCRs, especially for parallel interrogation, and is a valuable addition to the pharmacological toolbox.

## Abstract (web summary)

Interrogating the dynamic and functionally diverse signaling of GPCRs requires comprehensive cellular tools. Here Zeghal et al. develop Tango-Trio, a screening platform capable of profiling basal and drug-activated activities at hundreds of GPCRs.

## Introduction

As central orchestrators of cellular and physiological processes, G protein-coupled receptors (GPCRs) mediate the transduction of extracellular stimuli into conformationally-driven intracellular signals. Comprised of more than 800 members in the human genome, the diversity of this superfamily of membrane proteins is shaped by both the multiplicity of ligands they respond to, as well as the diverse array of signaling pathways they coordinate^1–3^. Moreover, GPCRs function in conjunction with protein interactors, whose identities and abundances vary by virtue of tissue- and/or cellular-specific expression^4^.

The dynamism of GPCR signaling events is due to the receptors’ conformational and locational changes throughout their life cycle, including activation, desensitization, internalization and resensitization. Although there is great diversity of ligands among them, GPCRs share a common fundamental mechanism of receptor activation. GPCRs in their inactive conformation are coupled to a heterotrimeric G-proteins complex, formed of a Gα subunit bound to GDP and Gβγ dimer stabilizing the inactive conformation of the heterotrimer. Activation of the GPCR results in conformational changes which enable the exchange of bound GDP by Gα for GTP, resulting in the dissociation of Gα-GTP and Gβγ-subunits from the receptor, which transduce different downstream signaling cascades depending on the nature of the GPCR and the subclasses of the G-protein subunits, composing the basis of G-protein dependent signaling^5,6^. This classical paradigm posits that activation can be induced not only by agonist binding, but also by virtue of GPCRs’ ability to spontaneously adopt active conformations in the absence of agonist, termed constitutive activity^7^. Although it is now widely recognized that all GPCRs exhibit spontaneous activation, albeit at varying degrees, a large-scale quantification of constitutive activity across the GPCRome, including druggable and orphan receptors, has yet to be conducted.

To prevent overstimulation, active GPCRs can be desensitized, wherein kinases such as GRKs phosphorylate the receptor at specific serine/threonine residues, typically C-terminal or intracellular loop 3 (IL3) sites^5^. Phosphorylation in turn leads to the recruitment of arrestins, the most well-known and characterized scaffold proteins comprising four isoforms, the two visual arrestins, arrestin-1 and arrestin-4, that are confined to retinal cones and rods, and the ubiquitously expressed nonvisual arrestins, β-arrestin-1 and −2^8^. While nonvisual arrestins have been shown to bind to hundreds of different GPCR members, the vast majority of demonstrations have been conducted with β-arrestin-2, with few studies addressing the contributions of the relevant but often forgotten β-arrestin-1 isoform^9^. Besides inducing receptor desensitization through steric hindrance of the G-protein binding site, arrestins also redirect GPCR signaling to alternative G-protein independent pathways such as MAPKs, JNKs, and Src^10^. Additionally, the engagement of arrestin initiates receptor internalization via dynamin- and clathrin-dependent endocytosis^11^. Besides canonical arrestin-mediated endocytosis, increasing evidence has emerged describing GPCRs internalizing independently of arrestins^12^.

Herein, we describe a comprehensive screening and interrogation platform evolved from the PRESTO-Tango, capable of the simultaneous interrogation of β-arrestin-2 recruitment at ~300 non-olfactory druggable GPCRs^13^. The reconstruction of this system was sought in part to increase the dynamic range and sensitivity of the original system, specifically improving the TRE promoter and TEV protease elements, and to expand its versatility beyond monitoring β-arrestin-2. Indeed, our platform, named Tango-Trio, includes monoclonal cell lines expressing trackers of β-arrestin-2, β-arrestin-1, and FYVE domain for internalization, all sharing the common luciferase reporter lineage. Moreover, their cumate-inducible nature enables the study of the various GPCR state-dependent and independent activities. Hereafter, we refer to the following states: the manifest agonist-induced active state; the constitutive active state, which represents ligand-independent activated receptor; steady-state, which refers to state-independent interaction level; and the basal level, which includes the steady-state plus constitutively active receptor pool, which cannot be discriminated in most cases. We are revealing divergent basal versus agonist-dependent β-arrestin-1/2 couplings, selectivities, and receptor endocytosis signatures across the GPCRome. We report the basal sigmoidal-fitted activities of more than 200 class A GPCRs, including ~50 orphans. Our findings represent a step towards uncovering the differences behind the mechanisms of constitutive versus agonist-induced activation, as well as state-independent activity. Moreover, we believe the Tango-Trio platform could facilitate the development of new GPCR-acting drugs and deorphanization efforts.

## Results

### Development of the Tango-Trio and its comparison to the PRESTO-Tango

The PRESTO-Tango has a number of advantages, including selective read-out as the response is specific to the target receptor, sensitivity due to signal integration to produce a read-out, and the ability to study a multitude of GPCRs as the assay is independent of the G protein family the receptor signals through^13^. As such, we exploited these strategic features to undergird the development of the Tango-Trio platform, while addressing its original limitations, chiefly the tetracycline-response element (TRE) promoter and tobacco etch virus (TEV) protease.

**Fig. 1:**
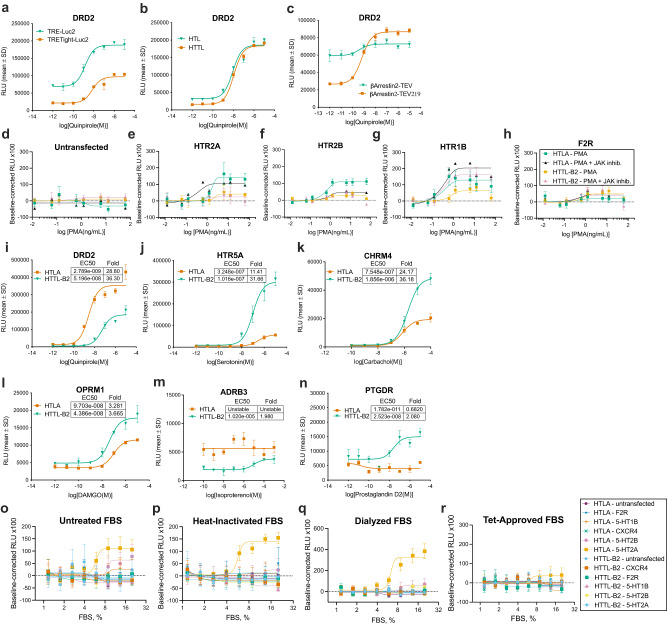
Optimization of the dynamic range, sensitivity, and specificity of the Tango-Trio platform. **a** Comparison of TRE and TRE-Tight. Promoters were cloned upstream luc2, and expression vectors were transfected in HEK293T cells along with the β-arrestin2-TEV fusion protein and DRD2. Transfected cells were stimulated with the DRD2 specific agonist quinpirole. **b** Selection and pharmacological characterization of the monoclonal reporter cell line HTTL (HEK293T-TRE-Tight-Luc2) compared to the original HTL (HEK293T-TRE-Luc) cell line. **c** Comparison of TEV and TEV219 proteases. β-arrestin2 was cloned to both proteases, and transfected in HTL cells with DRD2. Transfected cells were stimulated with the DRD2 specific agonist quinpirole. HTTL-B2 and HTLA were transfected with HTR2A (**e**), HTR2B (**f**), HTR1B (**g**), and F2R (**h**) and stimulated, along with untransfected cells (**d**), with dose-response curve of PMA and in presence/absence of 10 µM JAK inhibitor I. HTTL-B2 and HTLA dose-response curves at various targets: DRD2 to quinpirole (**i**), HTR5A to serotonin (**j**), CHRM4 to carbachol (**k**), OPRM1 to DAMGO (**l**), ADRB3 to isoproterenol (**m**), and PTGDR to prostaglandin D2 (**n**). **o**–**r** Comparison of the specificity of HTTL-B2 and HTLA readouts. Cell lines were transfected with GPCRs that activate the Jak/STAT Pathway and stimulated with serial dilutions of untreated FBS (**o**), heat-inactivated (**p**), dialyzed (**q**), and Tet-System Approved (**r**) sera. HTTL-B2 was maintained in cumate-containing media throughout. Dose- response curves were built using XY analysis for non-linear regression curve and the 3-parameters dose-response stimulation function. Data are presented as mean values, with error bars representing SD. Data are representative of 2 biological replicates, with 3 technical replicates each. Generic receptor codes refer to the GPCR-Tango constructs.

To stringently control gene expression, tTA binding to tetO7 permits transcriptional activation of the luciferase reporter^14^. However, the main limitation to the Tet system is the leakiness due to the strong positional effects on the tetO7 minimal promoter^15^, resulting in relatively high background transcription. In turn, this would lead to basal expression that would not be dependent on the tTA, which is intended to be cleaved from the GPCR by the β-arrestin2-TEV fusion protein. The second-generation promoter called TRE-Tight (Clonetech), redesigned tetO7 to remove potential bindings sites of endogenous transcription factors within the operon such as ISRE and GATA, renders this promoter virtually silent in the absence of induction^16^. As expected, lower RLU counts were obtained with TRE-Tight, but the induction factor remained higher for the TRE-Tight promoter (4.5 fold) compared to TRE (2.7 fold) (Fig. 1a); the dopamine D2 receptor (DRD2) Tango receptor was used as it is a strong β-arrestin2 recruiter^13^. Thus, the minimal basal leakage and increased fold window suggest TRE-Tight to be an improved promoter for Tango-Trio and reduce potential arrestin-independent modulation of the reporter activity.

Based on these observations, we generated a monoclonal TRE-Tight Luciferase reporter cell line for the Tango-Trio platform as an improvement over the HTL (HEK293T cells stably expressing TRE-Luc) cells of the original Tango assay (Fig. 1b). Although it is unknown whether Luc or Luc+ was used in creating the HTL cell line, we opted to clone TRE-Tight upstream the Luc2 gene (Promega), a markedly improved variant over its predecessors with significantly lower levels of cryptic transcription from the coding region and codon optimized expression^17^. Henceforth referred to as HTTL (HEK293T TRE-Tight-Luc), our reporter cell line had a comparable level of maximal expression to HTL but possessed a much lower baseline compared to its counterpart, resulting in a larger induction factor.

The Tango system involves a protein fusion consisting of β-arrestin-2 with TEV, which cleaves the engineered GPCR following β-arrestin-2 recruitment to the receptor to release the tTA. However, one limitation of the WT TEV is that it undergoes self-cleavage, generating a truncated protease with greatly diminished activity^18^. A variant of the WT, S219V-stop (TEV219), carries a stabilizing point mutation and was truncated to remove the auto-inhibitory C-terminal tail^19^. Given previous reports of this variant being 100-fold more stable than the full-length TEV and a more efficient catalyst, TEV219 was tested as a replacement for the WT TEV (Fig. 1c). TEV219 significantly lowered the baseline while producing maximal induced expression similar to that observed with the original protease, resulting in a signal ratio more than double (3.3-fold) that of TEV (1.3-fold).

Based on the aforementioned findings, β-arrestin-1, β-arrestin-2, and FYVE, a domain used to probe endocytosis given its high binding affinity and specificity to phosphatidylinositol 3-phosphate (PI3P)-enriched early endosomes^20^, were cloned to the chosen TEV219 protease. These trackers were subsequently transferred into the pcDH cumate-inducible destination lentivector, providing robust and reversible expression of genes, and adjustable expression levels by titrating the amount of cumate added to cell medium^21^. The effect of its addition in cumate-independent systems was assessed in the PRESTO-Tango, with negligeable changes to the basal signal in untransfected HTLA (Supplementary Fig. 1a), as well as at the arbitrary Tango-receptors tested (Supplementary Fig. 1b); nonetheless, considering that certain receptors produce weak maximum signals in Tango-based platforms, it is recommended that users test to confirm that cumate does not produce any significant agonistic or antagonistic behavior at the receptors they are employing. HTTL was used as the host cells for the subsequent generation of double stable cell lines, ensuring uniform genetic and reporter background. Monoclonal cell lines for β-arrestin1-, β-arrestin2-, and FYVE-TEV219, henceforth referred to as HTTL-B1, HTTL-B2 and HTTL-F respectively, were screened by functional assay and the final selection was based on pharmacological parameters, including baseline, efficacy, potency, and fold change (E_max_/E_0_). Seeing as the basal signal varies across the three different HTTL cell lines in the absence of receptor expression (Supplementary Fig. 2), the baseline was henceforth defined for each independent experiment and dose-curve construction, specifically as the mean luminescence readings of the three lowest drug dilution concentrations. Following selection, the amelioration of Tango-Trio over the PRESTO-Tango was assayed by comparing the original HTLA (HTL cells stably expressing β-Arrestin-2-TEV) cell line to our corresponding HTTL-B2.

While both GATA and ISRE are present in the TRE, the redesign of TRE-Tight saw the removal of only ISRE, with GATA still present as it is overlapping with tetO^22^. Based on previous work revealing that phorbol 12-myristate 13-acetate (PMA) activates the ISRE-Luc reporter and induces JAK-STAT signal transduction^23^, we postulated that activators of the Jak/Stat pathway would have an impact on the TRE promoter, but not TRE-Tight.

Corroborating this hypothesis, stimulation of HTLA and HTTL-B2 cells with PMA induced a significant response at 5-HT2A- and 5-HT2B-Tango receptors (2.6 and 8.6 fold, respectively) in the HTLA, but was absent in the latter, an effect that could be reversed in HTLA with the addition of Jak Inhibitor I (Fig. 1d–h)^24^. In the same vein, confirmation of the higher specificity of HTTL-B2 over HTLA is exemplified by the lack of activation observed following stimulation of transfected 5-HT2A-Tango receptor with untreated and heat-inactivated FBS, as well as with dialyzed FBS, sera with removed serotonin to prevent nonspecific activity at GPCRs^25^. This effect was also minimally observed with 5-HT2B-Tango, yet absent in the untransfected cells, and negligeable at 5-HT1B-Tango and at other GPCRs that activate the Jak/STAT Pathway, such as CXCR4- and F2R-Tango receptors^26^; interestingly, this artifactual response in HTLA was absent following stimulation with Tet-approved FBS (Fig. 1o–r). We believe that subtraction of external control of the TRE-Tight promoter compared to the original TRE explains the difference observed for β-arrestin-2 recruitment at some receptors. Hence, some factors present in the serum might artificially enhance promoter activity as shown for DRD2-, HTR5A-, CHRM4-, OPRM1-, ADRB3-, and PTGDR-Tango receptors (Fig. 1i–n).

**Fig. 2:**
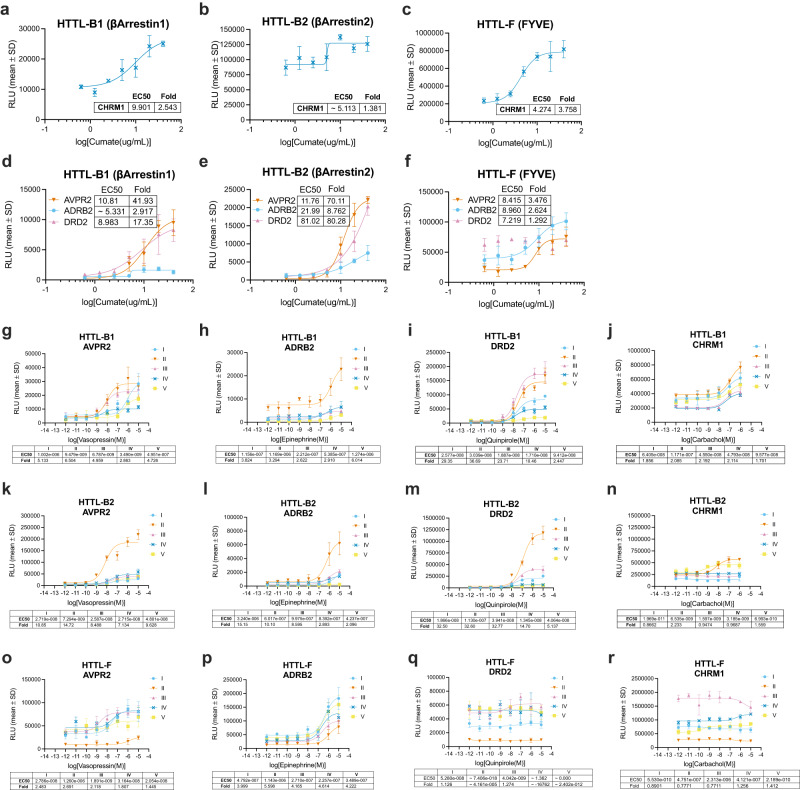
Dose-response and time-course verification of cumate-induced expression. Validation of fusion protein induction initiated by cumate dose-responses in HTTL-B1 (**a**, **d**), HTTL-B2 (**b**, **e**), and HTTL-F (**c**, **f**) cell lines. Cells were transfected with AVPR2, ADRB2, DRD2 and CHRM1 Tango receptors and stimulated with a cumate dose-curve starting from 40 µg/mL with 2-fold dilutions. Timing optimization of fusion protein induction in HTTL-B1 (**g**–**j**), HTTL-B2 (**k**–**n**), and HTTL-F (**o**–**r**) cell lines. Cells were transfected with AVPR2, ADRB2, DRD2 and CHRM1 Tango receptors and stimulated with receptor selective agonist. Cumate (30 µg/mL) was added at the following time points and maintained in the cell medium thenceforth: I − 5 days; II − 3 days; III − 2 days; IV − 24 h; V − 18 h total cumate exposure. Dose- response curves were built using XY analysis for non-linear regression curve and the 3-parameters dose-response stimulation function. Data are presented as mean values, with error bars representing SD. Data are representative of 2 biological replicates, with 3 technical replicates each. Generic receptor codes refer to the GPCR-Tango constructs.

To validate cumate induction, time-course and dose-response experiments were conducted on all three of our established cell lines using prototypical GPCR-Tango receptors covering the main subtypes of G-protein primary couplings: AVPR2 (G_s_), ADRB2 (G_s_), DRD2 (G_i_), and CHRM1 (G_q_)^27^. To confirm the control of gene expression was dose-dependent, monoclonal cell lines were transfected without cumate, and then stimulated with a cumate concentration-curve starting from 40 μg/mL with 2-fold dilutions (Fig. 2a–f). Based on the EC50 values of tested receptors, maximal activation is achieved at ~10 μg/mL, corroborating other studies that have also used the cumate switch system^28^. Cumate induction was also confirmed to have minimal impact on the basal signals and fold windows of the three HTTL cell lines after reaching maximal activation, yet a slight decrease was generally observed for both parameters at the highest tested induction concentration (Supplementary Fig. 3). The time-course experiments consisted of adding cumate at the different time points and maintaining it in the cell medium from then on, ranging from as long as 5 days total cumate exposure to a minimum of 18 h throughout (Fig. 2g–r). In general, the best response was observed following ~3 days of total cumate exposure, which we expected as the developers of the cumate-gene switch previously observed a maximal expression after 72 h^21^. Overall, Tango-Trio presents greater dynamic range, sensitivity, specificity, and versatility over the original tried-and-true Tango system.

### Tango-Trio generates a compendium of GPCRome basal and agonist-dependent activities

**Fig. 3:**
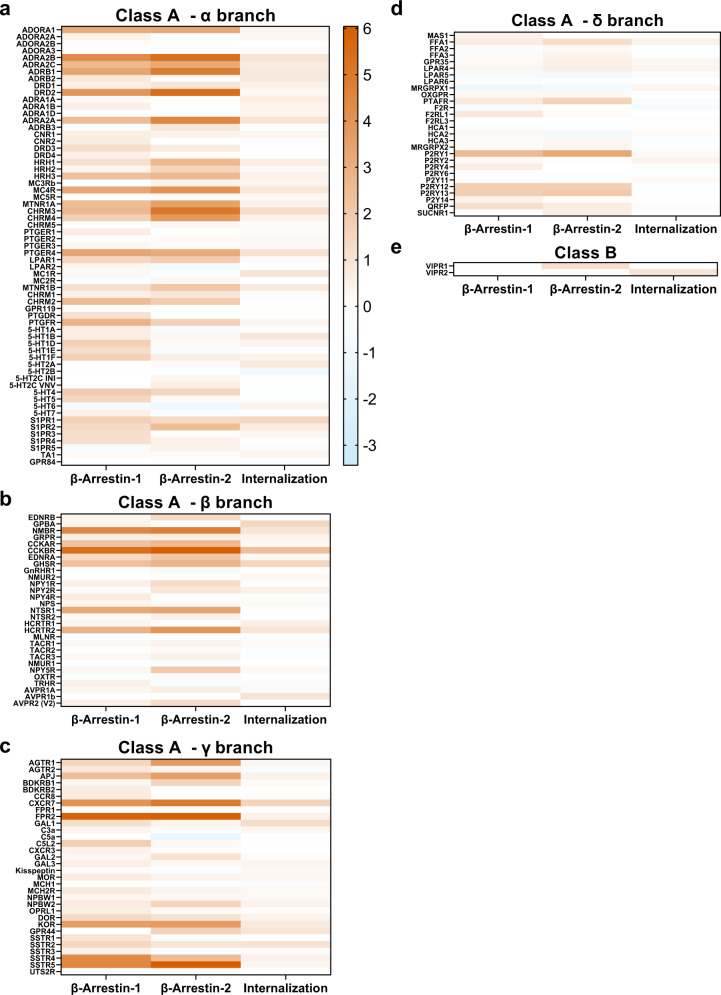
Heatmap representation of hits identified from agonist-dependent HTS. To analyze agonist-induced activities within the GPCRome, HTTL-B1, HTTL-B2 and HTTL-F cells were plated in cumate-containing (30 µg/mL) medium and transfected with a library of 162 non-orphan GPCR Tango constructs. Transfected cells were stimulated either with HBSS-Hepes buffer or with a panel of selective agonists. Log2 fold changes in agonist-dependent arrestin recruitment/dissociation or GPCR internalization was calculated between the wells in the absence or presence of agonist and plotted as heat maps, grouping class A α (**a**), β (**b**), γ (**c**), and δ (**d**) branches, and class B receptors (**e**). Log2 values are the means calculated from quadruplicate conditions, generated from two separate screens (*n* = 8, 2 biological measurements with 4 technical replicates each). Generic receptor codes refer to the GPCR-Tango constructs.

**Fig. 4:**
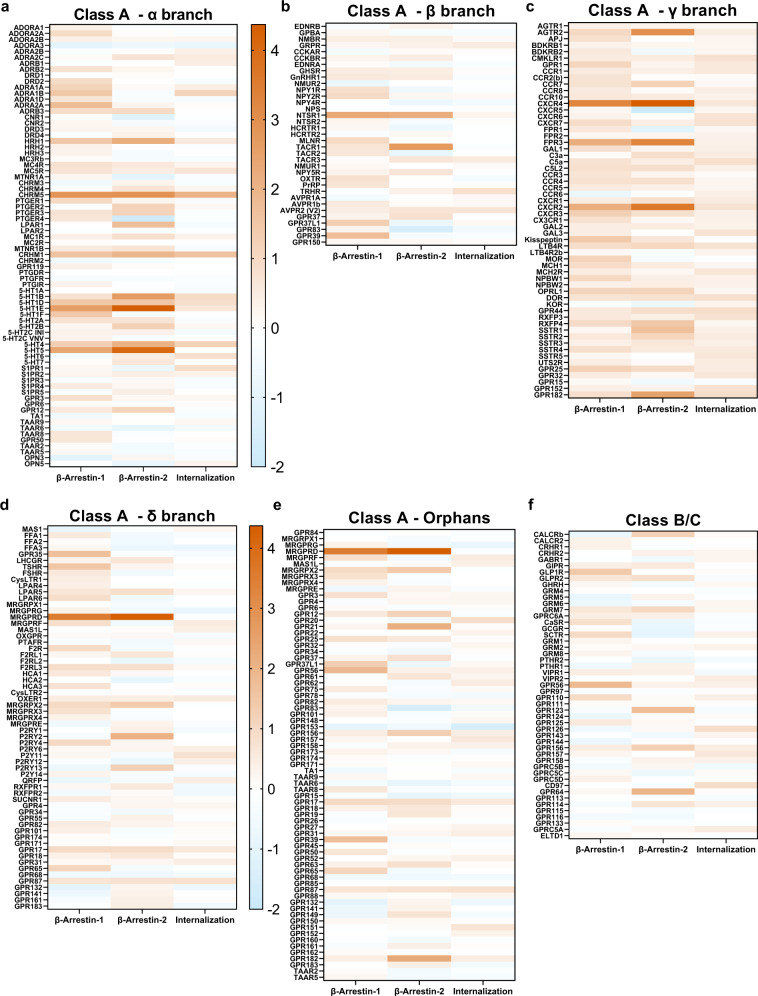
Heatmap representation of hits identified from basal activity HTS. To analyze basal activities within the GPCRome, HTTL-B1, HTTL-B2 and HTTL-F cells were plated alternating rows with or without cumate (30 µg/mL). Cells were transfected with a library of 350 GPCR Tango constructs, including ~100 orphan receptors. Log2 fold changes in basal arrestin recruitment/dissociation or GPCR internalization was calculated between the wells in the absence or presence of cumate and plotted as heat maps, grouping class A α (**a**), β (**b**), γ (**c**), and δ (**d**) branches, class A orphans (**e**) and class B/C receptors (**f**). Log2 values are the means calculated from quadruplicate conditions, generated from two separate screens (*n* = 8, 2 biological measurements with 4 technical replicates each). Generic receptor codes refer to the GPCR-Tango constructs.

With our established monoclonal cell lines forming the foundations of the Tango-Trio HTS platform, parallel interrogations of the GPCRome were conducted by transfecting a panel of 350 GPCR PRESTO-Tango constructs in arrayed format, the large majority of which consist of Class A members^29^. To investigate proximal GPCR-arrestin interactions and receptor endocytosis at the two possible activated receptor states, agonist-induced activities were screened for in presence of selective agonist at ~150 non-orphan GPCRs, while basal activity was probed for using the presence of cumate. Based on initial hit thresholds set to <−2 and >2 log2, fold-changes (E_max/_E_0_) were visualized as heatmaps to depict contrasts between the couplings of arrestin isoforms and corresponding GPCR internalization efficiencies (Figs. 3a–e and 4a–f). Based on NC‐IUPHAR classification, heatmaps were constructed for each of the four branches of the non-olfactory class A members (α, β, γ and δ), for orphan class A members, and one covering a select number of receptors spanning the B, C, and adhesion classes, illustrating the diversity within the GPCR superfamily^27,30^.

Consistent for both screenings, the similitudes of profiles amongst receptor members of the same subfamily varied on a case-by-case basis. Drawing on examples from the α branch, the Alpha-1 adrenergic-Tango receptors (ADRA1A, 1B, 1D) all preferentially recruited β-arrestin-1 over β-arrestin-2 (~2-3 fold difference) at the basal level, whereas in the case of the Prostaglandin EP-Tango subfamily (PTGER1–4), different basal arrestin selectivity profiles were observed among members, such as PTGER2-Tango’s marked selectivity towards β-arrestin-2 and that of PTGER4-Tango towards β-arrestin-1 (Fig. 4a). These marked differences in arrestin selectivity profiles between receptor subfamilies could be due to the differences in their C-terminal tail and intracellular loop sequences which dictate different phosphorylation codes, influencing the isoform type and degree of arrestin recruitment. It is also important to note however that these selectivity findings must be interpreted with the understanding that all Tango receptors are fused to a “V2 tail”, originating from the C-terminus of AVPR2. Such a phospho-peptide addition is also present in other β-arrestin recruitment assays such as the PathHunter assay^31^. The V2 tail was originally added given its high affinity for β-arrestin2 and for its ability to stabilize the interaction between a given receptor and the recruited arrestin; this addition is indispensable for those of low-affinity or for transient β-arrestin recruitment at many GPCRs. The stabilization of the interaction allows efficient cleavage by the TEV protease but also generates a detectable basal level, resulting in an increased assay quality (z-factor), which is strongly affected when RLU are too low; this is particularly important when performing parallel interrogation or high-throughput screening. When working on a single or a select set of receptors, the V2-tail can easily be removed and experimental conditions adjusted to achieve an acceptable level of RLU counts, if possible. However, for some receptors, the interaction of β-arrestin is of low affinity or transient such that it cannot be accurately detected using a protease-dependent reporter assay. In such cases, the V2 tail should be retained or an alternative assay such as BRET should be envisaged. To further compare GPCR-Tango constructs used in Tango-Trio and unmodified wildtype counterparts, a supplementary table comprising the agonist-induced β-arrestin-1/2 recruitments observed from the EMTA studies and our Tango-Trio work was compiled (Supplementary Table 1). Although a large number of receptors behave similarly toward β-arrestin-1/2 recruitment, several discrepancies were noted. For example, Tango-Trio detects β-arrestin recruitment at HTR1D-, PTGER1-, GNRGR- and MTNR1B-Tango receptors, while the EMTA biosensors were unsuccessful, or oppositely, β-arrestin recruitment at F2R, LPAR1, LPAR2 and VIPR1 was observed with EMTA but not with Tango-Trio. Moreover, a stronger proclivity for β-arrestin-2 over β-arrestin-1 was also observed in Tango-Trio for certain receptors, such as AGTR1-, PTGER4-, HCRTR2-, and AVPR2-Tango receptors. Similarly, another facet to consider is the influence of the V2-tail on the changes of the arrestin-independent and dependent internalization patterns of GPCRs. For example, based on the HTTL-F agonist-dependent screen (Fig. 3), ADRB1-, 5-HT2A-, ADRA2A-, CHRM3-, CHRM4-Tango receptors all exhibited significant internalization following agonist stimulation, all of which have been previously reported to undergo arrestin-independent internalization; however, other reported GPCRs exhibiting this behavior such as DRD3, DRD4, UTS2R, AGTR1, ENDRA, EDNRB and APJ were not among our hits. It is not surprising to observe a certain degree of inconsistency between two heterologous systems^32^. Numerous possibilities could thus contribute to the inconsistency observed, especially for β-arrestin recruitment, which requires receptor phosphorylation by endogenous kinases. In addition to endogenous modulators, such as kinases, the fusion of the receptor and β-arrestins with functionalized proteins tags can affect the recruitment and/or stability of the complexes. The presence of the phosphopeptide (V2 tail) could also contribute to some divergences, but the significant difference in the duration of the experiments (<1 vs. 18 h) is probably a major factor, especially for efficacy, which is strongly dependent on cell surface receptor abundance. Notwithstanding these discrepancies, we are confident that comparing EC50 (potency) and Emax (efficacy) to an internal reference will provide an accurate differential measure, but as is the case for any artificial system, we cannot rule out that β-arrestin recruitment is over/underestimated compared to endogenous recruitment in a physiological context. The main advantage of our Tango-Trio assay remains the ease of performing parallel high-content or high-throughput screening.

Across the array of interrogated GPCRs, it is obvious that β-arrestin-1/2 are quite promiscuous; however, similarly to Avet et al. who reported that 22% of receptors investigated did not recruit arrestins beyond their established threshold^33^, we also observed a significant pool of receptors which exhibited no β-arrestin-1/2 translocation at either basal or agonist-induced states. It should be noted however that very few GPCRs lacked arrestin interactions at both of these states. Additionally, as seen in Figs. 3 and 4, there is little overlap between basal and agonist-induced signatures across the GPCRome; for example, very strong agonist-induced arrestin recruitment at SSTR5-Tango did not correspond to high basal activity (Figs. 3c and 4c). This implies that there are different mechanisms at play that regulate internalization and arrestin activities between an agonist-stabilized GPCR versus basal activity in the absence of agonist^34^, as discussed below.

The representation of our screens as heat maps allows one to easily identify receptors with the strongest basal activities, such as CHRM5-, 5-HT1E-, 5-HT5-, NTSR1-, CXCR4-, and MRGPRD-Tango. To exclude the possibility of cumate addition contributing to marked increase of receptor expression, an ELISA was conducted to evaluate receptor surface expression on a select subset of constitutive hits (Supplementary Fig. 4). No significant differences between non-treated cells versus those with the addition of saturating cumate concentration, compared to the drastic fold-differences observed in the constitutive screen, corroborated that detected hits were a result of bona fide constitutive activities; for example, a modest 1.2-fold difference in receptor expression was observed in HTTL-B2 cells transfected with CXCR4-Tango, compared to the 67-fold change in constitutive β-arrestin-2 recruitment. The lack of correlation between receptor expression and apparent basal activity was also confirmed across a larger panel of receptors including a wide range of basal arrestin recruitment profiles (Supplementary Fig. 5). Finally, absolute receptor expression levels were not found to affect constitutive activity, as titrating Tango construct DNA did not generally reduce the cumate-induced fold change (Supplementary Fig. 6).

### Validation of GPCR internalization, β-arrestin-1/2 coupling and selectivity profiles

**Fig. 5:**
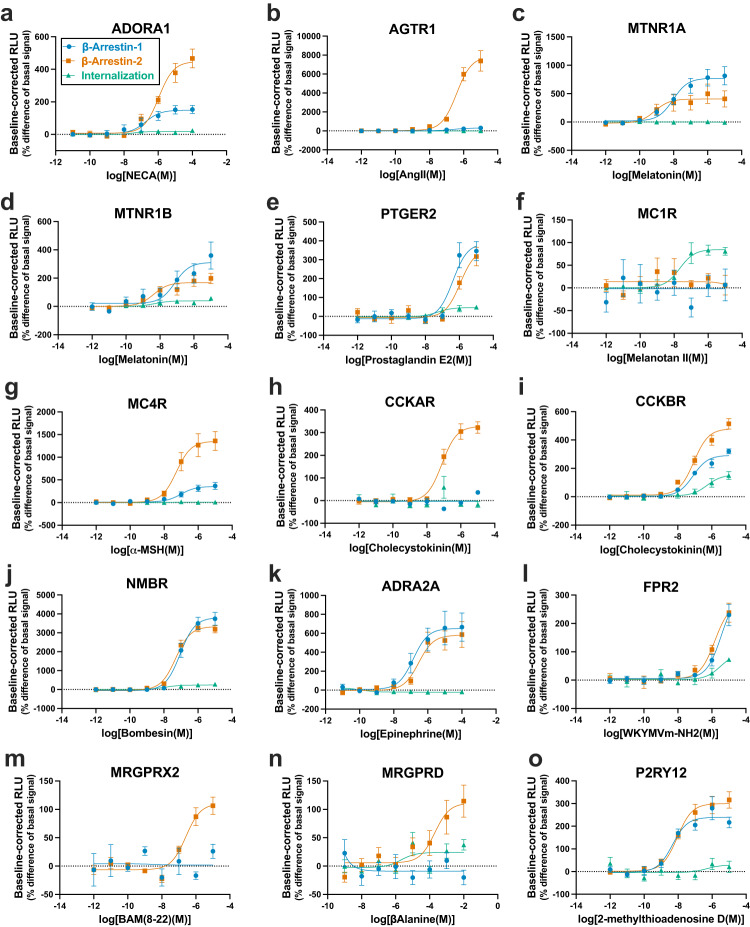
Validation of compiled positive hits from agonist-dependent HTS in dose-response. **a**–**o** HTTL-B1, HTTL-B2 and HTTL-F cells were plated in cumate-containing (30 µg/mL) medium and transfected with potential GPCR hits identified from the agonist-dependent HTS. Transfected cells were stimulated with the receptor specific agonist and dose- response curves were built using XY analysis for non-linear regression curve and the 3-parameters dose-response stimulation function, followed by baseline correction. Data are presented as mean values, with error bars representing SEM. Data are representative of 2 biological replicates, with 3 technical replicates each. Generic receptor codes refer to the GPCR-Tango constructs.

**Fig. 6:**
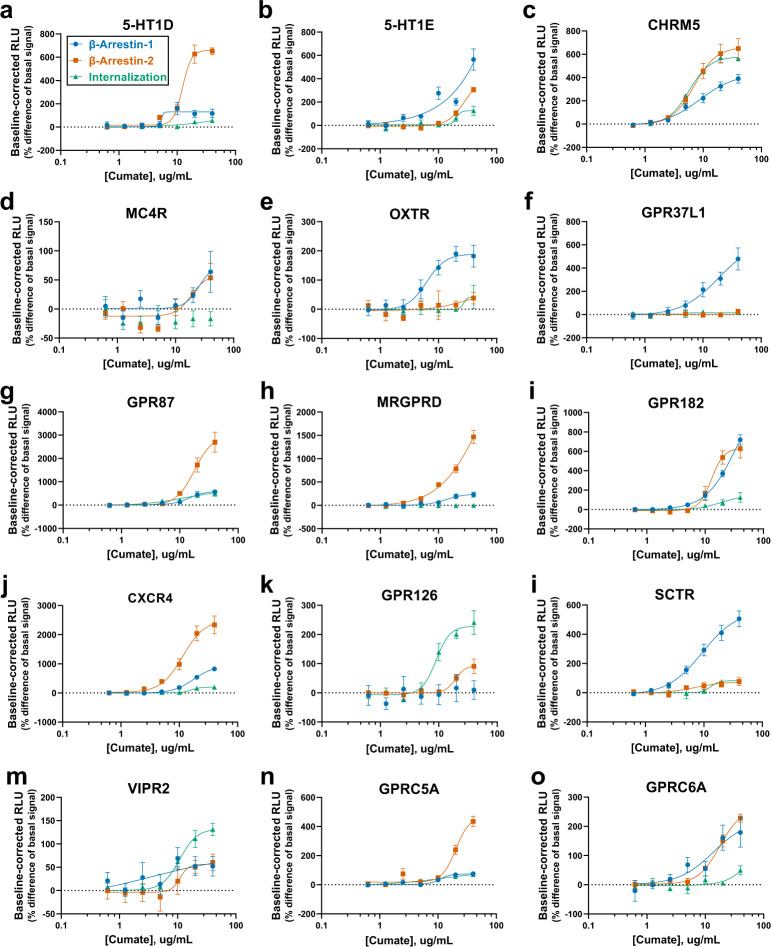
Validation of compiled positive hits from basal activity HTS in dose-response. **a**–**o** HTTL-B1, HTTL-B2 and HTTL-F cells were transfected with potential GPCR hits identified from the basal HTS. Transfected cells were stimulated with cumate, and dose- response curves were built using XY analysis for non-linear regression curve and the 4-parameters dose-response stimulation function, followed by baseline correction. Data are presented as mean values, with error bars representing SEM. Data are representative of 2 biological replicates, with 3 technical replicates each. Generic receptor codes refer to the GPCR-Tango constructs.

Secondary screening of top potential hits was carried out in a dose-dependent manner (agonist or cumate, accordingly) to validate our platform’s high-throughput performance (Figs. 5a–o and 6a–o). With the primary screen findings in agreement with our concentration-response profiles, thus confirming the platform’s reproducibility, we exploited Tango-Trio to perform more detailed analyses of the arrestin selectivities and corresponding GPCR endocytosis patterns observed. This was accomplished by producing the β-arrestin-1/2 and internalization dose-response curves for ~150 non-orphan GPCRs, and most important, presenting for the first-time dose dependent constitutive activation curves at ~200 receptors, including more than 50 orphans (Supplementary Figs. 11–26). Many of our high-basal and agonist-dependent findings are in agreement with previous studies, such as high constitutive activity at GPR182-Tango receptor (Fig. 6i)^35,36^. Moreover, Tango-Trio was able to detect activity at GPCRs that could not be validated in PRESTO-Tango^13^, including BAM-22 at MRGRPX2-Tango (Fig. 5m), and β-Alanine at MRGPRD-Tango (Fig. 5n).

While HTTL-B1 and HTTL-B2 captures the nuanced differences between arrestin couplings and selectivities at receptors, our internalization measures are not as robust. Especially in our HTTL-F agonist-dependent screen, very few hits were detected, with the majority of receptors producing the strongest agonist-induced internalization belonging to the β branch, such as GPBA-, NMBR-, CCKBR-, GHSR-, and HCRTR2-Tango receptors (Fig. 5i, j). On the other hand, our HTTL-F is better-suited for studying constitutive endocytosis, seeing as considerably more receptors had stronger constitutive internalization profiles, such as GPR126-, GPR87-, and CHRM5-Tango (Fig. 6c, g, k). As discussed below, the selectivity of FYVE-targeted early endosome trafficking should be re-evaluated.

Based on our validation of hits from our β-arrestin-1, β-arrestin-2, and FYVE screens, distinct GPCR selectivity profiles towards the arrestin isoforms were observed. Regarding constitutive activity, receptors can be clustered into three distinct functional classes, specifically those that interact fairly equally with both isoforms, those that preferentially recruit β-arrestin-1 over β-arrestin-2, and vice versa. Based on constitutive activity curves, it seems that the constitutive internalization at a given receptor corresponds to the profile of one of the β-arrestin isoforms, such as those observed at 5-HT1D-, CHRM5-, OXTR-, SCTR-, and GPRC5A-Tango receptors, amongst others (Fig. 6a, c, e, l, n). This observation follows the widely accepted classical paradigm of how arrestins play a central role in GPCR endocytosis via the predominant clathrin-mediated pathway. However, this is not a uniform correlation, involving exceptions where either significant constitutive internalization is observed in the absence of arrestin activities, for example in the case of GPR126- and VIPR2-Tango (Fig. 6k, m) or oppositely, strong β-arrestin-1 and/or β-arrestin-2 recruitment but negligeable receptor internalization, such as the case of GPR37L1- and MRGPRD-Tango (Fig. 6f, h). Indeed, these findings confirm that GPCR endocytic pathways are more diverse than originally defined, as an increasing number of receptors are found to be endocytosed via alternate pathways besides clathrin-mediated, including the caveolae-dependent and fast endophilin-mediated endocytosis (FEME) pathways, as well as another 30 known examples of GPCRs have been found to internalize independently of arrestins altogether^32^.

As for agonist-dependent activity, although similar arrestin selectivity profiles were also observed, the vast majority fell under two functional classes, equal (e.g., Fig. 5c, d, j) or preferential (e.g., Fig. 5b, g, m) recruitment of β-arrestin-2 over β-arrestin-1. Our results parallel those of Oakley et al. who delineated two major classes of receptors based on the 10 GPCRs that they studied, namely “Class A” receptors which bound β-arrestin2 with higher affinity than β-arrestin1, and “Class B” receptors which bound both β-arrestin isoforms with similar high affinities^37^. Similar subsets were observed in Avet et al.’s recent publication profiling the engagement of different G-protein families at 100 therapeutically relevant GPCRs, including β-arrestin1 and β-arrestin2 following agonist stimulation^33^. Orthogonal validation of GPCRs with pronounced arrestin isoform selectivities was conducted in BRET2, which revealed discrepancies between the two systems (Supplementary Fig. 7). For example, β-arrestin1 is recruited at a much lower efficacy compared to β-arrestin2 at AGTR1 in Tango-Trio (Fig. 5b). In BRET however, this selectivity is not observed, with very little difference in recruitment observed between the two isoforms (Supplementary Fig. 7e). Given that BRET experiments occur over a short duration, the results obtained are based on the amount of receptor present at the time of adding the ligand, and thus, other factors such as receptor internalization, the role of the intracellular pool, and binding kinetic profiles will have minimal effects, whereas the Tango-Trio, being a signal amplification system, may disproportionately magnify of efficacies due to the aforementioned factors. Nonetheless, both systems have different limitations and are useful in their own respects for the purposes of screening and pharmacological characterization and should not be interpreted as a measure of endogenous recruitment, but rather as a pharmacological tool to compare drug activity towards a refence compound.

Thus, by using Tango-Trio to screen the GPCRome and distinguishing functional subsets of GPCRs, this might give molecular insight into structural interface positions common among these related receptors, which could be involved in recruitment and internalization.

### Mechanistic insights into basal GPCR activities revealed by Tango-Trio

**Fig. 7:**
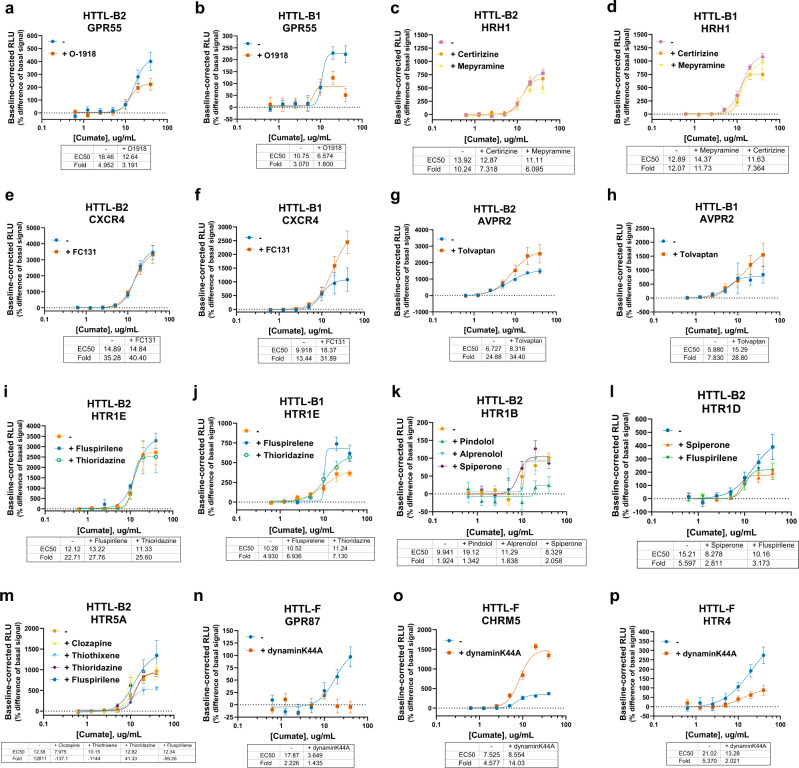
Applications and further investigations into basal activities revealed by Tango-Trio. HTTL-B1 and HTTL-B2 were transfected with GPCRs exhibiting strong basal arrestin recruitment. Transfected cells were stimulated as cumate dose-response in the presence or absence of the following inverse agonists/antagonists at saturating (EC80) concentrations: O-1918 at GPR55 (**a**, **b**), Cetirizine and Mepyramine at HRH1 (**c**, **d**), FC131 at CXCR4 (**e**, **f**), Tolvaptan at AVPR2 (**g**, **h**), Fluspirelene and Thioridazine at HTR1E (**i**, **j**), Pindolol, Alprenolol and Spiperone at HTR1B (**k**), Spiperone and Fluspirelene at HTR1D (**l**), and Clozapine, Thiothexene, Thioridazine and Fluspirelene at HTR5A (**m**). Dynamin-dependence of high basal internalization was tested by co-transfecting HTTL-F cells with GPR87 (**n**), CHRM5 (**o**), and HTR4 (**p**) with/without dynaminK44A. Transfected cells were stimulated as a cumate dose-response, and stimulation curves were built using XY analysis for non-linear regression curve and the 4-parameters dose-response stimulation function, followed by baseline correction. Data are presented as mean values, with error bars representing SEM. Data are representative of 2 biological replicates, with 3 technical replicates each. Generic receptor codes refer to the GPCR-Tango constructs.

Besides the wealth of basal and agonist-induced activation profiles generated with Tango-Trio, additional explorations of the applications of this platform were undertaken, including studying inverse agonists and their relative abilities to subdue constitutive activity versus steady-state recruitment. Seeing as inverse agonism may appear differently based on cell phenotypes^38^, we chose a panel of drugs classified as either inverse agonists or antagonists to target GPCRs exhibiting high constitutive β-arrestin-1 and/or β-arrestin-2 recruitment (Fig. 7a–m)^39^. A spectrum of inverse agonistic properties was validated, albeit no drug was able to completely ablate the basal activity observed. For example, O-1918 reduced the response observed at GPR55-Tango by almost half in both HTTL-B1 and HTTL-B2 (Fig. 7a, b)^40^, while at HRH1-Tango, stimulation with Mepyramine reduced constitutive activity only for β-arrestin-2, unlike its counterpart Cetirizine which could inhibit activities in both cell lines (Fig. 7c, d). Furthermore, certain compounds previously designated as antagonists/inverse agonists, such as FC-131 at CXCR4-^41^ (Fig. 7e, f) and Tolvaptan at AVPR2-Tango (Fig. 7g, h), increased the constitutive translocation of β-arrestin-1 and β-arrestin-2 in our system. Thus, our platform, nor other arrestin-based assays, are not entirely suitable for quantifying measurements of inverse agonism given the range of arrestin activities observed. For instance, in both PRESTO-Tango and Tango-Trio, Tolvaptan increased arrestin recruitment at AVPR2-Tango, while Pindolol resulted in a depletion of arrestin recruitment at the 5-HT1B-Tango (Supplementary Fig. 8b, e). To exclude the possibility of artifacts arising due to endogenous cleavage GPCR-Tango fusion constructs, the same receptors were co-transfected with β-arrestin-2 in HTTL, with no arrestin recruitment detected (Supplementary Fig. 9). Thus, although the identification of inverse agonists is not possible with arrestin-based assays per say, Tango-Trio is valuable for their characterization, more specifically providing information about their effects on constitutive arrestin recruitment and receptor internalization. It seems that G-protein uncoupling using inverse agonist is clearly a different receptor pool or receptor state and cannot be directly translated toward β-arrestin activity. We cannot rule out that G-protein uncoupling could result in β-arrestin recruitment for some receptors, as seen for CXCR4- (FC131-treated) and AVPR2- (Tolvaptan-treated) Tango receptors.

Tango-Trio HTS produces variegated snapshots of the arrestin couplings of GPCRs and their corresponding internalization efficacies, suggesting that the mechanisms of endocytosis among GPCR members are more heterogeneous than originally conceived, especially those of constitutive nature. To further this point, the dominant-negative dynaminK44A was co-transfected in HTTL-F cells with select GPCRs exhibiting strong constitutive internalization. As expected, various degrees of inhibition were observed, from partial inhibition in the case of 5-HT4-Tango (Fig. 7p), to complete blocking of GPR87-Tango endocytosis (Fig. 7n), suggesting a greater dynamin-dependence involved during its constitutive internalization process. Intriguingly, overexpressing dynaminK44A resulted in a substantial increase in constitutive CHRM5-Tango endocytosis (Fig. 7o), bringing to light the possibility of multiple compensatory internalization mechanisms at play at a given receptor. Expanding this idea, β-arrestin-1/2 knockdown was performed to evaluate the arrestin dependence at certain GPCRs with high constitutive internalization, confirming partially arrestin-independent endocytosis at CHRM5- and CD97-Tango receptors (Supplementary Fig. 10a, e).

**Fig. 8:**
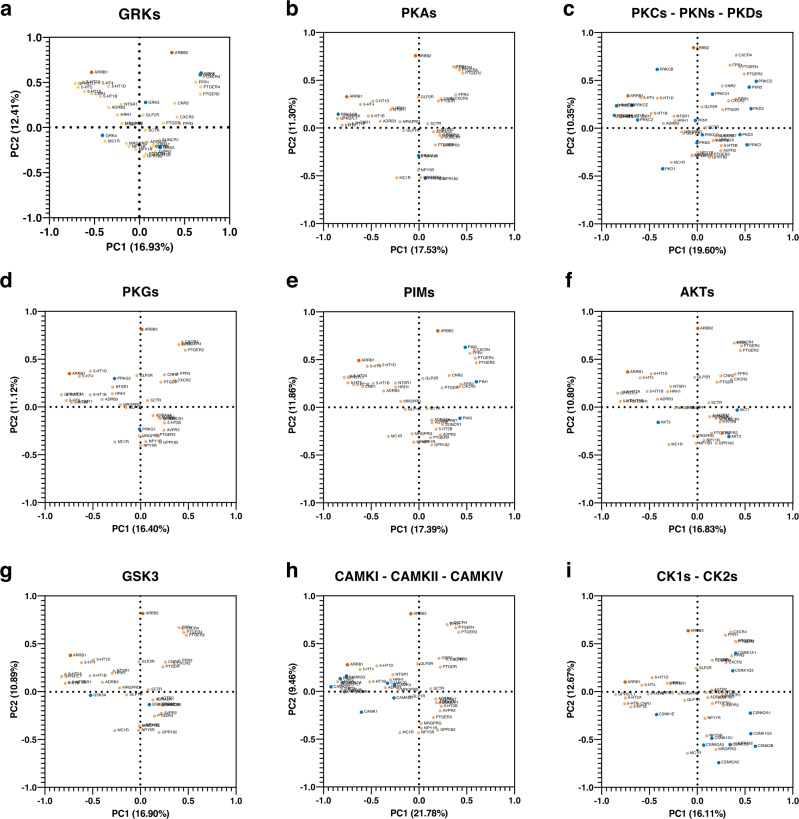
Visualization of tissue-specific expression levels of select GPCRs with high basal activities, serine/threonine kinases, β-arrestin-1 and β-arrestin−2. Human Protein Atlas (HPA) RNA consensus tissue gene data (version 21.0 and Ensembl version 103.38.) summarizing the expression levels in 55 tissues was extracted for β-arrestin-1 and −2 (ARRB1 and ARRB2), select receptors with high constitutive selectivity for one arrestin isoform over the other (GPR182, AGTR2, ADRA2A, GPR37L1, SCTR, ADRB3, PTGER4, SUNCR1, PTGER3, MRGPRG, NPY5R, NPY1R, GLP1R, FPR1, MC1R, FPR3, 5-HT5, MRGPRD, GPR87, CXCR4, HRH1, AVPR2, 5-HT4, 5-HT2A, NTSR1, GLP2R, 5-HT1D, CXCR2, 5-HT1B, 5-HT1E, PTGER2, 5-HT2B, PTGDR), and either and either GRKs (**a**), PKA (**b**), PKCs, PKNs, and PKDs (**c**), PKGs (**d**), PIMs (**e**), AKTs (**f**), GSK3 (**g**), CAMKI, CAMKII, and CAMIV (**h**), CK1s and CK2s (**i**). The data was analyzed using principal component analysis; β-arrestin-1 and −2 are denoted with red, ST kinases with blue, and GPCRs with orange symbols. Generic receptor codes refer to the GPCR-Tango constructs.

A recent appreciation has grown for GPCR-interacting proteins, with emerging findings supporting how they modulate GPCR expression at the cell surface, signal transduction, and receptor endocytosis, amongst others^42^. Of particular note are the protein kinases that phosphorylate specific sites on the intracellular loops and C-terminal tail of GPCRs, inducing specific arrestin roles and varying functional consequences for the modified receptors^43^. Given limited literature exploring the distinct functions of kinases and their contributions to constitutive activity, we examined the HPA consensus tissue-specific expression levels of serine/threonine-specific protein kinases (ST kinases) previously reported to phosphorylate GPCRs, such as GRKs, PKAs, and PKCs amongst other^43^, as well as the expression levels of β-arrestin-1/2 and of select receptors with high constitutive selectivity for one arrestin isoform over the other. We postulated that the kinases and receptors of similar tissue expression profiles may have overlapping activities. Our generated PCA plots revealed varying signatures for each of ST kinase families, some of which forming clusters with our constitutively active GPCRs based on shared expression patterns (Fig. 8). Based on the GRK plot, it seems that GRK2, GRK6 and β-arrestin-2 might share a functional network^44^, especially at CXCR4^45^ and PTGER2, which were found to be highly selective for β-arrestin-2 at constitutively active receptors (Fig. 8a). A dense cluster including GRK5 also leads us to speculate a greater involvement of this GRK at constitutively active receptors selective for β-arrestin-1, such as AGTR2, ADRA2A, PTGER3, and SUNCR1. The restricted expression profile of GRK4 also suggests that this might be the predominant GRK acting at receptors such as MC1R. Despite a lack of research into the lesser-reported kinases capable of phosphorylating GPCRs, certain interactions could be confirmed from past studies, such as the role of PIMs at CRCR4 (Fig. 8e)^46,47^. Thus, our analyses may also reveal functions at these ST kinases GPCRs; for example, we hypothesize that Ca^2+^/calmodulin-dependent kinases, including CAMKI, CAMKII and CAMKIV complexes, might significantly contribute to the phosphorylation and subsequent recruitment of β-arrestin-1 to GPCRs, although this has yet to be experimentally investigated (Fig. 8h).

## Discussion

The functional heterogeneity of GPCRs is attributed in part to their spectral activation states, desensitization, and internalization, accounting for their multifaceted signaling processes. Towards establishing accurate and comprehensive functional profiles, this study developed Tango-Trio, a polyvalent screening platform consisting of a triad of stable cell lines, capable of interrogating constitutive and agonist-activated GPCRome activities. The foundation of the platform, the HTTL reporter cell line, stably expresses a luciferase gene under the control of an improved low-background and sensitive TRE-Tight promoter compared to the original PRESTO-Tango^16^. To increase the versatility of this system, Tango-Trio monitors the translocation of β-arrestin1, β-arrestin2, and GPCR internalization using a FYVE domain, all of which were cloned to the truncated TEV219 protease for its enhanced signal-noise ratio^19^; these chosen elements provide valuable insight into crucial stages in the GPCR life cycle. Despite sharing a high degree of structural and sequence similarity, the non-visual arrestin isoforms have been reported to accomplish disparate roles^9^. For example, one study demonstrated that silencing β-arrestin2 reduced agonist-induced PAC1R and C3aR receptor internalization, whereas silencing β-arrestin1 had no effects^48,49^. Given its implications in signal regulation, desensitization, resensitization, and ligand scavenging functions of some receptors, our measurements of GPCR internalization also contribute to our account of the diversity of GPCR-dependent dynamics^50^.

The scope of our platform is broadened further by the controlling the expression of these multiple probes using a cumate-controlled lentiviral vector^21^, enabling the interrogation of not only at the agonist-induced state, but also monitoring basal activities in a dose-dependent manner by adjusting the expression level of the fusion proteins, which is not feasible with existing HTS technologies. Two aspects affect the magnitude of basal activity, specifically a receptor’s conformational flexibility from the inactive to active states in the absence of ligand, and state-independent GPCR-effector coupling. Previous quantifications of constitutive activity have been extracted based on the latter factor, specifically constructing receptor-density response curves by regulating the amount of receptor expression and measure resultant increases in the basal responses^38^. Tango-Trio modulates GPCR-effector coupling stoichiometry not through changing receptor density, but by tuning the density of cytoplasmic effectors, specifically β-arrestin1, β-arrestin2, and FYVE, to probe basal activity. Moreover, seeing as continuous overexpression of engineered proteins could potentially give rise to spurious GPCR dynamics^51^, titratable induction of Tango-Trio fusion proteins enables greater management of the cellular environment to one more reflective of the native in vivo setting if desired, by tuning the intensity of gene expression during profiling of agonist-stimulated receptors.

Following establishment of our stable cell lines, we conducted HTS of the GPCRome to shed light into how β-arrestin1 and β-arrestin2 differ in their couplings and selectivity at GPCRs, and provide insight on how this arrestin selectivity may play a role in GPCR trafficking properties. After validating select hits from our primary screen, we sought to further characterize constitutive and ligand-dependent dynamics. Given the inability to capture pharmacological parameters (EC_50_, E_max_/E_0_, etc.) regarding basal activities in general, as well as the lack of information regarding ligand-induced β-arrestin1 recruitment and internalization across the GPCRome, we profiled ~200 GPCRs, including over 50 orphans, in cumate and/or agonist dose-response fashion. Interestingly, we observed distinct functional signatures between basal and agonist-dependent activities observed at GPCRs, suggesting different mechanisms in play at these different activation states^34^. Although the promiscuity of couplings detected for both isoforms is supported by the fact that these non-visual β-arrestins are ubiquitously expressed to regulate hundreds of GPCRs within the human body^8^, we observed a significantly larger percentage of receptors which were more selective towards β-arrestin2 over β-arrestin1 at both basal and agonist-activated states. From a structural standpoint, β-arrestin2 has less defined secondary structure within its C-terminal basket, resulting in increased flexibility and adaptability to the structural differences of GPCRs^52^, which may attribute why it is less selective and couples preferentially to more receptors compared to β-arrestin1. Thus, Tango-Trio may be useful for future development and testing of arrestin-isoform biased compounds, if there are positive functional outcomes that are shown to emerge from favouring β-arrestin1 versus β-arrestin2 recruitment; although few reports of this nature of functional selectivity have been explored, a couple of existing agonists have been demonstrated to favor one arrestin isoform over another, such as 2-arachidonoylglycerol and anandamide^53^. Addedly, the original PRESTO-Tango platform interrogates only the recruitment of β-arrestin2, one of the two non-visual arrestins expressed in vertebrates. While it has been previously demonstrated that β-arrestin1 is the most prevalent isoform in most cells, comprising more than 90% of the total arrestin complement^54^, few studies have investigated its recruitment to GPCRs on a larger scale; Tango-Trio enables such an interrogation, and simultaneous comparison to β-arrestin2 selectivity. Finally, some receptors lacked interactions with either isoform, which has been typically attributed to the lack of consensus sequences for GRKs^55^. Nonetheless, a growing body of evidence has indicated that arrestin recruitment is not entirely dependent on phosphorylation by GRKs specifically, but also by other serine/threonine kinases^43^, as we explored later on. Moreover, arrestin recruitment is a biphasic process that involves the partial engagement of the phosphorylated GPCR’s C-tail with arrestin and a fully engaged complex where the receptor core interacts with the arrestin finger loops^56^. As a highly sensitive reporter assay, we cannot exclude that Tango-Trio can detect partially engaged complexes to phosphorylated and non-phosphorylated receptors. Given that interaction with the receptor core requires complete activation of the receptor and G-protein dissociation, the constitutive interaction observed is probably highly dependent on receptor C-tail phosphorylation and thus highly dependent on kinases present within the cellular system used^57^. It will be interesting in futures studies to measure the changes in this receptor constitutive distribution while overexpressing a specific kinase.

Unfortunately, one caveat to Tango-Trio is that the dynamic window of GPCR internalization is smaller than that observed with arrestin activity. This could be attributed to multiple reasons but we believe that the range of observed internalization across the GPCRome is not as expansive as the range of arrestin activities. Moreover, the transit of the receptor to early endosomes could be too fast for efficient TEV cleavage or may lead to weak tTA translocation into the nucleus. Another potential pitfall of using the FYVE domain to track GPCR internalization is the known PI3K activation by some GPCRs, which in turn will increase phosphatidylinositol 3-phosphate (PIP3) at the cell membrane and endosomes, possibly contributing to biased results. Another important distinction is that for HTTL-B1 and HTTL-B2, it is the expression of arrestin effector proteins that is affected by cumate induction, hence tuning the process of GPCR-arrestin coupling itself, whereas for HTTL-F, titratable cumate addition does not influence the process of receptor internalization, but rather only changes the expression of the FYVE probe that tracks it. Finally, the FYVE domain directly interacts with the inositol polar heads of PIP3, which is present on the surface of the endosome but also at the plasma membrane. It has been proposed that dimerization of FYVE-domain containing protein amplifies the weak binding of individual FYVE fingers to the phospholipid. We cannot exclude that the TEV219 fusion disrupts dimerization, nor that the expression of only the FYVE-domain has a very low affinity, thus reducing sensitivity. We do not exclude to test other fusion proteins such as EEA1 containing the FYVE domain and the adjacent dimerization coiled-coil region^58^. Overall, the use of FYVE domain fusion protein for tracking GPCR internalization should be used with caution; nonetheless, the variety of responses observed in our GPCRome screening highlights some interesting observations that will require further investigation.

Tango-Trio is especially valuable for studying orphan receptors, whose lack of identified endogenous ligands presents a challenge to studying said receptors^59^. Seeing as existing tools have focused on detecting classical G-protein signaling, Tango-Trio’s ability to quantify activity independent of G-proteins is a suitable tool for deorphanization efforts^60^. By using the Tango-Trio to investigate the internalization profiles and potential biases of orphan receptors towards different isoforms of β-arrestin, we hope our platform can identify ligands for orphans that would not be detected by G-protein dependent changes, especially given that Tango-Trio represents an assay which is able to concurrently quantify and compare the degree of constitutive β-arrestin1/2 translocation and internalization at a GPCRome-wide level, and is also capable of profiling orphan activity in a dose-dependent manner.

Interestingly, none of the inverse agonists tested in our study was able to completely block the basal arrestin recruitment. For this reason, we defined the basal activity as the summation of state-dependent constitutive activity and state-independent activity (steady-state). We cannot exclude that amplification systems, including the Tango assay, fail to accurately detect constitutive activity, which represent a small percentage within the ensemble of a receptor’s conformational landscape^61^. Given that basal activity is highly cell-dependent as it influenced by the basal phosphorylation of GPCRs^57^, it is difficult to discriminate between basal and constitutive activity in our system apart from using inverse agonists. Thus, the partial decrease in arrestin recruitment observed for certain compounds, such as O-1918, Cetirizine, and Thiothixene, could be attributed to their effects on the small population of constitutively active receptors, but they cannot blunt the level of basal arrestin recruitment. Nonetheless, if this were the case, further studies would be warranted to explain why a significant enhancement in arrestin recruitment was observed for other inverse agonists, such as FC131 and Fluspirilene. It is becoming more evident that receptor activation follows a multistate model in which G-proteins and arrestins stabilize specific conformations. Each of these conformations can be further stabilized by different ligands, leading to a broad intrinsic efficacy landscape, which in certain cases, can be opposite to each other when comparing two signaling pathways^62^. Finally, for some receptors, the uncoupling of G-proteins with an inverse agonist could facilitate β-arrestin recruitment. Another important consideration is that in vitro assays may fail to capture the true potency and efficacy of certain drugs with slow on-rates^63^.

While Tango-Trio’s ability to measure basal activity supports the discovery and study of inverse agonists in vitro, whose properties can be assessed based on the depression of constitutive arrestin translocation, it should be complemented with G-protein dependent profiling, especially since constitutive activity, by definition, is observed due to spontaneous G-protein activation^7^. Indeed, several inverse agonists profiled in our study produced either no change or an enhancement in constitutive arrestin recruitment, and a previous study saw no arrestin recruitment occurring in the presence of the GHSR inverse agonist SPA^61^. Therefore, given the diversity of responses we observed based on the type of inverse agonist tested, arrestin activities should not be the sole measure of the inverse agonistic properties of a compound. Although assays such as BRET will capture the direct effect of most drugs onto the conformational landscape of the receptors, some have a more complex pharmacology, acting as allosteric modulators, bitopic ligands, protean agonists, pharmacochaperones, or even a mixed pharmacological profile depending on receptor subpopulation. Moreover, the overall resultant activity, when used in vivo, is a summation of all effects of the drug toward the receptor when used for an extended period and thus, must also include its impact on receptor abundance and receptor post-translational modification. These effects are rather slow and require prolonged incubation of the drug onto the cell expressing the target receptor. In those cases, a reporter system such as the Tango-Trio, which involves incubation with drugs for >8 hrs, may capture pharmacological behavior not detected with short term incubation (<1 h).

By using the dynaminK44A loss-of-function mutant, we were also able to determine the extent of dynamin dependence during internalization^64^. However, given the numerous dynamin-dependent (e.g., clathrin-mediated, FEME,) and -independent pathways (e.g., CLIC/GEEC, micropinocytosis), further investigations into the other mediators of endocytosis are needed to elucidate the specific type of endocytosis mechanism employed at a given receptor. Of notable distinction is FEME, which has been shown to cargo several amine GPCRs, including ADRB1, ADRA2A, DRD4, and CHRM4, caveolae mechanisms observed for ADRB2, AGTR1, ENDRA, and GLP2R, and of course, the canonical clathrin-dependent endocytosis such as at APJ, DRD3, and CHRM3^32,65^. Arrestin-dependence of either isoform during the internalization of a given GPCR can also be orthogonally assayed used dominant negative arrestins^66^.

While Tango-Trio uses modified GPCR constructs, the findings presented herein demonstrate that the addition of the V2-tail itself cannot fully account for differences between our Tango-based platform and those that employ unmodified WT GPCRs, such as the EMTA system. With regard to the V2 tail, while its original purpose was to increase basal β-arrestin2 recruitment and thus may artificially enhance its detection, seeing as it was added to all the receptors found in the PRESTO-Tango kit, any artificial increases in the Tango signal would still be proportional amongst all. Furthermore, PRESTO-Tango developers tested the effects of removing the V2 tail for some receptors and found variable results; notably, the removal of the V2 tail decreased the ligand-induced responses of some, e.g., the FFAR2 free fatty acid receptor, and had little effect on the ligand-induced responses of others e.g., the LTBR4 leukotriene receptor^1^. Another factor that could also contribute to the discrepancies in results between EMTA and Tango-based methods is the effect of receptor internalization when β-arrestin1/2 are overexpressed, especially considering the difference in the duration of the experiments. Overexpression of β-arrestin1/2 could contribute to increased internalization for certain receptors, which could account for the extent of arrestin recruitment, such as the case with OPRM1; agonists will also vary in their capacity to induce endocytosis of a given receptor; e.g., DAMGO at OPRM1 promotes rapid internalization, as opposed to morphine^67^. On the other hand, given that EMTA experiments occur over a short duration, the result obtained is based on the amount of receptor present at the time of adding the ligand, and thus, other factors such as receptor internalization will have minimal effects unlike in Tango-based. Finally, one of the biggest factors that limit comparisons between systems is that the EMTA method, as like other systems which use unmodified GPCRs, requires overexpression of GRK2 to examine β-arrestin-1/2 interactions, a limitation which could influence the cellular context, stoichiometry and levels of expression of GRKs, possibly leading to potential artifactual downstream signaling/arrestin recruitment measurements obtained. For example, the EMTA method found that among the receptors able to recruit β-arrestins, only a very small number selectively recruited β-arrestin-1 (1.3%) or β-arrestin-2 (6.4%), most of them recruiting both β-arrestins in the presence of GRK2 (92.3%)^33^. Tango-Trio does not require the overexpression of GRKs, so is preferable in that regard, and the level of expression of β-arrestin-1/2 and Fyve fusion proteins is modifiable using cumate induction, to be as close to the native environment/context as possible. If GRK2 or other kinases were overexpressed in Tango-Trio, we believe that several of the GPCRs with weak/no arrestin signals would also be detected in our system, and the degree of preferential β-arrestin isoform recruitment may also shift. In short, it must be underscored that both systems may artificially increase arrestin recruitment, either using modified GPCR constructs such as in Tango-Trio or using WT GPCRs but with the addition of a kinase such as in EMTA. Nonetheless, based on the intended purpose of an experiment, these methods have their own advantages and disadvantages and thus one system cannot truly replace the other, but rather both should be used as a complement.

One limitation to the profiles captured by Tango-Trio is that they may not be portable from different tissues, as the level of expression and identities of GPCR interactors directly influence the regulation of receptor signaling, localization and trafficking^4^. One study demonstrated that the constitutive activity of glutamate metabotropic receptors is depressed in the presence of Homer 3 scaffold protein^68^. Another example reports that of the GRK family members, GRK4 exclusively mediates the constitutive phosphorylation of DRD1^69^. Relevant to Tango-Trio, mRNA expression analysis of endogenously expressed GPCR-related proteins reveals that while HEK293 cells, the cell lineage upon which our platform was established, express numerous isoforms and full repertoires of numerous GPCR-interacting proteins, such as PKA and PKCs, they also do not express significant levels of certain essential effectors, such as GRK2^70^, thereby impairing complete profiling at certain GPCRs dependent on GRK2 phosphorylation. The importance of GRK specificity is epitomized by a study in which GRK2 and GRK3 phosphorylation of their tested receptors (ADRB2 and CHRM2) was agonist-dependent, whereas GRK5 and GRK6 were able to phosphorylate in the absence of agonists^71^. Despite the challenge introduced by tissue-specific variations, Tango-Trio is a rich resource of arrestin coupling, selectivity and internalization profiles of hundreds of GPCRs, which can be confirmed and supplemented using orthogonal assays.

Studying GPCR activities and differences between signaling events is crucial for expanding our mechanistic understanding of GPCR signaling, and in turn, advancing the development of improved GPCR-targeted therapeutics. Towards these efforts, our wealth of data will help to functionally characterize GPCRs based on their β-arrestin1 and β-arrestin2 couplings, selectivities, and internalization efficacies. On a larger scale, the versatility and robustness of our platform is well-suited to illuminating the big picture on the elements governing GPCRome pharmacological activities. We envision Tango-Trio to spur a transformational change on the study of basal and constitutive GPCR activities, and to promote research into GPCR constitutive versus agonist-induced activation mechanisms.

## Methods

### Cell culture

Human Embryonic Kidney cells (HEK293T) were maintained in Dulbecco’s modified Eagle’s medium (DMEM) supplemented with 5% fetal bovine serum (FBS), 5% bovine calf serum (BCS), and 100 μg/mL of penicillin-streptomycin at 37 °C in a humidified atmosphere containing 5% CO_2_.

HTL (HEK293T stably expressing a luciferase reporter gene) and HTLA cells (HTL cells stably expressing a human β-arrestin-2 fused to Tobacco Etch Virus protease), both kindly provided by Dr. Richard Axel, were maintained in DMEM supplemented with 5% FBS, 5% BCS, 100 μg/mL of penicillin-streptomycin, 2.5 μg/mL of puromycin and 50 μg/mL of hygromycin.

HTTL were generated by transfection of HEK293T with modified pNLCoI1 vector (Promega) containing the luciferase2 (luc2) coding sequence under the control of the TRE-Tight promoter. Cells were transfected using PEI transfection method^72^ and selected with hygromycin at 100 μg/mL. Colonies were picked, expanded, eventually duplicated, and further tested in 6-well format by transient transfection of a receptor and β-arrestin2-TEV219/pcDNA3.1+. The best clone was selected based on growth and β-arrestin2 recruitment at different GPCRs, which were previously validated by PRESTO-Tango^13^.

HTLL-B1, -B2 and -F were generated by lentiviral infection of pCDH-CuO-MCS-EF1α-CymR-T2A-Bleo3 SparQ plasmid encoding β-arrestin2-TEV219, β-arrestin1-TEV219 or FYVE-TEV219 as per supplier instructions and selected using zeocin at 200 μg/mL. Colonies were picked, expanded, eventually duplicated, and further tested in 6-well format by transient transfection of a given receptor. The best clone was selected based on growth and β-arrestin1/2 recruitment or internalization at previously validated GPCRs.

Tango-Trio cell lines generated herein (HTTL, HTTL-B1, HTTL-B2, HTTL-F) are maintained continuously on dishes coated with 5 μg/mL collagen (Gibco). Tango-Trio cell lines are readily available and free of charge from the corresponding author upon request.

### Transfection

Cell transfections were performed using a modified polyethyleneimine (PEI) transfection method^72^. Briefly, 1.5 × 10^6^ cells were plated in a collagen-coated well of a 6-well plate with 2 mL of complete growth medium. 2 μg of DNA was mixed with 200 μL of Opti-MEM medium followed by addition of 6 μL of PEI (Polysciences) reagent stock solution (1 mg/mL, pH 7.0). The mixture was added dropwise to cells after 20 min incubation at room temperature. Medium was changed the next day and replaced with complete fresh medium. For stable cell line generation, antibiotics were added 48-hours post-transfection.

### Tango β-arrestin recruitment assay

Assays were performed using modifications of the original Tango assay, as detailed below^13,29^. Cells were plated on collagen-coated dishes and transfected by the PEI precipitation method as described above. The day following transfection, the cells were plated in DMEM supplemented with 1% dialyzed FBS into collagen-coated 384-well white clear bottom cell culture plates at a density of 20,000 cells/well (or 16,000 cells/well for same-day transfection) in a total volume of 40 μL. The following day or the same day 5 h after seeding, ligand solutions were prepared in filtered assay buffer (20 mM HEPES, 1× Hanks’ balanced salt solution (HBSS), pH 7.40) at 3X and added to cells (20 μL per well) for overnight incubation (16–20 h). Cumate, at indicated concentrations, was directly added in the complete medium from a water-soluble stock solution (10,000X in 95% ethanol). For most experiments, cumate was added at the time of cell plating (a day before transfection) and kept throughout the experiment. For time-dependent experiments, cumate was added as indicated in the text. The following day, media and drug solutions were removed, and 20 μL per well of homemade luciferase detection reagent (108 mM Tris–HCl; 42 mM Tris-Base, 75 mM NaCl, 3 mM MgCl2, 5 mM Dithiothrei-tol (DTT), 0.2 mM Coenzyme A, 0.14 mg/ml D-Luciferin, 1.1 mM ATP, 0.25% v/v Triton X-100, 2 mM Sodium hydrosulfite) was added. Plates were incubated for 10 min at room temperature in the dark before counting using Synergy Neo2 microplate reader (BioTek Instruments) and collected using Gen5 software v3.11 (BioTek Instruments). Data were subjected to non-linear least-squares regression analysis using the sigmoidal dose-response function (3-parameters modeled using Y = Bottom + (Top-Bottom)/(1 + 10^((LogEC50-X))); 4-parameters modeled using Y = Bottom + (X^Hillslope)*(Top-Bottom)/(X^HillSlope + EC50^HillSlope)) provided in GraphPad Prism v9.5.1. Data is presented as Relative Luminescence Units (RLU) and was processed (calculation of mean, SD or SEM, baseline correction as percentage difference using 100* (Value-Baseline)/Baseline) as indicated in figure legends. Parallel interrogation was performed as previously published by us^29^.

### Measurement of cell surface expression by ELISA

HTTL-B1, HTTL-B2 and HTTL-F were plated in collagen-coated 6-wells either with or without 30 µg/mL cumate. 24 h later, cells were transfected with a select number of validated GPCR hits from the constitutive HTS. Transfected cells were subsequently re-plated in 384-well plates at 30,000 cells/well and fixed for 10 min using 20 µL/well of 4% paraformaldehyde. Blocking was performed by incubating cells for 30 min with 20 µL/well of 5% normal goat serum in PBS, followed by the addition of 20 µL/well of 1/10,000 diluted anti-FLAG-HRP conjugated antibody (MilliporeSigma) for 1 h and two washes of 80 µL/well PBS. Supersignal ELISA Femto Substrate (Thermo Fisher Scientific) was applied per well, and luminescence was subsequently read with Synergy Neo2 microplate reader (BioTek Instruments).

### Principal component analysis and visualization of RNA tissue-specific expression data

Human Protein Atlas (HPA) RNA consensus tissue gene data (version 21.0 and Ensembl version 103.38., accessed at https://www.proteinatlas.org/about/download) summarizing the expression levels in 55 tissues was extracted for β-arrestin-1 and −2 (ARRB1 and ARRB2), for select receptors with significant constitutive selectivity for at least one arrestin isoform (GPR182, AGTR2, ADRA2A, GPR37L1, SCTR, ADRB3, PTGER4, SUNCR1, PTGER3, MRGPRG, NPY5R, NPY1R, GLP1R, FPR1, MC1R, FPR3, 5-HT5, MRGPRD, GPR87, CXCR4, HRH1, AVPR2, 5-HT4, 5-HT2A, NTSR1, GLP2R, 5-HT1D, CXCR2, 5-HT1B, 5-HT1E, PTGER2, 5-HT2B, PTGDR), and the following serine/threonine kinases: GRKs (GRK2, GRK3, GRK5 GRK6), PKA (PRKACA, PRKACB, PRKACC), PKCs, PKNs, and PKDs (PRKCA, PRKCB, PRKCG, PRKCD, PRKCE, PRKCH, PRKCQ, PRKCI, PRKCZ, PKN1, PKN2, PKN3, PKD1, PKD2, PKD3), PKGs (PRKG1-2), PIMs (PIM1-3), AKTs (AKT1-3), GSK3 (GSK3A, GSK3B), CAMKI, CAMKII, and CAMIV (CAMK1D, CAMK1G, CAMK2A, CAMK2B, CAMK2D, CAMK2G, CAMK1, CAMK4, PNCK), CK1s and CK2s (CSNK1A1, CSNK1D, CSNK1E, CSNK1G1, CSNK1G2, CSNK1G3, CSNK2A1, CSNK2A2, CSNK2A3, CSNK2B). Despite protein levels not always equating to RNA expression levels, the latter was used as it was more complete than the existing protein expression data. The data was analyzed using principal component analysis (PCA) on our standardized data (Xstandardized = (Xraw − X̄)/sx, where X̄ is the mean and sx is the standard deviation of the variable value). The number of PCs were selected using GraphPad Prism v9.5.1’s Parallel Analysis Approach (*n* = 1000 Monte Carlo simulations; PC1 and PC2 selected with eigenvalues greater than the 95th percentile of simulated counterparts), and subsequently visualized as loading plots.

### Bioluminescence resonance energy transfer (BRET2) measurements

HEK293T cells were seeded in 6 well plates at 1.2 × 10^6^ cells per well and were transfected with 0.5 µg of GPRC-RLuc8 construct and 0.5 µg of β-arrestin1/2-GFP2 using Jetprime (PolyPlus transfection). Following transfection, cells were detached and split on PLL-coated white 96-well assay plates (Perkin Elmer). 24 h later, spent medium was aspirated and replaced with 60 µL of 1X HBSS buffer, followed by 30 µL of serial dilutions of agonist at 3X concentration. Plates were incubated as 37 °C for 30 min, and 10 µL of Coelenterazine 400a (Nanolight Technologies) at 50 µM was added to each well, for a final concentration of 5 µM. Plates were incubated for 10–15 min at room temperature to allow the signal to stabilize, and subsequently read using the Hidex Sense Beta Plus microplate reader (Gamble Technologies) with 405 nm (RLuc8-Coelenterazine 400a) and 500 nm (GFP2) emission filters, at 1 s/well integration times.

### shRNA knockdown, RNA isolation and RT-qPCR assay

Lentiviral β-arrestin-1 and −2 shRNA plasmids, obtained from the High-Throughput Screening Lab at the Children’s Hospital of Eastern Ontario Research Institute, were transfected in HEK293T cells, along with psPAX2 and VSV-G vectors. The medium was replaced the following day with complete growth medium, and lentiviral shRNA medium was collected following 48 h transfection. For the knockdown experiment, HTTL-F cells were seeded in either complete medium or in the previously prepared lentiviral β-arrestin-1 and −2 shRNA medium (combined at a 1:1 ratio), with infection of cells facilitated with polybrene at 8 μg/mL.

Total RNA was isolated from transduced HTTL-F cells using the RNeasy Mini Kit (Qiagen) and quantified using the NanoDrop 2000 spectrophotometer (Thermo Fisher Scientific). First strand cDNA was synthesized with 900 ng of extracted RNA using the TransStart IV Reverse Transcriptase Kit (TransGen) according to the manufacturer’s protocol. Human Actin (sense: 5’- CATGTACGTTGCTATCCAGGC-3’; antisense: 5’- CTCCTTAATGTCACGCACGAT-3’), β-arrestin-1 (sense: 5’- CCTGACCTTTCGCAAGGACC-3’; antisense: 5’-CAAGCCTTCCCCGTGTCTTC-3’) and β-arrestin-2 (sense: 5’-AAGCTCACCGTGTACTTGGG-3’; antisense: 5’-AGGGTCACAAACACTACAGGG-3’) primers were synthesized by IDT, Inc. Quantitative real time PCR experiments were performed with 2 µL of the synthesized cDNA in a total volume of 20 µL using the SYBR™ Green PCR Master Mix (Thermo Fisher Scientific), with the following cycling parameters: 95 °C for 10 min, followed by 40 cycles of 95 °C for 30 s, 60 °C for 30 s and 72 °C for 30 s. Data was analyzed using the comparative Ct (ΔΔCT) method, with the relative degree of response determined by 2^−(ΔΔCT)^.

### Molecular biology

TRE-Tight-Luc2 expression plasmid was constructed using the pNLCoI1[luc2-P2A-NlucP/Hygro] Vector (Promega) as backbone vector (Accession no. KM359771), and a stop codon was added using QuikChange mutagenesis (Agilent) at the end of luc2 gene. This vector was chosen because it contains a synthetic poly(A) transcription pause site before the promoter, which reduces background and does not contain any SV40 ori, which is not compatible with the large T antigen expression in HEK293T. TRE-Tight promoter was PCR amplified from pTRETightBI-RY-0, which was a gift from Phil Sharp (Addgene plasmid # 31463) and cloned at NheI-HindIII restriction sites.

Codon optimized β-arrestin1-TEV219 was initially synthesized (Bio Basic) and cloned in pcDNA3.1+ (Thermo Fisher Scientific). β-arrestin2 was PCR amplified from pLX317-β-arrestin2 and the FYVE domain from pLX317-ZFYVE16 (Endofin) both from the MISSION TRC3 Human LentiORF Collection (MilliporeSigma). Both were cloned into the β-arrestin1-TEV219/pcDNA3.1+ at HindIII-BamHI sites. The PURO resistance gene in the all-in-one lentivector pCDH-CuO-MCS-EF1α-CymR-T2A-PURO SparQ (System Biosciences, QM800A-1) was changed for the BLEO3 resistance using PCR amplification and restriction site cloning (EcoRI-SalI). β-arrestin1-TEV219, β-arrestin2-TEV219, and FYVE-TEV219 were PCR amplified from the pcDNA3.1+ plasmid and cloned at NheI-SwaI restriction sites. Sequence maps for the aforementioned constructs can be found in the Supplementary Information.

GPCR-RLuc8 constructs for BRET2 experiments were cloned by PCR amplifying RLuc8 and cloned into Tango constructs at AgeI-XbaI site. β-arrestin1-GFP2 and β-arrestin2-GFP2 were cloned by PCR amplifying GFP2 and cloned at BamHI-XbaI sites of β-arrestin1-TEV219 and β-arrestin2-TEV219 in pcDNA3.1+.

The Roth Lab PRESTO-Tango GPCR Kit was from Dr. Bryan Roth and is available through Addgene [www.addgene.org/kits/roth-gpcr-presto-tango/].

### Reporting summary

Further information on research design is available in the Nature Portfolio Reporting Summary linked to this article.

## Supplementary information

Supplementary Information

Reporting Summary

## Data Availability

The data that support this study are available from the corresponding authors upon request. All data generated or analyzed during this study, including data underlying Figs. 1–8 and all Supplementary Figures are provided as a Source Data file accessible at the Figshare repository [10.6084/m9.figshare.22802948]. Human Protein Atlas (HPA) RNA consensus tissue gene data (version 21.0 and Ensembl version 103.38.) used for the production of Fig. 8 was accessed at [https://www.proteinatlas.org/about/]. EMTA data compared in Supplementary Table 1, including Emax (in % of vehicle response) and absolute pEC50 values, was downloaded from [https://cdn.elifesciences.org/articles/74101/elife-74101-supp2-v2.xlsx].

## References

[1] Kroeze WK, Sheffler DJ, Roth BL (2003). G-protein-coupled receptors at a glance. J. Cell Sci..

[2] Pierce KL, Premont RT, Lefkowitz RJ (2002). Seven-transmembrane receptors. Nat. Rev. Mol. Cell Biol..

[3] Lynch, J. R. & Wang, J. Y. G protein-coupled receptor signaling in stem cells and cancer. *Int. J. Mol. Sci*. **17**, 707 (2016). 10.3390/ijms17050707 PMC4881529 27187360

[4] Ritter SL, Hall RA (2009). Fine-tuning of GPCR activity by receptor-interacting proteins. Nat. Rev. Mol. Cell Biol..

[5] Black JB, Premont RT, Daaka Y (2016). Feedback regulation of G protein-coupled receptor signaling by GRKs and arrestins. Semin. Cell Dev. Biol..

[6] Draper-Joyce C, Furness SGB (2019). Conformational transitions and the activation of heterotrimeric G proteins by G protein-coupled receptors. ACS Pharmacol. Transl. Sci..

[7] Rosenbaum DM, Rasmussen SGF, Kobilka BK (2009). The structure and function of G-protein-coupled receptors. Nature.

[8] Smith JS, Rajagopal S (2016). The β-Arrestins: multifunctional regulators of G protein-coupled receptors. J. Biol. Chem..

[9] Kohout TA, Lin F-T, Perry SJ, Conner DA, Lefkowitz RJ (2001). β-Arrestin 1 and 2 differentially regulate heptahelical receptor signaling and trafficking. Proc. Natl Acad. Sci..

[10] Tilley DG (2011). G protein-dependent and G protein-independent signaling pathways and their impact on cardiac function. Circ. Res..

[11] Thomsen ARB, Jensen DD, Hicks GA, Bunnett NW (2018). Therapeutic targeting of endosomal G-protein-coupled receptors. Trends Pharmacol. Sci..

[12] Van Koppen CJ, Jakobs KH (2004). Arrestin-independent internalization of G protein-coupled receptors. Mol. Pharmacol..

[13] Kroeze WK (2015). PRESTO-Tango as an open-source resource for interrogation of the druggable human GPCRome. Nat. Struct. Mol. Biol..

[14] Blau HM, Rossi FM (1999). Tet B or not tet B: advances in tetracycline-inducible gene expression. Proc. Natl Acad. Sci. USA..

[15] Jaisser F (2000). Inducible gene expression and gene modification in transgenic mice. J. Am. Soc. Nephrol.

[16] Liu B, Wang S, Brenner M, Paton JFR, Kasparov S (2008). Enhancement of cell-specific transgene expression from a Tet-Off regulatory system using a transcriptional amplification strategy in the rat brain. J. Gene Med..

[17] Vopálenský V (2008). Firefly luciferase gene contains a cryptic promoter. RNA.

[18] Parks TD, Howard ED, Wolpert TJ, Arp DJ, Dougherty WG (1995). Expression and purification of a recombinant tobacco etch virus NIa proteinase: biochemical analyses of the full-length and a naturally occurring truncated proteinase form. Virology.

[19] Kapust RB (2001). Tobacco etch virus protease: mechanism of autolysis and rational design of stable mutants with wild-type catalytic proficiency. Protein Eng..

[20] Seet L-F, Hong W (2001). Endofin, an endosomal FYVE domain protein. J. Biol. Chem..

[21] Mullick A (2006). The cumate gene-switch: a system for regulated expression in mammalian cells. BMC Biotechnol..

[22] Gould DJ, Chernajovsky Y (2004). Endogenous GATA factors bind the core sequence of the tetO and influence gene regulation with the tetracycline system. Mol. Ther..

[23] Cohen S, Dovrat S, Sarid R, Huberman E, Salzberg S (2005). JAK–STAT signaling involved in phorbol 12-myristate 13-acetate- and dimethyl sulfoxide-induced 2′−5′ oligoadenylate synthetase expression in human HL-60 leukemia cells. Leuk. Res..

[24] Pedranzini L (2006). Pyridone 6, A Pan-Janus–activated kinase inhibitor, induces growth inhibition of multiple myeloma cells. Cancer Res..

[25] Saucier C, Morris SJ, Albert PR (1998). Endogenous serotonin-2A and −2C receptors in Balb/c-3T3 cells revealed in serotonin-free medium: Desensitization and down-regulation by serotonin. Biochem. Pharmacol..

[26] Bousoik E, Montazeri Aliabadi H (2018). “Do We Know Jack” About JAK? A Closer Look at JAK/STAT Signaling Pathway. Front. Oncol..

[27] Alexander SPH (2021). THE CONCISE GUIDE TO PHARMACOLOGY 2021/22: G protein-coupled receptors. Br. J. Pharmacol..

[28] Seo SO, Schmidt-Dannert C (2019). Development of a synthetic cumate-inducible gene expression system for Bacillus. Appl. Microbiol. Biotechnol..

[29] Zeghal, M., Laroche, G. & Giguère, P. M. Parallel interrogation of β-arrestin2 recruitment for ligand screening on a GPCR-wide scale using PRESTO-Tango assay. *J. Vis. Exp.* **2020**, e60823 (2020). 10.3791/60823 32225148

[30] Rinne M, Tanoli ZUR, Khan A, Xhaard H (2019). Cartography of rhodopsin-like G protein-coupled receptors across vertebrate genomes. Sci. Rep. 2019 91.

[31] Zhao X (2008). A homogeneous enzyme fragment complementation-based beta-arrestin translocation assay for high-throughput screening of G-protein-coupled receptors. J. Biomol. Screen..

[32] Moo EVon, van Senten JR, Bräuner-Osborne H, Møller TC (2021). Arrestin-dependent and -independent internalization of G protein-coupled receptors: methods, mechanisms, and implications on cell signaling. Mol. Pharmacol..

[33] Avet, C. et al. Effector membrane translocation biosensors reveal G protein and Parrestin coupling profiles of 100 therapeutically relevant GPCRs. *Elife* **11**, e74101 (2022). 10.7554/eLife.74101 PMC9005190 35302493

[34] Zhou, Q. et al. Common activation mechanism of class a GPCRs. *Elife* **8**, e50279 (2019). 10.7554/eLife.50279 PMC6954041 31855179

[35] Lu, S., Jang, W., Inoue, A. & Lambert, N. A. Constitutive G protein coupling profiles of understudied orphan GPCRs. *PLoS One* **16**, e0247743 (2021). 10.1371/journal.pone.0247743 PMC8062009 33886554

[36] Watkins LR, Orlandi C (2021). In vitro profiling of orphan G protein coupled receptor (GPCR) constitutive activity. Br. J. Pharmacol..

[37] Oakley RH, Laporte SA, Holt JA, Caron MG, Barak LS (2000). Differential affinities of visual arrestin, beta arrestin1, and beta arrestin2 for G protein-coupled receptors delineate two major classes of receptors. J. Biol. Chem..

[38] Berg KA, Clarke WP (2018). Making sense of pharmacology: inverse agonism and functional selectivity. Int. J. Neuropsychopharmacol..

[39] Harding SD (2022). The IUPHAR/BPS guide to PHARMACOLOGY in 2022: curating pharmacology for COVID-19, malaria and antibacterials. Nucleic Acids Res..

[40] Simcocks AC (2019). Atypical cannabinoid ligands O-1602 and O-1918 administered chronically in diet-induced obesity. Endocr. Connect.

[41] Thiele S, Mungalpara J, Steen A, Rosenkilde MM, Våbenø J (2014). Determination of the binding mode for the cyclopentapeptide CXCR4 antagonist FC131 using a dual approach of ligand modifications and receptor mutagenesis. Br. J. Pharmacol..

[42] Magalhaes AC, Dunn H, Ferguson SSG (2012). Regulation of GPCR activity, trafficking and localization by GPCR-interacting proteins. Br. J. Pharm..

[43] Yang Z (2017). Phosphorylation of G protein-coupled receptors: from the barcode hypothesis to the flute model. Mol. Pharmacol..

[44] Drube, J. et al. GRK2/3/5/6 knockout: the impact of individual GRKs on arrestin-binding and GPCR regulation. *bioRxiv*10.1101/2021.02.12.430971 (2021).

[45] Busillo JM (2010). Site-specific phosphorylation of CXCR4 is dynamically regulated by multiple kinases and results in differential modulation of CXCR4 signaling. J. Biol. Chem..

[46] Białopiotrowicz E (2018). Microenvironment-induced PIM kinases promote CXCR4-triggered mTOR pathway required for chronic lymphocytic leukaemia cell migration. J. Cell. Mol. Med..

[47] Decker S (2014). PIM kinases are essential for chronic lymphocytic leukemia cell survival (PIM2/3) and CXCR4-mediated microenvironmental interactions (PIM1). Mol. Cancer Ther..

[48] Shintani Y (2018). β-Arrestin1 and 2 differentially regulate PACAP-induced PAC1 receptor signaling and trafficking. PLoS One.

[49] Vibhuti A, Gupta K, Subramanian H, Guo Q, Ali H (2011). Distinct and shared roles of β-Arrestin-1 and β-Arrestin-2 on the regulation of C3a receptor signaling in human mast cells. PLoS One.

[50] Levoye A (2015). A broad G protein-coupled receptor internalization assay that combines SNAP-tag labeling, diffusion-enhanced resonance energy transfer, and a highly emissive terbium cryptate. Front. Endocrinol..

[51] Moriya H (2015). Quantitative nature of overexpression experiments. Mol. Biol. Cell.

[52] Peterson YK, Luttrell LM (2017). The diverse roles of arrestin scaffolds in G protein–coupled receptor signaling. Pharmacol. Rev..

[53] Miljuš, T. et al. Diverse chemotypes drive biased signaling by cannabinoid receptors. *bioRxiv*10.1101/2020.11.09.375162, (2020).

[54] Gurevich EV, Benovic JL, Gurevich VV (2004). Arrestin2 expression selectively increases during neural differentiation. J. Neurochem.

[55] Komolov KE, Benovic JL (2018). G protein-coupled receptor kinases: past, present and future. Cell. Signal..

[56] Kumari P (2017). Core engagement with β-arrestin is dispensable for agonist-induced vasopressin receptor endocytosis and ERK activation. Mol. Biol. Cell.

[57] Tobin AB (2008). G-protein-coupled receptor phosphorylation: where, when and by whom. Br. J. Pharm..

[58] Stenmark H, Aasland R, Driscoll PC (2002). The phosphatidylinositol 3-phosphate-binding FYVE finger. FEBS Lett..

[59] Roth BL (2013). Impossible or merely difficult? Two grand challengesfrom a biologist’s perspective. ACS Med. Chem. Lett..

[60] Ngo T (2016). Identifying ligands at orphan GPCRs: current status using structure-based approaches. Br. J. Pharmacol..

[61] Mary S (2012). Ligands and signaling proteins govern the conformational landscape explored by a G protein-coupled receptor. Proc. Natl Acad. Sci. USA.

[62] Azzi M (2003). Beta-arrestin-mediated activation of MAPK by inverse agonists reveals distinct active conformations for G protein-coupled receptors. Proc. Natl Acad. Sci. USA.

[63] Williams C, Hill SJ (2009). GPCR signaling: understanding the pathway to successful drug discovery. Methods Mol. Biol..

[64] Huber M, Brabec M, Bayer N, Blaas D, Fuchs R (2001). Elevated endosomal pH in HeLa cells overexpressing mutant dynamin can affect infection by pH-sensitive viruses. Traffic.

[65] Casamento A, Boucrot E (2020). Molecular mechanism of fast endophilin-mediated endocytosis. Biochem. J..

[66] Orsini MJ, Benovic JL (1998). Characterization of dominant negative arrestins that inhibit beta2-adrenergic receptor internalization by distinct mechanisms. J. Biol. Chem..

[67] Al-Hasani R, Bruchas MR (2011). Molecular mechanisms of opioid receptor-dependent signaling and behavior. Anesthesiology.

[68] Chung G, Kim SJ (2017). Sustained activity of metabotropic glutamate receptor: homer, arrestin, and beyond. Neural Plast..

[69] Rankin ML (2006). The D1 dopamine receptor is constitutively phosphorylated by G protein-coupled receptor kinase 4. Mol. Pharmacol..

[70] Atwood BK, Lopez J, Wager-Miller J, Mackie K, Straiker A (2011). Expression of G protein-coupled receptors and related proteins in HEK293, AtT20, BV2, and N18 cell lines as revealed by microarray analysis. BMC Genom..

[71] Li L (2015). G protein-coupled receptor kinases of the GRK4 protein subfamily phosphorylate inactive G protein-coupled receptors (GPCRs). J. Biol. Chem..

[72] Longo PA, Kavran JM, Kim MS, Leahy DJ (2013). Transient mammalian cell transfection with polyethylenimine (PEI). Methods Enzymol..

