# Supplementary material for: Profiling of basal and ligand-dependent GPCR activities by means of a polyvalent cell-based high-throughput platform
Source: Nat Commun. 2023 Jul 5;14:3684. doi: 10.1038/s41467-023-39132-x (PMC10322906; doi:10.1038/s41467-023-39132-x)
Supplement: Supplementary file 1 — Supplementary Information [file 41467_2023_39132_MOESM1_ESM.pdf]

Supplementary Information for

Profiling of basal and ligand-dependent GPCR activities by means of a polyvalent cell-based high-throughput platform

Manel Zeghal[1], Geneviève Laroche[1], Julia Douglas Freitas[1], Rebecca Wang[1], Patrick M. Giguère[1,2*]

[1] Department of Biochemistry, Microbiology and Immunology, University of Ottawa, Ottawa, ON, K1H8M5, Canada
[2] Brain and Mind Research Institute, University of Ottawa, Ottawa, ON, K1H8M5, Canada
*Correspondence to Patrick M. Giguère. Email: patrick.giguere@uottawa.ca

Supplemental Figures

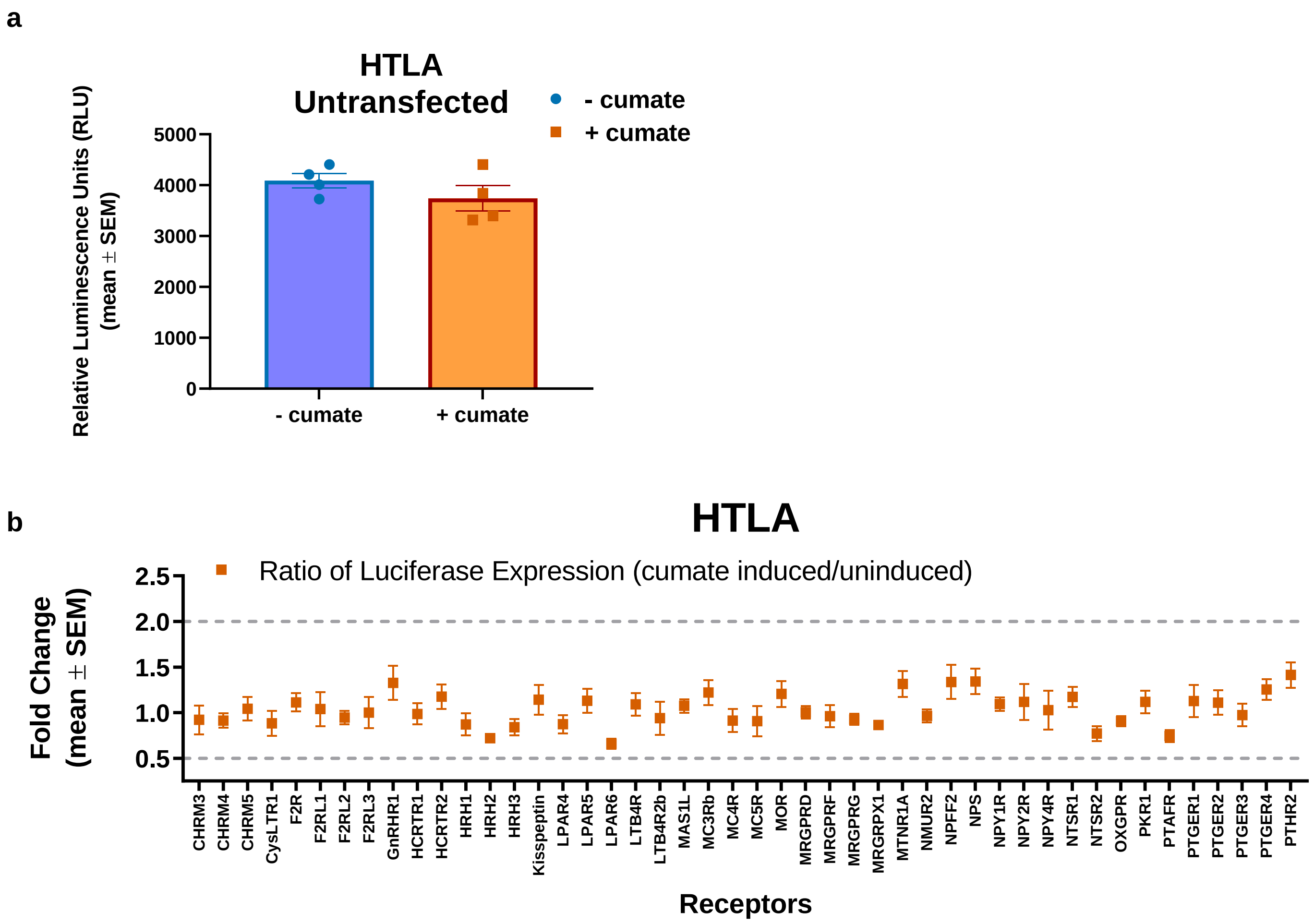

Supplementary Figure 1. Assessment of the effect of cumate in the PRESTO-Tango (cumate-independent system). To confirm that cumate itself does not possess any agonistic or antagonistic properties in a cumate-independent system, HTLA cells from the PRESTO-Tango platform were plated in the presence and absence of cumate (30 µg/mL) (a) and were transfected with a panel of diverse GPCR Tango constructs (b). 96 hours following its initial addition, fold changes in basal arrestin recruitment were calculated between the wells in the absence or presence of cumate. Data are presented as mean values, with error bars representing SEM (Supplementary Fig 1A: n = 12, with 3 technical replicates from 4 biological samples; Supplementary Fig 1B: n = 4, with 4 technical replicates from one biological sample). Generic receptor codes refer to the GPCR-Tango constructs.

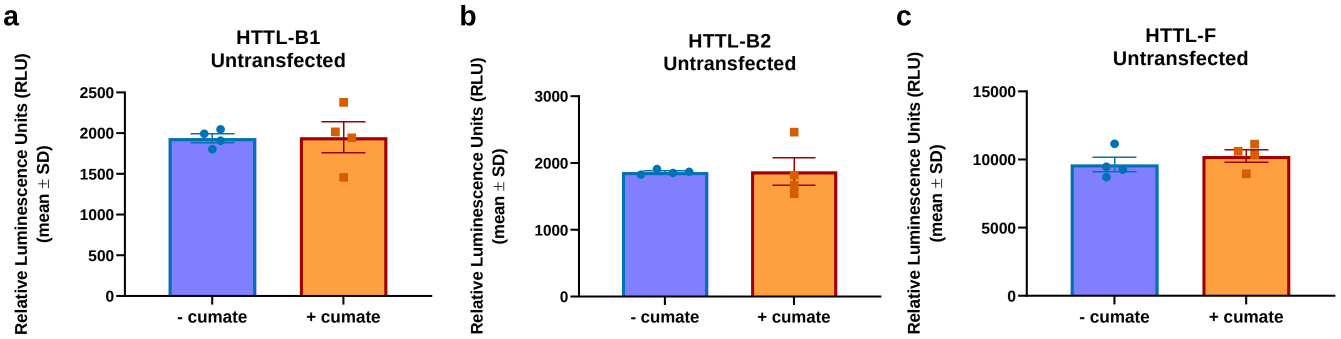

Supplementary Figure 2. Baseline signal of Tango-Trio cell lines. (A-C) HTTL-B1, HTTL-B2, and HTTL-F cells were plated in the presence or absence of cumate (30 µg/mL), which was maintained throughout (totalling approximately 72 hours). As per standard protocol, cells were serum starved for the last 24 hours of the experiment, and luminescence was subsequently read to compare the differences in baseline signal of untransfected cells. Data are presented as mean values, with error bars representing SD (n = 12, with 3 technical replicates from 4 biological samples).

a

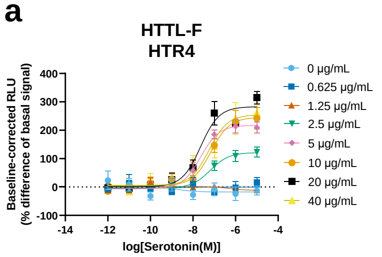

	EC50	Fold
0 µg/mL	1.522e-009	0.7986
0.625 µg/mL	6.476e-009	1.032
1.25 µg/mL	5.666e-007	0.8598
2.5 µg/mL	6.493e-008	2.302
5 µg/mL	2.277e-008	3.396
10 µg/mL	7.106e-008	3.305
20 µg/mL	2.281e-008	3.797
40 µg/mL	5.685e-008	3.347

b

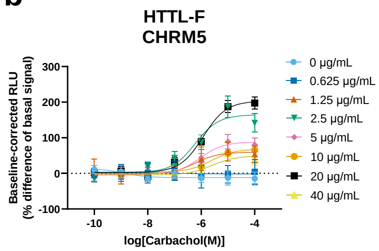

	EC50	Fold
0 µg/mL	1.261e-009	0.7768
0.625 µg/mL	2.616e-007	0.9735
1.25 µg/mL	5.848e-007	1.630
2.5 µg/mL	5.948e-007	2.585
5 µg/mL	9.187e-007	1.923
10 µg/mL	2.424e-006	1.626
20 µg/mL	1.187e-006	2.952
40 µg/mL	2.552e-006	1.560

c

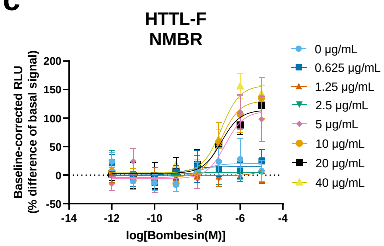

	EC50	Fold
0 µg/mL	1.333e-008	1.255
0.625 µg/mL	2.705e-009	1.146
1.25 µg/mL	0.1617	Unstable
2.5 µg/mL	2.063e-009	1.053
5 µg/mL	2.005e-007	2.256
10 µg/mL	1.295e-007	2.403
20 µg/mL	1.348e-007	2.077
40 µg/mL	1.316e-007	2.475

d

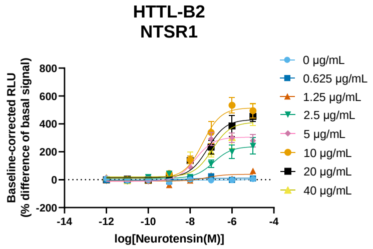

	EC50	Fold
0 µg/mL	0.03127	Unstable
0.625 µg/mL	5.844e-009	1.140
1.25 µg/mL	6.036e-008	1.545
2.5 µg/mL	1.300e-007	3.099
5 µg/mL	1.779e-008	4.610
10 µg/mL	4.025e-008	5.504
20 µg/mL	6.903e-008	4.617
40 µg/mL	1.153e-007	4.251

e

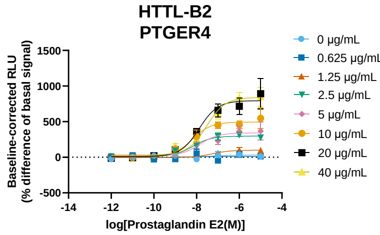

	EC50	Fold
0 µg/mL	5.024e-008	1.309
0.625 µg/mL	2.567e-009	1.143
1.25 µg/mL	5.236e-008	2.099
2.5 µg/mL	6.696e-009	3.636
5 µg/mL	1.387e-008	3.731
10 µg/mL	6.337e-009	5.511
20 µg/mL	1.327e-008	8.103
40 µg/mL	2.933e-008	7.132

f

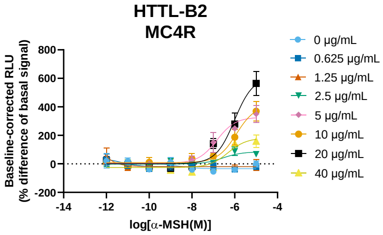

	EC50	Fold
0 µg/mL	2.220e-009	0.6302
0.625 µg/mL	4.753e-012	0.5670
1.25 µg/mL	2.018e-015	Unstable
2.5 µg/mL	2.823e-007	1.864
5 µg/mL	1.459e-007	4.093
10 µg/mL	1.204e-006	4.687
20 µg/mL	9.759e-007	7.012
40 µg/mL	4.858e-007	3.775

g

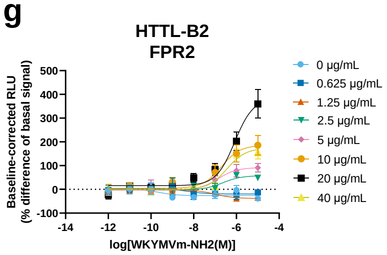

	EC50	Fold
0 µg/mL	2.372e-010	0.7269
0.625 µg/mL	5.153e-009	0.7824
1.25 µg/mL	1.013e-007	0.6304
2.5 µg/mL	2.296e-007	1.621
5 µg/mL	1.377e-007	1.970
10 µg/mL	1.981e-007	2.739
20 µg/mL	8.317e-007	4.177
40 µg/mL	3.618e-007	2.758

h

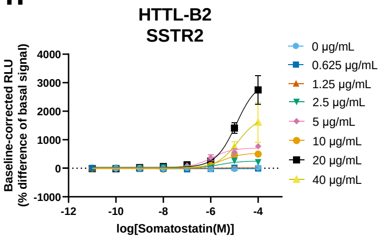

	EC50	Fold
0 µg/mL	1.306e-007	0.9568
0.625 µg/mL	1.517e-010	0.7628
1.25 µg/mL	2.058e-006	1.253
2.5 µg/mL	2.399e-006	3.481
5 µg/mL	1.086e-006	7.144
10 µg/mL	2.812e-006	7.369
20 µg/mL	1.202e-005	24.43
40 µg/mL	1.403e-005	16.52

i

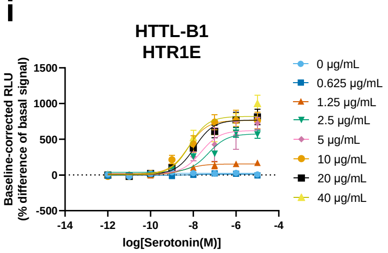

	EC50	Fold
0 µg/mL	6.643e-011	1.347
0.625 µg/mL	9.436e-009	1.154
1.25 µg/mL	4.096e-009	2.500
2.5 µg/mL	5.166e-008	4.929
5 µg/mL	2.304e-008	6.100
10 µg/mL	5.882e-009	7.516
20 µg/mL	1.179e-008	7.945
40 µg/mL	6.688e-009	8.827

j

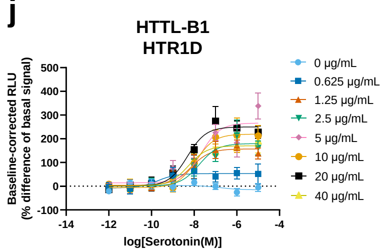

	EC50	Fold
0 µg/mL	1.711e-007	0.8261
0.625 µg/mL	1.945e-010	1.660
1.25 µg/mL	7.274e-009	2.478
2.5 µg/mL	1.703e-008	2.768
5 µg/mL	2.194e-008	3.205
10 µg/mL	1.199e-008	3.403
20 µg/mL	4.799e-009	3.491
40 µg/mL	5.383e-009	2.669

k

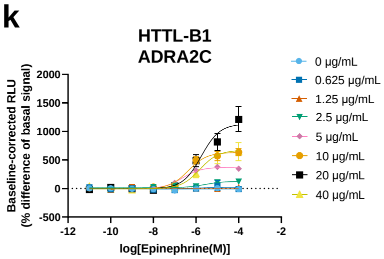

	EC50	Fold
0 µg/mL	6.870e-010	0.8535
0.625 µg/mL	1.161e-006	1.332
1.25 µg/mL	6.461e-016	Unstable
2.5 µg/mL	2.674e-006	2.013
5 µg/mL	2.320e-007	4.982
10 µg/mL	4.337e-007	7.992
20 µg/mL	1.761e-006	12.92
40 µg/mL	1.491e-006	8.250

l

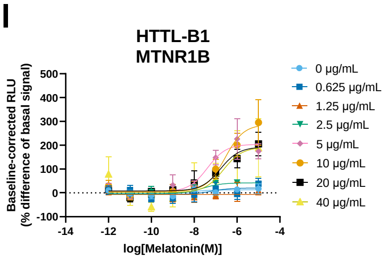

	EC50	Fold
0 µg/mL	8.045e-009	1.165
0.625 µg/mL	6.530e-008	1.287
1.25 µg/mL	90711713	Unstable
2.5 µg/mL	2.453e-008	1.478
5 µg/mL	4.129e-008	2.906
10 µg/mL	2.752e-007	3.653
20 µg/mL	1.686e-007	2.702
40 µg/mL	2.283e-007	2.870

Supplementary Figure 3. Changes in baseline signals and fold windows due to cumate induction. HTTL-B1, HTTL-B2 and HTTL-F cells were plated in increasing concentrations of cumate (0, 0.625, 1.25, 2.5, 5, 10, 20, and 40 µg/mL), which was maintained throughout the entire experiment, and were transfected with select GPCRs: HTR4 (a), CHRM5 (b), and NMBR (c) in HTTL-F; NTSR1 (d), PTGER4 (e), MC4R (f), FPR2 (g), and SSTR2 (h) in HTTL-B2; HTR1E (i), HTR1D (j), ADRA2C (k), and MTNR1B (l) in HTTL-B1. Transfected cells were stimulated with the receptor specific agonist and dose-response curves were built using XY analysis for non-linear regression curve and the 3-parameters dose-response stimulation function, followed by baseline correction. Data are presented as mean values, with error bars representing SEM (n = 3, with three technical replicates from one biological sample). Generic receptor codes refer to the GPCR-Tango constructs.

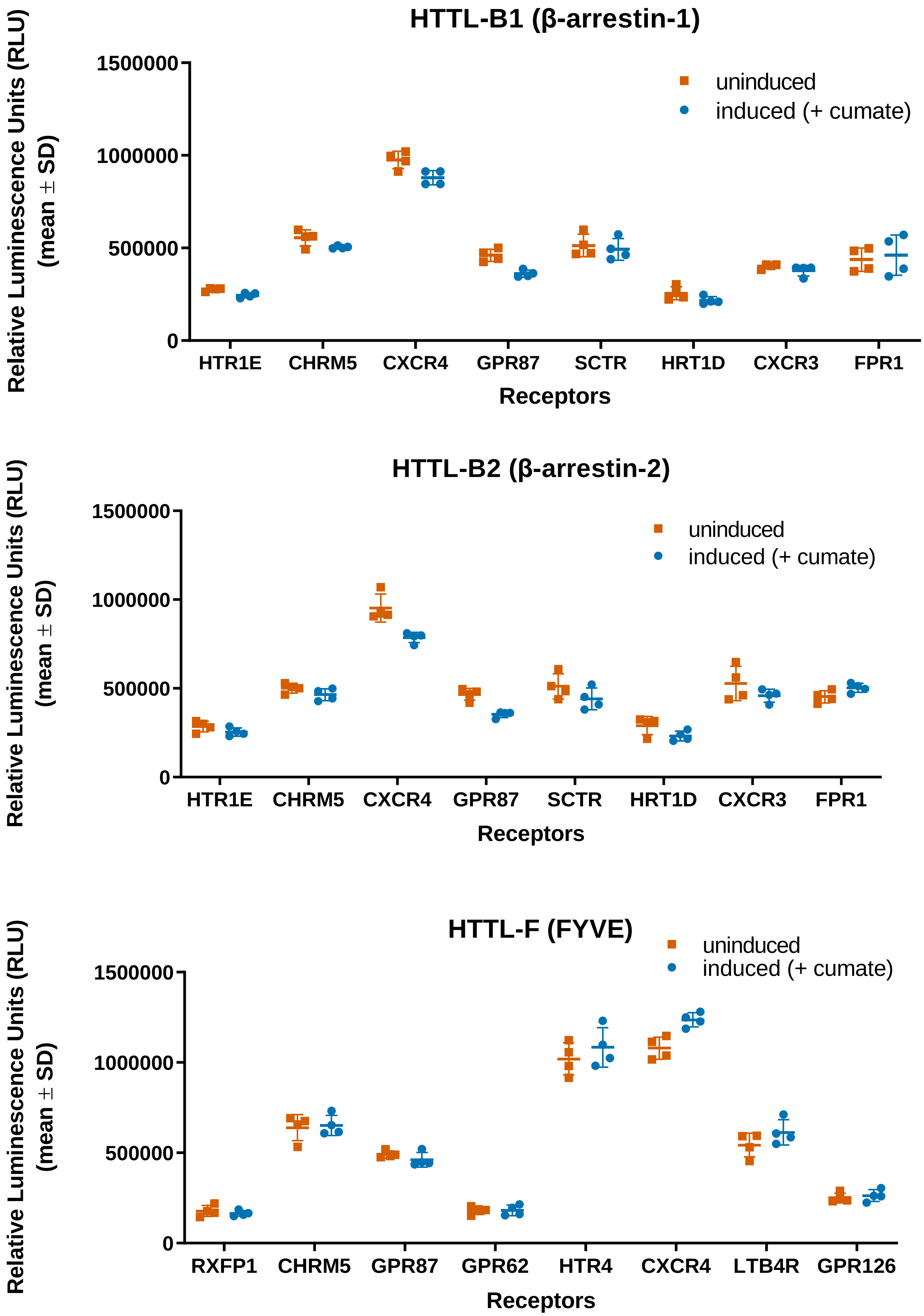
HTTL-B1 (β-arrestin-1)
Relative Luminescence Units (RLU) (mean ± SD)
1500000
1000000
500000
0
uninduced
induced (+ cumate)
HTR1E
CHRM5
CXCR4
GPR87
SCTR
HRT1D
CXCR3
FPR1
Receptors
HTTL-B2 (β-arrestin-2)
Relative Luminescence Units (RLU) (mean ± SD)
1500000
1000000
500000
0
uninduced
induced (+ cumate)
HTR1E
CHRM5
CXCR4
GPR87
SCTR
HRT1D
CXCR3
FPR1
Receptors
HTTL-F (FYVE)
Relative Luminescence Units (RLU) (mean ± SD)
1500000
1000000
500000
0
uninduced
induced (+ cumate)
RXFP1
CHRM5
GPR87
GPR62
HTR4
CXCR4
LTB4R
GPR126
Receptors

Supplementary Figure 4. Receptor surface expression following cumate induction of fusion protein expression HTTL-B1, HTTL-B2 and HTTL-F were plated in the presence or absence of cumate (30 µg/mL) and were transfected with a select number of positive GPCR hits from the constitutive HTS. ELISA experiments were subsequently carried out on transfected cells to determine receptor surface expression. Data are presented as mean values, with error bars representing SD (n = 4, with four technical replicates from one biological sample). Generic receptor codes refer to the GPCR-Tango constructs.

a

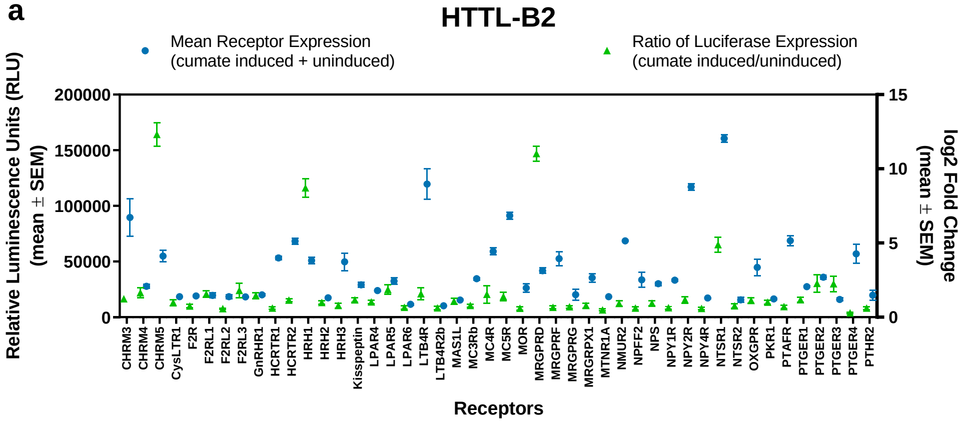

Pearson r	
r	0.2084
95% confidence interval	-0.08714 to 0.4703
R squared	0.04344
P value	
P (two-tailed)	0.1645
P value summary	ns
Significant? (alpha = 0.05)	No
Number of XY Pairs	46

b

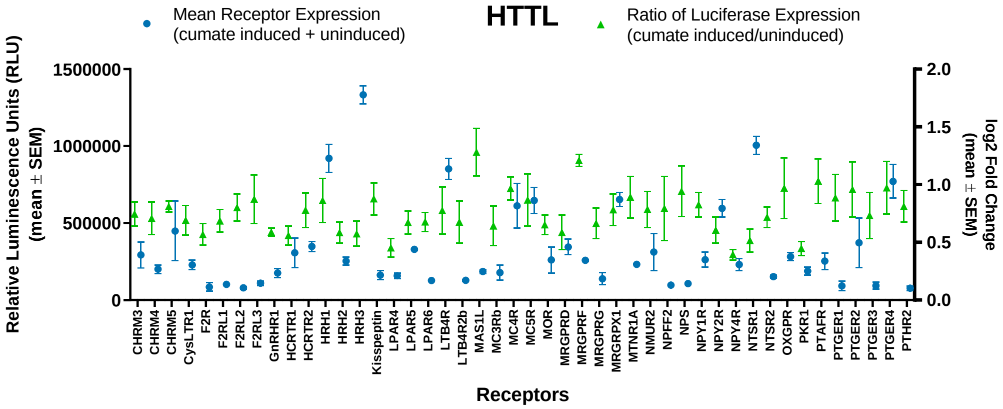

Pearson r	
r	-0.03125
95% confidence interval	-0.3187 to 0.2614
R squared	0.0009767
P value	
P (two-tailed)	0.8366
P value summary	ns
Significant? (alpha = 0.05)	No
Number of XY Pairs	46

c

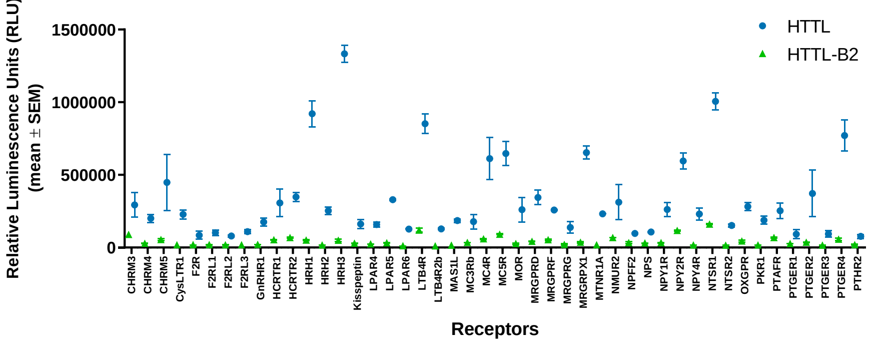

Pearson r	
r	0.6659
95% confidence interval	0.4656 to 0.8013
R squared	0.4434
P value	
P (two-tailed)	<0.0001
P value summary	****
Significant? (alpha = 0.05)	Yes
Number of XY Pairs	46

d

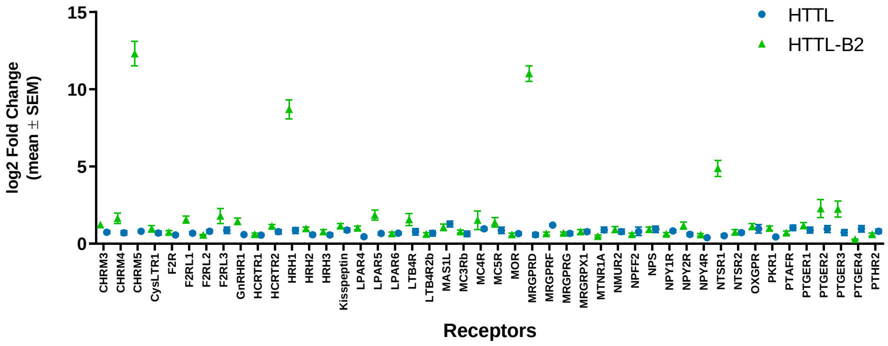

Pearson r	
r	-0.04866
95% confidence interval	-0.3342 to 0.2451
R squared	0.002368
P value	
P (two-tailed)	0.7481
P value summary	ns
Significant? (alpha = 0.05)	No
Number of XY Pairs	46

Supplementary Figure 5. Comparison of receptor expression and constitutive activity across panel of GPCRs. HTTL-B2 (a) and HTTL (b) were plated in the presence or absence of cumate (30 µg/mL) and were transfected with a panel of GPCRs with a varying range of constitutive activities. ELISA experiments were subsequently carried out on transfected cells to determine receptor surface expression, and log2 fold changes in constitutive arrestin recruitment were calculated between the wells in the absence or presence of cumate. Mean receptor expression (c) and log2 fold changes in constitutive arrestin recruitment (d) were also compared between the HTTL and HTTL-B2 cell lines. Pearson correlation coefficients (r) and corresponding p values (two-tailed) were computed between data sets using GraphPad Prism. Data are presented as mean values, with error bars representing SEM (n = 4, with four technical replicates from one biological sample). Generic receptor codes refer to the GPCR-Tango constructs.

a

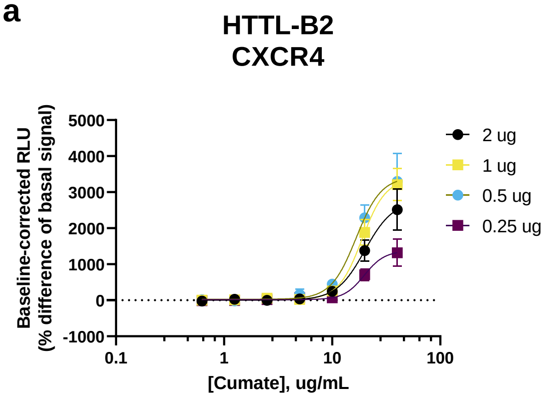

	2 ug	1 ug	0.5 ug	0.25 ug
EC50	20.05	18.88	16.67	19.82
Fold	28.29	30.91	27.49	12.56

b

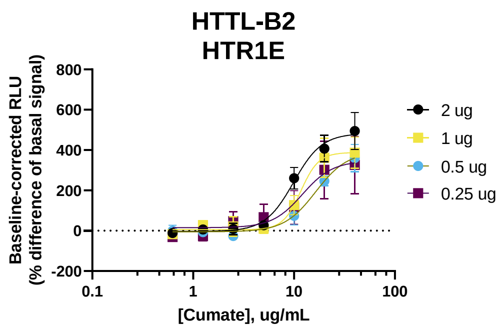

	2 ug	1 ug	0.5 ug	0.25 ug
EC50	9.896	11.68	16.41	12.12
Fold	6.026	4.646	5.200	3.877

c

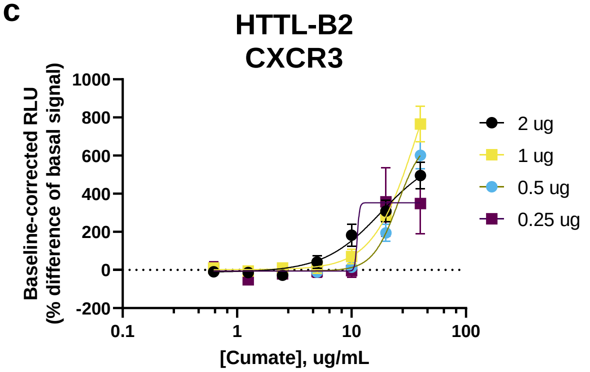

	2 ug	1 ug	0.5 ug	0.25 ug
EC50	18.09	37.73	25.45	11.23
Fold	8.086	15.09	8.477	4.811

d

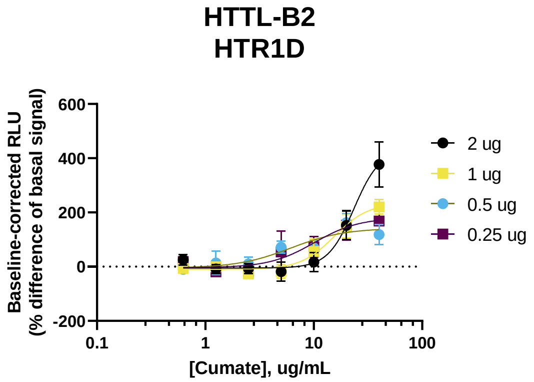

	2 ug	1 ug	0.5 ug	0.25 ug
EC50	23.47	15.51	6.125	10.10
Fold	5.594	3.706	2.548	2.920

Supplementary Figure 6. Titration of GPCR Tango DNA and consequent effects on constitutive activity. HTTL-B2 cells were seeded in 6-well plates and transfected with various amount of CXCR4 (a), HTR1E (b), CXCR3 (c), and HTR1D (d) Tango DNA, ranging from 0.25 ug – 2 ug total per well. Transfected cells were stimulated with cumate, and dose- response curves were built using XY analysis for non-linear regression curve and the 4-parameters dose-response stimulation function, followed by baseline correction. Data are presented as mean values, with error bars representing SEM (n = 3, with three technical replicates from one biological sample). Generic receptor codes refer to the GPCR-Tango constructs.

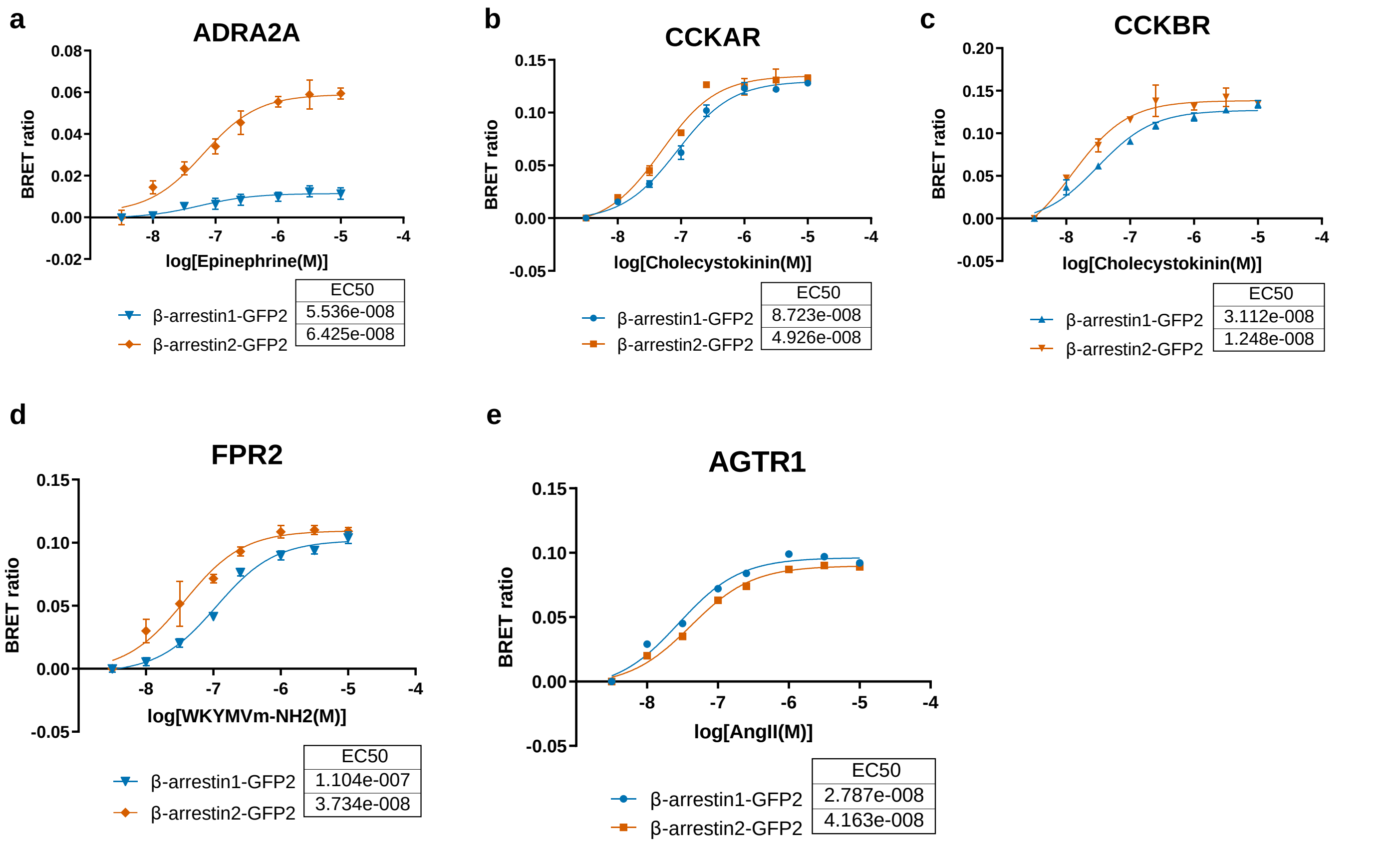

Supplementary Figure 7. Orthogonal determination of arrestin isoform selectivity using BRET2. HEK293T cells were transfected with β-arrestin1/2-GFP2, as well as the following GPCRs: ADRA2A (a), CCKAR (b), CCKBR (c), FPR2 (d), AGTR1 (e). Following transfection, cells were stimulated with serial dilutions of selective agonist, and read with 405 nm (RLuc8-Coelenterazine 400a) and 500 nm (GFP2) emission filters. Dose- response curves were built using XY analysis for non-linear regression curve and the 3-parameters dose-response stimulation function, followed by baseline correction. Data are presented as mean values, with error bars representing SEM (n = 2, with two technical replicates from one biological sample). Generic receptor codes refer to the GPCR-Rluc8 constructs.

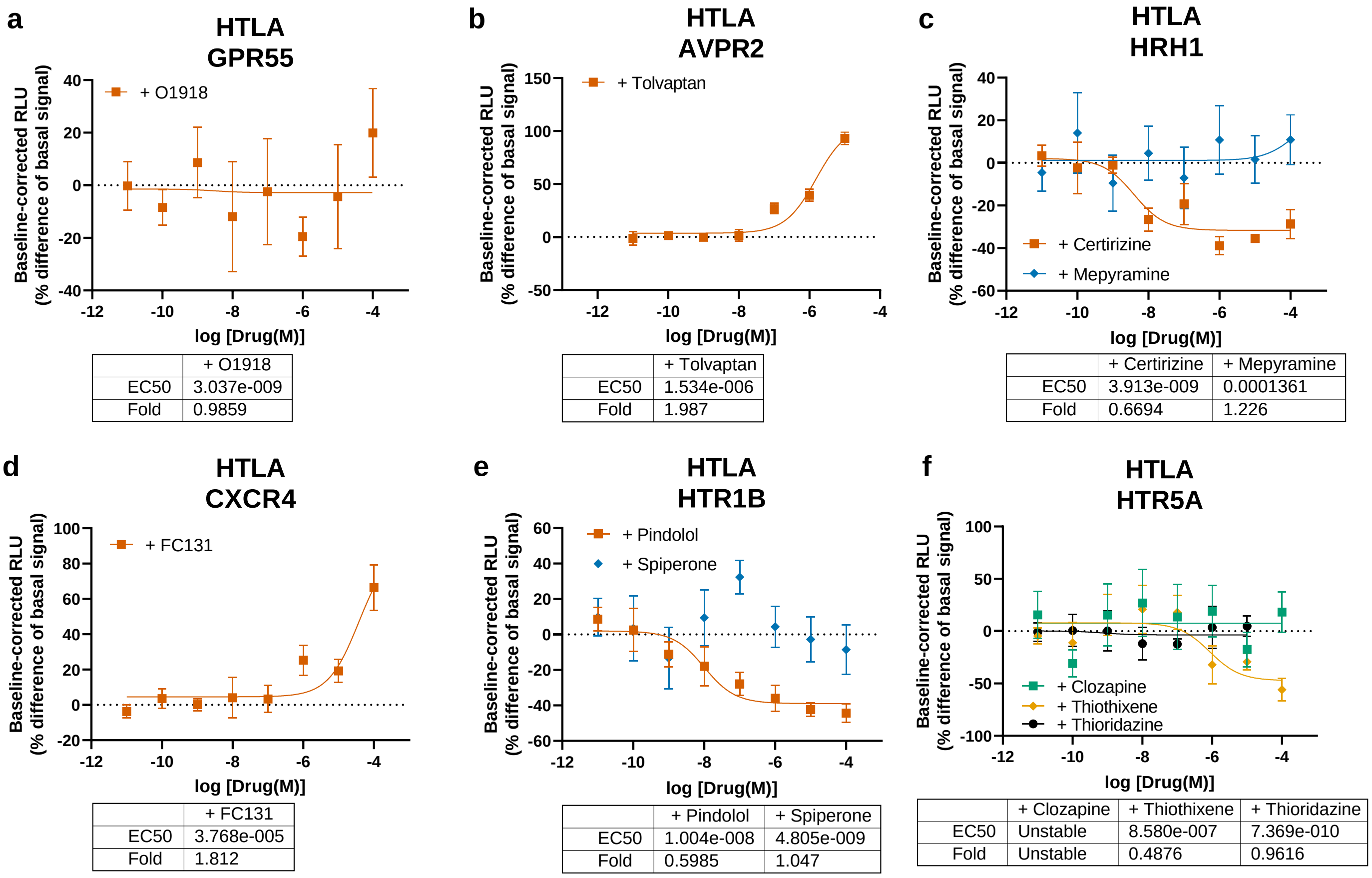

	+ O1918
EC50	3.037e-009
Fold	0.9859

	+ Tolvaptan
EC50	1.534e-006
Fold	1.987

	+ Certirizine	+ Mepyramine
EC50	3.913e-009	0.0001361
Fold	0.6694	1.226

	+ FC131
EC50	3.768e-005
Fold	1.812

	+ Pindolol	+ Spiperone
EC50	1.004e-008	4.805e-009
Fold	0.5985	1.047

	+ Clozapine	+ Thiothixene	+ Thioridazine
EC50	Unstable	8.580e-007	7.369e-010
Fold	Unstable	0.4876	0.9616

Supplementary Figure 8. Orthogonal characterization of select inverse agonists and antagonists using PRESTO-Tango. HTLA cells were transfected with GPCRs exhibiting strong constitutive arrestin recruitment from the primary Tango-Trio screen, serving to validate the findings shown in Figure 7. Transfected cells were stimulated with a dose-response curve using the following inverse agonists/antagonists: O-1918 at GPR55 (a), Tolvaptan at AVPR2 (b), Cetirizine and Mepyramine at HRH1 (c), FC131 at CXCR4 (d), Pindolol and Spiperone at HTR1E (e), and Clozapine, Thiothexene, and Thioridazine at HTR5A (f). Stimulation curves were built using XY analysis for non-linear regression curve and the 3-parameters dose-response stimulation function, followed by baseline correction. Data are presented as mean values, with error bars representing SEM (Supplementary Figs 8a, 8c-f: n = 3, with three technical replicates from one biological sample; Supplementary Fig 8b: n = 6, with three technical replicates from two biological samples). Generic receptor codes refer to the GPCR-Tango constructs.

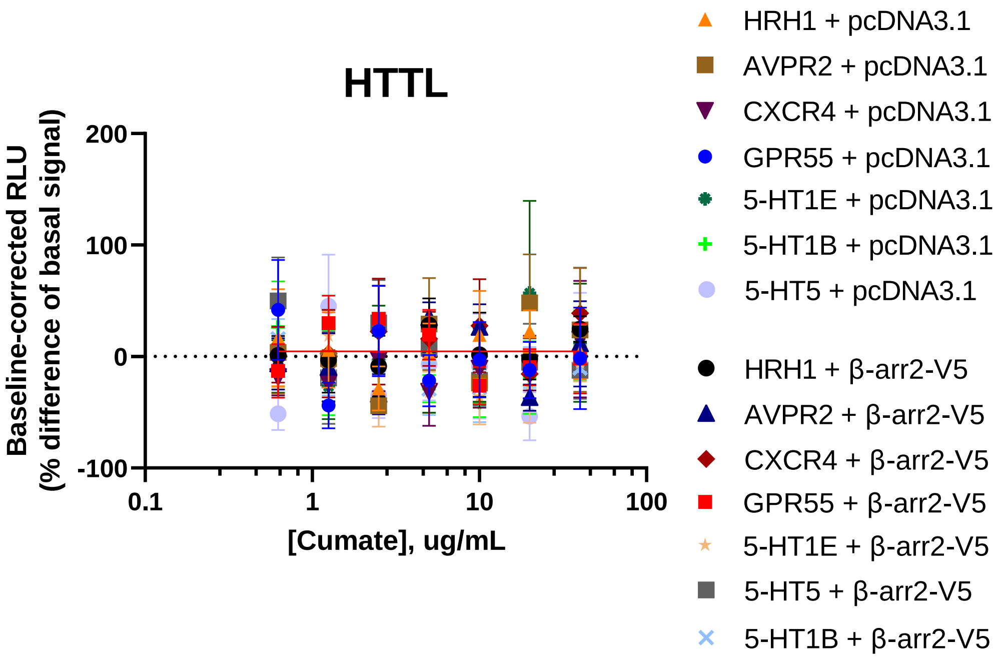

Supplementary Figure 9. Expression of GPCR-Tango constructs in the absence of TEV protease. HTTL cells were co-transfected with select GPCR constructs (tested with antagonists/inverse agonists from Fig 7), and either β-arrestin-2 (not tagged to TEV protease) or pcDNA3.1 as a control. Transfected cells were stimulated as a cumate dose-response, and stimulation curves were built using XY analysis for non-linear regression curve and the 4-parameters dose-response stimulation function, followed by baseline correction. Data are presented as mean values, with error bars representing SEM (n = 3, with three technical replicates from one biological sample). Generic receptor codes refer to the GPCR-Tango constructs.

a

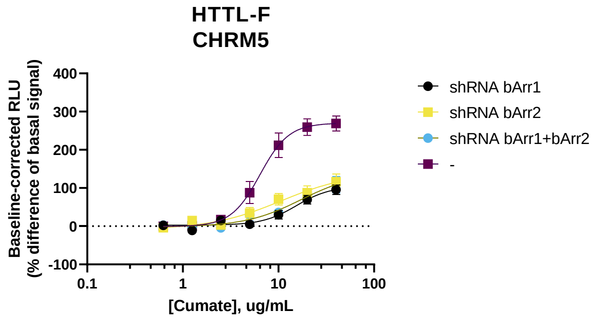

	EC50	logEC50	Span	Fold
shRNA bArr1	15.38	1.187	459303	1.993
shRNA bArr2	10.60	1.025	669083	2.622
shRNA bArr1+bArr2	17.20	1.236	564353	2.461
-	6.405	0.8065	139806	3.725

b

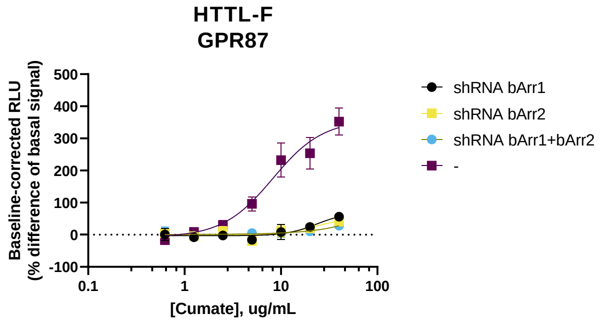

	EC50	logEC50	Span	Fold
shRNA bArr1	23.39	1.369	30849	1.744
shRNA bArr2	21.66	1.336	29759	1.513
shRNA bArr1+bArr2	557592	5.746	Unstable	Unstable
-	8.221	0.9149	48577	4.983

c

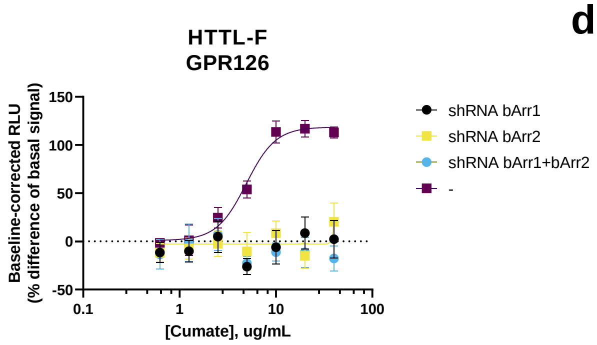

	EC50	logEC50	Span	Fold
shRNA bArr1	3.069	0.4871	30035	1.651
shRNA bArr2	42.20	1.625	Unstable	Unstable
shRNA bArr1+bArr2	3.419	0.5339	21766	1.486
-	4.921	0.6920	38098	2.169

d

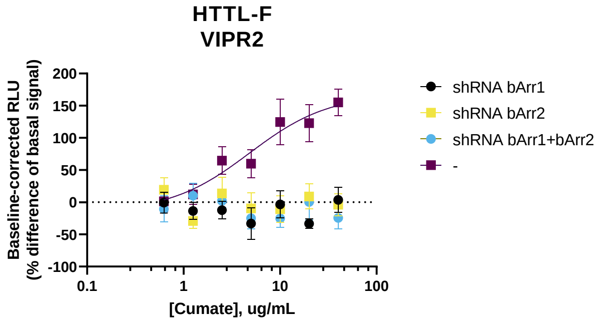

	EC50	logEC50	Span	Fold
shRNA bArr1	8.957	0.9522	63180	1.713
shRNA bArr2	4.508	0.6540	46960	1.685
shRNA bArr1+bArr2	3.386	0.5297	43671	1.472
-	4.821	0.6831	57025	3.172

e

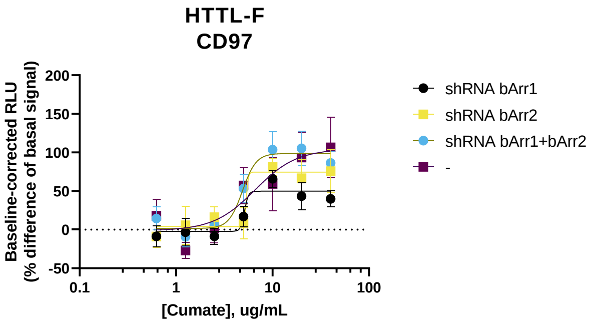

	EC50	logEC50	Span	Fold
shRNA bArr1	5.126	0.7098	59654	1.532
shRNA bArr2	5.352	0.7285	71885	1.678
shRNA bArr1+bArr2	4.878	0.6883	94061	1.956
-	6.606	0.8200	67253	2.072

f

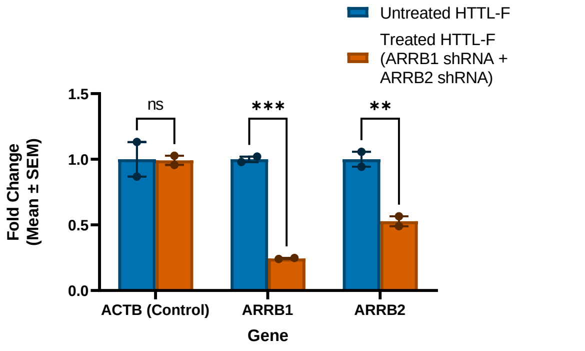

	ACTB (Control)	ARRB1	ARRB2
P value	0.930008	0.000147	0.001847
Mean of Untreated HTTL-F	1.000	1.000	1.000
Mean of Column B	0.9918	0.2444	0.5285
Difference	0.008163	0.7556	0.4715
SE of difference	0.08913	0.08913	0.08913
t ratio	0.09159	8.477	5.290
df	6.000	6.000	6.000
Adjusted P Value	0.930008	0.000442	0.003691

Supplementary Figure 10. Internalization of GPCRs in HTTL-F following β-arrestin-1 and β-arrestin-2 knockdown. Lentiviral β-arrestin-1 and -2 shRNA plasmids were transfected in HEK293T cells, along with psPAX2 and VSV-G vectors. The medium was replaced the following day with complete fresh medium, and lentiviral shRNA medium was collected following 48 hours transfection. For the knockdown experiment, HTTL-F cells were seeded in either complete medium or in the previously prepared lentiviral β-arrestin-1 and -2 shRNA medium (combined at a 1:1 ratio), with infection of cells facilitated with polybrene at 8 μg/mL. HTTL-F cells were transfected with GPCRs demonstrating high constitutive internalization: CHRM5 (a), GPR87 (b), GPR126 (c), VIPR2 (d), and CD97 (e). Transfected cells were stimulated as a cumate dose-response, and stimulation curves were built using XY analysis for non-linear regression curve and the 4-parameters dose-response stimulation function, followed by baseline correction. Data are presented as mean values, with error bars representing SEM ($n = 3$, with three technical replicates from one biological sample), and generic receptor codes refer to the GPCR-Tango constructs. qPCR was performed on untreated and infected HTTL-F cells to confirm sufficient knockdown of β-arrestin-1 and -2 (f). Data are presented as mean values, with error bars representing SEM. Fold change of gene expression and corresponding p values (two-tailed) were assessed with multiple unpaired t test using the FDR method of Benjamini & Yekutieli ($n = 2$, with two technical replicates from one biological sample).

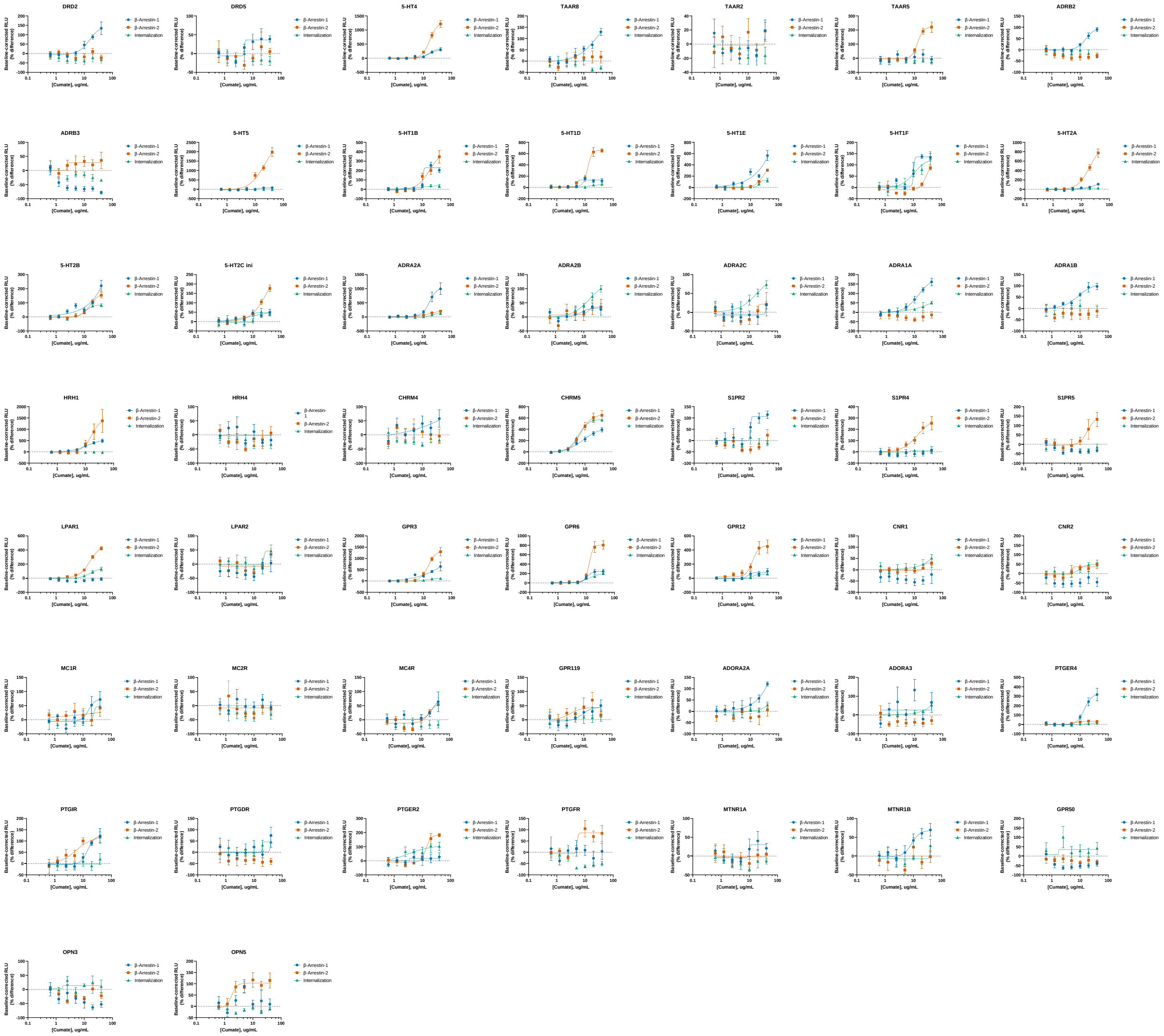

Supplementary Figure 11. Basal profiles of β-arrestin-1 and β-arrestin-2 translocation, and receptor internalization generated using Tango-Trio (Class A, α-branch). To profile basal activities, HTTL-B1, HTTL-B2 and HTTL-F cells were transfected with GPCR Tango constructs. Transfected cells were stimulated with cumate in a dose-dependent manner. Dose-response curves were built using XY analysis for non-linear regression curve and the 4-parameters dose-response stimulation function, followed by baseline correction. Data are presented as mean values, with error bars representing SEM (n = 3, with 3 technical replicates from one biological sample). Generic receptor codes refer to the GPCR-Tango constructs.

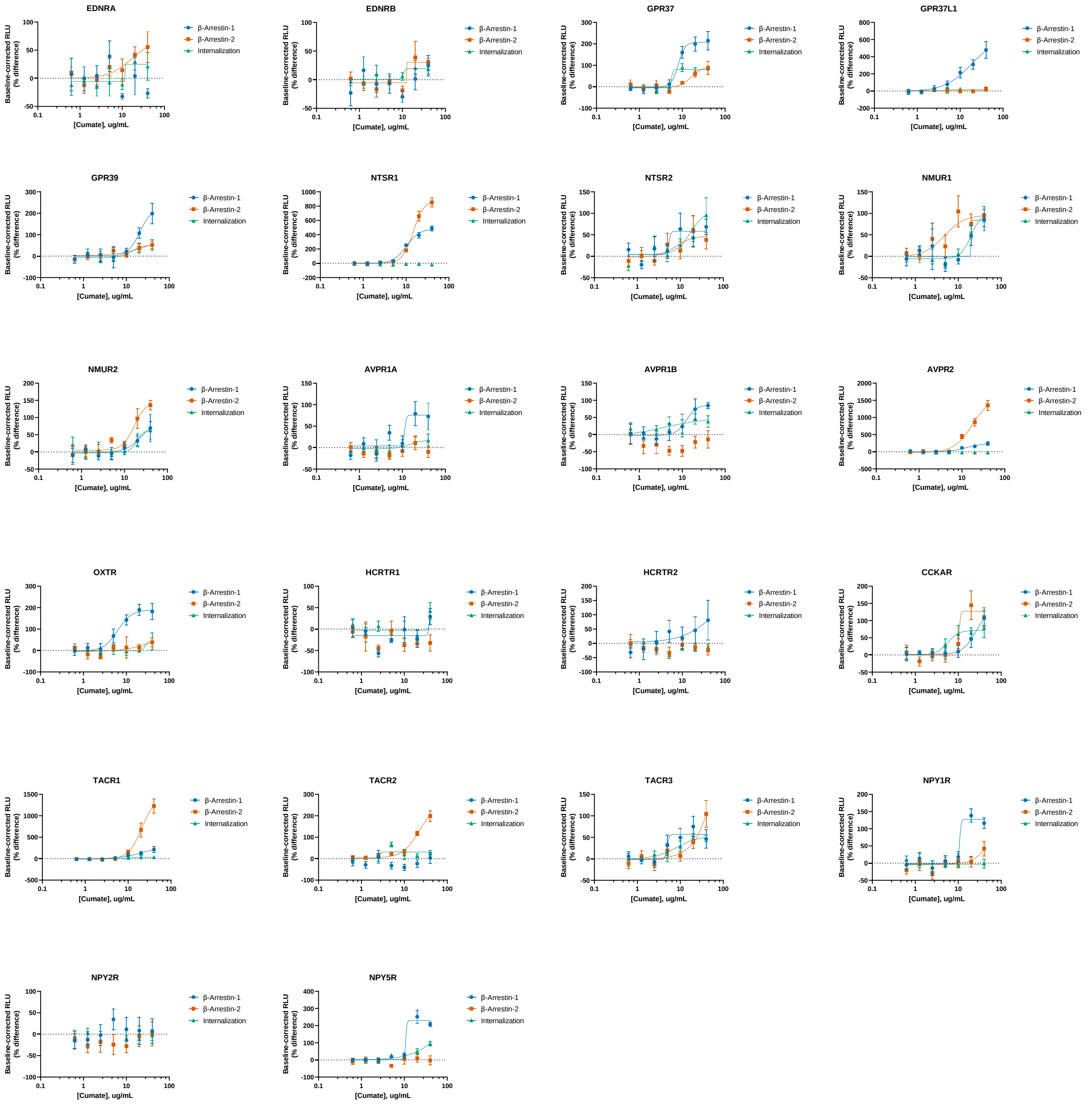

Supplementary Figure 12. Basal profiles of β-arrestin-1 and β-arrestin-2 translocation, and receptor internalization generated using Tango-Trio (Class A, β-branch). To profile basal activities, HTTL-B1, HTTL-B2 and HTTL-F cells were transfected with GPCR Tango constructs. Transfected cells were stimulated with cumate in a dose-dependent manner. Dose-response curves were built using XY analysis for non-linear regression curve and the 4-parameters dose-response stimulation function, followed by baseline correction. Data are presented as mean values, with error bars representing SEM (n = 3, with 3 technical replicates from one biological sample). Generic receptor codes refer to the GPCR-Tango constructs.

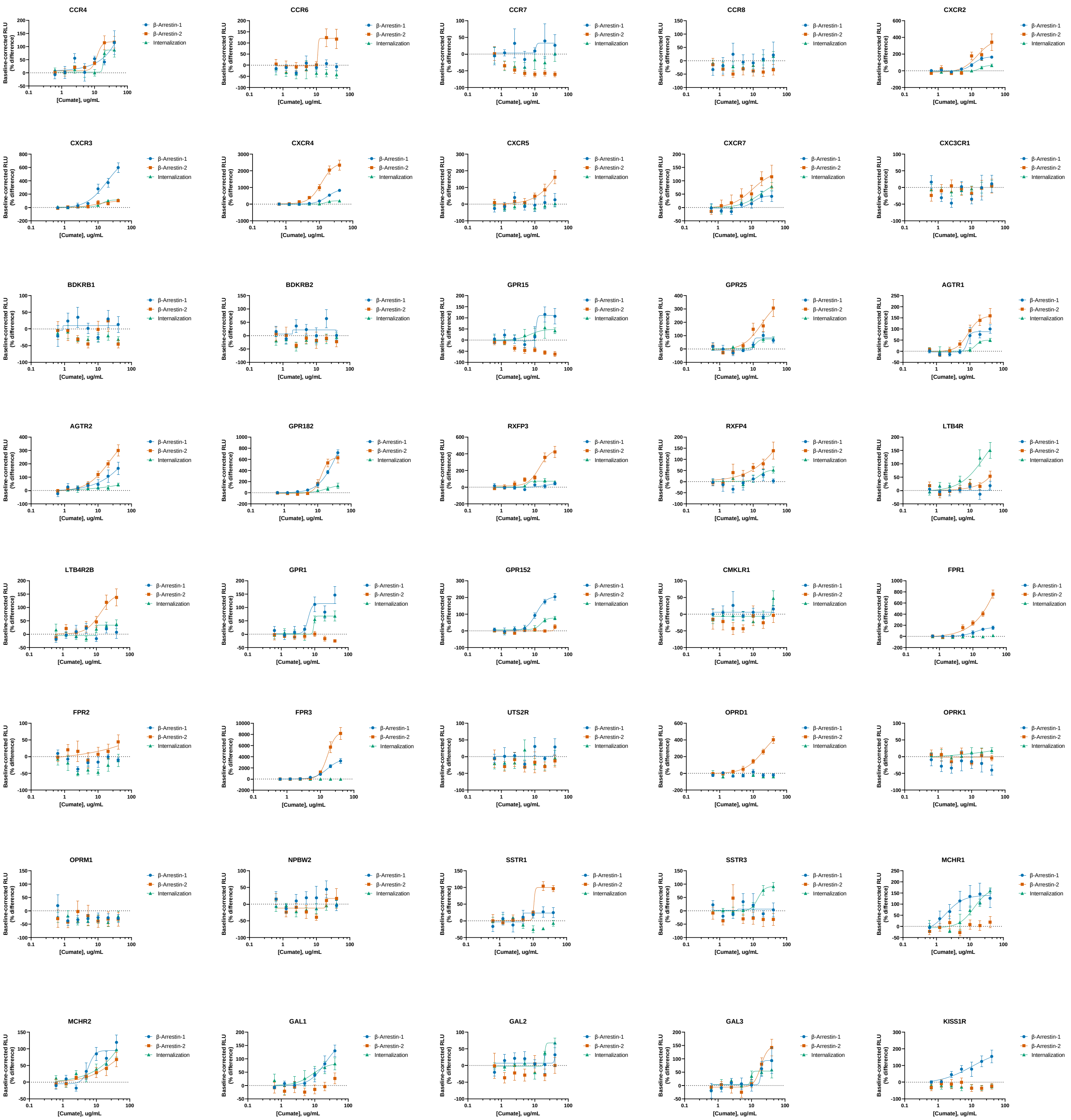

Supplementary Figure 13. Basal profiles of β-arrestin-1 and β-arrestin-2 translocation, and receptor internalization generated using Tango-Trio (Class A, γ-branch). To profile basal activities, HTTL-B1, HTTL-B2 and HTTL-F cells were transfected with GPCR Tango constructs. Transfected cells were stimulated with cumate in a dose-dependent manner. Dose-response curves were built using XY analysis for non-linear regression curve and the 4-parameters dose-response stimulation function, followed by baseline correction. Data are presented as mean values, with error bars representing SEM (n = 3, with 3 technical replicates from one biological sample). Generic receptor codes refer to the GPCR-Tango constructs.

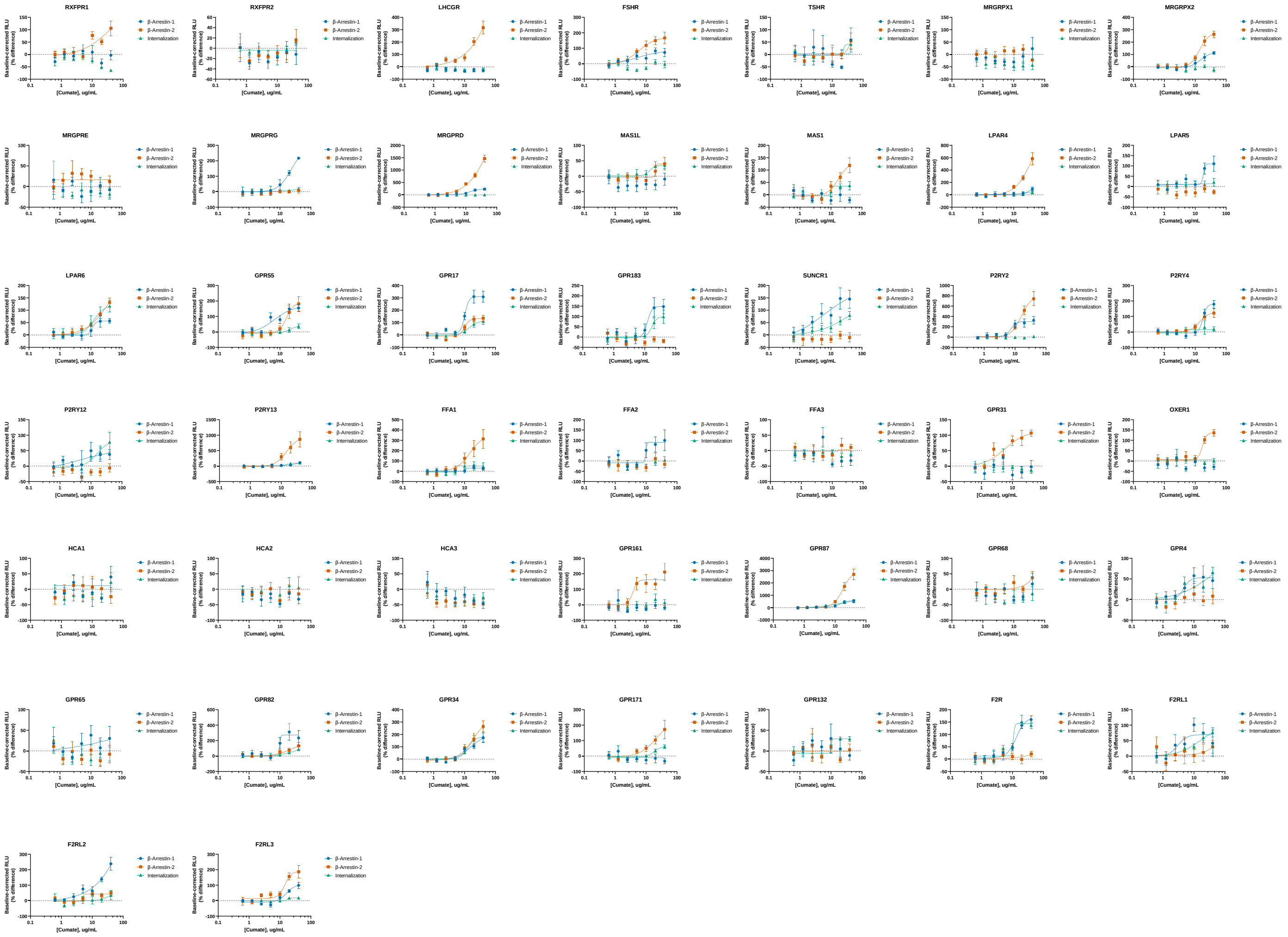

Supplementary Figure 14. Basal profiles of β-arrestin-1 and β-arrestin-2 translocation, and receptor internalization generated using Tango-Trio (Class A, δ-branch). To profile basal activities, HTTL-B1, HTTL-B2 and HTTL-F cells were transfected with GPCR Tango constructs. Transfected cells were stimulated with cumate in a dose-dependent manner. Dose-response curves were built using XY analysis for non-linear regression curve and the 4-parameters dose-response stimulation function, followed by baseline correction. Data are presented as mean values, with error bars representing SEM (n = 3, with 3 technical replicates from one biological sample). Generic receptor codes refer to the GPCR-Tango constructs.

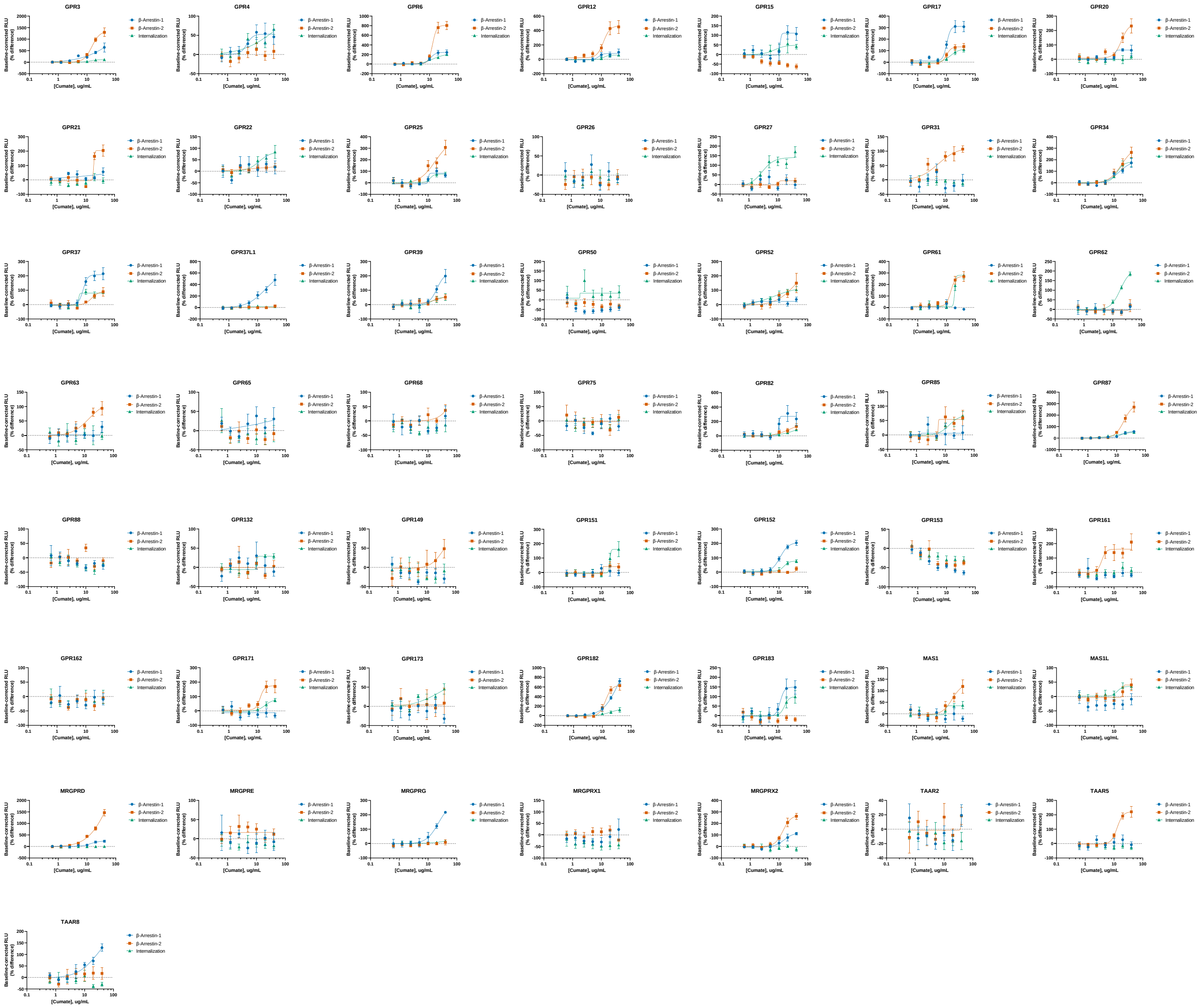

Supplementary Figure 15. Basal profiles of β-arrestin-1 and β-arrestin-2 translocation, and receptor internalization generated using Tango-Trio (Class A, orphan receptors). To profile basal activities, HTTL-B1, HTTL-B2 and HTTL-F cells were transfected with GPCR Tango constructs. Transfected cells were stimulated with cumate in a dose-dependent manner. Dose-response curves were built using XY analysis for non-linear regression curve and the 4-parameters dose-response stimulation function, followed by baseline correction. Data are presented as mean values, with error bars representing SEM (n = 3, with 3 technical replicates from one biological sample). Generic receptor codes refer to the GPCR-Tango constructs.

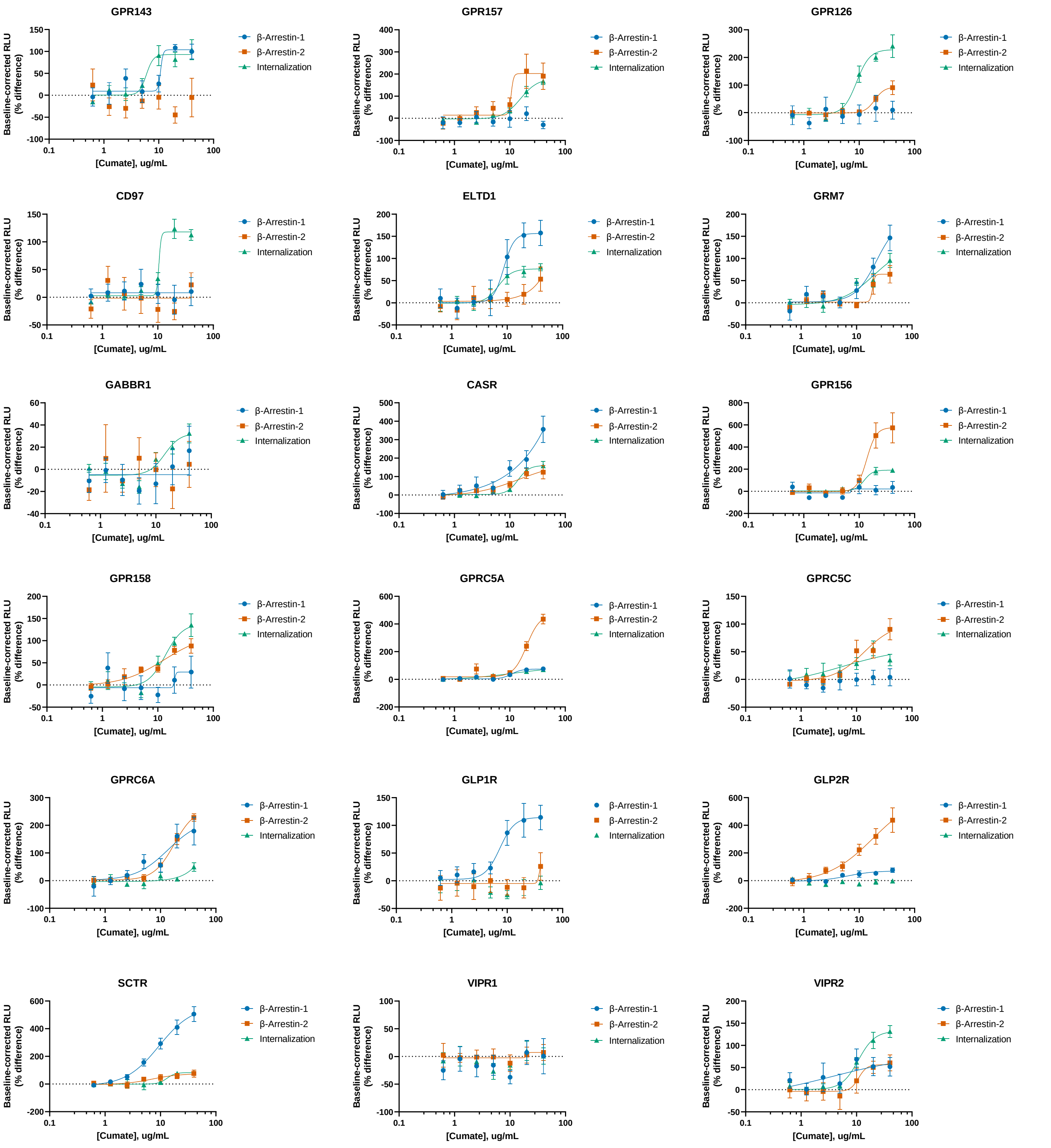

Supplementary Figure 16. Basal profiles of β-arrestin-1 and β-arrestin-2 translocation, and receptor internalization generated using Tango-Trio (Class B/C). To profile basal activities, HTTL-B1, HTTL-B2 and HTTL-F cells were transfected with GPCR Tango constructs. Transfected cells were stimulated with cumate in a dose-dependent manner. Dose-response curves were built using XY analysis for non-linear regression curve and the 4-parameters dose-response stimulation function, followed by baseline correction. Data are presented as mean values, with error bars representing SEM (n = 3, with 3 technical replicates from one biological sample). Generic receptor codes refer to the GPCR-Tango constructs.

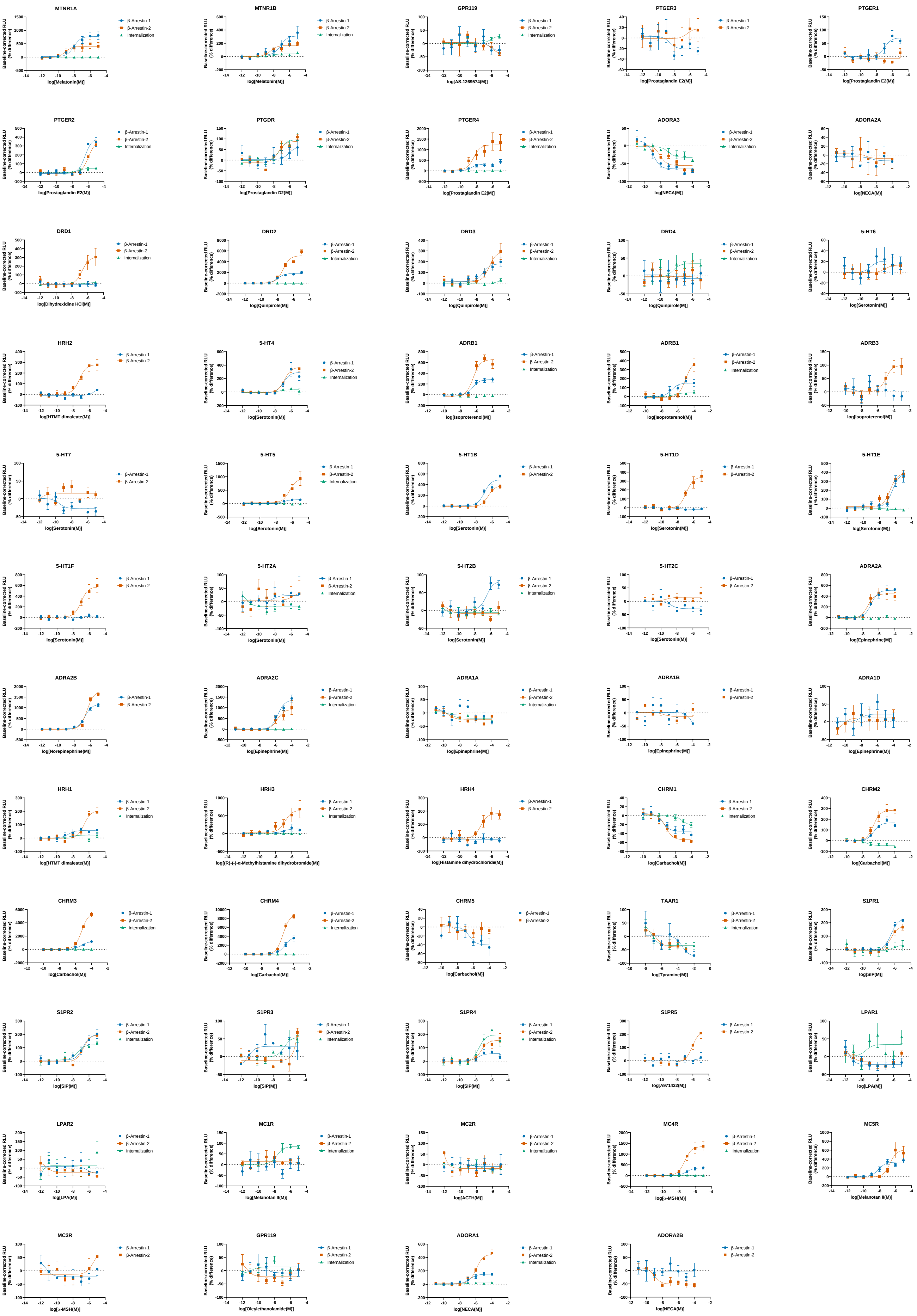
Baseline-corrected RLU (% difference)
β-Arrestin-1
β-Arrestin-2
Internalization
MTNR1A log[Melatonin(M)]
MTNR1B log[Melatonin(M)]
GPR119 log[AS-1269574(M)]
PTGER3 log[Prostaglandin E2(M)]
PTGER1 log[Prostaglandin E2(M)]
PTGER2 log[Prostaglandin E2(M)]
PTGDR log[Prostaglandin D2(M)]
PTGER4 log[Prostaglandin E2(M)]
ADORA3 log[NECA(M)]
ADORA2A log[NECA(M)]
DRD1 log[Dihydrexidine HCl(M)]
DRD2 log[Quinpirole(M)]
DRD3 log[Quinpirole(M)]
DRD4 log[Quinpirole(M)]
5-HT6 log[Serotonin(M)]
HRH2 log[HTMT dimaleate(M)]
5-HT4 log[Serotonin(M)]
ADRB1 log[Isoproterenol(M)]
ADRB1 log[Isoproterenol(M)]
ADRB3 log[Isoproterenol(M)]
5-HT7 log[Serotonin(M)]
5-HT5 log[Serotonin(M)]
5-HT1B log[Serotonin(M)]
5-HT1D log[Serotonin(M)]
5-HT1E log[Serotonin(M)]
5-HT1F log[Serotonin(M)]
5-HT2A log[Serotonin(M)]
5-HT2B log[Serotonin(M)]
5-HT2C log[Serotonin(M)]
ADRA2A log[Epinephrine(M)]
ADRA2B log[Norepinephrine(M)]
ADRA2C log[Epinephrine(M)]
ADRA1A log[Epinephrine(M)]
ADRA1B log[Epinephrine(M)]
ADRA1D log[Epinephrine(M)]
HRH1 log[HTMT dimaleate(M)]
HRH3 log[(R)-(-)-α-Methylhistamine dihydrobromide(M)]
HRH4 log[Histamine dihydrochloride(M)]
CHRM1 log[Carbachol(M)]
CHRM2 log[Carbachol(M)]
CHRM3 log[Carbachol(M)]
CHRM4 log[Carbachol(M)]
CHRM5 log[Carbachol(M)]
TAAR1 log[Tyramine(M)]
S1PR1 log[SIP(M)]
S1PR2 log[SIP(M)]
S1PR3 log[SIP(M)]
S1PR4 log[SIP(M)]
S1PR5 log[A971432(M)]
LPAR1 log[LPA(M)]
LPAR2 log[LPA(M)]
MC1R log[Melanotan II(M)]
MC2R log[ACTH(M)]
MC4R log[α-MSH(M)]
MC5R log[Melanotan II(M)]
MC3R log[α-MSH(M)]
GPR119 log[Oleylethanolamide(M)]
ADORA1 log[NECA(M)]
ADORA2B log[NECA(M)]

Supplementary Figure 17. Agonist-induced profiles of β-arrestin-1 and β-arrestin-2 translocation, and receptor internalization generated using Tango-Trio (Class A, α-branch). To quantify agonist-dependent activities, HTTL-B1, HTTL-B2 and HTTL-F cells were plated in cumate-containing (30 µg/mL) medium and transfected with non-orphan GPCR Tango constructs. Transfected cells were stimulated with the receptor-specific agonist, and dose-response curves were built using XY analysis for non-linear regression curve and the 3-parameters dose-response stimulation function, followed by baseline correction. Data are presented as mean values, with error bars representing SEM (n = 3, with 3 technical replicates from one biological sample). Generic receptor codes refer to the GPCR-Tango constructs.

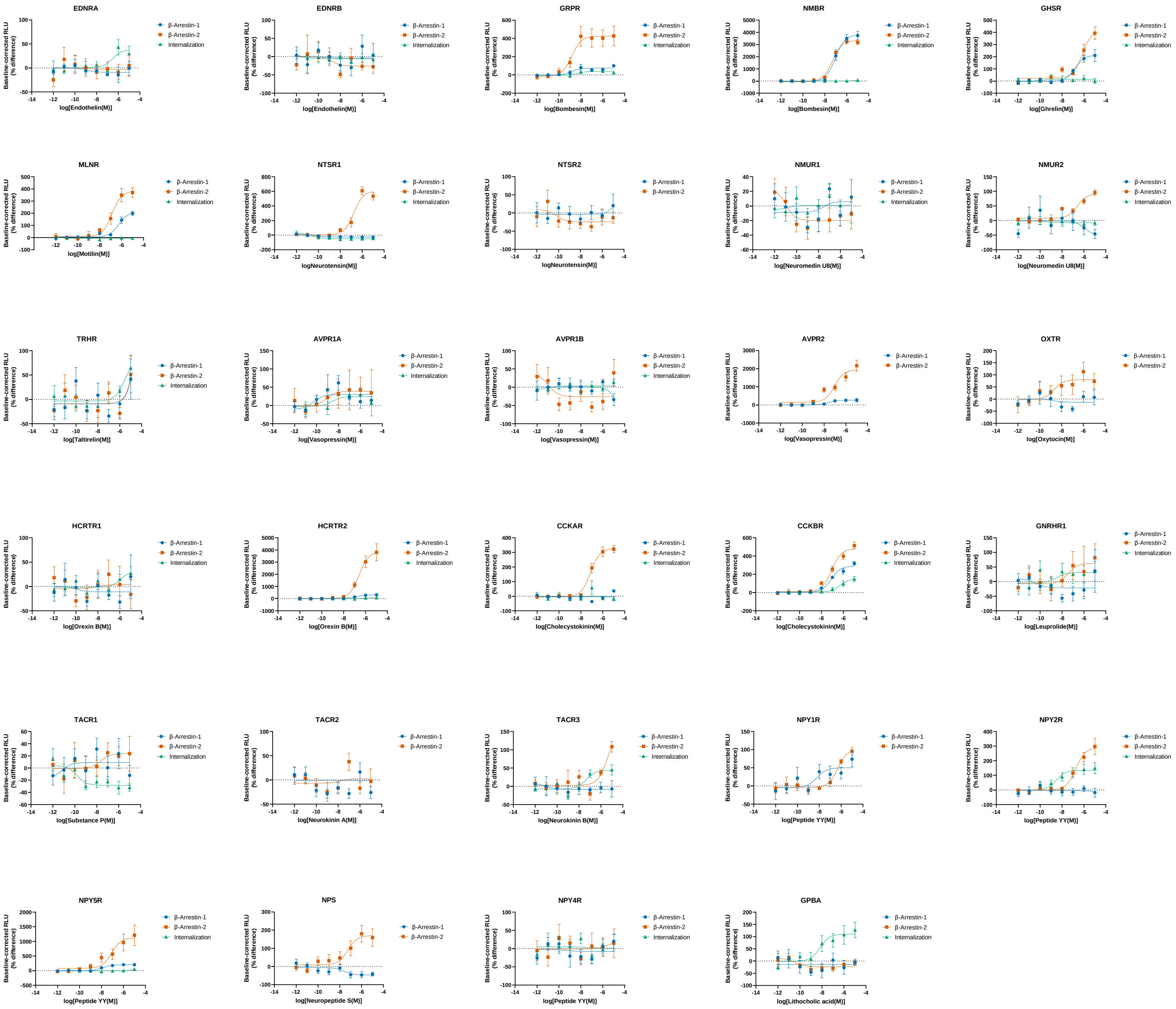

Supplementary Figure 18. Agonist-induced profiles of β-arrestin-1 and β-arrestin-2 translocation, and receptor internalization generated using Tango-Trio (Class A, β-branch). To quantify agonist-dependent activities, HTTL-B1, HTTL-B2 and HTTL-F cells were plated in cumate-containing (30 µg/mL) medium and transfected with non-orphan GPCR Tango constructs. Transfected cells were stimulated with the receptor-specific agonist, and dose-response curves were built using XY analysis for non-linear regression curve and the 3-parameters dose-response stimulation function, followed by baseline correction. Data are presented as mean values, with error bars representing SEM (n = 3, with 3 technical replicates from one biological sample). Generic receptor codes refer to the GPCR-Tango constructs.

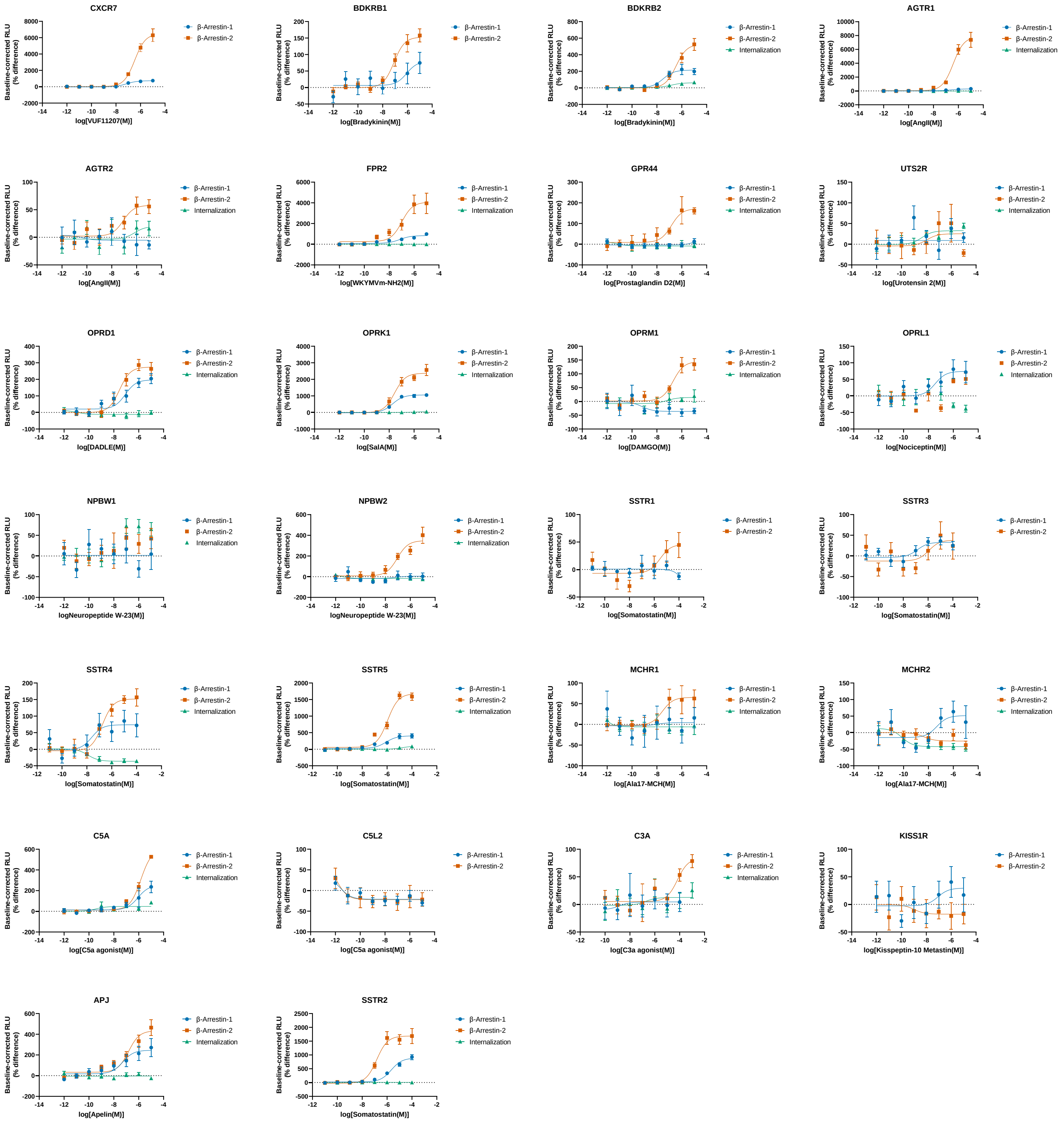

Supplementary Figure 19. Agonist-induced profiles of β-arrestin-1 and β-arrestin-2 translocation, and receptor internalization generated using Tango-Trio (Class A, γ-branch). To quantify agonist-dependent activities, HTTL-B1, HTTL-B2 and HTTL-F cells were plated in cumate-containing (30 µg/mL) medium and transfected with non-orphan GPCR Tango constructs. Transfected cells were stimulated with the receptor-specific agonist, and dose-response curves were built using XY analysis for non-linear regression curve and the 3-parameters dose-response stimulation function, followed by baseline correction. Data are presented as mean values, with error bars representing SEM (n = 3, with 3 technical replicates from one biological sample). Generic receptor codes refer to the GPCR-Tango constructs.

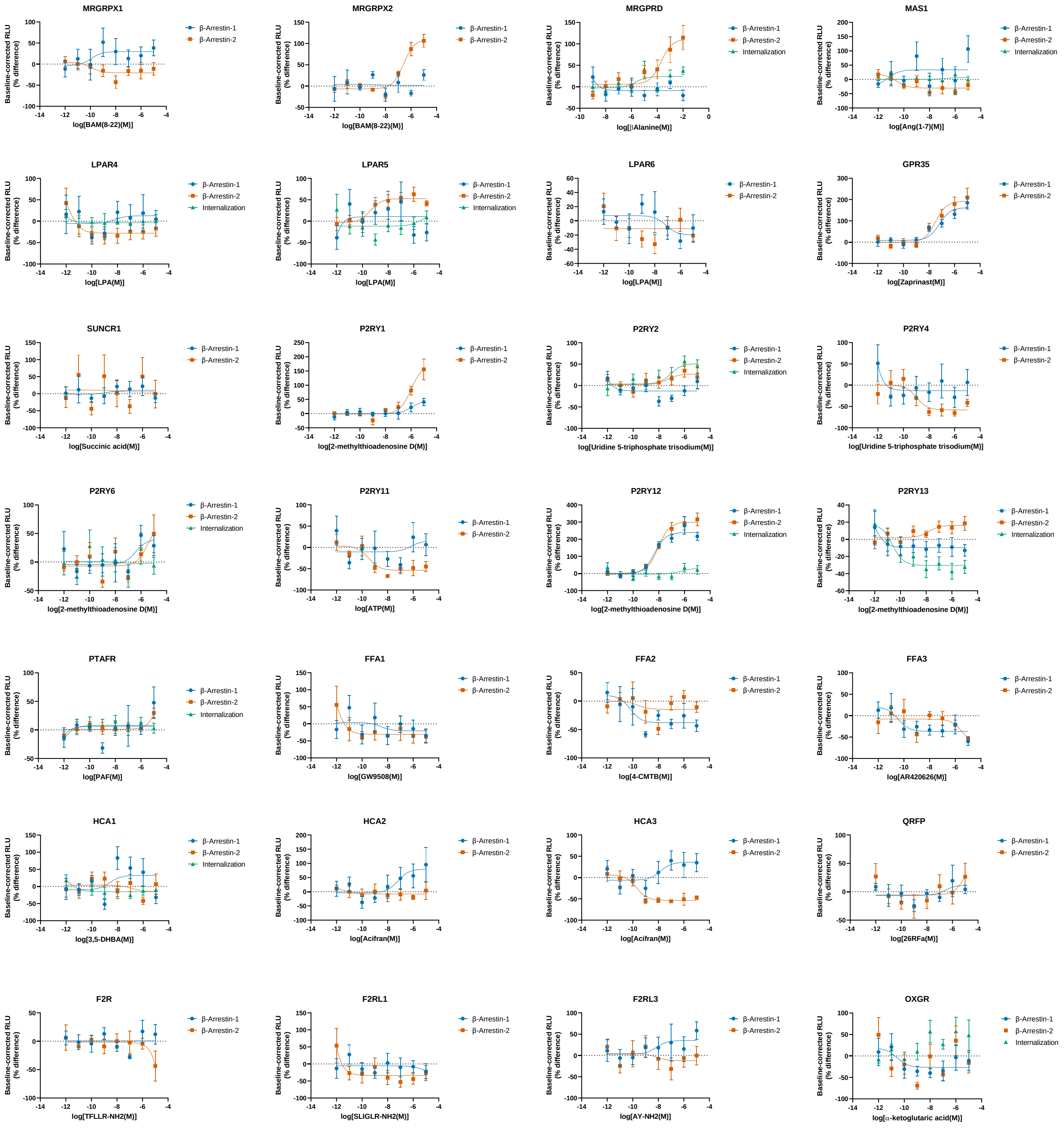

Supplementary Figure 20. Agonist-induced profiles of β-arrestin-1 and β-arrestin-2 translocation, and receptor internalization generated using Tango-Trio (Class A, δ-branch). To quantify agonist-dependent activities, HTTL-B1, HTTL-B2 and HTTL-F cells were plated in cumate-containing (30 µg/mL) medium and transfected with non-orphan GPCR Tango constructs. Transfected cells were stimulated with the receptor-specific agonist, and dose-response curves were built using XY analysis for non-linear regression curve and the 3-parameters dose-response stimulation function, followed by baseline correction. Data are presented as mean values, with error bars representing SEM (n = 3, with 3 technical replicates from one biological sample). Generic receptor codes refer to the GPCR-Tango constructs.

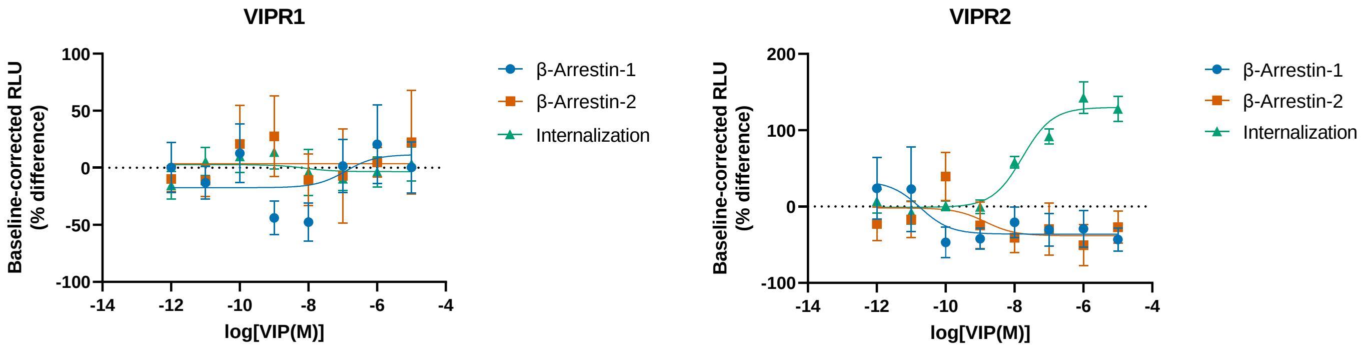

Supplementary Figure 21. Agonist-induced profiles of β-arrestin-1 and β-arrestin-2 translocation, and receptor internalization generated using Tango-Trio (Class B/C). To quantify agonist-dependent activities, HTTL-B1, HTTL-B2 and HTTL-F cells were plated in cumate-containing (30 µg/mL) medium and transfected with non-orphan GPCR Tango constructs. Transfected cells were stimulated with the receptor-specific agonist, and dose-response curves were built using XY analysis for non-linear regression curve and the 3-parameters dose-response stimulation function, followed by baseline correction. Data are presented as mean values, with error bars representing SEM (n = 3, with 3 technical replicates from one biological sample). Generic receptor codes refer to the GPCR-Tango constructs.

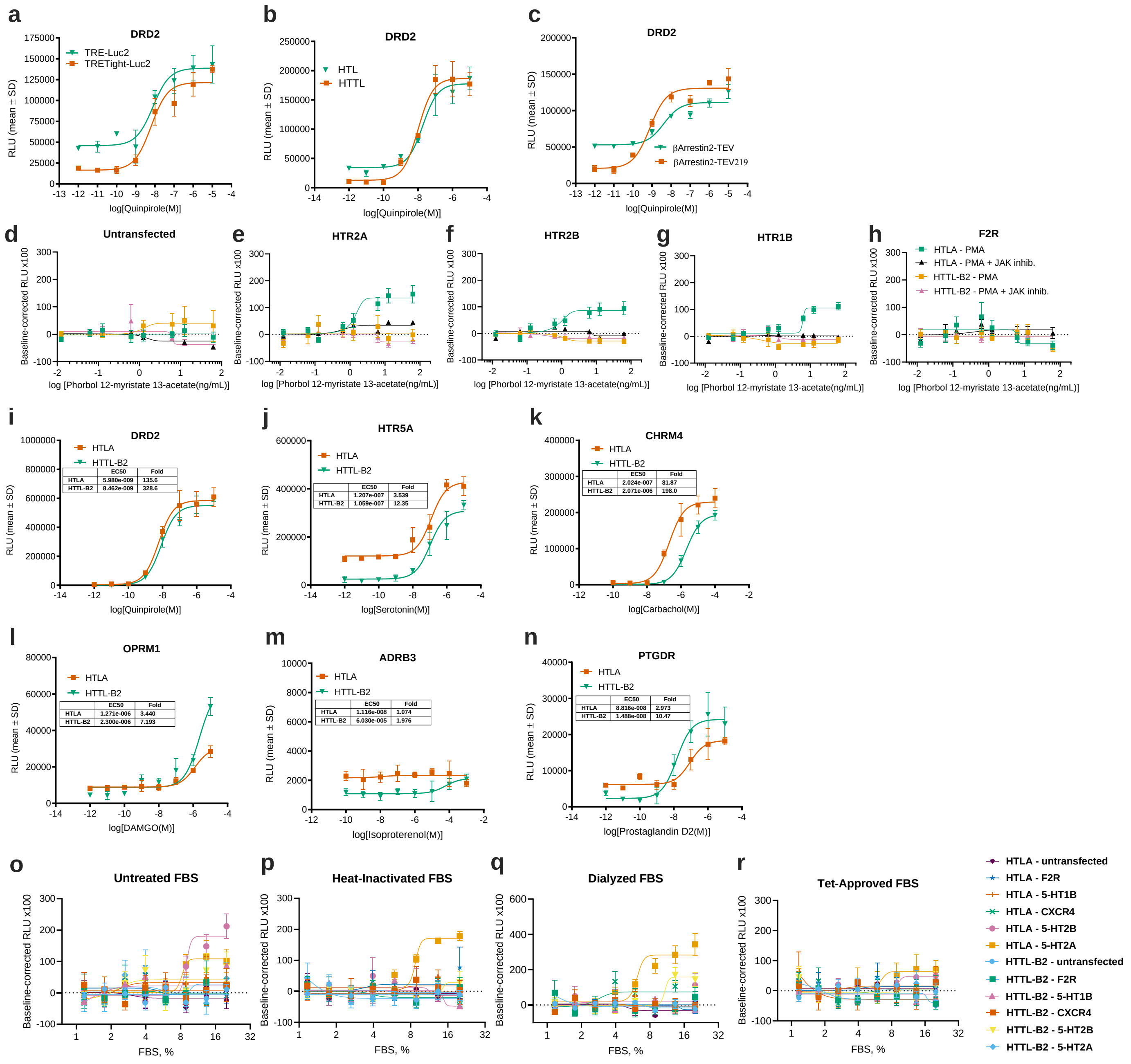

Supplementary Figure 22. Optimization of the dynamic range, sensitivity, and specificity of the Tango-Trio platform - independent biological replicate of the main manuscript Figure 1. (A-R) Data are representative of 2 biological replicates, with 3 technical replicates each.

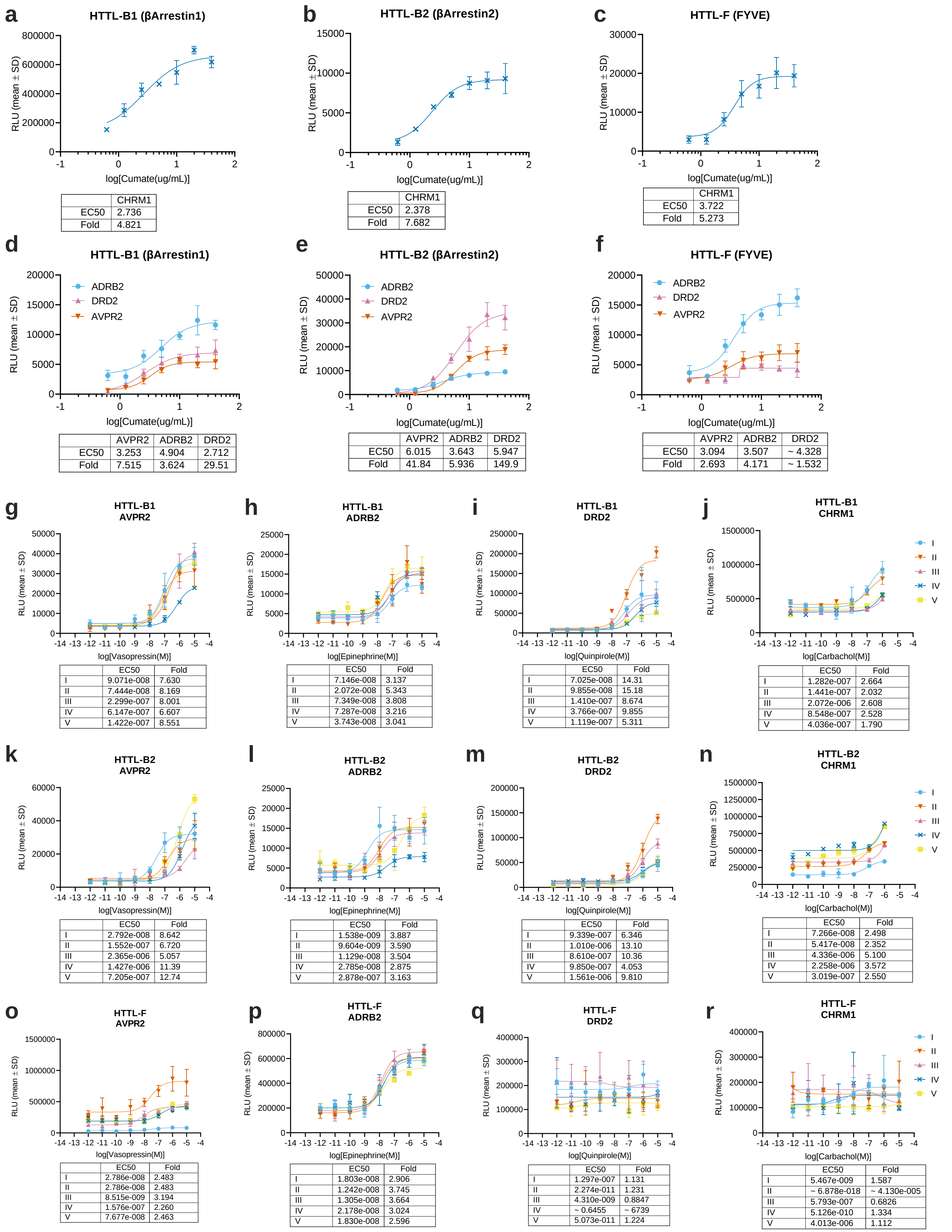

a

	CHRM1
EC50	2.736
Fold	4.821

b

	CHRM1
EC50	2.378
Fold	7.682

c

	CHRM1
EC50	3.722
Fold	5.273

d

	AVPR2	ADRB2	DRD2
EC50	3.253	4.904	2.712
Fold	7.515	3.624	29.51

e

	AVPR2	ADRB2	DRD2
EC50	6.015	3.643	5.947
Fold	41.84	5.936	149.9

f

	AVPR2	ADRB2	DRD2
EC50	3.094	3.507	~ 4.328
Fold	2.693	4.171	~ 1.532

g

	EC50	Fold
I	9.071e-008	7.630
II	7.444e-008	8.169
III	2.299e-007	8.001
IV	6.147e-007	6.607
V	1.422e-007	8.551

h

	EC50	Fold
I	7.146e-008	3.137
II	2.072e-008	5.343
III	7.349e-008	3.808
IV	7.287e-008	3.216
V	3.743e-008	3.041

i

	EC50	Fold
I	7.025e-008	14.31
II	9.855e-008	15.18
III	1.410e-007	8.674
IV	3.766e-007	9.855
V	1.119e-007	5.311

j

	EC50	Fold
I	1.282e-007	2.664
II	1.441e-007	2.032
III	2.072e-006	2.608
IV	8.548e-007	2.528
V	4.036e-007	1.790

k

	EC50	Fold
I	2.792e-008	8.642
II	1.552e-007	6.720
III	2.365e-006	5.057
IV	1.427e-006	11.39
V	7.205e-007	12.74

l

	EC50	Fold
I	1.538e-009	3.887
II	9.604e-009	3.590
III	1.129e-008	3.504
IV	2.785e-008	2.875
V	2.878e-007	3.163

m

	EC50	Fold
I	9.339e-007	6.346
II	1.010e-006	13.10
III	8.610e-007	10.36
IV	9.850e-007	4.053
V	1.561e-006	9.810

n

	EC50	Fold
I	7.266e-008	2.498
II	5.417e-008	2.352
III	4.336e-006	5.100
IV	2.258e-006	3.572
V	3.019e-007	2.550

o

	EC50	Fold
I	2.786e-008	2.483
II	2.786e-008	2.483
III	8.515e-009	3.194
IV	1.576e-007	2.260
V	7.677e-008	2.463

p

	EC50	Fold
I	1.803e-008	2.906
II	1.242e-008	3.745
III	1.305e-008	3.664
IV	2.178e-008	3.024
V	1.830e-008	2.596

q

	EC50	Fold
I	1.297e-007	1.131
II	2.274e-011	1.231
III	4.310e-009	0.8847
IV	~ 0.6455	~ 6739
V	5.073e-011	1.224

r

	EC50	Fold
I	5.467e-009	1.587
II	~ 6.878e-018	~ 4.130e-005
III	5.793e-007	0.6826
IV	5.126e-010	1.334
V	4.013e-006	1.112

Supplementary Figure 23. Dose-response and time-course verification of cumate-induced expression - independent biological replicate of the main manuscript Figure 2. (A-R) Data are representative of 2 biologi cal replicates, with 3 technical replicates each.

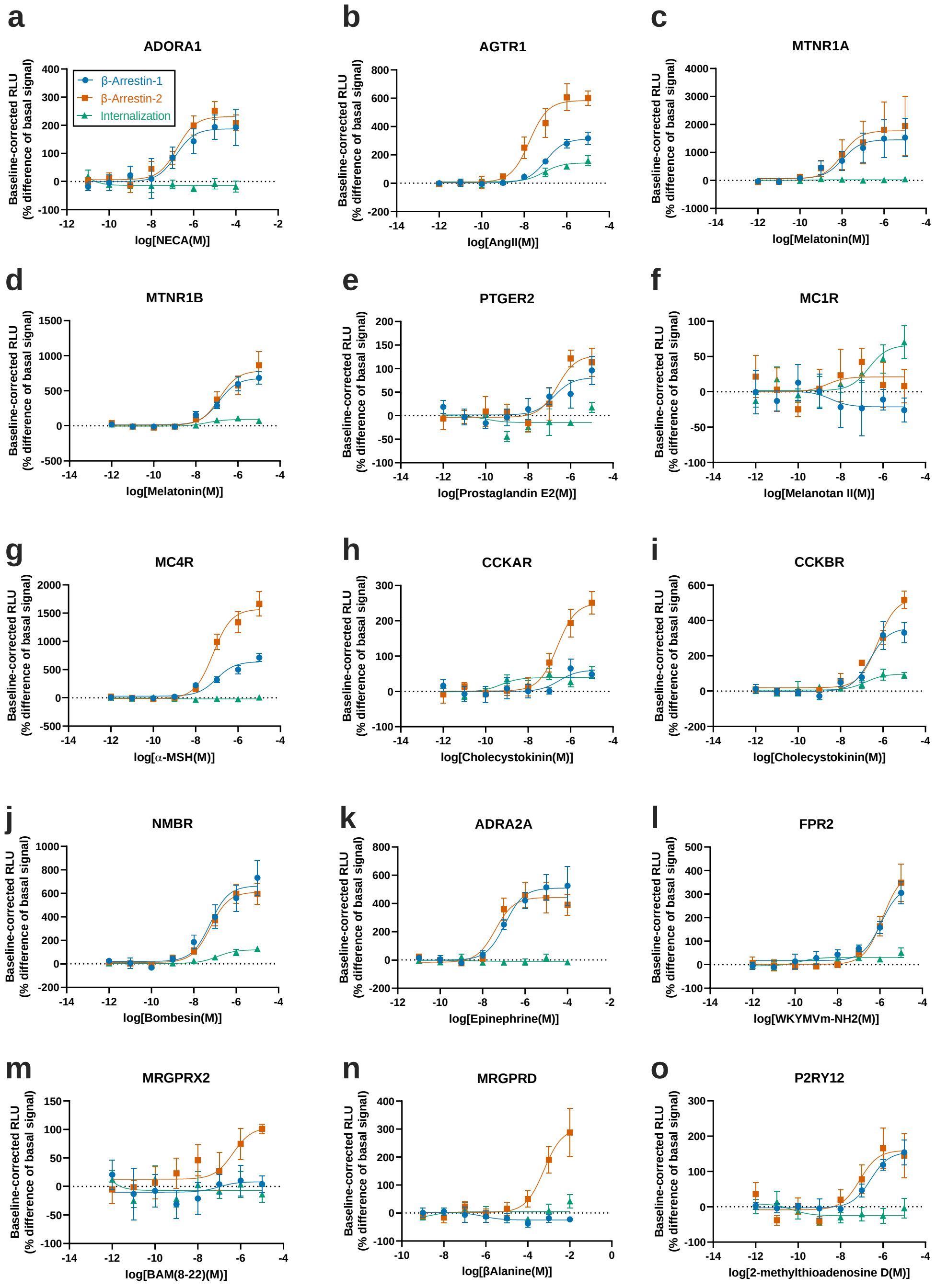

Supplementary Figure 24. Validation of compiled positive hits from agonist-dependent HTS in dose response - independent biological replicate of the main manuscript Figure 5. (A-O) Data are representative of 2 biological replicates, with 3 technical replicates each.

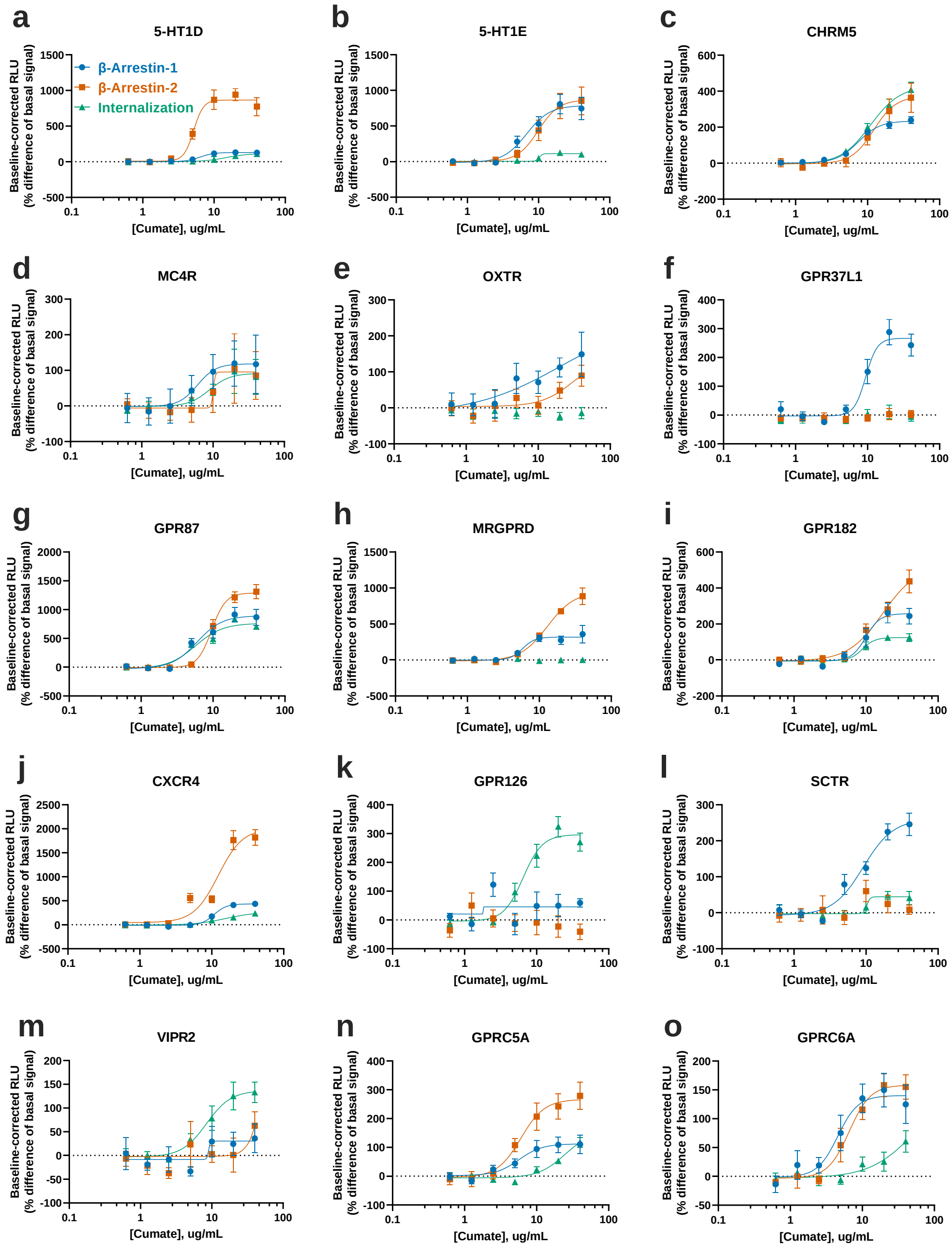

Supplementary Figure 25. Validation of compiled positive hits from basal activity HTS in dose-response - independent biological replicate of the main manuscript Figure 6. (A-O) Data are representative of 2 biologi cal replicates, with 3 technical replicates each.

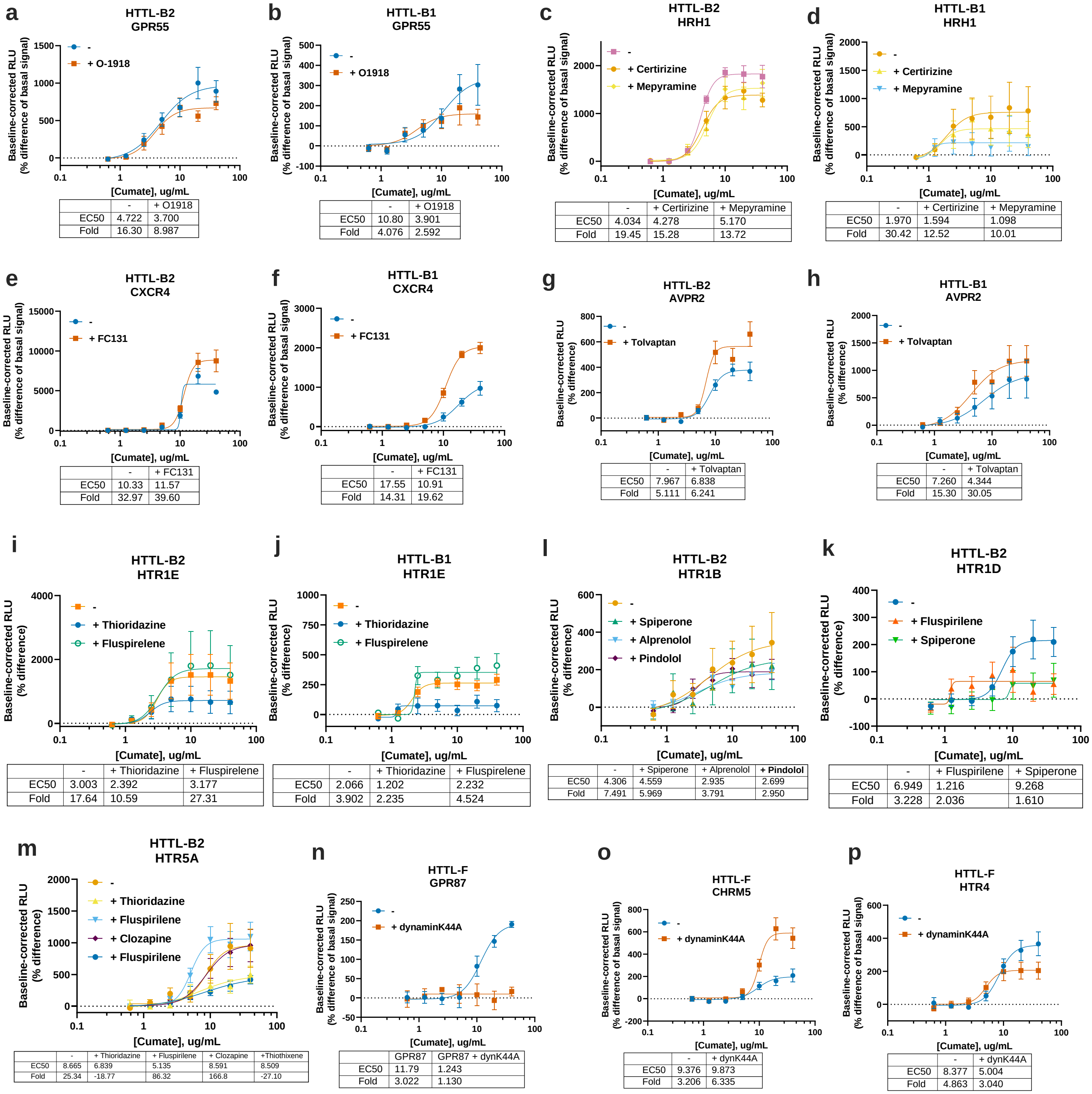

a

	-	+ O1918
EC50	4.722	3.700
Fold	16.30	8.987

b

	-	+ O1918
EC50	10.80	3.901
Fold	4.076	2.592

c

	-	+ Certirizine	+ Mepyramine
EC50	4.034	4.278	5.170
Fold	19.45	15.28	13.72

d

	-	+ Certirizine	+ Mepyramine
EC50	1.970	1.594	1.098
Fold	30.42	12.52	10.01

e

	-	+ FC131
EC50	10.33	11.57
Fold	32.97	39.60

f

	-	+ FC131
EC50	17.55	10.91
Fold	14.31	19.62

g

	-	+ Tolvaptan
EC50	7.967	6.838
Fold	5.111	6.241

h

	-	+ Tolvaptan
EC50	7.260	4.344
Fold	15.30	30.05

i

	-	+ Thioridazine	+ Fluspirelene
EC50	3.003	2.392	3.177
Fold	17.64	10.59	27.31

j

	-	+ Thioridazine	+ Fluspirelene
EC50	2.066	1.202	2.232
Fold	3.902	2.235	4.524

l

	-	+ Spiperone	+ Alprenolol	**+ Pindolol**
EC50	4.306	4.559	2.935	2.699
Fold	7.491	5.969	3.791	2.950

k

	-	+ Fluspirilene	+ Spiperone
EC50	6.949	1.216	9.268
Fold	3.228	2.036	1.610

m

	-	+ Thioridazine	+ Fluspirilene	+ Clozapine	+Thiothixene
EC50	8.665	6.839	5.135	8.591	8.509
Fold	25.34	-18.77	86.32	166.8	-27.10

n

	GPR87	GPR87 + dynK44A
EC50	11.79	1.243
Fold	3.022	1.130

o

	-	+ dynK44A
EC50	9.376	9.873
Fold	3.206	6.335

p

	-	+ dynK44A
EC50	8.377	5.004
Fold	4.863	3.040

Supplementary Figure 26. Applications and further investigations into basal activities revealed by Tango-Trio - independent biological replicate of the main manuscript Figure 7. (A-P) Data are representativ e of 2 biological replicates, with 3 technical replicates each.

Supplemental Tables

Supplementary Table 1. Comparison of the pharmacological parameters extracted from EMTA and Tango-Trio. Absolute pEC50 and Emax values of β-arrestin-1/2 activity at GPCRs stimulated with common ligands were extracted from EMTA (Avet et al. 2022) and Tango-Trio studies. Tango-Trio pEC50 data was extracted from the non-linear least-squares regression analysis using the sigmoidal dose-response function (3-parameters modeled using Y=Bottom + (Top-Bottom)/(1+10^((LogEC50-X))) and Emax values from the baseline correction as percentage difference using 100* (Value-Baseline)/Baseline), both provided in GraphPad Prism 9.5.1. (n = 3, with three technical replicates from one biological sample). Generic receptor codes refer to the GPCR-Tango constructs.

		pEC50				Emax (in % of vehicle response for EMTA & % difference from baseline for Tango-Trio)			
**Receptor (Gene Name)**	**Ligand**	**βArrestin1 /GRK2 EMTA**	**βArrestin1 Tango-Trio**	**βArrestin2 /GRK2 EMTA**	**β-Arrestin2 Tango-Trio**	**βArrestin1 /GRK2 EMTA**	**βArrestin1 Tango-Trio**	**βArrestin2 /GRK2 EMTA**	**β-Arrestin2 Tango-Trio**
HTR1B	Serotonin	7.6	6.96	7.685	6.49	36.35	490	57.78	393.4
HTR1D	Serotonin		7.66		6.89		-14.78		347.9
HTR2A	Serotonin	6.709	8.22	7.028	5.76	518.1	24.81	778.6	29.24
HTR2B	Serotonin	7.809	6.5	8.168	11.31	171.6	80.54	247.2	-26.67
HTR2C	Serotonin	7.995	8.76	8.456	9.33	1564	-28.38	705	14.12
AGTR1	Angiotensin II	8.64	6.62	8.84	6.41	207.8	289.2	220.6	7802
CCKAR	Cholecystokinin	8.329	unstable	8.721	7.11	1374	unstable	1520	332.9
PTGER1	PGE2		6.95		unstable		71.02		unstable
PTGER2	PGE2	6.515	6.38	6.925	5.95	107.8	400.8	100.5	363
PTGER3	PGE2		8.53		6.324		-24.13		19.44
PTGER4	PGE2	8.921	8.62	9.25	8.35	251.5	326.9	146.8	1192
EDNRA	Endothelin-1	8.232	8.9	8.381	8.7	588.1	-8.86	1581	-2.98
GHSR	Ghrelin	8.249	6.81	8.515	6.14	68.41	217.8	169.6	396.8
GNRHR	GnRH		10.32		7.43		-31.52		70.49
GPBAR1	Litocholic acid		unstable		10.41		unstable		-34.66
LPAR1	O-LPA	7.528	11.93	7.464	11.21	289.4	-90.04	232	-32.09
LPAR2	O-LPA	6.773	6.13	7.078	11.33	715.2	-41.64	938.9	-64.29
MC4R	α-MSH		7.01	7.449	7.28		346.3	76.44	1351
OPRM1	DAMGO	7.099	9.19	7.424	6.7	1008	-35.21	1198	142.9
MTNR1A	Melatonin	8.937	8.03	8.75	9.11	78.64	754.1	157.5	425.5
MTNR1B	Melatonin		7.12		8.38		290		168
OPRL1	Nociceptin	8.249	6.15	8.716	6.17	147.5	73.78	238.1	-50.64
OXTR	Oxytocin	8.091	8.78	8.6	8.67	128.3	-13.08	229.8	82.31
HCRTR2	Orexin-A	8.471	6.9	8.558	6.58	852.9	315.7	863.9	3850
P2RY2	UTP	5.235	unstable	5.36	7.29	184.7	unstable	94.68	24.6
F2R	TFLLR-NH2	5.082	unstable	5.177	-2.55	445.5	unstable	469.5	unstable
S1PR1	Sphingosine 1-phosphate	7.269	6.47	7.459	6.5	477	233.6	557.1	182.8
SSTR2	Somatostatin	8.815	5.66	8.918	6.83	2867	866	1820	1714
AVPR1A	AVP	7.743	10.25	7.824	9.08	890.1	40.7	934.7	40.67
AVP2R	AVP	7.603	7.52	7.846	7.03	353.3	258.6	334.2	1804
VIPR1	VIP	9.109	6.92	9.254	unstable	1367	28.97	1060	unstable
NPY1R	NPY		8.2	8.815	6.28		53.76	42.09	106.1
NPY5R	NPY		7.92		7.07		210.3		1046

Supplemental Notes

Supplementary Note 1. Sequences of Tango-Trio constructs stably expressed in HTTL, HTTL-B1, HTTL-B2, and HTTL-F cell lines. Information regarding the cloning of the plasmids is provided in the Methods.

Tight-pNLCoI1-Luc2 (6844 bp)

```
PaeR7I PspXI XhoI NheI SfiI KpnI SacI BmtI BglI Acc65I Eco53kI TliI
GGCCTAACTGGCCGGTACCTGAGCTCGCTAGCCTCGAGTTTACTCCCTATCAGTGATAGAGAACGTATGTCGAGTTTACTCCCTATCAGTGATAGAGAACGATGTCG
CCGGATTGACCGGCCATGGACTCGAGCGATCGGAGCTCAAATGAGGGATAGTCACTATCTCTTGCATACAGCTCAAATGAGGGATAGTCACTATCTCTTGCTACAGC
TRE Tight
MCS
10 20 30 40 50 60 70 80 90 100
```

```
AGTTTACTCCCTATCAGTGATAGAGAACGTATGTCGAGTTTACTCCCTATCAGTGATAGAGAACGTATGTCGAGTTTACTCCCTATCAGTGATAGAGAACGTATGTC
TCAAATGAGGGATAGTCACTATCTCTTGCATACAGCTCAAATGAGGGATAGTCACTATCTCTTGCATACAGCTCAAATGAGGGATAGTCACTATCTCTTGCATACAG
TRE Tight
MCS
110 120 130 140 150 160 170 180 190 200 210
```

```
StuI
GAGTTTATCCCTATCAGTGATAGAGAACGTATGTCGAGTTTACTCCCTATCAGTGATAGAGAACGTATGTCGAGGTAGGCGTGTACGGTGGGAGGCCTATATAAGCA
CTCAAATAGGGATAGTCACTATCTCTTGCATACAGCTCAAATGAGGGATAGTCACTATCTCTTGCATACAGCTCCATCCGCACATGCCACCCTCCGGATATATTCGT
minCMV
TRE Tight
MCS
220 230 240 250 260 270 280 290 300 310 320
```

```
SacI Eco53kI EcoRI HindIII ApaI PspOMI
GAGCTCGTTTAGTGAACCGTCAGATCGCCTGGAGAATTCAAGCTTGGCAATCCGGTACTGTTGGTAAAGCCACCATGGAAGATGCCAAAAACATTAAGAAGGGCCCA
CTCGAGCAAATCACTTGGCAGTCTAGCGGACCTCTTAAGTTCGAACCGTTAGGCCATGACAACCATTTCGGTGGTACCTTCTACGGTTTTTGTAATTCTTCCCGGGT
M E D A K N I K K G P
minCMV
TRE Tight
MCS
Luc2
330 340 350 360 370 380 390 400 410 420
```

```
SgrAI MreI PvuII
GCGCCATTCTACCCACTCGAAGACGGGACCGCCGGCGAGCAGCTGCACAAAGCCATGAAGCGCTACGCCCTGGTGCCCGGCACCATCGCCTTTACCGACGCACATAT
CGCGGTAAGATGGGTGAGCTTCTGCCCTGGCGGCCGCTCGTCGACGTGTTTCGGTACTTCGCGATGCGGGACCACGGGCCGTGGTAGCGGAAATGGCTGCGTGTATA
A P F Y P L E D G T A G E Q L H K A M K R Y A L V P G T I A F T D A H I
Luc2
430 440 450 460 470 480 490 500 510 520 530
```

Tight-pNLCol1-Luc2 (6844 bp) (from 536-1177 bp)

CGAGGTGGACATTACCTACGCCGAGTACTTCGAGATGAGCGTTCGGCTGGCAGAAGCTATGAAGCGCTATGGGCTGAATACAAACCATCGGATCGTGGTGTGCAGCG
GCTCCACCTGTAATGGATGCGGCTCATGAAGCTCTACTCGCAAGCCGACCGTCTTCGATACTTCGCGATACCCGACTTATGTTTGGTAGCCTAGCACCACACGTCGC
E V D I T Y A E Y F E M S V R L A E A M K R Y G L N T N H R I V V C S

Luc2

540 550 560 570 580 590 600 610 620 630 640

AGAATAGCTTGCAGTTCTTCATGCCCGTGTTGGGTGCCCTGTTCATCGGTGTGGCTGTGGCCCCAGCTAACGACATCTACAACGAGCGCGAGCTGCTGAACAGCATG
TCTTATCGAACGTCAAGAAGTACGGGCACAACCCACGGGACAAGTAGCCACACCGACACCGGGGTCGATTGCTGTAGATGTTGCTCGCGCTCGACGACTTGTCGTAC
E N S L Q F F M P V L G A L F I G V A V A P A N D I Y N E R E L L N S M

Luc2

650 660 670 680 690 700 710 720 730 740

GGCATCAGCCAGCCCACCGTCGTATTCGTGAGCAAGAAAGGGCTGCAAAAGATCCTCAACGTGCAAAAGAAGCTACCGATCATACAAAAGATCATCATCATGGATAG
CCGTAGTCGGTCGGGTGGCAGCATAAGCACTCGTTCTTTCCCGACGTTTTCTAGGAGTTGCACGTTTTCTTCGATGGCTAGTATGTTTTCTAGTAGTAGTACCTATC
G I S Q P T V V F V S K K G L Q K I L N V Q K K L P I I Q K I I I M D S

Luc2

750 760 770 780 790 800 810 820 830 840 850

BsrGI AleI

CAAGACCGACTACCAGGGCTTCCAAAGCATGTACACCTTCGTGACTTCCCATTTGCCACCCGGCTTCAACGAGTACGACTTCGTGCCCGAGAGCTTCGACCGGGACA
GTTCTGGCTGATGGTCCCGAAGGTTTCGTACATGTGGAAGCACTGAAGGGTAAACGGTGGGCCGAAGTTGCTCATGCTGAAGCACGGGCTCTCGAAGCTGGCCCTGT
K T D Y Q G F Q S M Y T F V T S H L P P G F N E Y D F V P E S F D R D

Luc2

860 870 880 890 900 910 920 930 940 950 960

AAACCATCGCCCTGATCATGAACAGTAGTGGCAGTACCGGATTGCCCAAGGGCGTAGCCCTACCGCACCGCACCGCTTGTGTCCGATTCAGTCATGCCCGCGACCCC
TTTGGTAGCGGGACTAGTACTTGTCATCACCGTCATGGCCTAACGGGTTCCCGCATCGGGATGGCGTGGCGTGGCGAACACAGGCTAAGTCAGTACGGGCGCTGGGG
K T I A L I M N S S G S T G L P K G V A L P H R T A C V R F S H A R D P

Luc2

970 980 990 1,000 1,010 1,020 1,030 1,040 1,050 1,060 1,070

BbvCI

ATCTTCGGCAACCAGATCATCCCCGACACCGCTATCCTCAGCGTGGTGCCATTTCACCACGGCTTCGGCATGTTCACCACGCTGGGCTACTTGATCTGCGGCTTTCG
TAGAAGCCGTTGGTCTAGTAGGGGCTGTGGCGATAGGAGTCGCACCACGGTAAAGTGGTGCCGAAGCCGTACAAGTGGTGCGACCCGATGAACTAGACGCCGAAAGC
I F G N Q I I P D T A I L S V V P F H H G F G M F T T L G Y L I C G F R

Luc2

1,080 1,090 1,100 1,110 1,120 1,130 1,140 1,150 1,160 1,170

Tight-pNLCol1-Luc2 (6844 bp) (from 1178-1819 bp)

GGTCGTGCTCATGTACCGCTTCGAGGAGGAGCTATTCTTGCGCAGCTTGCAAGACTATAAGATTCAATCTGCCCTGCTGGTGCCCACACTATTTAGCTTCTTCGCTA
CCAGCACGAGTACATGGCGAAGCTCCTCCTCGATAAGAACGCGTCGAACGTTCTGATATTCTAAGTTAGACGGGACGACCACGGGTGTGATAAATCGAAGAAGCGAT
V V L M Y R F E E E L F L R S L Q D Y K I Q S A L L V P T L F S F F A

Luc2

1,180 1,190 1,200 1,210 1,220 1,230 1,240 1,250 1,260 1,270 1,280

PluTI
SfoI
NarI
KasI BlpI

AGAGCACTCTCATCGACAAGTACGACCTAAGCAACTTGCACGAGATCGCCAGCGGCGGGGCGCCGCTCAGCAAGGAGGTAGGTGAGGCCGTGGCCAAACGCTTCCAC
TCTCGTGAGAGTAGCTGTTCATGCTGGATTCGTTGAACGTGCTCTAGCGGTCGCCGCCCCGCGGCGAGTCGTTCCTCCATCCACTCCGGCACCGGTTTGCGAAGGTG
K S T L I D K Y D L S N L H E I A S G G A P L S K E V G E A V A K R F H

Luc2

1,290 1,300 1,310 1,320 1,330 1,340 1,350 1,360 1,370 1,380 1,390

CTACCAGGCATCCGCCAGGGCTACGGCCTGACAGAAACAACCAGCGCCATTCTGATCACCCCCGAAGGGGACGACAAGCCTGGCGCAGTAGGCAAGGTGGTGCCCTT
GATGGTCCGTAGGCGGTCCCGATGCCGGACTGTCTTTGTTGGTCGCGGTAAGACTAGTGGGGGCTTCCCCTGCTGTTCGGACCGCGTCATCCGTTCCACCACGGGAA
L P G I R Q G Y G L T E T T S A I L I T P E G D D K P G A V G K V V P F

Luc2

1,400 1,410 1,420 1,430 1,440 1,450 1,460 1,470 1,480 1,490

AgeI DraIII HpaI

CTTCGAGGCTAAGGTGGTGGACTTGGACACCGGTAAGACACTGGGTGTGAACCAGCGCGGCGAGCTGTGCGTCCGTGGCCCCATGATCATGAGCGGCTACGTTAACA
GAAGCTCCGATTCCACCACCTGAACCTGTGGCCATTCTGTGACCCACACTTGGTCGCGCCGCTCGACACGCAGGCACCGGGGTACTAGTACTCGCCGATGCAATTGT
F E A K V V D L D T G K T L G V N Q R G E L C V R G P M I M S G Y V N

Luc2

1,500 1,510 1,520 1,530 1,540 1,550 1,560 1,570 1,580 1,590 1,600

ACCCCGAGGCTACAAACGCTCTCATCGACAAGGACGGCTGGCTGCACAGCGGCGACATCGCCTACTGGGACGAGGACGAGCACTTCTTCATCGTGGACCGGCTGAAG
TGGGGCTCCGATGTTTGCGAGAGTAGCTGTTCCTGCCGACCGACGTGTCGCCGCTGTAGCGGATGACCCTGCTCCTGCTCGTGAAGAAGTAGCACCTGGCCGACTTC
N P E A T N A L I D K D G W L H S G D I A Y W D E D E H F F I V D R L K

Luc2

1,610 1,620 1,630 1,640 1,650 1,660 1,670 1,680 1,690 1,700 1,710

SexAI

AGCCTGATCAAATACAAGGGCTACCAGGTAGCCCCAGCCGAACTGGAGAGCATCCTGCTGCAACACCCCAACATCTTCGACGCCGGGGTCGCCGGCCTGCCCGACGA
TCGGACTAGTTTATGTTCCCGATGGTCCATCGGGGTCGGCTTGACCTCTCGTAGGACGACGTTGTGGGGTTGTAGAAGCTGCGGCCCCAGCGGCCGGACGGGCTGCT
S L I K Y K G Y Q V A P A E L E S I L L Q H P N I F D A G V A G L P D D

Luc2

1,720 1,730 1,740 1,750 1,760 1,770 1,780 1,790 1,800 1,810

Tight-pNLCol1-Luc2 (6844 bp) (from 1820-2461 bp)

CGATGCCGGCGAGCTGCCCGCCGCAGTCGTCGTGCTGGAACACGGTAAAACCATGACCGAGAAGGAGATCGTGGACTATGTGGCCAGCCAGGTTACAACCGCCAAGA
GCTACGGCCGCTCGACGGGCGGCGTCAGCAGCACGACCTTGTGCCATTTTGGTACTGGCTCTTCCTCTAGCACCTGATACACCGGTCGGTCCAATGTTGGCGGTTCT
D A G E L P A A V V V L E H G K T M T E K E I V D Y V A S Q V T T A K
Luc2
1,820 1,830 1,840 1,850 1,860 1,870 1,880 1,890 1,900 1,910 1,920

AGCTGCGCGGTGGTGTTGTGTTCGTGGACGAGGTGCCTAAAGGACTGACCGGCAAGTTGGACGCCCGCAAGATCCGCGAGATTCTCATTAAGGCCAAGAAGGGCGGC
TCGACGCGCCACCACAACACAAGCACCTGCTCCACGGATTTCCTGACTGGCCGTTCAACCTGCGGGCGTTCTAGGCGCTCTAAGAGTAATTCCGGTTCTTCCCGCCG
K L R G G V V F V D E V P K G L T G K L D A R K I R E I L I K A K K G G
Luc2
1,930 1,940 1,950 1,960 1,970 1,980 1,990 2,000 2,010 2,020 2,030

Esp3I
BsmBI
BsmAI
BarI BcoDI BmgBI
AAGATCGCCGTGtgaAGCGGAGCTACTAACTTCAGCCTGCTGAAGCAGGCTGGAGACGTGGAGGAGAACCCTGGACCTATGGTCTTCACACTCGAAGATTTCGTTGG
TTCTAGCGGCACactTCGCCTCGATGATTGAAGTCGGACGACTTCGTCCGACCTCTGCACCTCCTCTTGGGACCTGGATACCAGAAGTGTGAGCTTCTAAAGCAACC
K I A V * S G A T N F S L L K Q A G D V E E N P G P M V F T L E D F V G
Luc2 P2A NLucP
2,040 2,050 2,060 2,070 2,080 2,090 2,100 2,110 2,120 2,130 2,140

Tth111I
PflFI EcoNI
GGACTGGCGACAGACAGCCGGCTACAACCTGGACCAAGTCCTTGAACAGGGAGGTGTGTCCAGTTTGTTTCAGAATCTCGGGGTGTCCGTAACTCCGATCCAAAGGA
CCTGACCGCTGTCTGTCGGCCGATGTTGGACCTGGTTCAGGAACTTGTCCCTCCACACAGGTCAAACAAAGTCTTAGAGCCCCACAGGCATTGAGGCTAGGTTTCCT
D W R Q T A G Y N L D Q V L E Q G G V S S L F Q N L G V S V T P I Q R
NLucP
2,150 2,160 2,170 2,180 2,190 2,200 2,210 2,220 2,230 2,240

TTGTCCTGAGCGGTGAAAATGGGCTGAAGATCGACATCCATGTCATCATCCCGTATGAAGGTCTGAGCGGCGACCAAATGGGCCAGATCGAAAAAATTTTTAAGGTG
AACAGGACTCGCCACTTTTACCCGACTTCTAGCTGTAGGTACAGTAGTAGGGCATACTTCCAGACTCGCCGCTGGTTTACCCGGTCTAGCTTTTTTAAAAATTCCAC
I V L S G E N G L K I D I H V I I P Y E G L S G D Q M G Q I E K I F K V
NLucP
2,250 2,260 2,270 2,280 2,290 2,300 2,310 2,320 2,330 2,340 2,350

GTGTACCCTGTGGATGATCATCACTTTAAGGTGATCCTGCACTATGGCACACTGGTAATCGACGGGGTTACGCCGAACATGATCGACTATTTCGGACGGCCGTATGA
CACATGGGACACCTACTAGTAGTGAAATTCCACTAGGACGTGATACCGTGTGACCATTAGCTGCCCCAATGCGGCTTGTACTAGCTGATAAAGCCTGCCGGCATACT
V Y P V D D H H F K V I L H Y G T L V I D G V T P N M I D Y F G R P Y E
NLucP
2,360 2,370 2,380 2,390 2,400 2,410 2,420 2,430 2,440 2,450 2,460

Tight-pNLCol1-Luc2 (6844 bp) (from 2462-3103 bp)

PpuMI
KflI

AGGCATCGCCGTGTTCGACGGCAAAAAGATCACTGTAACAGGGACCCTGTGGAACGGCAACAAAATTATCGACGAGCGCCTGATCAACCCCGACGGCTCCCTGCTGT
TCCGTAGCGGCACAAGCTGCCGTTTTTCTAGTGACATTGTCCCTGGGACACCTTGCCGTTGTTTTAATAGCTGCTCGCGGACTAGTTGGGGCTGCCGAGGGACGACA

G I A V F D G K K I T V T G T L W N G N K I I D E R L I N P D G S L L

NLucP

2,470 2,480 2,490 2,500 2,510 2,520 2,530 2,540 2,550 2,560

XmnI EcoRI Bsu36I

TCCGAGTAACCATCAACGGAGTGACCGGCTGGCGGCTGTGCGAACGCATTCTGGCGAATTCTCACGGCTTTCCGCCTGAGGTTGAAGAGCAAGCCGCCGGTACATTG
AGGCTCATTGGTAGTTGCCTCACTGGCCGACCGCCGACACGCTTGCGTAAGACCGCTTAAGAGTGCCGAAAGGCGGACTCCAACTTCTCGTTCGGCGGCCATGTAAC

F R V T I N G V T G W R L C E R I L A N S H G F P P E V E E Q A A G T L

PEST

NLucP

2,570 2,580 2,590 2,600 2,610 2,620 2,630 2,640 2,650 2,660 2,670

XbaI FseI

CCTATGTCCTGCGCACAAGAAAGCGGTATGGACCGGCACCCAGCCGCTTGTGCTTCAGCTCGCATCAACGTCTAAGGCCGCGACTCTAGAGTCGGGGCGGCCGGCCG
GGATACAGGACGCGTGTTCTTTCGCCATACCTGGCCGTGGGTCGGCGAACACGAAGTCGAGCGTAGTTGCAGATTCCGGCGCTGAGATCTCAGCCCCGCCGGCCGGC

P M S C A Q E S G M D R H P A A C A S A R I N V *

PEST

NLucP

2,680 2,690 2,700 2,710 2,720 2,730 2,740 2,750 2,760 2,770 2,780

CTTCGAGCAGACATGATAAGATACATTGATGAGTTTGGACAAACCACAACTAGAATGCAGTGAAAAAAATGCTTTATTTGTGAAATTTGTGATGCTATTGCTTTATT
GAAGCTCGTCTGTACTATTCTATGTAACTACTCAAACCTGTTTGGTGTTGATCTTACGTCACTTTTTTTACGAAATAAACACTTTAAACACTACGATAACGAAATAA

SV40 late polyA

2,790 2,800 2,810 2,820 2,830 2,840 2,850 2,860 2,870 2,880

PsiI HpaI MfeI

TGTAACCATTATAAGCTGCAATAAACAAGTTAACAACAACAATTGCATTCATTTTATGTTTCAGGTTCAGGGGGAGGTGTGGGAGGTTTTTTAAAGCAAGTAAAACC
ACATTGGTAATATTCGACGTTATTTGTTCAATTGTTGTTGTTAACGTAAGTAAAATACAAAGTCCAAGTCCCCCTCCACACCCTCCAAAAAATTTCGTTCATTTTGG

SV40 late polyA

2,890 2,900 2,910 2,920 2,930 2,940 2,950 2,960 2,970 2,980 2,990

BspDI
ClaI BamHI

TCTACAAATGTGGTAAAATCGATAAGGATCCGTTTGCGTATTGGGCGCTCTTCCGCTGATCTGCGCAGCACCATGGCCTGAAATAACCTCTGAAAGAGGAACTTGGT
AGATGTTTACACCATTTTAGCTATTCCTAGGCAAACGCATAACCCGCGAGAAGGCGACTAGACGCGTCGTGGTACCGGACTTTATTGGAGACTTTCTCCTTGAACCA

3,000 3,010 3,020 3,030 3,040 3,050 3,060 3,070 3,080 3,090 3,100

NsiI SphI BstAPI PvuII

TAGCTACCTTCTGAGGCGGAAAGAACCAGCTGTGGAATGTGTGTCAGTTAGGGTGTGGAAAGTCCCCAGGCTCCCCAGCAGGCAGAAGTATGCAAAGCATGCATCTC
ATCGATGGAAGACTCCGCCTTTCTTGGTCGACACCTTACACACAGTCAATCCCACACCTTTCAGGGGTCCGAGGGGTCGTCCGTCTTCATACGTTTCGTACGTAGAG

3,110 3,120 3,130 3,140 3,150 3,160 3,170 3,180 3,190 3,200 3,210

NsiI SphI SexAI BstAPI

AATTAGTCAGCAACCAGGTGTGGAAAGTCCCCAGGCTCCCCAGCAGGCAGAAGTATGCAAAGCATGCATCTCAATTAGTCAGCAACCATAGTCCCGCCCCTAACTCC
TTAATCAGTCGTTGGTCCACACCTTTCAGGGGTCCGAGGGGTCGTCCGTCTTCATACGTTTCGTACGTAGAGTTAATCAGTCGTTGGTATCAGGGCGGGGATTGAGG

3,220 3,230 3,240 3,250 3,260 3,270 3,280 3,290 3,300 3,310

BplI BplI

GCCCATCCCGCCCCTAACTCCGCCCAGTTCCGCCCATTCTCCGCCCCATGGCTGACTAATTTTTTTTATTTATGCAGAGGCCGAGGCCGCCTCTGCCTCTGAGCTAT
CGGGTAGGGCGGGGATTGAGGCGGGTCAAGGCGGGTAAGAGGCGGGGTACCGACTGATTAAAAAAAATAAATACGTCTCCGGCTCCGGCGGAGACGGAGACTCGATA

3,320 3,330 3,340 3,350 3,360 3,370 3,380 3,390 3,400 3,410 3,420

AvrII StuI

TCCAGAAGTAGTGAGGAGGCTTTTTTGGAGGCCTAGGCTTTTGCAAAAAGCTCGATTCTTCTGACACTAGCGCCACCATGAAGAAGCCCGAACTCACCGCTACCAGC
AGGTCTTCATCACTCCTCCGAAAAAACCTCCGGATCCGAAAACGTTTTTCGAGCTAAGAAGACTGTGATCGCGGTGGTACTTCTTCGGGCTTGAGTGGCGATGGTCG

3,430 3,440 3,450 3,460 3,470 3,480 3,490 3,500 3,510 3,520 3,530

XmnI

GTTGAAAAATTTCTCATCGAGAAGTTCGACAGTGTGAGCGACCTGATGCAGTTGTCGGAGGGCGAAGAGAGCCGAGCCTTCAGCTTCGATGTCGGCGGACGCGGCTA
CAACTTTTTAAAGAGTAGCTCTTCAAGCTGTCACACTCGCTGGACTACGTCAACAGCCTCCCGCTTCTCTCGGCTCGGAAGTCGAAGCTACAGCCGCCTGCGCCGAT

3,540 3,550 3,560 3,570 3,580 3,590 3,600 3,610 3,620 3,630

BsaAI AflIII BarI

TGTACTGCGGGTGAATAGCTGCGCTGATGGCTTCTACAAAGACCGCTACGTGTACCGCCACTTCGCCAGCGCTGCACTACCCATCCCCGAAGTGTTGGACATCGGCG
ACATGACGCCCACTTATCGACGCGACTACCGAAGATGTTTCTGGCGATGCACATGGCGGTGAAGCGGTCGCGACGTGATGGGTAGGGGCTTCACAACCTGTAGCCGC

3,640 3,650 3,660 3,670 3,680 3,690 3,700 3,710 3,720 3,730 3,740

AGTTCAGCGAGAGCCTGACATACTGCATCAGTAGACGCGCCCAAGGCGTTACTCTCCAAGACCTCCCCGAAACAGAGCTGCCTGCTGTGTTACAGCCTGTCGCCGAA
TCAAGTCGCTCTCGGACTGTATGACGTAGTCATCTGCGCGGGTTCCGCAATGAGAGGTTCTGGAGGGGCTTTGTCTCGACGGACGACACAATGTCGGACAGCGGCTT

3,750 3,760 3,770 3,780 3,790 3,800 3,810 3,820 3,830 3,840 3,850

Tight-pNLCol1-Luc2 (6844 bp) (from 3853-4815 bp)

PshAI PspOMI ApaI

GCTATGGATGCTATTGCCGCCGCCGACCTCAGTCAAACCAGCGGCTTCGGCCCATTCGGGCCCCAAGGCATCGGCCAGTACACAACCTGGCGGGATTTCATTTGCGC
CGATACCTACGATAACGGCGGCGGCTGGAGTCAGTTTGGTCGCCGAAGCCGGGTAAGCCCGGGGTTCCGTAGCCGGTCATGTGTTGGACCGCCCTAAAGTAAACGCG

3,860 3,870 3,880 3,890 3,900 3,910 3,920 3,930 3,940 3,950

CATTGCTGATCCCCATGTCTACCACTGGCAGACCGTGATGGACGACACCGTGTCCGCCAGCGTAGCTCAAGCCCTGGACGAACTGATGCTGTGGGCCGAAGACTGTC
GTAACGACTAGGGGTACAGATGGTGACCGTCTGGCACTACCTGCTGTGGCACAGGCGGTCGCATCGAGTTCGGGACCTGCTTGACTACGACACCCGGCTTCTGACAG

3,960 3,970 3,980 3,990 4,000 4,010 4,020 4,030 4,040 4,050 4,060

CCGAGGTGCGCCACCTCGTCCATGCCGACTTCGGCAGCAACAACGTCCTGACCGACAACGGCCGCATCACCGCCGTAATCGACTGGTCCGAAGCTATGTTCGGGGAC
GGCTCCACGCGGTGGAGCAGGTACGGCTGAAGCCGTCGTTGTTGCAGGACTGGCTGTTGCCGGCGTAGTGGCGGCATTAGCTGACCAGGCTTCGATACAAGCCCCTG

4,070 4,080 4,090 4,100 4,110 4,120 4,130 4,140 4,150 4,160 4,170

AGTCAGTACGAGGTGGCCAACATCTTCTTCTGGCGGCCCTGGCTGGCTTGCATGGAGCAGCAGACTCGCTACTTCGAGCGCCGGCATCCCGAGCTGGCCGGCAGCCC
TCAGTCATGCTCCACCGGTTGTAGAAGAAGACCGCCGGGACCGACCGAACGTACCTCGTCGTCTGAGCGATGAAGCTCGCGGCCGTAGGGCTCGACCGGCCGTCGGG

4,180 4,190 4,200 4,210 4,220 4,230 4,240 4,250 4,260 4,270 4,280

TCGTCTGCGAGCCTACATGCTGCGCATCGGCCTGGATCAGCTCTACCAGAGCCTCGTGGACGGCAACTTCGACGATGCTGCCTGGGCTCAAGGCCGCTGCGATGCCA
AGCAGACGCTCGGATGTACGACGCGTAGCCGGACCTAGTCGAGATGGTCTCGGAGCACCTGCCGTTGAAGCTGCTACGACGGACCCGAGTTCCGGCGACGCTACGGT

4,290 4,300 4,310 4,320 4,330 4,340 4,350 4,360 4,370 4,380

TCGTCCGCAGCGGGGCCGGCACCGTCGGTCGCACACAAATCGCTCGCCGGAGCGCAGCCGTATGGACCGACGGCTGCGTCGAGGTGCTGGCCGACAGCGGCAACCGC
AGCAGGCGTCGCCCCGGCCGTGGCAGCCAGCGTGTGTTTAGCGAGCGGCCTCGCGTCGGCATACCTGGCTGCCGACGCAGCTCCACGACCGGCTGTCGCCGTTGGCG

4,390 4,400 4,410 4,420 4,430 4,440 4,450 4,460 4,470 4,480 4,490

BssHII PmeI XbaI AgeI

CGGCCCAGTACACGACCGCGCGCTAAGGAGGTAGGTCGAGTTTAAACTCTAGAACCGGTCATGGCCGCAATAAAATATCTTTATTTTCATTACATCTGTGTGTTGGT
GCCGGGTCATGTGCTGGCGCGCGATTCCTCCATCCAGCTCAAATTTGAGATCTTGGCCAGTACCGGCGTTATTTTATAGAAATAAAAGTAATGTAGACACACAACCA

4,500 4,510 4,520 4,530 4,540 4,550 4,560 4,570 4,580 4,590 4,600

BstBI SalI PshAI

TTTTTGTGTGTTCGAACTAGATGCTGTCGACCGATGCCCTTGAGAGCCTTCAACCCAGTCAGCTCCTTCCGGTGGGCGCGGGGCATGACTATCGTCGCCGCACTTAT
AAAAACACACAAGCTTGATCTACGACAGCTGGCTACGGGAACTCTCGGAAGTTGGGTCAGTCGAGGAAGGCCACCCGCGCCCCGTACTGATAGCAGCGGCGTGAATA

4,610 4,620 4,630 4,640 4,650 4,660 4,670 4,680 4,690 4,700

GACTGTCTTCTTTATCATGCAACTCGTAGGACAGGTGCCGGCAGCGCTCTTCCGCTTCCTCGCTCACTGACTCGCTGCGCTCGGTCGTTCGGCTGCGGCGAGCGGTA
CTGACAGAAGAAATAGTACGTTGAGCATCCTGTCCACGGCCGTCGCGAGAAGGCGAAGGAGCGAGTGACTGAGCGACGCGAGCCAGCAAGCCGACGCCGCTCGCCAT

4,710 4,720 4,730 4,740 4,750 4,760 4,770 4,780 4,790 4,800 4,810

Tight-pNLCol1-Luc2 (6844 bp) (from 4816-5564 bp)

AflIII
PciI

```
TCAGCTCACTCAAAGGCGGTAATACGGTTATCCACAGAATCAGGGGATAACGCAGGAAAGAACATGTGAGCAAAAGGCCAGCAAAAGGCCAGGAACCGTAAAAAGGC
AGTCGAGTGAGTTTCCGCCATTATGCCAATAGGTGTCTTAGTCCCCTATTGCGTCCTTTCTTGTACACTCGTTTTCCGGTCGTTTTCCGGTCCTTGGCATTTTTCCG
```

4,820 4,830 4,840 4,850 4,860 4,870 4,880 4,890 4,900 4,910 4,920

```
CGCGTTGCTGGCGTTTTTCCATAGGCTCCGCCCCCCTGACGAGCATCACAAAAATCGACGCTCAAGTCAGAGGTGGCGAAACCCGACAGGACTATAAAGATACCAGG
GCGCAACGACCGCAAAAAGGTATCCGAGGCGGGGGGACTGCTCGTAGTGTTTTTAGCTGCGAGTTCAGTCTCCACCGCTTTGGGCTGTCCTGATATTTCTATGGTCC
```

ColE1 origin

4,930 4,940 4,950 4,960 4,970 4,980 4,990 5,000 5,010 5,020

BciVI

```
CGTTTCCCCCTGGAAGCTCCCTCGTGCGCTCTCCTGTTCCGACCCTGCCGCTTACCGGATACCTGTCCGCCTTTCTCCCTTCGGGAAGCGTGGCGCTTTCTCATAGC
GCAAAGGGGGACCTTCGAGGGAGCACGCGAGAGGACAAGGCTGGGACGGCGAATGGCCTATGGACAGGCGGAAAGAGGGAAGCCCTTCGCACCGCGAAAGAGTATCG
```

ColE1 origin

5,030 5,040 5,050 5,060 5,070 5,080 5,090 5,100 5,110 5,120 5,130

ApaLI

```
TCACGCTGTAGGTATCTCAGTTCGGTGTAGGTCGTTCGCTCCAAGCTGGGCTGTGTGCACGAACCCCCCGTTCAGCCCGACCGCTGCGCCTTATCCGGTAACTATCG
AGTGCGACATCCATAGAGTCAAGCCACATCCAGCAAGCGAGGTTCGACCCGACACACGTGCTTGGGGGGCAAGTCGGGCTGGCGACGCGGAATAGGCCATTGATAGC
```

ColE1 origin

5,140 5,150 5,160 5,170 5,180 5,190 5,200 5,210 5,220 5,230 5,240

AlwNI

```
TCTTGAGTCCAACCCGGTAAGACACGACTTATCGCCACTGGCAGCAGCCACTGGTAACAGGATTAGCAGAGCGAGGTATGTAGGCGGTGCTACAGAGTTCTTGAAGT
AGAACTCAGGTTGGGCCATTCTGTGCTGAATAGCGGTGACCGTCGTCGGTGACCATTGTCCTAATCGTCTCGCTCCATACATCCGCCACGATGTCTCAAGAACTTCA
```

ColE1 origin

5,250 5,260 5,270 5,280 5,290 5,300 5,310 5,320 5,330 5,340 5,350

```
GGTGGCCTAACTACGGCTACACTAGAAGAACAGTATTTGGTATCTGCGCTCTGCTGAAGCCAGTTACCTTCGGAAAAAGAGTTGGTAGCTCTTGATCCGGCAAACAA
CCACCGGATTGATGCCGATGTGATCTTCTTGTCATAAACCATAGACGCGAGACGACTTCGGTCAATGGAAGCCTTTTTCTCAACCATCGAGAACTAGGCCGTTTGTT
```

ColE1 origin

5,360 5,370 5,380 5,390 5,400 5,410 5,420 5,430 5,440 5,450

```
ACCACCGCTGGTAGCGGTGGTTTTTTTGTTTGCAAGCAGCAGATTACGCGCAGAAAAAAAGGATCTCAAGAAGATCCTTTGATCTTTTCTACGGGGTCTGACGCTCA
TGGTGGCGACCATCGCCACCAAAAAAACAAACGTTCGTCGTCTAATGCGCGTCTTTTTTTCCTAGAGTTCTTCTAGGAAACTAGAAAAGATGCCCCAGACTGCGAGT
```

ColE1 origin

5,460 5,470 5,480 5,490 5,500 5,510 5,520 5,530 5,540 5,550 5,560

Tight-pNLCol1-Luc2 (6844 bp) (from 5565-6527 bp)

GTGGAACGAAAACTCACGTTAAGGGATTTTGGTCATGAGATTATCAAAAAGGATCTTCACCTAGATCCTTTTAAATTAAAAATGAAGTTTTAAATCAATCTAAAGTA
CACCTTGCTTTTGAGTGCAATTCCCTAAAACCAGTACTCTAATAGTTTTTCCTAGAAGTGGATCTAGGAAAATTTAATTTTTACTTCAAAATTTAGTTAGATTTCAT

ColE1 origin

5,570 5,580 5,590 5,600 5,610 5,620 5,630 5,640 5,650 5,660 5,670

BstEII
BstXI
PstI
NotI AleI

TATATGAGTAAACTTGGTCTGACAGCGGCCGCAAATGCTAAACCACTGCAGTGGTTACCAGTGCTTGATCAGTGAGGCACCGATCTCAGCGATCTGCCTATTTCGTT
ATATACTCATTTGAACCAGACTGTCGCCGGCGTTTACGATTTGGTGACGTCACCAATGGTCACGAACTAGTCACTCCGTGGCTAGAGTCGCTAGACGGATAAAGCAA

5,680 5,690 5,700 5,710 5,720 5,730 5,740 5,750 5,760 5,770

CGTCCATAGTGGCCTGACTCCCCGTCGTGTAGATCACTACGATTCGTGAGGGCTTACCATCAGGCCCCAGCGCAGCAATGATGCCGCGAGAGCCGCGTTCACCGGCC
GCAGGTATCACCGGACTGAGGGGCAGCACATCTAGTGATGCTAAGCACTCCCGAATGGTAGTCCGGGGTCGCGTCGTTACTACGGCGCTCTCGGCGCAAGTGGCCGG

5,780 5,790 5,800 5,810 5,820 5,830 5,840 5,850 5,860 5,870 5,880

CCCGATTTGTCAGCAATGAACCAGCCAGCAGGGAGGGCCGAGCGAAGAAGTGGTCCTGCTACTTTGTCCGCCTCCATCCAGTCTATGAGCTGCTGTCGTGATGCTAG
GGGCTAAACAGTCGTTACTTGGTCGGTCGTCCCTCCCGGCTCGCTTCTTCACCAGGACGATGAAACAGGCGGAGGTAGGTCAGATACTCGACGACAGCACTACGATC

5,890 5,900 5,910 5,920 5,930 5,940 5,950 5,960 5,970 5,980 5,990

AGTAAGAAGTTCGCCAGTGAGTAGTTTCCGAAGAGTTGTGGCCATTGCTACTGGCATCGTGGTATCACGCTCGTCGTTCGGTATGGCTTCGTTCAACTCTGGTTCCC
TCATTCTTCAAGCGGTCACTCATCAAAGGCTTCTCAACACCGGTAACGATGACCGTAGCACCATAGTGCGAGCAGCAAGCCATACCGAAGCAAGTTGAGACCAAGGG

6,000 6,010 6,020 6,030 6,040 6,050 6,060 6,070 6,080 6,090

Bsu36I PvuI SacII

AGCGGTCAAGCCGGGTCACATGATCACCCATATTATGAAGAAATGCAGTCAGCTCCTTAGGGCCTCCGATCGTTGTCAGAAGTAAGTTGGCCGCGGTGTTGTCGCTC
TCGCCAGTTCGGCCCAGTGTACTAGTGGGTATAATACTTCTTTACGTCAGTCGAGGAATCCCGGAGGCTAGCAACAGTCTTCATTCAACCGGCGCCACAACAGCGAG

6,100 6,110 6,120 6,130 6,140 6,150 6,160 6,170 6,180 6,190 6,200

BstZ17I

ATGGTAATGGCAGCACTACACAATTCTCTTACCGTCATGCCATCCGTAAGATGCTTTTCCGTGACCGGCGAGTACTCAACCAAGTCGTTTTGTGAGTAGTGTATACG
TACCATTACCGTCGTGATGTGTTAAGAGAATGGCAGTACGGTAGGCATTCTACGAAAAGGCACTGGCCGCTCATGAGTTGGTTCAGCAAAACACTCATCACATATGC

6,210 6,220 6,230 6,240 6,250 6,260 6,270 6,280 6,290 6,300 6,310

GCGACCAAGCTGCTCTTGCCCGGCGTCTATACGGGACAACACCGCGCCACATAGCAGTACTTTGAAAGTGCTCATCATCGGGAATCGTTCTTCGGGGCGGAAAGACT
CGCTGGTTCGACGAGAACGGGCCGCAGATATGCCCTGTTGTGGCGCGGTGTATCGTCATGAAACTTTCACGAGTAGTAGCCCTTAGCAAGAAGCCCCGCCTTTCTGA

6,320 6,330 6,340 6,350 6,360 6,370 6,380 6,390 6,400 6,410 6,420

CAAGGATCTTGCCGCTATTGAGATCCAGTTCGATATAGCCCACTCTTGCACCCAGTTGATCTTCAGCATCTTTTACTTTCACCAGCGTTTCGGGGTGTGCAAAAACA
GTTCCTAGAACGGCGATAACTCTAGGTCAAGCTATATCGGGTGAGAACGTGGGTCAACTAGAAGTCGTAGAAAATGAAAGTGGTCGCAAAGCCCCACACGTTTTTGT

6,430 6,440 6,450 6,460 6,470 6,480 6,490 6,500 6,510 6,520

Tight-pNLCol1-Luc2 (6844 bp) (from 6528-6844 bp)

SspI SpeI

```
GGCAAGCAAAATGCCGCAAAGAAGGGAATGAGTGCGACACGAAAATGTTGGATGCTCATACTCGTCCTTTTTCAATATTATTGAAGCATTTATCAGGGTTACTAGTA
CCGTTCGTTTTACGGCGTTTCTTCCCTTACTCACGCTGTGCTTTTACAACCTACGAGTATGAGCAGGAAAAAGTTATAATAACTTCGTAAATAGTCCCAATGATCAT
```

6,530 6,540 6,550 6,560 6,570 6,580 6,590 6,600 6,610 6,620 6,630

Esp3I
BsmBI
BsmAI
BcoDI
SspI

```
CGTCTCTCAAGGATAAGTAAGTAATATTAAGGTACGGGAGGTATTGGACAGGCCGCAATAAAATATCTTTATTTTCATTACATCTGTGTGTTGGTTTTTTGTGTGAA
GCAGAGAGTTCCTATTCATTCATTATAATTCCATGCCCTCCATAACCTGTCCGGCGTTATTTTATAGAAATAAAAGTAATGTAGACACACAACCAAAAAACACACTT
```

6,640 6,650 6,660 6,670 6,680 6,690 6,700 6,710 6,720 6,730 6,740

PaqCI
BspDI BspMI
ClaI BfuAI

```
TCGATAGTACTAACATACGCTCTCCATCAAAACAAAACGAAACAAAACAAACTAGCAAAATAGGCTGTCCCCAGTGCAAGTGCAGGTGCCAGAACATTTCTCT
AGCTATCATGATTGTATGCGAGAGGTAGTTTTGTTTTGCTTTGTTTTGTTTGATCGTTTTATCCGACAGGGGTCACGTTCACGTCCACGGTCTTGTAAAGAGA
```

6,750 6,760 6,770 6,780 6,790 6,800 6,810 6,820 6,830 6,840

(from 1-642 bp)

bArr1-HA-TEV219 pCDH-CuO-MCS-EF1α-CymR-T2A-Bleo...

BspEI PspOMI

GGATCCGGTAGTGGTGGTGGGTCTGGGGGAGGGTCCGGAGGCGGCTCTGGTGGTGGGTCATACCCTTACGACGTACCTGATTACGCGGGCTCATCTCTGTTCAAGGG

CCTAGGCCATCACCACCACCCAGACCCCCTCCCAGGCCTCCGCCGAGACCACCACCCAGTATGGGAATGCTGCATGGACTAATGCGCCCGAGTAGAGACAAGTTCCC

G S G S G G G S G G G S G G G S G G G S Y P Y D V P D Y A G S S L F K G

Linker

HA-Tag

Tev 219

10 20 30 40 50 60 70 80 90 100

ApaI

CCCAAGAGACTATAATCCTATCTCATCAACGATCTGCCATCTCACAAACGAATCCGACGGACACACGACCTCTCTTTACGGGATTGGTTTCGGCCCTTTTATCATAA

GGGTTCTCTGATATTAGGATAGAGTAGTTGCTAGACGGTAGAGTGTTTGCTTAGGCTGCCTGTGTGCTGGAGAGAAATGCCCTAACCAAAGCCGGGAAAATAGTATT

P R D Y N P I S S T I C H L T N E S D G H T T S L Y G I G F G P F I I

Tev 219

110 120 130 140 150 160 170 180 190 200 210

XcmI

CTAATAAACATCTTTTCCGCCGGAACAACGGAACTTTGCTTGTCCAGTCACTTCATGGCGTATTTAAAGTGAAGAATACTACTACTTTGCAACAACATCTCATTGAC

GATTATTTGTAGAAAAGGCGGCCTTGTTGCCTTGAAACGAACAGGTCAGTGAAGTACCGCATAAATTTCACTTCTTATGATGATGAAACGTTGTTGTAGAGTAACTG

T N K H L F R R N N G T L L V Q S L H G V F K V K N T T T L Q Q H L I D

Tev 219

220 230 240 250 260 270 280 290 300 310 320

GGCCGGGACATGATTATTATAAGAATGCCGAAGGATTTTCCACCTTTCCCCCAAAAACTTAAGTTTCGGGAGCCACAACGAGAGGAACGAATATGCCTGGTAACAAC

CCGGCCCTGTACTAATAATATTCTTACGGCTTCCTAAAAGGTGGAAAGGGGGTTTTTGAATTCAAAGCCCTCGGTGTTGCTCTCCTTGCTTATACGGACCATTGTTG

G R D M I I I R M P K D F P P F P Q K L K F R E P Q R E E R I C L V T T

Tev 219

330 340 350 360 370 380 390 400 410 420

BsrGI

AAATTTTCAAACTAAATCTATGTCTTCAATGGTGAGTGACACATCTTGTACATTTCCCTCTAGTGACGGGATTTTCTGGAAGCATTGGATACAGACTAAGGACGGGC

TTTAAAAGTTTGATTTAGATACAGAAGTTACCACTCACTGTGTAGAACATGTAAAGGGAGATCACTGCCCTAAAAGACCTTCGTAACCTATGTCTGATTCCTGCCCG

N F Q T K S M S S M V S D T S C T F P S S D G I F W K H W I Q T K D G

Tev 219

430 440 450 460 470 480 490 500 510 520 530

AGTGCGGATCACCACTCGTGTCCACACGCGATGGATTCATTGTCGGGATTCATAGTGCTTCTAACTTCACCAACACCAATAATTATTTTACTTCTGTGCCAAAAAAC

TCACGCCTAGTGGTGAGCACAGGTGTGCGCTACCTAAGTAACAGCCCTAAGTATCACGAAGATTGAAGTGGTTGTGGTTATTAATAAAATGAAGACACGGTTTTTTG

Q C G S P L V S T R D G F I V G I H S A S N F T N T N N Y F T S V P K N

Tev 219

540 550 560 570 580 590 600 610 620 630 640

bArr1-HA-TEV219 pCDH-CuO-MCS-EF1α-CymR-T2A-Bleo3 SparQ (10934 bp)

AloI Tth111I PflFI

TTCATGGAGCTGCTTACTAGCCAAGAAGCCCAACAGTGGGTGAGTGGGTGGCGCCTGAACGCCGACTCCGTCTTGTGGGGGGGTCACAAGGTCTTTATGGTATAAAT
AAGTACCTCGACGAATGATCGGTTCTTCGGGTTGTCACCCACTCACCCACCGCGGACTTGCGGCTGAGGCAGAACACCCCCCCAGTGTTCCAGAAATACCATATTTA
F M E L L T S Q E A Q Q W V S G W R L N A D S V L W G G H K V F M V *
Tev 219
650 660 670 680 690 700 710 720 730 740

SwaI NotI AsiSI MfeI

TTAAATCGGATCCGCGGCCGCGGATCTGCGATCGCTCCGGTGCCCGTCAGTGGGCAGAGCGCACATCGCCCACAGTCCCCGAGAAGTTGGGGGGAGGGGTCGGCAAT
AATTTAGCCTAGGCGCCGGCGCCTAGACGCTAGCGAGGCCACGGGCAGTCACCCGTCTCGCGTGTAGCGGGTGTCAGGGGCTCTTCAACCCCCCTCCCCAGCCGTTA
EF1 alpha
750 760 770 780 790 800 810 820 830 840 850

TGAACGGGTGCCTAGAGAAGGTGGCGCGGGGTAAACTGGGAAAGTGATGTCGTGTACTGGCTCCGCCTTTTTCCCGAGGGTGGGGGAGAACCGTATATAAGTGCAGT
ACTTGCCCACGGATCTCTTCCACCGCGCCCCATTTGACCCTTTCACTACAGCACATGACCGAGGCGGAAAAAGGGCTCCCACCCCCTCTTGGCATATATTCACGTCA
EF1 alpha
860 870 880 890 900 910 920 930 940 950 960

Bsu36I

AGTCGCCGTGAACGTTCTTTTTCGCAACGGGTTTGCCGCCAGAACACAGCTGAAGCTTCGAGGGGCTCGCATCTCTCCTTCACGCGCCCGCCGCCCTACCTGAGGCC
TCAGCGGCACTTGCAAGAAAAAGCGTTGCCCAAACGGCGGTCTTGTGTCGACTTCGAAGCTCCCCGAGCGTAGAGAGGAAGTGCGCGGGCGGCGGGATGGACTCCGG
EF1 alpha
970 980 990 1,000 1,010 1,020 1,030 1,040 1,050 1,060 1,070

CspCI

GCCATCCACGCCGGTTGAGTCGCGTTCTGCCGCCTCCCGCCTGTGGTGCCTCCTGAACTGCGTCCGCCGTCTAGGTAAGTTTAAAGCTCAGGTCGAGACCGGGCCTT
CGGTAGGTGCGGCCAACTCAGCGCAAGACGGCGGAGGGCGGACACCACGGAGGACTTGACGCAGGCGGCAGATCCATTCAAATTTCGAGTCCAGCTCTGGCCCGGAA
EF1 alpha
1,080 1,090 1,100 1,110 1,120 1,130 1,140 1,150 1,160 1,170

TGTCCGGCGCTCCCTTGGAGCCTACCTAGACTCAGCCGGCTCTCCACGCTTTGCCTGACCCTGCTTGCTCAACTCTACGTCTTTGTTTCGTTTTCTGTTCTGCGCCG
ACAGGCCGCGAGGGAACCTCGGATGGATCTGAGTCGGCCGAGAGGTGCGAAACGGACTGGGACGAACGAGTTGAGATGCAGAAACAAAGCAAAAGACAAGACGCGGC
EF1 alpha
1,180 1,190 1,200 1,210 1,220 1,230 1,240 1,250 1,260 1,270 1,280

XbaI

TTACAGATCCAAGCTGTGACCGGCGCCTACTCTAGAGCCGCCATGGCTTACCCATACGATGTTCCAGATTACGCTAGTTTGGTGAGGCAGCAGAGACCGATGACATC
AATGTCTAGGTTCGACACTGGCCGCGGATGAGATCTCGGCGGTACCGAATGGGTATGCTACAAGGTCTAATGCGATCAAACCACTCCGTCGTCTCTGGCTACTGTAG
M A Y P Y D V P D Y A S L V R Q Q R P M T S
EF1 alpha HA
1,290 1,300 1,310 1,320 1,330 1,340 1,350 1,360 1,370 1,380 1,390

bArr1-HA-TEV219 pCDH-CuO-MCS-EF1α-CymR-T2A-Bleo3 SparQ (10934 bp)

BclI

CACTTTGCCTTTCTCTCCACAGGTGTCCACTCCCAGGTCCAAGTTTGGAAGATCTACCATGCCAAAGAGACCCAGACCCGTGATCATGAGTCCAAAGAGAAGAACAC
GTGAAACGGAAAGAGAGGTGTCCACAGGTGAGGGTCCAGGTTCAAACCTTCTAGATGGTACGGTTTCTCTGGGTCTGGGCACTAGTACTCAGGTTTCTCTTCTTGTG
T L P F S P Q V S T P R S K F G R S T M P K R P R P V I M S P K R R T

CymR

1,400 1,410 1,420 1,430 1,440 1,450 1,460 1,470 1,480 1,490

PasI PasI BspEI PaqCI

AGGCAGAGCGCGCAATGGAGACCCAGGGCAAGTTGATTGCAGCGGCCCTGGGGGTTTTACGGGAAAAAGGTTACGCGGGATTCCGGATCGCAGATGTGCCCGGTGCT
TCCGTCTCGCGCGTTACCTCTGGGTCCCGTTCAACTAACGTCGCCGGGACCCCCAAAATGCCCTTTTTCCAATGCGCCCTAAGGCCTAGCGTCTACACGGGCCACGA
Q A E R A M E T Q G K L I A A A L G V L R E K G Y A G F R I A D V P G A

CymR

1,500 1,510 1,520 1,530 1,540 1,550 1,560 1,570 1,580 1,590 1,600

PaeR7I
XhoI
PstI TliI BcgI

GCAGGTGTCTCGAGAGGAGCGCAGAGCCATCATTTCCCGACAAAGCTTGAGCTTCTGCTTGCCACTTTTGAATGGCTTTACGAACAGATCACCGAACGCAGTCGGGC
CGTCCACAGAGCTCTCCTCGCGTCTCGGTAGTAAAGGGCTGTTTCGAACTCGAAGACGAACGGTGAAAACTTACCGAAATGCTTGTCTAGTGGCTTGCGTCAGCCCG
A G V S R G A Q S H H F P T K L E L L L A T F E W L Y E Q I T E R S R A

CymR

1,610 1,620 1,630 1,640 1,650 1,660 1,670 1,680 1,690 1,700 1,710

TCGATTAGCGAAATTGAAGCCAGAGGATGACGTCATCCAGCAAATGCTGGACGACGCCGCCGAATTTTTCCTCGACGATGACTTCTCTATCAGCCTTGATTTGATTG
AGCTAATCGCTTTAACTTCGGTCTCCTACTGCAGTAGGTCGTTTACGACCTGCTGCGGCGGCTTAAAAAGGAGCTGCTACTGAAGAGATAGTCGGAACTAAACTAAC
R L A K L K P E D D V I Q Q M L D D A A E F F L D D D F S I S L D L I

CymR

1,720 1,730 1,740 1,750 1,760 1,770 1,780 1,790 1,800 1,810

TGGCTGCCGACCGGGATCCAGCGTTACGCGAGGGTATTCAGCGCACGGTAGAGAGGAATCGGTTTGTCGTCGAGGATATGTGGCTTGGTGTTCTGGTGAGCCGTGGT
ACCGACGGCTGGCCCTAGGTCGCAATGCGCTCCCATAAGTCGCGTGCCATCTCTCCTTAGCCAAACAGCAGCTCCTATACACCGAACCACAAGACCACTCGGCACCA
V A A D R D P A L R E G I Q R T V E R N R F V V E D M W L G V L V S R G

CymR

1,820 1,830 1,840 1,850 1,860 1,870 1,880 1,890 1,900 1,910 1,920

EcoRV

CTTTCGCGTGATGATGCAGAAGATATCCTTTGGTTGATATTCAATTCGGTGCGTGGGCTTGCTGTTCGTAGCCTATGGCAGAAGGACAAAGAACGCTTTGAGCGTGT
GAAAGCGCACTACTACGTCTTCTATAGGAAACCAACTATAAGTTAAGCCACGCACCCGAACGACAAGCATCGGATACCGTCTTCCTGTTTCTTGCGAAACTCGCACA
L S R D D A E D I L W L I F N S V R G L A V R S L W Q K D K E R F E R V

CymR

1,930 1,940 1,950 1,960 1,970 1,980 1,990 2,000 2,010 2,020 2,030

bArr1-HA-TEV219 pCDH-CuO-MCS-EF1α-CymR-T2A-Bleo3 SparQ (10934 bp)

CAGGAACTCGACACTCGAAATTGCGCGAGAGCGGTACGCGAAATTCAAGCGCGCGGCCAAACCCTCTGGCCGGTCTCTCTCTGTTCGAGAGGGCAGAGGAAGTCTTC
GTCCTTGAGCTGTGAGCTTTAACGCGCTCTCGCCATGCGCTTTAAGTTCGCGCGCCGGTTTGGGAGACCGGCCAGAGAGAGACAAGCTCTCCCGTCTCCTTCAGAAG
R N S T L E I A R E R Y A K F K R A A K P S G
CymR
T2A
2,040 2,050 2,060 2,070 2,080 2,090 2,100 2,110 2,120 2,130 2,140

BmgBI EcoRI MauBI
TAACATGCGGTGACGTGGAGGAGAATCCCGGCCCTGAATTCATGGCCAAGTTGACCAGTGCCGTTCCGGTGCTCACCGCGCGCGACGTCGCCGGAGCGGTCGAGTTC
ATTGTACGCCACTGCACCTCCTCTTAGGGCCGGGACTTAAGTACCGGTTCAACTGGTCACGGCAAGGCCACGAGTGGCGCGCGCTGCAGCGGCCTCGCCAGCTCAAG
M A K L T S A V P V L T A R D V A G A V E F
T2A
bleo
2,150 2,160 2,170 2,180 2,190 2,200 2,210 2,220 2,230 2,240

SmaI
TspMI
XmaI SgrAI BmgBI SexAI
TGGACCGACCGGCTCGGGTTCTCCCGGGACTTCGTGGAGGACGACTTCGCCGGTGTGGTCCGGGACGACGTGACCCTGTTCATCAGCGCGGTCCAGGACCAGGTGGT
ACCTGGCTGGCCGAGCCCAAGAGGGCCCTGAAGCACCTCCTGCTGAAGCGGCCACACCAGGCCCTGCTGCACTGGGACAAGTAGTCGCGCCAGGTCCTGGTCCACCA
W T D R L G F S R D F V E D D F A G V V R D D V T L F I S A V Q D Q V V
bleo
2,250 2,260 2,270 2,280 2,290 2,300 2,310 2,320 2,330 2,340 2,350

FseI
GCCGGACAACACCCTGGCCTGGGTGTGGGTGCGCGGCCTGGACGAGCTGTACGCCGAGTGGTCGGAGGTCGTGTCCACGAACTTCCGGGACGCCTCCGGGCCGGCCA
CGGCCTGTTGTGGGACCGGACCCACACCCACGCGCCGGACCTGCTCGACATGCGGCTCACCAGCCTCCAGCACAGGTGCTTGAAGGCCCTGCGGAGGCCCGGCCGGT
P D N T L A W V W V R G L D E L Y A E W S E V V S T N F R D A S G P A
bleo
2,360 2,370 2,380 2,390 2,400 2,410 2,420 2,430 2,440 2,450 2,460

SalI
Tth111I
DraIII PflFI
TGACCGAGATCGGCGAGCAGCCGTGGGGGCGGGAGTTCGCCCTGCGCGACCCGGCCGGCAACTGCGTGCACTTCGTGGCCGAGGAGCAGGACTGAGTCGACTCGACA
ACTGGCTCTAGCCGCTCGTCGGCACCCCCGCCCTCAAGCGGGACGCGCTGGGCCGGCCGTTGACGCACGTGAAGCACCGGCTCCTCGTCCTGACTCAGCTGAGCTGT
M T E I G E Q P W G R E F A L R D P A G N C V H F V A E E Q D *
bleo
2,470 2,480 2,490 2,500 2,510 2,520 2,530 2,540 2,550 2,560

ATCAACCTCTGGATTACAAAATTTGTGAAAGATTGACTGGTATTCTTAACTATGTTGCTCCTTTTACGCTATGTGGATACGCTGCTTTAATGCCTTTGTATCATGCT
TAGTTGGAGACCTAATGTTTTAAACACTTTCTAACTGACCATAAGAATTGATACAACGAGGAAAATGCGATACACCTATGCGACGAAATTACGGAAACATAGTACGA
WPRE
2,570 2,580 2,590 2,600 2,610 2,620 2,630 2,640 2,650 2,660 2,670

ATTGCTTCCCGTATGGCTTTCATTTTCTCCTCCTTGTATAAATCCTGGTTGCTGTCTCTTTATGAGGAGTTGTGGCCCGTTGTCAGGCAACGTGGCGTGGTGTGCAC
TAACGAAGGGCATACCGAAAGTAAAAGAGGAGGAACATATTTAGGACCAACGACAGAGAAATACTCCTCAACACCGGGCAACAGTCCGTTGCACCGCACCACACGTG

WPRE

2,680 2,690 2,700 2,710 2,720 2,730 2,740 2,750 2,760 2,770 2,780

TGTGTTTGCTGACGCAACCCCCACTGGTTGGGGCATTGCCACCACCTGTCAGCTCCTTTCCGGGACTTTCGCTTTCCCCCTCCCTATTGCCACGGCGGAACTCATCG
ACACAAACGACTGCGTTGGGGGTGACCAACCCCGTAACGGTGGTGGACAGTCGAGGAAAGGCCCTGAAAGCGAAAGGGGGAGGGATAACGGTGCCGCCTTGAGTAGC

WPRE

2,790 2,800 2,810 2,820 2,830 2,840 2,850 2,860 2,870 2,880

CCGCCTGCCTTGCCCGCTGCTGGACAGGGGCTCGGCTGTTGGGCACTGACAATTCCGTGGTGTTGTCGGGGAAATCATCGTCCTTTCCTTGGCTGCTCGCCTGTGTT
GGCGGACGGAACGGGCGACGACCTGTCCCCGAGCCGACAACCCGTGACTGTTAAGGCACCACAACAGCCCCTTTAGTAGCAGGAAAGGAACCGACGAGCGGACACAA

WPRE

2,890 2,900 2,910 2,920 2,930 2,940 2,950 2,960 2,970 2,980 2,990

GCCACCTGGATTCTGCGCGGGACGTCCTTCTGCTACGTCCCTTCGGCCCTCAATCCAGCGGACCTTCCTTCCCGCGGCCTGCTGCCGGCTCTGCGGCCTCTTCCGCG
CGGTGGACCTAAGACGCGCCCTGCAGGAAGACGATGCAGGGAAGCCGGGAGTTAGGTCGCCTGGAAGGAAGGGCGCCGGACGACGGCCGAGACGCCGGAGAAGGCGC

WPRE

3,000 3,010 3,020 3,030 3,040 3,050 3,060 3,070 3,080 3,090 3,100

KpnI
Acc65I

TCTTCGCCTTCGCCCTCAGACGAGTCGGATCTCCCTTTGGGCCGCCTCCCCGCCTGGTACCTTTAAGACCAATGACTTACAAGGCAGCTGTAGATCTTAGCCACTTT
AGAAGCGGAAGCGGGAGTCTGCTCAGCCTAGAGGGAAACCCGGCGGAGGGGCGGACCATGGAAATTCTGGTTACTGAATGTTCCGTCGACATCTAGAATCGGTGAAA

WPRE

3,110 3,120 3,130 3,140 3,150 3,160 3,170 3,180 3,190 3,200 3,210

TTAAAAGAAAAGGGGGGACTGGAAGGGCTAATTCACTCCCAACGAAGATAAGATCTGCTTTTTGCTTGTACTGGGTCTCTCTGGTTAGACCAGATCTGAGCCTGGGA
AATTTTCTTTTCCCCCCTGACCTTCCCGATTAAGTGAGGGTTGCTTCTATTCTAGACGAAAAACGAACATGACCCAGAGAGACCAATCTGGTCTAGACTCGGACCCT

3’LTR

3,220 3,230 3,240 3,250 3,260 3,270 3,280 3,290 3,300 3,310

GCTCTCTGGCTAACTAGGGAACCCACTGCTTAAGCCTCAATAAAGCTTGCCTTGAGTGCTTCAAGTAGTGTGTGCCCGTCTGTTGTGTGACTCTGGTAACTAGAGAT
CGAGAGACCGATTGATCCCTTGGGTGACGAATTCGGAGTTATTTCGAACGGAACTCACGAAGTTCATCACACACGGGCAGACAACACACTGAGACCATTGATCTCTA

3’LTR

3,320 3,330 3,340 3,350 3,360 3,370 3,380 3,390 3,400 3,410 3,420

CCCTCAGACCCTTTTAGTCAGTGTGGAAAATCTCTAGCAGTAGTAGTTCATGTCATCTTATTATTCAGTATTTATAACTTGCAAAGAAATGAATATCAGAGAGTGAG
GGGAGTCTGGGAAAATCAGTCACACCTTTTAGAGATCGTCATCATCAAGTACAGTAGAATAATAAGTCATAAATATTGAACGTTTCTTTACTTATAGTCTCTCACTC

3’LTR

3,430 3,440 3,450 3,460 3,470 3,480 3,490 3,500 3,510 3,520 3,530

bArr1-HA-TEV219 pCDH-CuO-MCS-EF1α-CymR-T2A-Bleo3 SparQ (10934 bp)

AGGAACTTGTTTATTGCAGCTTATAATGGTTACAAATAAAGCAATAGCATCACAAATTTCACAAATAAAGCATTTTTTTCACTGCATTCTAGTTGTGGTTTGTCCAA
TCCTTGAACAAATAACGTCGAATATTACCAATGTTTATTTCGTTATCGTAGTGTTTAAAGTGTTTATTTCGTAAAAAAAGTGACGTAAGATCAACACCAAACAGGTT

SV40 Polyadenylation signal

3,540 3,550 3,560 3,570 3,580 3,590 3,600 3,610 3,620 3,630

ACTCATCAATGTATCTTATCATGTCTGGCTCTAGCTATCCCGCCCCTAACTCCGCCCATCCCGCCCCTAACTCCGCCCAGTTCCGCCCATTCTCCGCCCCATGGCTG
TGAGTAGTTACATAGAATAGTACAGACCGAGATCGATAGGGCGGGGATTGAGGCGGGTAGGGCGGGGATTGAGGCGGGTCAAGGCGGGTAAGAGGCGGGGTACCGAC

SV40 Polyad...ion signal SV40 Ori

3,640 3,650 3,660 3,670 3,680 3,690 3,700 3,710 3,720 3,730 3,740

SfiI

ACTAATTTTTTTTATTTATGCAGAGGCCGAGGCCGCCTCGGCCTCTGAGCTATTCCAGAAGTAGTGAGGAGGCTTTTTTGGAGGCCTAGACTTTTGCAGAGACCAAA
TGATTAAAAAAAATAAATACGTCTCCGGCTCCGGCGGAGCCGGAGACTCGATAAGGTCTTCATCACTCCTCCGAAAAAACCTCCGGATCTGAAAACGTCTCTGGTTT

SV40 Ori

3,750 3,760 3,770 3,780 3,790 3,800 3,810 3,820 3,830 3,840 3,850

TTCGTAATCATGTCATAGCTGTTTCCTGTGTGAAATTGTTATCCGCTCACAATTCCACACAACATACGAGCCGGAAGCATAAAGTGTAAAGCCTGGGGTGCCTAATG
AAGCATTAGTACAGTATCGACAAAGGACACACTTTAACAATAGGCGAGTGTTAAGGTGTGTTGTATGCTCGGCCTTCGTATTTCACATTTCGGACCCCACGGATTAC

3,860 3,870 3,880 3,890 3,900 3,910 3,920 3,930 3,940 3,950

AGTGAGCTAACTCACATTAATTGCGTTGCGCTCACTGCCCGCTTTCCAGTCGGGAAACCTGTCGTGCCAGCTGCATTAATGAATCGGCCAACGCGCGGGGAGAGGCG
TCACTCGATTGAGTGTAATTAACGCAACGCGAGTGACGGGCGAAAGGTCAGCCCTTTGGACAGCACGGTCGACGTAATTACTTAGCCGGTTGCGCGCCCCTCTCCGC

3,960 3,970 3,980 3,990 4,000 4,010 4,020 4,030 4,040 4,050 4,060

BspQI
SapI

GTTTGCGTATTGGGCGCTCTTCCGCTTCCTCGCTCACTGACTCGCTGCGCTCGGTCGTTCGGCTGCGGCGAGCGGTATCAGCTCACTCAAAGGCGGTAATACGGTTA
CAAACGCATAACCCGCGAGAAGGCGAAGGAGCGAGTGACTGAGCGACGCGAGCCAGCAAGCCGACGCCGCTCGCCATAGTCGAGTGAGTTTCCGCCATTATGCCAAT

4,070 4,080 4,090 4,100 4,110 4,120 4,130 4,140 4,150 4,160 4,170

AflIII
PciI

TCCACAGAATCAGGGGATAACGCAGGAAAGAACATGTGAGCAAAAGGCCAGCAAAAGGCCAGGAACCGTAAAAAGGCCGCGTTGCTGGCGTTTTTCCATAGGCTCCG
AGGTGTCTTAGTCCCCTATTGCGTCCTTTCTTGTACACTCGTTTTCCGGTCGTTTTCCGGTCCTTGGCATTTTTCCGGCGCAACGACCGCAAAAAGGTATCCGAGGC

pUC ori

4,180 4,190 4,200 4,210 4,220 4,230 4,240 4,250 4,260 4,270 4,280

CCCCCCTGACGAGCATCACAAAAATCGACGCTCAAGTCAGAGGTGGCGAAACCCGACAGGACTATAAAGATACCAGGCGTTTCCCCCTGGAAGCTCCCTCGTGCGCT
GGGGGGACTGCTCGTAGTGTTTTTAGCTGCGAGTTCAGTCTCCACCGCTTTGGGCTGTCCTGATATTTCTATGGTCCGCAAAGGGGGACCTTCGAGGGAGCACGCGA

pUC ori

4,290 4,300 4,310 4,320 4,330 4,340 4,350 4,360 4,370 4,380

CTCCTGTTCCGACCCTGCCGCTTACCGGATACCTGTCCGCCTTTCTCCCTTCGGGAAGCGTGGCGCTTTCTCATAGCTCACGCTGTAGGTATCTCAGTTCGGTGTAG
GAGGACAAGGCTGGGACGGCGAATGGCCTATGGACAGGCGGAAAGAGGGAAGCCCTTCGCACCGCGAAAGAGTATCGAGTGCGACATCCATAGAGTCAAGCCACATC
pUC ori
4,390 4,400 4,410 4,420 4,430 4,440 4,450 4,460 4,470 4,480 4,490

GTCGTTCGCTCCAAGCTGGGCTGTGTGCACGAACCCCCCGTTCAGCCCGACCGCTGCGCCTTATCCGGTAACTATCGTCTTGAGTCCAACCCGGTAAGACACGACTT
CAGCAAGCGAGGTTCGACCCGACACACGTGCTTGGGGGGCAAGTCGGGCTGGCGACGCGGAATAGGCCATTGATAGCAGAACTCAGGTTGGGCCATTCTGTGCTGAA
pUC ori
4,500 4,510 4,520 4,530 4,540 4,550 4,560 4,570 4,580 4,590 4,600

ATCGCCACTGGCAGCAGCCACTGGTAACAGGATTAGCAGAGCGAGGTATGTAGGCGGTGCTACAGAGTTCTTGAAGTGGTGGCCTAACTACGGCTACACTAGAAGGA
TAGCGGTGACCGTCGTCGGTGACCATTGTCCTAATCGTCTCGCTCCATACATCCGCCACGATGTCTCAAGAACTTCACCACCGGATTGATGCCGATGTGATCTTCCT
pUC ori
4,610 4,620 4,630 4,640 4,650 4,660 4,670 4,680 4,690 4,700

CAGTATTTGGTATCTGCGCTCTGCTGAAGCCAGTTACCTTCGGAAAAAGAGTTGGTAGCTCTTGATCCGGCAAACAAACCACCGCTGGTAGCGGTGGTTTTTTTGTT
GTCATAAACCATAGACGCGAGACGACTTCGGTCAATGGAAGCCTTTTTCTCAACCATCGAGAACTAGGCCGTTTGTTTGGTGGCGACCATCGCCACCAAAAAAACAA
pUC ori
4,710 4,720 4,730 4,740 4,750 4,760 4,770 4,780 4,790 4,800 4,810

TGCAAGCAGCAGATTACGCGCAGAAAAAAAGGATCTCAAGAAGATCCTTTGATCTTTTCTACGGGGTCTGACGCTCAGTGGAACGAAAACTCACGTTAAGGGATTTT
ACGTTCGTCGTCTAATGCGCGTCTTTTTTTCCTAGAGTTCTTCTAGGAAACTAGAAAAGATGCCCCAGACTGCGAGTCACCTTGCTTTTGAGTGCAATTCCCTAAAA
pUC ori
4,820 4,830 4,840 4,850 4,860 4,870 4,880 4,890 4,900 4,910 4,920

GGTCATGAGATTATCAAAAAGGATCTTCACCTAGATCCTTTTAAATTAAAAATGAAGTTTTAAATCAATCTAAAGTATATATGAGTAAACTTGGTCTGACAGTTACC
CCAGTACTCTAATAGTTTTTCCTAGAAGTGGATCTAGGAAAATTTAATTTTTACTTCAAAATTTAGTTAGATTTCATATATACTCATTTGAACCAGACTGTCAATGG
4,930 4,940 4,950 4,960 4,970 4,980 4,990 5,000 5,010 5,020

AhdI
AATGCTTAATCAGTGAGGCACCTATCTCAGCGATCTGTCTATTTCGTTCATCCATAGTTGCCTGACTCCCCGTCGTGTAGATAACTACGATACGGGAGGGCTTACCA
TTACGAATTAGTCACTCCGTGGATAGAGTCGCTAGACAGATAAAGCAAGTAGGTATCAACGGACTGAGGGGCAGCACATCTATTGATGCTATGCCCTCCCGAATGGT
AmpR
5,030 5,040 5,050 5,060 5,070 5,080 5,090 5,100 5,110 5,120 5,130

TCTGGCCCCAGTGCTGCAATGATACCGCGAGACCCACGCTCACCGGCTCCAGATTTATCAGCAATAAACCAGCCAGCCGGAAGGGCCGAGCGCAGAAGTGGTCCTGC
AGACCGGGGTCACGACGTTACTATGGCGCTCTGGGTGCGAGTGGCCGAGGTCTAAATAGTCGTTATTTGGTCGGTCGGCCTTCCCGGCTCGCGTCTTCACCAGGACG
AmpR
5,140 5,150 5,160 5,170 5,180 5,190 5,200 5,210 5,220 5,230 5,240

bArr1-HA-TEV219 pCDH-CuO-MCS-EF1α-CymR-T2A-Bleo3 SparQ (10934 bp)

FspI
AACTTTATCCGCCTCCATCCAGTCTATTAATTGTTGCCGGGAAGCTAGAGTAAGTAGTTCGCCAGTTAATAGTTTGCGCAACGTTGTTGCCATTGCTACAGGCATCG
TTGAAATAGGCGGAGGTAGGTCAGATAATTAACAACGGCCCTTCGATCTCATTCATCAAGCGGTCAATTATCAAACGCGTTGCAACAACGGTAACGATGTCCGTAGC
AmpR
5,250 5,260 5,270 5,280 5,290 5,300 5,310 5,320 5,330 5,340 5,350

TGGTGTCACGCTCGTCGTTTGGTATGGCTTCATTCAGCTCCGGTTCCCAACGATCAAGGCGAGTTACATGATCCCCCATGTTGTGCAAAAAAGCGGTTAGCTCCTTC
ACCACAGTGCGAGCAGCAAACCATACCGAAGTAAGTCGAGGCCAAGGGTTGCTAGTTCCGCTCAATGTACTAGGGGGTACAACACGTTTTTTCGCCAATCGAGGAAG
AmpR
5,360 5,370 5,380 5,390 5,400 5,410 5,420 5,430 5,440 5,450

GGTCCTCCGATCGTTGTCAGAAGTAAGTTGGCCGCAGTGTTATCACTCATGGTTATGGCAGCACTGCATAATTCTCTTACTGTCATGCCATCCGTAAGATGCTTTTC
CCAGGAGGCTAGCAACAGTCTTCATTCAACCGGCGTCACAATAGTGAGTACCAATACCGTCGTGACGTATTAAGAGAATGACAGTACGGTAGGCATTCTACGAAAAG
AmpR
5,460 5,470 5,480 5,490 5,500 5,510 5,520 5,530 5,540 5,550 5,560

BcgI
TGTGACTGGTGAGTACTCAACCAAGTCATTCTGAGAATAGTGTATGCGGCGACCGAGTTGCTCTTGCCCGGCGTCAATACGGGATAATACCGCGCCACATAGCAGAA
ACACTGACCACTCATGAGTTGGTTCAGTAAGACTCTTATCACATACGCCGCTGGCTCAACGAGAACGGGCCGCAGTTATGCCCTATTATGGCGCGGTGTATCGTCTT
AmpR
5,570 5,580 5,590 5,600 5,610 5,620 5,630 5,640 5,650 5,660 5,670

XmnI
CTTTAAAAGTGCTCATCATTGGAAAACGTTCTTCGGGGCGAAAACTCTCAAGGATCTTACCGCTGTTGAGATCCAGTTCGATGTAACCCACTCGTGCACCCAACTGA
GAAATTTTCACGAGTAGTAACCTTTTGCAAGAAGCCCCGCTTTTGAGAGTTCCTAGAATGGCGACAACTCTAGGTCAAGCTACATTGGGTGAGCACGTGGGTTGACT
AmpR
5,680 5,690 5,700 5,710 5,720 5,730 5,740 5,750 5,760 5,770

TCTTCAGCATCTTTTACTTTCACCAGCGTTTCTGGGTGAGCAAAAACAGGAAGGCAAAATGCCGCAAAAAAGGGAATAAGGGCGACACGGAAATGTTGAATACTCAT
AGAAGTCGTAGAAAATGAAAGTGGTCGCAAAGACCCACTCGTTTTTGTCCTTCCGTTTTACGGCGTTTTTTCCCTTATTCCCGCTGTGCCTTTACAACTTATGAGTA
AmpR
5,780 5,790 5,800 5,810 5,820 5,830 5,840 5,850 5,860 5,870 5,880

SspI
ACTCTTCCTTTTTCAATATTATTGAAGCATTTATCAGGGTTATTGTCTCATGAGCGGATACATATTTGAATGTATTTAGAAAAATAAACAAATAGGGGTTCCGCGCA
TGAGAAGGAAAAAGTTATAATAACTTCGTAAATAGTCCCAATAACAGAGTACTCGCCTATGTATAAACTTACATAAATCTTTTTATTTGTTTATCCCCAAGGCGCGT
5,890 5,900 5,910 5,920 5,930 5,940 5,950 5,960 5,970 5,980 5,990

CATTTCCCCGAAAAGTGCCACCTGACGTCTAAGAAACCATTATTATCATGACATTAACCTATAAAAATAGGCGTATCACGAGGCCCTTTCGTCTCGCGCGTTTCGGT
GTAAAGGGGCTTTTCACGGTGGACTGCAGATTCTTTGGTAATAATAGTACTGTAATTGGATATTTTTATCCGCATAGTGCTCCGGGAAAGCAGAGCGCGCAAAGCCA
6,000 6,010 6,020 6,030 6,040 6,050 6,060 6,070 6,080 6,090

GATGACGGTGAAAACCTCTGACACATGCAGCTCCCGGAGACGGTCACAGCTTGTCTGTAAGCGGATGCCGGGAGCAGACAAGCCCGTCAGGGCGCGTCAGCGGGTGT
CTACTGCCACTTTTGGAGACTGTGTACGTCGAGGGCCTCTGCCAGTGTCGAACAGACATTCGCCTACGGCCCTCGTCTGTTCGGGCAGTCCCGCGCAGTCGCCCACA

6,100 6,110 6,120 6,130 6,140 6,150 6,160 6,170 6,180 6,190 6,200

NdeI

TGGCGGGTGTCGGGGCTGGCTTAACTATGCGGCATCAGAGCAGATTGTACTGAGAGTGCACCATATGCGGTGTGAAATACCGCACAGATGCGTAAGGAGAAAATACC
ACCGCCCACAGCCCCGACCGAATTGATACGCCGTAGTCTCGTCTAACATGACTCTCACGTGGTATACGCCACACTTTATGGCGTGTCTACGCATTCCTCTTTTATGG

6,210 6,220 6,230 6,240 6,250 6,260 6,270 6,280 6,290 6,300 6,310

FspI

GCATCAGGCGCCATTCGCCATTCAGGCTGCGCAACTGTTGGGAAGGGCGATCGGTGCGGGCCTCTTCGCTATTACGCCAGCTGGCGAAAGGGGGATGTGCTGCAAGG
CGTAGTCCGCGGTAAGCGGTAAGTCCGACGCGTTGACAACCCTTCCCGCTAGCCACGCCCGGAGAAGCGATAATGCGGTCGACCGCTTTCCCCCTACACGACGTTCC

6,320 6,330 6,340 6,350 6,360 6,370 6,380 6,390 6,400 6,410 6,420

AflIII
MluI

CGATTAAGTTGGGTAACGCCAGGGTTTTCCCAGTCACGACGTTGTAAAACGACGGCCAGTGCCAAGCTGACGCGTGTAGTCTTATGCAATACTCTTGTAGTCTTGCA
GCTAATTCAACCCATTGCGGTCCCAAAAGGGTCAGTGCTGCAACATTTTGCTGCCGGTCACGGTTCGACTGCGCACATCAGAATACGTTATGAGAACATCAGAACGT

RSV

6,430 6,440 6,450 6,460 6,470 6,480 6,490 6,500 6,510 6,520

SphI BaeI

ACATGGTAACGATGAGTTAGCAACATGCCTTACAAGGAGAGAAAAAGCACCGTGCATGCCGATTGGTGGAAGTAAGGTGGTACGATCGTGCCTTATTAGGAAGGCAA
TGTACCATTGCTACTCAATCGTTGTACGGAATGTTCCTCTCTTTTTCGTGGCACGTACGGCTAACCACCTTCATTCCACCATGCTAGCACGGAATAATCCTTCCGTT

RSV

6,530 6,540 6,550 6,560 6,570 6,580 6,590 6,600 6,610 6,620 6,630

CAGACGGGTCTGACATGGATTGGACGAACCACTGAATTGCCGCATTGCAGAGATATTGTATTTAAGTGCCTAGCTCGATACATAAACGGGTCTCTCTGGTTAGACCA
GTCTGCCCAGACTGTACCTAACCTGCTTGGTGACTTAACGGCGTAACGTCTCTATAACATAAATTCACGGATCGAGCTATGTATTTGCCCAGAGAGACCAATCTGGT

RSV HIV LTR

6,640 6,650 6,660 6,670 6,680 6,690 6,700 6,710 6,720 6,730 6,740

GATCTGAGCCTGGGAGCTCTCTGGCTAACTAGGGAACCCACTGCTTAAGCCTCAATAAAGCTTGCCTTGAGTGCTTCAAGTAGTGTGTGCCCGTCTGTTGTGTGACT
CTAGACTCGGACCCTCGAGAGACCGATTGATCCCTTGGGTGACGAATTCGGAGTTATTTCGAACGGAACTCACGAAGTTCATCACACACGGGCAGACAACACACTGA

HIV LTR

6,750 6,760 6,770 6,780 6,790 6,800 6,810 6,820 6,830 6,840

CTGGTAACTAGAGATCCCTCAGACCCTTTTAGTCAGTGTGGAAAATCTCTAGCAGTGGCGCCCGAACAGGGACTTGAAAGCGAAAGGGAAACCAGAGGAGCTCTCTC
GACCATTGATCTCTAGGGAGTCTGGGAAAATCAGTCACACCTTTTAGAGATCGTCACCGCGGGCTTGTCCCTGAACTTTCGCTTTCCCTTTGGTCTCCTCGAGAGAG

HIV LTR

6,850 6,860 6,870 6,880 6,890 6,900 6,910 6,920 6,930 6,940 6,950

```
GACGCAGGACTCGGCTTGCTGAAGCGCGCACGGCAAGAGGCGAGGGGCGGCGACTGGTGAGTACGCCAAAAATTTTGACTAGCGGAGGCTAGAAGGAGAGAGATGGG
CTGCGTCCTGAGCCGAACGACTTCGCGCGTGCCGTTCTCCGCTCCCCGCCGCTGACCACTCATGCGGTTTTTAAAACTGATCGCCTCCGATCTTCCTCTCTCTACCC
```

Psi

6,960 6,970 6,980 6,990 7,000 7,010 7,020 7,030 7,040 7,050 7,060

NruI XmnI

```
TGCGAGAGCGTCAGTATTAAGCGGGGGAGAATTAGATCGCGATGGGAAAAAATTCGGTTAAGGCCAGGGGGAAAGAAAAAATATAAATTAAAACATATAGTATGGGC
ACGCTCTCGCAGTCATAATTCGCCCCCTCTTAATCTAGCGCTACCCTTTTTTAAGCCAATTCCGGTCCCCCTTTCTTTTTTATATTTAATTTTGTATATCATACCCG
```

Gag

7,070 7,080 7,090 7,100 7,110 7,120 7,130 7,140 7,150 7,160

```
AAGCAGGGAGCTAGAACGATTCGCAGTTAATCCTGGCCTGTTAGAAACATCAGAAGGCTGTAGACAAATACTGGGACAGCTACAACCATCCCTTCAGACAGGATCAG
TTCGTCCCTCGATCTTGCTAAGCGTCAATTAGGACCGGACAATCTTTGTAGTCTTCCGACATCTGTTTATGACCCTGTCGATGTTGGTAGGGAAGTCTGTCCTAGTC
```

Gag

7,170 7,180 7,190 7,200 7,210 7,220 7,230 7,240 7,250 7,260 7,270

BspQI
SapI

```
AAGAACTTAGATCATTATATAATACAGTAGCAACCCTCTATTGTGTGCATCAAAGGATAGAGATAAAAGACACCAAGGAAGCTTTAGACAAGATAGAGGAAGAGCAA
TTCTTGAATCTAGTAATATATTATGTCATCGTTGGGAGATAACACACGTAGTTTCCTATCTCTATTTTCTGTGGTTCCTTCGAAATCTGTTCTATCTCCTTCTCGTT
```

Gag

7,280 7,290 7,300 7,310 7,320 7,330 7,340 7,350 7,360 7,370 7,380

MfeI

```
AACAAAAGTAAGACCACCGCACAGCAAGCGGCCACTGATCTTCAGACCTGGAGGAGGAGATATGAGGGACAATTGGAGAAGTGAATTATATAAATATAAAGTAGTAA
TTGTTTTCATTCTGGTGGCGTGTCGTTCGCCGGTGACTAGAAGTCTGGACCTCCTCCTCTATACTCCCTGTTAACCTCTTCACTTAATATATTTATATTTCATCATT
```

Gag Env

7,390 7,400 7,410 7,420 7,430 7,440 7,450 7,460 7,470 7,480 7,490

```
AAATTGAACCATTAGGAGTAGCACCCACCAAGGCAAAGAGAAGAGTGGTGCAGAGAGAAAAAAGAGCAGTGGGAATAGGAGCTTTGTTCCTTGGGTTCTTGGGAGCA
TTTAACTTGGTAATCCTCATCGTGGGTGGTTCCGTTTCTCTTCTCACCACGTCTCTCTTTTTTCTCGTCACCCTTATCCTCGAAACAAGGAACCCAAGAACCCTCGT
```

RRE
Env

7,500 7,510 7,520 7,530 7,540 7,550 7,560 7,570 7,580 7,590

BbvCI

```
GCAGGAAGCACTATGGGCGCAGCGTCAATGACGCTGACGGTACAGGCCAGACAATTATTGTCTGGTATAGTGCAGCAGCAGAACAATTTGCTGAGGGCTATTGAGGC
CGTCCTTCGTGATACCCGCGTCGCAGTTACTGCGACTGCCATGTCCGGTCTGTTAATAACAGACCATATCACGTCGTCGTCTTGTTAAACGACTCCCGATAACTCCG
```

RRE
Env

7,600 7,610 7,620 7,630 7,640 7,650 7,660 7,670 7,680 7,690 7,700

GCAACAGCATCTGTTGCAACTCACAGTCTGGGGCATCAAGCAGCTCCAGGCAAGAATCCTGGCTGTGGAAAGATACCTAAAGGATCAACAGCTCCTGGGGATTTGGG
CGTTGTCGTAGACAACGTTGAGTGTCAGACCCCGTAGTTCGTCGAGGTCCGTTCTTAGGACCGACACCTTTCTATGGATTTCCTAGTTGTCGAGGACCCCTAAACCC
RRE
Env
7,710 7,720 7,730 7,740 7,750 7,760 7,770 7,780 7,790 7,800 7,810

GTTGCTCTGGAAAACTCATTTGCACCACTGCTGTGCCTTGGAATGCTAGTTGGAGTAATAAATCTCTGGAACAGATTTGGAATCACACGACCTGGATGGAGTGGGAC
CAACGAGACCTTTTGAGTAAACGTGGTGACGACACGGAACCTTACGATCAACCTCATTATTTAGAGACCTTGTCTAAACCTTAGTGTGCTGGACCTACCTCACCCTG
RRE
Env
7,820 7,830 7,840 7,850 7,860 7,870 7,880 7,890 7,900 7,910

AGAGAAATTAACAATTACACAAGCTTAATACACTCCTTAATTGAAGAATCGCAAACCAGCAAGAAAAGAATGAACAAGAATTATTGGAATTAGATAAATGGGCAAGT
TCTCTTTAATTGTTAATGTGTTCGAATTATGTGAGGAATTAACTTCTTAGCGTTTGGTCGTTCTTTTCTTACTTGTTCTTAATAACCTTAATCTATTTACCCGTTCA
Env
7,920 7,930 7,940 7,950 7,960 7,970 7,980 7,990 8,000 8,010 8,020

TTGTGGAATTGGTTTAACATAACAAATTGGCTGTGGTATATAAAATTATTCATAATGATAGTAGGAGGCTTGGTAGGTTTAAGAATAGTTTTTGCTGTACTTTCTAT
AACACCTTAACCAAATTGTATTGTTTAACCGACACCATATATTTTAATAAGTATTACTATCATCCTCCGAACCATCCAAATTCTTATCAAAAACGACATGAAAGATA
Env
8,030 8,040 8,050 8,060 8,070 8,080 8,090 8,100 8,110 8,120 8,130

KflI
AGTGAATAGAGTTAGGCAGGGATATTCACCATTATCGTTTCAGACCCACCTCCCAACCCCGAGGGGACCCGACAGGCCCGAAGGAATAGAAGAAGAAGGTGGAGAGA
TCACTTATCTCAATCCGTCCCTATAAGTGGTAATAGCAAAGTCTGGGTGGAGGGTTGGGGCTCCCCTGGGCTGTCCGGGCTTCCTTATCTTCTTCTTCCACCTCTCT
Env
8,140 8,150 8,160 8,170 8,180 8,190 8,200 8,210 8,220 8,230

HpaI
GAGACAGAGACAGATCCATTCGATTAGTGAACGGATCTCGACGGTATCGGTTAACTTTTAAAAGAAAAGGGGGGATTGGGGGGTACAGTGCAGGGGAAAGAATAGTA
CTCTGTCTCTGTCTAGGTAAGCTAATCACTTGCCTAGAGCTGCCATAGCCAATTGAAAATTTTCTTTTCCCCCCTAACCCCCCATGTCACGTCCCCTTTCTTATCAT
Env
cPPT
8,240 8,250 8,260 8,270 8,280 8,290 8,300 8,310 8,320 8,330 8,340

BspDI
ClaI
SpeI
GACATAATAGCAACAGACATACAAACTAAAGAATTACAAAAACAAATTACAAAATTCAAAATTTTATCGATAGACTAGTTATTAATAGTAATCAATTACGGGGTCAT
CTGTATTATCGTTGTCTGTATGTTTGATTTCTTAATGTTTTTGTTTAATGTTTTAAGTTTTAAAATAGCTATCTGATCAATAATTATCATTAGTTAATGCCCCAGTA
cPPT
CMV + CUO
8,350 8,360 8,370 8,380 8,390 8,400 8,410 8,420 8,430 8,440 8,450

bArr1-HA-TEV219 pCDH-CuO-MCS-EF1α-CymR-T2A-Bleo3 SparQ (10934 bp)

TAGTTCATAGCCCATATATGGAGTTCCGCGTTACATAACTTACGGTAAATGGCCCGCCTGGCTGACCGCCCAACGACCCCCGCCCATTGACGTCAATAATGACGTAT
ATCAAGTATCGGGTATATACCTCAAGGCGCAATGTATTGAATGCCATTTACCGGGCGGACCGACTGGCGGGTTGCTGGGGGCGGGTAACTGCAGTTATTACTGCATA
CMV + CUO
8,460 8,470 8,480 8,490 8,500 8,510 8,520 8,530 8,540 8,550 8,560

NdeI
GTTCCCATAGTAACGCCAATAGGGACTTTCCATTGACGTCAATGGGTGGAGTATTTACGGTAAACTGCCCACTTGGCAGTACATCAAGTGTATCATATGCCAAGTCC
CAAGGGTATCATTGCGGTTATCCCTGAAAGGTAACTGCAGTTACCCACCTCATAAATGCCATTTGACGGGTGAACCGTCATGTAGTTCACATAGTATACGGTTCAGG
CMV + CUO
8,570 8,580 8,590 8,600 8,610 8,620 8,630 8,640 8,650 8,660

SnaBI
GCCCCCTATTGACGTCAATGACGGTAAATGGCCCGCCTGGCATTATGCCCAGTACATGACCTTACGGGACTTTCCTACTTGGCAGTACATCTACGTATTAGTCATCG
CGGGGGATAACTGCAGTTACTGCCATTTACCGGGCGGACCGTAATACGGGTCATGTACTGGAATGCCCTGAAAGGATGAACCGTCATGTAGATGCATAATCAGTAGC
CMV + CUO
8,670 8,680 8,690 8,700 8,710 8,720 8,730 8,740 8,750 8,760 8,770

CspCI
CTATTACCATGGTGATGCGGTTTTGGCAGTACACCAATGGGCGTGGATAGCGGTTTGACTCACGGGGATTTCCAAGTCTCCACCCCATTGACGTCAATGGGAGTTTG
GATAATGGTACCACTACGCCAAAACCGTCATGTGGTTACCCGCACCTATCGCCAAACTGAGTGCCCCTAAAGGTTCAGAGGTGGGGTAACTGCAGTTACCCTCAAAC
CMV + CUO
8,780 8,790 8,800 8,810 8,820 8,830 8,840 8,850 8,860 8,870 8,880

TTTTGGCACCAAAATCAACGGGACTTTCCAAAATGTCGTAATACCCCGCCCCGTTGACGCAAATGGGCAAGCTTGCCGGGTCGAGGTAGGCGTGTACGGTGGGAGG
AAAACCGTGGTTTTAGTTGCCCTGAAAGGTTTTACAGCATTATGGGGGCGGGGCAACTGCGTTTACCCGTTCGAACGGCCCAGCTCCATCCGCACATGCCACCCTCC
CMV + CUO CMV + CUO
8,890 8,900 8,910 8,920 8,930 8,940 8,950 8,960 8,970 8,980

AgeI AgeI AscI
CCTATATAAGCAACCGGTATAATCAAACAGACCAGATTGTCTGTTTGTTACCGGTGTTTAGTGAACCGGGCGCGCCTCATATCGCCTGGAGACGCCATCCACGCTGT
GGATATATTCGTTGGCCATATTAGTTTGTCTGGTCTAACAGACAAACAATGGCCACAAATCACTTGGCCCGCGCGGAGTATAGCGGACCTCTGCGGTAGGTGCGACA
CMV + CUO
8,990 9,000 9,010 9,020 9,030 9,040 9,050 9,060 9,070 9,080 9,090

TTTGACCTCCATAGAAGACACCGGGACCGATCCAGCCTCCGCGGTCACTCTCTTCCGCATCGCTGTCTGCGAGGGCCAGCTGTTGGGCTCGCGGTTGAGGACAAACT
AAACTGGAGGTATCTTCTGTGGCCCTGGCTAGGTCGGAGGCGCCAGTGAGAGAAGGCGTAGCGACAGACGCTCCCGGTCGACAACCCGAGCGCCAACTCCTGTTTGA
CMV + CUO
9,100 9,110 9,120 9,130 9,140 9,150 9,160 9,170 9,180 9,190 9,200

CTTCGCGGTCTTTCCAGTACTCTTGGATCGGAAACCCGTCGGCCTCCGAACGGTACTCCGCCACCGAGGGACCTGAGCGAGTCCGCATCGACCGGATCGGAAAACCT
GAAGCGCCAGAAAGGTCATGAGAACCTAGCCTTTGGGCAGCCGGAGGCTTGCCATGAGGCGGTGGCTCCCTGGACTCGCTCAGGCGTAGCTGGCCTAGCCTTTTGGA

CMV + CUO CMV + CUO

9,210 9,220 9,230 9,240 9,250 9,260 9,270 9,280 9,290 9,300

PaeR7I
XhoI
TliI
BlpI

CTCGAGAAAGGCGTCTAACCAGTCACAGTCGCAAGGTAGGCTGAGCACCGTGGCGGGCGGCAGCGGGTGGCGGTCGGGGTTGTTTCTGGCGGAGGTGCTGCTGATGA
GAGCTCTTTCCGCAGATTGGTCAGTGTCAGCGTTCCATCCGACTCGTGGCACCGCCCGCCGTCGCCCACCGCCAGCCCCAACAAAGACCGCCTCCACGACGACTACT

CMV + CUO

9,310 9,320 9,330 9,340 9,350 9,360 9,370 9,380 9,390 9,400 9,410

TGTAATTAAAGTAGGCGGTCTTGAGACGGCGGATGGTCGAGGTGAGGTGTGGCAGGCTTGAGATCCAGCTGTTGGGGTGAGTACTCCCTCTCAAAAGCGGGCATTAC
ACATTAATTTCATCCGCCAGAACTCTGCCGCCTACCAGCTCCACTCCACACCGTCCGAACTCTAGGTCGACAACCCCACTCATGAGGGAGAGTTTTCGCCCGTAATG

CMV + CUO CMV + CUO

9,420 9,430 9,440 9,450 9,460 9,470 9,480 9,490 9,500 9,510 9,520

TTCTGCGCTAAGATTGTCAGTTTCCAAAAACGAGGAGGATTTGATATTCACCTGGCCCGATCTGGCCATACACTTGAGTGACAATGACATCCACTTTGCCTTTCTCT
AAGACGCGATTCTAACAGTCAAAGGTTTTTGCTCCTCCTAAACTATAAGTGGACCGGGCTAGACCGGTATGTGAACTCACTGTTACTGTAGGTGAAACGGAAAGAGA

CMV + CUO

9,530 9,540 9,550 9,560 9,570 9,580 9,590 9,600 9,610 9,620 9,630

BmtI
XbaI NheI

CCACAGGTGTCCACTCCCAGGTCCAAGTTTGGTCTAGAGCTAGCGCCACCATGGGCGACAAAGGCACGAGAGTGTTCAAGAAGGCCTCTCCTAACGGCAAGCTGACC
GGTGTCCACAGGTGAGGGTCCAGGTTCAAACCAGATCTCGATCGCGGTGGTACCCGCTGTTTCCGTGCTCTCACAAGTTCTTCCGGAGAGGATTGCCGTTCGACTGG

M G D K G T R V F K K A S P N G K L T

CMV + CUO Arrb1 optimized

9,640 9,650 9,660 9,670 9,680 9,690 9,700 9,710 9,720 9,730

BspDI
ClaI

GTGTACCTCGGCAAGAGGGACTTCGTGGACCACATCGATCTGGTGGACCCTGTGGATGGCGTGGTGCTGGTCGATCCCGAGTACCTGAAAGAACGGCGGGTGTACGT
CACATGGAGCCGTTCTCCCTGAAGCACCTGGTGTAGCTAGACCACCTGGGACACCTACCGCACCACGACCAGCTAGGGCTCATGGACTTTCTTGCCGCCCACATGCA

V Y L G K R D F V D H I D L V D P V D G V V L V D P E Y L K E R R V Y V

Arrb1 optimized

9,740 9,750 9,760 9,770 9,780 9,790 9,800 9,810 9,820 9,830 9,840

GACCCTGACCTGCGCCTTTAGATACGGCCGCGAGGATCTGGATGTGCTGGGCCTGACCTTCAGAAAGGACCTGTTCGTGGCCAACGTGCAGAGCTTCCCACCAGCTC
CTGGGACTGGACGCGGAAATCTATGCCGGCGCTCCTAGACCTACACGACCCGGACTGGAAGTCTTTCCTGGACAAGCACCGGTTGCACGTCTCGAAGGGTGGTCGAG
T L T C A F R Y G R E D L D V L G L T F R K D L F V A N V Q S F P P A

Arrb1 optimized

9,850 9,860 9,870 9,880 9,890 9,900 9,910 9,920 9,930 9,940 9,950

CAGAGGACAAGAAGCCCCTGACCAGACTGCAAGAGCGGCTGATTAAGAAGCTGGGCGAGCACGCTTACCCCTTCACCTTCGAGATCCCTCCAAACCTGCCTTGCAGC
GTCTCCTGTTCTTCGGGGACTGGTCTGACGTTCTCGCCGACTAATTCTTCGACCCGCTCGTGCGAATGGGGAAGTGGAAGCTCTAGGGAGGTTTGGACGGAACGTCG
P E D K K P L T R L Q E R L I K K L G E H A Y P F T F E I P P N L P C S

Arrb1 optimized

9,960 9,970 9,980 9,990 10,000 10,010 10,020 10,030 10,040 10,050

Bsu36I

GTGACACTCCAACCTGGACCTGAGGATACCGGAAAGGCCTGTGGCGTGGACTACGAAGTGAAGGCCTTTTGCGCCGAGAACCTGGAAGAGAAGATCCACAAGCGGAA
CACTGTGAGGTTGGACCTGGACTCCTATGGCCTTTCCGGACACCGCACCTGATGCTTCACTTCCGGAAAACGCGGCTCTTGGACCTTCTCTTCTAGGTGTTCGCCTT
V T L Q P G P E D T G K A C G V D Y E V K A F C A E N L E E K I H K R N

Arrb1 optimized

10,060 10,070 10,080 10,090 10,100 10,110 10,120 10,130 10,140 10,150 10,160

CAGCGTGCGGCTGGTCATCAGAAAGGTGCAGTATGCCCCTGAGAGGCCCGGACCTCAACCTACAGCCGAGACAACCAGACAGTTCCTGATGAGCGACAAGCCCCTGC
GTCGCACGCCGACCAGTAGTCTTTCCACGTCATACGGGGACTCTCCGGGCCTGGAGTTGGATGTCGGCTCTGTTGGTCTGTCAAGGACTACTCGCTGTTCGGGGACG
S V R L V I R K V Q Y A P E R P G P Q P T A E T T R Q F L M S D K P L

Arrb1 optimized

10,170 10,180 10,190 10,200 10,210 10,220 10,230 10,240 10,250 10,260 10,270

PmlI

ACCTGGAAGCCAGCCTGGACAAAGAGATCTACTACCACGGCGAGCCCATCAGCGTGAACGTCCACGTGACCAACAACACCAACAAGACCGTGAAGAAAATCAAGATC
TGGACCTTCGGTCGGACCTGTTTCTCTAGATGATGGTGCCGCTCGGGTAGTCGCACTTGCAGGTGCACTGGTTGTTGTGGTTGTTCTGGCACTTCTTTTAGTTCTAG
H L E A S L D K E I Y Y H G E P I S V N V H V T N N T N K T V K K I K I

Arrb1 optimized

10,280 10,290 10,300 10,310 10,320 10,330 10,340 10,350 10,360 10,370

TCCGTGCGGCAGTACGCCGACATCTGCCTGTTTAACACAGCCCAGTACAAGTGCCCCGTGGCCATGGAAGAGGCCGATGATACAGTGGCCCCTAGCAGCACCTTCTG
AGGCACGCCGTCATGCGGCTGTAGACGGACAAATTGTGTCGGGTCATGTTCACGGGGCACCGGTACCTTCTCCGGCTACTATGTCACCGGGGATCGTCGTGGAAGAC
S V R Q Y A D I C L F N T A Q Y K C P V A M E E A D D T V A P S S T F C

Arrb1 optimized

10,390 10,400 10,410 10,420 10,430 10,440 10,450 10,460 10,470 10,480

bArr1-HA-TEV219 pCDH-CuO-MCS-EF1α-CymR-T2A-Bleo3 SparQ (10934 bp)

BstZ17I

CAAAGTGTATACCCTGACACCTTTCCTGGCCAACAACCGCGAGAAGAGAGGACTGGCCCTGGACGGAAAGCTGAAGCACGAGGATACCAACCTGGCCAGCAGCACCC
GTTTCACATATGGGACTGTGGAAAGGACCGGTTGTTGGCGCTCTTCTCTCCTGACCGGGACCTGCCTTTCGACTTCGTGCTCCTATGGTTGGACCGGTCGTCGTGGG
K V Y T L T P F L A N N R E K R G L A L D G K L K H E D T N L A S S T

Arrb1 optimized

10,490 10,500 10,510 10,520 10,530 10,540 10,550 10,560 10,570 10,580 10,590

TGCTTAGAGAGGGCGCCAATAGAGAGATCCTGGGCATCATCGTGTCCTACAAAGTGAAAGTGAAGCTGGTGGTGTCCAGAGGCGGCCTGCTTGGAGATCTGGCCTCT
ACGAATCTCTCCCGCGGTTATCTCTCTAGGACCCGTAGTAGCACAGGATGTTTCACTTTCACTTCGACCACCACAGGTCTCCGCCGGACGAACCTCTAGACCGGAGA
L L R E G A N R E I L G I I V S Y K V K V K L V V S R G G L L G D L A S

Arrb1 optimized

10,600 10,610 10,620 10,630 10,640 10,650 10,660 10,670 10,680 10,690 10,700

TCTGATGTGGCCGTGGAACTGCCCCTTCACACTGATGCACCCCAAGCCTAAAGAGGAACCTCCTCACAGAGAGGTGCCCGAGAACGAGACACCCGTGGACACCAATCT
AGACTACACCGGCACCTTGACGGGGAAGTGTGACTACGTGGGGTTCGGATTTCTCCTTGGAGGAGTGTCTCTCCACGGGCTCTTGCTCTGTGGGCACCTGTGGTTAGA
S D V A V E L P F T L M H P K P K E E P P H R E V P E N E T P V D T N L

Arrb1 optimized

10,710 10,720 10,730 10,740 10,750 10,760 10,770 10,780 10,790 10,800

GATCGAGCTGGACACAAACGACGACGACATCGTGTTCGAGGACTTCGCCCGGCAGAGACTGAAGGGCATGAAGGACGACAAAGAGGAAGAAGAGGACGGCACCGGCT
CTAGCTCGACCTGTGTTTGCTGCTGCTGTAGCACAAGCTCCTGAAGCGGGCCGTCTCTGACTTCCCGTACTTCCTGCTGTTTCTCCTTCTTCTCCTGCCGTGGCCGA
I E L D T N D D D I V F E D F A R Q R L K G M K D D K E E E E D G T G

Arrb1 optimized

10,810 10,820 10,830 10,840 10,850 10,860 10,870 10,880 10,890 10,900 10,910

CTCCGCAGCTCAATAATCGG
GAGGCGTCGAGTTATTAGCC
S P Q L N N R

Arrb1 optimized

10,920 10,930

(from 1-642 bp)

bArr2-HA-TEV219 pCDH-CuO-MCS-EF1α-CymR-T2A-Bleo...

PspOMI

GGATCCGGTAGTGGTGGTGGGTCTGGGGGAGGGTCCGGAGGCGGCTCTGGTGGTGGGTCATACCCTTACGACGTACCTGATTACGCGGGCTCATCTCTGTTCAAGGG
CCTAGGCCATCACCACCACCCAGACCCCCTCCCAGGCCTCCGCCGAGACCACCACCCAGTATGGGAATGCTGCATGGACTAATGCGCCCGAGTAGAGACAAGTTCCC
G S G S G G G S G G G S G G G S G G G S Y P Y D V P D Y A G S S L F K G

Linker

HA-Tag

Tev 219

10 20 30 40 50 60 70 80 90 100

ApaI

CCCAAGAGACTATAATCCTATCTCATCAACGATCTGCCATCTCACAAACGAATCCGACGGACACACGACCTCTCTTTACGGGATTGGTTTCGGCCCTTTTATCATAA
GGGTTCTCTGATATTAGGATAGAGTAGTTGCTAGACGGTAGAGTGTTTGCTTAGGCTGCCTGTGTGCTGGAGAGAAATGCCCTAACCAAAGCCGGGAAAATAGTATT
P R D Y N P I S S T I C H L T N E S D G H T T S L Y G I G F G P F I I

Tev 219

110 120 130 140 150 160 170 180 190 200 210

XcmI

CTAATAAACATCTTTTCCGCCGGAACAACGGAACTTTGCTTGTCCAGTCACTTCATGGCGTATTTAAAGTGAAGAATACTACTACTTTGCAACAACATCTCATTGAC
GATTATTTGTAGAAAAGGCGGCCTTGTTGCCTTGAAACGAACAGGTCAGTGAAGTACCGCATAAATTTCACTTCTTATGATGATGAAACGTTGTTGTAGAGTAACTG
T N K H L F R R N N G T L L V Q S L H G V F K V K N T T T L Q Q H L I D

Tev 219

220 230 240 250 260 270 280 290 300 310 320

GGCCGGGACATGATTATTATAAGAATGCCGAAGGATTTTCCACCTTTCCCCCAAAAACTTAAGTTTCGGGAGCCACAACGAGAGGAACGAATATGCCTGGTAACAAC
CCGGCCCTGTACTAATAATATTCTTACGGCTTCCTAAAAGGTGGAAAGGGGGTTTTTGAATTCAAAGCCCTCGGTGTTGCTCTCCTTGCTTATACGGACCATTGTTG
G R D M I I I R M P K D F P P F P Q K L K F R E P Q R E E R I C L V T T

Tev 219

330 340 350 360 370 380 390 400 410 420

BsrGI

AAATTTTCAAACTAAATCTATGTCTTCAATGGTGAGTGACACATCTTGTACATTTCCCTCTAGTGACGGGATTTTCTGGAAGCATTGGATACAGACTAAGGACGGGC
TTTAAAAGTTTGATTTAGATACAGAAGTTACCACTCACTGTGTAGAACATGTAAAGGGAGATCACTGCCCTAAAAGACCTTCGTAACCTATGTCTGATTCCTGCCCG
N F Q T K S M S S M V S D T S C T F P S S D G I F W K H W I Q T K D G

Tev 219

430 440 450 460 470 480 490 500 510 520 530

AGTGCGGATCACCACTCGTGTCCACACGCGATGGATTCATTGTCGGGATTCATAGTGCTTCTAACTTCACCAACACCAATAATTATTTTACTTCTGTGCCAAAAAAC
TCACGCCTAGTGGTGAGCACAGGTGTGCGCTACCTAAGTAACAGCCCTAAGTATCACGAAGATTGAAGTGGTTGTGGTTATTAATAAAATGAAGACACGGTTTTTTG
Q C G S P L V S T R D G F I V G I H S A S N F T N T N N Y F T S V P K N

Tev 219

540 550 560 570 580 590 600 610 620 630 640

bArr2-HA-TEV219 pCDH-CuO-MCS-EF1α-CymR-T2A-Bleo3 SparQ (10907 bp)

Tth111I
AloI PflFI

TTCATGGAGCTGCTTACTAGCCAAGAAGCCCAACAGTGGGTGAGTGGGTGGCGCCTGAACGCCGACTCCGTCTTGTGGGGGGGTCACAAGGTCTTTATGGTATAAAT
AAGTACCTCGACGAATGATCGGTTCTTCGGGTTGTCACCCACTCACCCACCGCGGACTTGCGGCTGAGGCAGAACACCCCCCCAGTGTTCCAGAAATACCATATTTA
F M E L L T S Q E A Q Q W V S G W R L N A D S V L W G G H K V F M V *

Tev 219

650 660 670 680 690 700 710 720 730 740

SwaI NotI AsiSI MfeI

TTAAATCGGATCCGCGGCCGCGGATCTGCGATCGCTCCGGTGCCCGTCAGTGGGCAGAGCGCACATCGCCCACAGTCCCCGAGAAGTTGGGGGGAGGGGTCGGCAAT
AATTTAGCCTAGGCGCCGGCGCCTAGACGCTAGCGAGGCCACGGGCAGTCACCCGTCTCGCGTGTAGCGGGTGTCAGGGGCTCTTCAACCCCCCTCCCCAGCCGTTA

EF1 alpha

750 760 770 780 790 800 810 820 830 840 850

TGAACGGGTGCCTAGAGAAGGTGGCGCGGGGTAAACTGGGAAAGTGATGTCGTGTACTGGCTCCGCCTTTTTCCCGAGGGTGGGGGAGAACCGTATATAAGTGCAGT
ACTTGCCCACGGATCTCTTCCACCGCGCCCCATTTGACCCTTTCACTACAGCACATGACCGAGGCGGAAAAAGGGCTCCCACCCCCTCTTGGCATATATTCACGTCA

EF1 alpha

860 870 880 890 900 910 920 930 940 950 960

Bsu36I

AGTCGCCGTGAACGTTCTTTTTCGCAACGGGTTTGCCGCCAGAACACAGCTGAAGCTTCGAGGGGCTCGCATCTCTCCTTCACGCGCCCGCCGCCCTACCTGAGGCC
TCAGCGGCACTTGCAAGAAAAAGCGTTGCCCAAACGGCGGTCTTGTGTCGACTTCGAAGCTCCCCGAGCGTAGAGAGGAAGTGCGCGGGCGGCGGGATGGACTCCGG

EF1 alpha

970 980 990 1,000 1,010 1,020 1,030 1,040 1,050 1,060 1,070

CspCI

GCCATCCACGCCGGTTGAGTCGCGTTCTGCCGCCTCCCGCCTGTGGTGCCTCCTGAACTGCGTCCGCCGTCTAGGTAAGTTTAAAGCTCAGGTCGAGACCGGGCCTT
CGGTAGGTGCGGCCAACTCAGCGCAAGACGGCGGAGGGCGGACACCACGGAGGACTTGACGCAGGCGGCAGATCCATTCAAATTTCGAGTCCAGCTCTGGCCCGGAA

EF1 alpha

1,080 1,090 1,100 1,110 1,120 1,130 1,140 1,150 1,160 1,170

TGTCCGGCGCTCCCTTGGAGCCTACCTAGACTCAGCCGGCTCTCCACGCTTTGCCTGACCCTGCTTGCTCAACTCTACGTCTTTGTTTCGTTTTCTGTTCTGCGCCG
ACAGGCCGCGAGGGAACCTCGGATGGATCTGAGTCGGCCGAGAGGTGCGAAACGGACTGGGACGAACGAGTTGAGATGCAGAAACAAAGCAAAAGACAAGACGCGGC

EF1 alpha

1,180 1,190 1,200 1,210 1,220 1,230 1,240 1,250 1,260 1,270 1,280

XbaI

TTACAGATCCAAGCTGTGACCGGCGCCTACTCTAGAGCCGCCATGGCTTACCCATACGATGTTCCAGATTACGCTAGTTTGGTGAGGCAGCAGAGACCGATGACATC
AATGTCTAGGTTCGACACTGGCCGCGGATGAGATCTCGGCGGTACCGAATGGGTATGCTACAAGGTCTAATGCGATCAAACCACTCCGTCGTCTCTGGCTACTGTAG
M A Y P Y D V P D Y A S L V R Q Q R P M T S

EF1 alpha HA

1,290 1,300 1,310 1,320 1,330 1,340 1,350 1,360 1,370 1,380 1,390

bArr2-HA-TEV219 pCDH-CuO-MCS-EF1α-CymR-T2A-Bleo3 SparQ (10907 bp)

BclI

CACTTTGCCTTTCTCTCCACAGGTGTCCACTCCCAGGTCCAAGTTTGGAAGATCTACCATGCCAAAGAGACCCAGACCCGTGATCATGAGTCCAAAGAGAAGAACAC
GTGAAACGGAAAGAGAGGTGTCCACAGGTGAGGGTCCAGGTTCAAACCTTCTAGATGGTACGGTTTCTCTGGGTCTGGGCACTAGTACTCAGGTTTCTCTTCTTGTG
T L P F S P Q V S T P R S K F G R S T M P K R P R P V I M S P K R R T

CymR

1,400 1,410 1,420 1,430 1,440 1,450 1,460 1,470 1,480 1,490

PaqCI
BspMI
BfuAI
PasI PasI

AGGCAGAGCGCGCAATGGAGACCCAGGGCAAGTTGATTGCAGCGGCCCTGGGGGTTTTACGGGAAAAAGGTTACGCGGGATTCCGGATCGCAGATGTGCCCGGTGCT
TCCGTCTCGCGCGTTACCTCTGGGTCCCGTTCAACTAACGTCGCCGGGACCCCCAAAATGCCCTTTTTCCAATGCGCCCTAAGGCCTAGCGTCTACACGGGCCACGA
Q A E R A M E T Q G K L I A A A L G V L R E K G Y A G F R I A D V P G A

CymR

1,500 1,510 1,520 1,530 1,540 1,550 1,560 1,570 1,580 1,590 1,600

BcgI

GCAGGTGTCTCGAGAGGAGCGCAGAGCCATCATTTCCCGACAAAGCTTGAGCTTCTGCTTGCCACTTTTGAATGGCTTTACGAACAGATCACCGAACGCAGTCGGGC
CGTCCACAGAGCTCTCCTCGCGTCTCGGTAGTAAAGGGCTGTTTCGAACTCGAAGACGAACGGTGAAAACTTACCGAAATGCTTGTCTAGTGGCTTGCGTCAGCCCG
A G V S R G A Q S H H F P T K L E L L L A T F E W L Y E Q I T E R S R A

CymR

1,610 1,620 1,630 1,640 1,650 1,660 1,670 1,680 1,690 1,700 1,710

TCGATTAGCGAAATTGAAGCCAGAGGATGACGTCATCCAGCAAATGCTGGACGACGCCGCCGAATTTTTCCTCGACGATGACTTCTCTATCAGCCTTGATTTGATTG
AGCTAATCGCTTTAACTTCGGTCTCCTACTGCAGTAGGTCGTTTACGACCTGCTGCGGCGGCTTAAAAAGGAGCTGCTACTGAAGAGATAGTCGGAACTAAACTAAC
R L A K L K P E D D V I Q Q M L D D A A E F F L D D D F S I S L D L I

CymR

1,720 1,730 1,740 1,750 1,760 1,770 1,780 1,790 1,800 1,810

TGGCTGCCGACCGGGATCCAGCGTTACGCGAGGGTATTCAGCGCACGGTAGAGAGGAATCGGTTTGTCGTCGAGGATATGTGGCTTGGTGTTCTGGTGAGCCGTGGT
ACCGACGGCTGGCCCTAGGTCGCAATGCGCTCCCATAAGTCGCGTGCCATCTCTCCTTAGCCAAACAGCAGCTCCTATACACCGAACCACAAGACCACTCGGCACCA
V A A D R D P A L R E G I Q R T V E R N R F V V E D M W L G V L V S R G

CymR

1,820 1,830 1,840 1,850 1,860 1,870 1,880 1,890 1,900 1,910 1,920

EcoRV

CTTTCGCGTGATGATGCAGAAGATATCCTTTGGTTGATATTCAATTCGGTGCGTGGGCTTGCTGTTCGTAGCCTATGGCAGAAGGACAAAGAACGCTTTGAGCGTGT
GAAAGCGCACTACTACGTCTTCTATAGGAAACCAACTATAAGTTAAGCCACGCACCCGAACGACAAGCATCGGATACCGTCTTCCTGTTTCTTGCGAAACTCGCACA
L S R D D A E D I L W L I F N S V R G L A V R S L W Q K D K E R F E R V

CymR

1,930 1,940 1,950 1,960 1,970 1,980 1,990 2,000 2,010 2,020 2,030

CAGGAACTCGACACTCGAAATTGCGCGAGAGCGGTACGCGAAATTCAAGCGCGCGGCCAAACCCTCTGGCCGGTCTCTCTCTGTTCGAGAGGGCAGAGGAAGTCTTC
GTCCTTGAGCTGTGAGCTTTAACGCGCTCTCGCCATGCGCTTTAAGTTCGCGCGCCGGTTTGGGAGACCGGCCAGAGAGAGACAAGCTCTCCCGTCTCCTTCAGAAG
R N S T L E I A R E R Y A K F K R A A K P S G

CymR
T2A

2,040 2,050 2,060 2,070 2,080 2,090 2,100 2,110 2,120 2,130 2,140

EcoRI MscI MauBI

TAACATGCGGTGACGTGGAGGAGAATCCCGGCCCTGAATTCATGGCCAAGTTGACCAGTGCCGTTCCGGTGCTCACCGCGCGCGACGTCGCCGGAGCGGTCGAGTTC
ATTGTACGCCACTGCACCTCCTCTTAGGGCCGGGACTTAAGTACCGGTTCAACTGGTCACGGCAAGGCCACGAGTGGCGCGCGCTGCAGCGGCCTCGCCAGCTCAAG
M A K L T S A V P V L T A R D V A G A V E F

T2A
bleo

2,150 2,160 2,170 2,180 2,190 2,200 2,210 2,220 2,230 2,240

SmaI TspMI XmaI SgrAI SexAI

TGGACCGACCGGCTCGGGTTCTCCCGGGACTTCGTGGAGGACGACTTCGCCGGTGTGGTCCGGGACGACGTGACCCTGTTCATCAGCGCGGTCCAGGACCAGGTGGT
ACCTGGCTGGCCGAGCCCAAGAGGGCCCTGAAGCACCTCCTGCTGAAGCGGCCACACCAGGCCCTGCTGCACTGGGACAAGTAGTCGCGCCAGGTCCTGGTCCACCA
W T D R L G F S R D F V E D D F A G V V R D D V T L F I S A V Q D Q V V

bleo

2,250 2,260 2,270 2,280 2,290 2,300 2,310 2,320 2,330 2,340 2,350

FseI

GCCGGACAACACCCTGGCCTGGGTGTGGGTGCGCGGCCTGGACGAGCTGTACGCCGAGTGGTCGGAGGTCGTGTCCACGAACTTCCGGGACGCCTCCGGGCCGGCCA
CGGCCTGTTGTGGGACCGGACCCACACCCACGCGCCGGACCTGCTCGACATGCGGCTCACCAGCCTCCAGCACAGGTGCTTGAAGGCCCTGCGGAGGCCCGGCCGGT
P D N T L A W V W V R G L D E L Y A E W S E V V S T N F R D A S G P A

bleo

2,360 2,370 2,380 2,390 2,400 2,410 2,420 2,430 2,440 2,450 2,460

SalI Tth111I PflFI DraIII

TGACCGAGATCGGCGAGCAGCCGTGGGGGCGGGAGTTCGCCCTGCGCGACCCGGCCGGCAACTGCGTGCACTTCGTGGCCGAGGAGCAGGACTGAGTCGACTCGACA
ACTGGCTCTAGCCGCTCGTCGGCACCCCCGCCCTCAAGCGGGACGCGCTGGGCCGGCCGTTGACGCACGTGAAGCACCGGCTCCTCGTCCTGACTCAGCTGAGCTGT
M T E I G E Q P W G R E F A L R D P A G N C V H F V A E E Q D *

bleo

2,470 2,480 2,490 2,500 2,510 2,520 2,530 2,540 2,550 2,560

ATCAACCTCTGGATTACAAAATTTGTGAAAGATTGACTGGTATTCTTAACTATGTTGCTCCTTTTACGCTATGTGGATACGCTGCTTTAATGCCTTTGTATCATGCT
TAGTTGGAGACCTAATGTTTTAAACACTTTCTAACTGACCATAAGAATTGATACAACGAGGAAAATGCGATACACCTATGCGACGAAATTACGGAAACATAGTACGA

WPRE

2,570 2,580 2,590 2,600 2,610 2,620 2,630 2,640 2,650 2,660 2,670

```
ATTGCTTCCCGTATGGCTTTCATTTTCTCCTCCTTGTATAAATCCTGGTTGCTGTCTCTTTATGAGGAGTTGTGGCCCGTTGTCAGGCAACGTGGCGTGGTGTGCAC
TAACGAAGGGCATACCGAAAGTAAAAGAGGAGGAACATATTTAGGACCAACGACAGAGAAATACTCCTCAACACCGGGCAACAGTCCGTTGCACCGCACCACACGTG
WPRE
2,680 2,690 2,700 2,710 2,720 2,730 2,740 2,750 2,760 2,770 2,780

TGTGTTTGCTGACGCAACCCCCACTGGTTGGGGCATTGCCACCACCTGTCAGCTCCTTTCCGGGACTTTCGCTTTCCCCCTCCCTATTGCCACGGCGGAACTCATCG
ACACAAACGACTGCGTTGGGGGTGACCAACCCCGTAACGGTGGTGGACAGTCGAGGAAAGGCCCTGAAAGCGAAAGGGGGAGGGATAACGGTGCCGCCTTGAGTAGC
WPRE
2,790 2,800 2,810 2,820 2,830 2,840 2,850 2,860 2,870 2,880

CCGCCTGCCTTGCCCGCTGCTGGACAGGGGCTCGGCTGTTGGGCACTGACAATTCCGTGGTGTTGTCGGGGAAATCATCGTCCTTTCCTTGGCTGCTCGCCTGTGTT
GGCGGACGGAACGGGCGACGACCTGTCCCCGAGCCGACAACCCGTGACTGTTAAGGCACCACAACAGCCCCTTTAGTAGCAGGAAAGGAACCGACGAGCGGACACAA
WPRE
2,890 2,900 2,910 2,920 2,930 2,940 2,950 2,960 2,970 2,980 2,990

GCCACCTGGATTCTGCGCGGGACGTCCTTCTGCTACGTCCCTTCGGCCCTCAATCCAGCGGACCTTCCTTCCCGCGGCCTGCTGCCGGCTCTGCGGCCTCTTCCGCG
CGGTGGACCTAAGACGCGCCCTGCAGGAAGACGATGCAGGGAAGCCGGGAGTTAGGTCGCCTGGAAGGAAGGGCGCCGGACGACGGCCGAGACGCCGGAGAAGGCGC
WPRE
3,000 3,010 3,020 3,030 3,040 3,050 3,060 3,070 3,080 3,090 3,100

KpnI
Acc65I
TCTTCGCCTTCGCCCTCAGACGAGTCGGATCTCCCTTTGGGCCGCCTCCCCGCCTGGTACCTTTAAGACCAATGACTTACAAGGCAGCTGTAGATCTTAGCCACTTT
AGAAGCGGAAGCGGGAGTCTGCTCAGCCTAGAGGGAAACCCGGCGGAGGGGCGGACCATGGAAATTCTGGTTACTGAATGTTCCGTCGACATCTAGAATCGGTGAAA
WPRE
3,110 3,120 3,130 3,140 3,150 3,160 3,170 3,180 3,190 3,200 3,210

TTAAAAGAAAAGGGGGGACTGGAAGGGCTAATTCACTCCCAACGAAGATAAGATCTGCTTTTTGCTTGTACTGGGTCTCTCTGGTTAGACCAGATCTGAGCCTGGGA
AATTTTCTTTTCCCCCCTGACCTTCCCGATTAAGTGAGGGTTGCTTCTATTCTAGACGAAAAACGAACATGACCCAGAGAGACCAATCTGGTCTAGACTCGGACCCT
3'LTR
3,220 3,230 3,240 3,250 3,260 3,270 3,280 3,290 3,300 3,310

GCTCTCTGGCTAACTAGGGAACCCACTGCTTAAGCCTCAATAAAGCTTGCCTTGAGTGCTTCAAGTAGTGTGTGCCCGTCTGTTGTGTGACTCTGGTAACTAGAGAT
CGAGAGACCGATTGATCCCTTGGGTGACGAATTCGGAGTTATTTCGAACGGAACTCACGAAGTTCATCACACACGGGCAGACAACACACTGAGACCATTGATCTCTA
3'LTR
3,320 3,330 3,340 3,350 3,360 3,370 3,380 3,390 3,400 3,410 3,420

CCCTCAGACCCTTTTAGTCAGTGTGGAAAATCTCTAGCAGTAGTAGTTCATGTCATCTTATTATTCAGTATTTATAACTTGCAAAGAAATGAATATCAGAGAGTGAG
GGGAGTCTGGGAAAATCAGTCACACCTTTTAGAGATCGTCATCATCAAGTACAGTAGAATAATAAGTCATAAATATTGAACGTTTCTTTACTTATAGTCTCTCACTC
3'LTR
3,430 3,440 3,450 3,460 3,470 3,480 3,490 3,500 3,510 3,520 3,530
```

AGGAACTTGTTTATTGCAGCTTATAATGGTTACAAATAAAGCAATAGCATCACAAATTTCACAAATAAAGCATTTTTTTCACTGCATTCTAGTTGTGGTTTGTCCAA
TCCTTGAACAAATAACGTCGAATATTACCAATGTTTATTTCGTTATCGTAGTGTTTAAAGTGTTTATTTCGTAAAAAAAGTGACGTAAGATCAACACCAAACAGGTT

SV40 Polyadenylation signal

3,540 3,550 3,560 3,570 3,580 3,590 3,600 3,610 3,620 3,630

ACTCATCAATGTATCTTATCATGTCTGGCTCTAGCTATCCCGCCCCTAACTCCGCCCATCCCGCCCCTAACTCCGCCCAGTTCCGCCCATTCTCCGCCCCATGGCTG
TGAGTAGTTACATAGAATAGTACAGACCGAGATCGATAGGGCGGGGATTGAGGCGGGTAGGGCGGGGATTGAGGCGGGTCAAGGCGGGTAAGAGGCGGGGTACCGAC

SV40 Polyad...ion signal SV40 Ori

3,640 3,650 3,660 3,670 3,680 3,690 3,700 3,710 3,720 3,730 3,740

SfiI

ACTAATTTTTTTTATTTATGCAGAGGCCGAGGCCGCCTCGGCCTCTGAGCTATTCCAGAAGTAGTGAGGAGGCTTTTTTGGAGGCCTAGACTTTTGCAGAGACCAAA
TGATTAAAAAAAATAAATACGTCTCCGGCTCCGGCGGAGCCGGAGACTCGATAAGGTCTTCATCACTCCTCCGAAAAAACCTCCGGATCTGAAAACGTCTCTGGTTT

SV40 Ori

3,750 3,760 3,770 3,780 3,790 3,800 3,810 3,820 3,830 3,840 3,850

TTCGTAATCATGTCATAGCTGTTTCCTGTGTGAAATTGTTATCCGCTCACAATTCCACACAACATACGAGCCGGAAGCATAAAGTGTAAAGCCTGGGGTGCCTAATG
AAGCATTAGTACAGTATCGACAAAGGACACACTTTAACAATAGGCGAGTGTTAAGGTGTGTTGTATGCTCGGCCTTCGTATTTCACATTTCGGACCCCACGGATTAC

3,860 3,870 3,880 3,890 3,900 3,910 3,920 3,930 3,940 3,950

AGTGAGCTAACTCACATTAATTGCGTTGCGCTCACTGCCCGCTTTCCAGTCGGGAAACCTGTCGTGCCAGCTGCATTAATGAATCGGCCAACGCGCGGGGAGAGGCG
TCACTCGATTGAGTGTAATTAACGCAACGCGAGTGACGGGCGAAAGGTCAGCCCTTTGGACAGCACGGTCGACGTAATTACTTAGCCGGTTGCGCGCCCCTCTCCGC

3,960 3,970 3,980 3,990 4,000 4,010 4,020 4,030 4,040 4,050 4,060

BspQI
SapI

GTTTGCGTATTGGGCGCTCTTCCGCTTCCTCGCTCACTGACTCGCTGCGCTCGGTCGTTCGGCTGCGGCGAGCGGTATCAGCTCACTCAAAGGCGGTAATACGGTTA
CAAACGCATAACCCGCGAGAAGGCGAAGGAGCGAGTGACTGAGCGACGCGAGCCAGCAAGCCGACGCCGCTCGCCATAGTCGAGTGAGTTTCCGCCATTATGCCAAT

4,070 4,080 4,090 4,100 4,110 4,120 4,130 4,140 4,150 4,160 4,170

AflIII
PciI

TCCACAGAATCAGGGGATAACGCAGGAAAGAACATGTGAGCAAAAGGCCAGCAAAAGGCCAGGAACCGTAAAAAGGCCGCGTTGCTGGCGTTTTTCCATAGGCTCCG
AGGTGTCTTAGTCCCCTATTGCGTCCTTTCTTGTACACTCGTTTTCCGGTCGTTTTCCGGTCCTTGGCATTTTTCCGGCGCAACGACCGCAAAAAGGTATCCGAGGC

pUC ori

4,180 4,190 4,200 4,210 4,220 4,230 4,240 4,250 4,260 4,270 4,280

CCCCCCTGACGAGCATCACAAAAATCGACGCTCAAGTCAGAGGTGGCGAAACCCGACAGGACTATAAAGATACCAGGCGTTTCCCCCTGGAAGCTCCCTCGTGCGCT
GGGGGGACTGCTCGTAGTGTTTTTAGCTGCGAGTTCAGTCTCCACCGCTTTGGGCTGTCCTGATATTTCTATGGTCCGCAAAGGGGGACCTTCGAGGGAGCACGCGA

pUC ori

4,290 4,300 4,310 4,320 4,330 4,340 4,350 4,360 4,370 4,380

bArr2-HA-TEV219 pCDH-CuO-MCS-EF1α-CymR-T2A-Bleo3 SparQ (10907 bp)

CTCCTGTTCCGACCCTGCCGCTTACCGGATACCTGTCCGCCTTTCTCCCTTCGGGAAGCGTGGCGCTTTCTCATAGCTCACGCTGTAGGTATCTCAGTTCGGTGTAG
GAGGACAAGGCTGGGACGGCGAATGGCCTATGGACAGGCGGAAAGAGGGAAGCCCTTCGCACCGCGAAAGAGTATCGAGTGCGACATCCATAGAGTCAAGCCACATC

pUC ori

4,390 4,400 4,410 4,420 4,430 4,440 4,450 4,460 4,470 4,480 4,490

GTCGTTCGCTCCAAGCTGGGCTGTGTGCACGAACCCCCCGTTCAGCCCGACCGCTGCGCCTTATCCGGTAACTATCGTCTTGAGTCCAACCCGGTAAGACACGACTT
CAGCAAGCGAGGTTCGACCCGACACACGTGCTTGGGGGGCAAGTCGGGCTGGCGACGCGGAATAGGCCATTGATAGCAGAACTCAGGTTGGGCCATTCTGTGCTGAA

pUC ori

4,500 4,510 4,520 4,530 4,540 4,550 4,560 4,570 4,580 4,590 4,600

ATCGCCACTGGCAGCAGCCACTGGTAACAGGATTAGCAGAGCGAGGTATGTAGGCGGTGCTACAGAGTTCTTGAAGTGGTGGCCTAACTACGGCTACACTAGAAGGA
TAGCGGTGACCGTCGTCGGTGACCATTGTCCTAATCGTCTCGCTCCATACATCCGCCACGATGTCTCAAGAACTTCACCACCGGATTGATGCCGATGTGATCTTCCT

pUC ori

4,610 4,620 4,630 4,640 4,650 4,660 4,670 4,680 4,690 4,700

CAGTATTTGGTATCTGCGCTCTGCTGAAGCCAGTTACCTTCGGAAAAAGAGTTGGTAGCTCTTGATCCGGCAAACAAACCACCGCTGGTAGCGGTGGTTTTTTTGTT
GTCATAAACCATAGACGCGAGACGACTTCGGTCAATGGAAGCCTTTTTCTCAACCATCGAGAACTAGGCCGTTTGTTTGGTGGCGACCATCGCCACCAAAAAAACAA

pUC ori

4,710 4,720 4,730 4,740 4,750 4,760 4,770 4,780 4,790 4,800 4,810

TGCAAGCAGCAGATTACGCGCAGAAAAAAAGGATCTCAAGAAGATCCTTTGATCTTTTCTACGGGGTCTGACGCTCAGTGGAACGAAAACTCACGTTAAGGGATTTT
ACGTTCGTCGTCTAATGCGCGTCTTTTTTTCCTAGAGTTCTTCTAGGAAACTAGAAAAGATGCCCCAGACTGCGAGTCACCTTGCTTTTGAGTGCAATTCCCTAAAA

pUC ori

4,820 4,830 4,840 4,850 4,860 4,870 4,880 4,890 4,900 4,910 4,920

GGTCATGAGATTATCAAAAAGGATCTTCACCTAGATCCTTTTAAATTAAAAATGAAGTTTTAAATCAATCTAAAGTATATATGAGTAAACTTGGTCTGACAGTTACC
CCAGTACTCTAATAGTTTTTCCTAGAAGTGGATCTAGGAAAATTTAATTTTTACTTCAAAATTTAGTTAGATTTCATATATACTCATTTGAACCAGACTGTCAATGG

4,930 4,940 4,950 4,960 4,970 4,980 4,990 5,000 5,010 5,020

AhdI

AATGCTTAATCAGTGAGGCACCTATCTCAGCGATCTGTCTATTTCGTTCATCCATAGTTGCCTGACTCCCCGTCGTGTAGATAACTACGATACGGGAGGGCTTACCA
TTACGAATTAGTCACTCCGTGGATAGAGTCGCTAGACAGATAAAGCAAGTAGGTATCAACGGACTGAGGGGCAGCACATCTATTGATGCTATGCCCTCCCGAATGGT

AmpR

5,030 5,040 5,050 5,060 5,070 5,080 5,090 5,100 5,110 5,120 5,130

TCTGGCCCCAGTGCTGCAATGATACCGCGAGACCCACGCTCACCGGCTCCAGATTTATCAGCAATAAACCAGCCAGCCGGAAGGGCCGAGCGCAGAAGTGGTCCTGC
AGACCGGGGTCACGACGTTACTATGGCGCTCTGGGTGCGAGTGGCCGAGGTCTAAATAGTCGTTATTTGGTCGGTCGGCCTTCCCGGCTCGCGTCTTCACCAGGACG

AmpR

5,140 5,150 5,160 5,170 5,180 5,190 5,200 5,210 5,220 5,230 5,240

FspI

AACTTTATCCGCCTCCATCCAGTCTATTAATTGTTGCCGGGAAGCTAGAGTAAGTAGTTCGCCAGTTAATAGTTTGCGCAACGTTGTTGCCATTGCTACAGGCATCG
TTGAAATAGGCGGAGGTAGGTCAGATAATTAACAACGGCCCTTCGATCTCATTCATCAAGCGGTCAATTATCAAACGCGTTGCAACAACGGTAACGATGTCCGTAGC

AmpR

5,250 5,260 5,270 5,280 5,290 5,300 5,310 5,320 5,330 5,340 5,350

TGGTGTCACGCTCGTCGTTTGGTATGGCTTCATTCAGCTCCGGTTCCCAACGATCAAGGCGAGTTACATGATCCCCCATGTTGTGCAAAAAAGCGGTTAGCTCCTTC
ACCACAGTGCGAGCAGCAAACCATACCGAAGTAAGTCGAGGCCAAGGGTTGCTAGTTCCGCTCAATGTACTAGGGGGTACAACACGTTTTTTCGCCAATCGAGGAAG

AmpR

5,360 5,370 5,380 5,390 5,400 5,410 5,420 5,430 5,440 5,450

GGTCCTCCGATCGTTGTCAGAAGTAAGTTGGCCGCAGTGTTATCACTCATGGTTATGGCAGCACTGCATAATTCTCTTACTGTCATGCCATCCGTAAGATGCTTTTC
CCAGGAGGCTAGCAACAGTCTTCATTCAACCGGCGTCACAATAGTGAGTACCAATACCGTCGTGACGTATTAAGAGAATGACAGTACGGTAGGCATTCTACGAAAAG

AmpR

5,460 5,470 5,480 5,490 5,500 5,510 5,520 5,530 5,540 5,550 5,560

BcgI

TGTGACTGGTGAGTACTCAACCAAGTCATTCTGAGAATAGTGTATGCGGCGACCGAGTTGCTCTTGCCCGGCGTCAATACGGGATAATACCGCGCCACATAGCAGAA
ACACTGACCACTCATGAGTTGGTTCAGTAAGACTCTTATCACATACGCCGCTGGCTCAACGAGAACGGGCCGCAGTTATGCCCTATTATGGCGCGGTGTATCGTCTT

AmpR

5,570 5,580 5,590 5,600 5,610 5,620 5,630 5,640 5,650 5,660 5,670

XmnI

CTTTAAAAGTGCTCATCATTGGAAAACGTTCTTCGGGGCGAAAACTCTCAAGGATCTTACCGCTGTTGAGATCCAGTTCGATGTAACCCACTCGTGCACCCAACTGA
GAAATTTTCACGAGTAGTAACCTTTTGCAAGAAGCCCCGCTTTTGAGAGTTCCTAGAATGGCGACAACTCTAGGTCAAGCTACATTGGGTGAGCACGTGGGTTGACT

AmpR

5,680 5,690 5,700 5,710 5,720 5,730 5,740 5,750 5,760 5,770

TCTTCAGCATCTTTTACTTTCACCAGCGTTTCTGGGTGAGCAAAAACAGGAAGGCAAAATGCCGCAAAAAAGGGAATAAGGGCGACACGGAAATGTTGAATACTCAT
AGAAGTCGTAGAAAATGAAAGTGGTCGCAAAGACCCACTCGTTTTTGTCCTTCCGTTTTACGGCGTTTTTTCCCTTATTCCCGCTGTGCCTTTACAACTTATGAGTA

AmpR

5,780 5,790 5,800 5,810 5,820 5,830 5,840 5,850 5,860 5,870 5,880

SspI

ACTCTTCCTTTTTCAATATTATTGAAGCATTTATCAGGGTTATTGTCTCATGAGCGGATACATATTTGAATGTATTTAGAAAAATAAACAAATAGGGGTTCCGCGCA
TGAGAAGGAAAAAGTTATAATAACTTCGTAAATAGTCCCAATAACAGAGTACTCGCCTATGTATAAACTTACATAAATCTTTTTATTTGTTTATCCCCAAGGCGCGT

5,890 5,900 5,910 5,920 5,930 5,940 5,950 5,960 5,970 5,980 5,990

CATTTCCCCGAAAAGTGCCACCTGACGTCTAAGAAACCATTATTATCATGACATTAACCTATAAAAATAGGCGTATCACGAGGCCCTTTCGTCTCGCGCGTTTCGGT
GTAAAGGGGCTTTTCACGGTGGACTGCAGATTCTTTGGTAATAATAGTACTGTAATTGGATATTTTTATCCGCATAGTGCTCCGGGAAAGCAGAGCGCGCAAAGCCA

6,000 6,010 6,020 6,030 6,040 6,050 6,060 6,070 6,080 6,090

GATGACGGTGAAAACCTCTGACACATGCAGCTCCCGGAGACGGTCACAGCTTGTCTGTAAGCGGATGCCGGGAGCAGACAAGCCCGTCAGGGCGCGTCAGCGGGTGT
CTACTGCCACTTTTGGAGACTGTGTACGTCGAGGGCCTCTGCCAGTGTCGAACAGACATTCGCCTACGGCCCTCGTCTGTTCGGGCAGTCCCGCGCAGTCGCCCACA

6,100 6,110 6,120 6,130 6,140 6,150 6,160 6,170 6,180 6,190 6,200

BstAPI
NdeI

TGGCGGGTGTCGGGGCTGGCTTAACTATGCGGCATCAGAGCAGATTGTACTGAGAGTGCACCATATGCGGTGTGAAATACCGCACAGATGCGTAAGGAGAAAATACC
ACCGCCCACAGCCCCGACCGAATTGATACGCCGTAGTCTCGTCTAACATGACTCTCACGTGGTATACGCCACACTTTATGGCGTGTCTACGCATTCCTCTTTTATGG

6,210 6,220 6,230 6,240 6,250 6,260 6,270 6,280 6,290 6,300 6,310

FspI

GCATCAGGCGCCATTCGCCATTCAGGCTGCGCAACTGTTGGGAAGGGCGATCGGTGCGGGCCTCTTCGCTATTACGCCAGCTGGCGAAAGGGGGATGTGCTGCAAGG
CGTAGTCCGCGGTAAGCGGTAAGTCCGACGCGTTGACAACCCTTCCCGCTAGCCACGCCCGGAGAAGCGATAATGCGGTCGACCGCTTTCCCCCTACACGACGTTCC

6,320 6,330 6,340 6,350 6,360 6,370 6,380 6,390 6,400 6,410 6,420

AflIII
MluI

CGATTAAGTTGGGTAACGCCAGGGTTTTCCCAGTCACGACGTTGTAAAACGACGGCCAGTGCCAAGCTGACGCGTGTAGTCTTATGCAATACTCTTGTAGTCTTGCA
GCTAATTCAACCCATTGCGGTCCCAAAAGGGTCAGTGCTGCAACATTTTGCTGCCGGTCACGGTTCGACTGCGCACATCAGAATACGTTATGAGAACATCAGAACGT

RSV

6,430 6,440 6,450 6,460 6,470 6,480 6,490 6,500 6,510 6,520

SphI BaeI

ACATGGTAACGATGAGTTAGCAACATGCCTTACAAGGAGAGAAAAAGCACCGTGCATGCCGATTGGTGGAAGTAAGGTGGTACGATCGTGCCTTATTAGGAAGGCAA
TGTACCATTGCTACTCAATCGTTGTACGGAATGTTCCTCTCTTTTTCGTGGCACGTACGGCTAACCACCTTCATTCCACCATGCTAGCACGGAATAATCCTTCCGTT

RSV

6,530 6,540 6,550 6,560 6,570 6,580 6,590 6,600 6,610 6,620 6,630

CAGACGGGTCTGACATGGATTGGACGAACCACTGAATTGCCGCATTGCAGAGATATTGTATTTAAGTGCCTAGCTCGATACATAAACGGGTCTCTCTGGTTAGACCA
GTCTGCCCAGACTGTACCTAACCTGCTTGGTGACTTAACGGCGTAACGTCTCTATAACATAAATTCACGGATCGAGCTATGTATTTGCCCAGAGAGACCAATCTGGT

RSV HIV LTR

6,640 6,650 6,660 6,670 6,680 6,690 6,700 6,710 6,720 6,730 6,740

GATCTGAGCCTGGGAGCTCTCTGGCTAACTAGGGAACCCACTGCTTAAGCCTCAATAAAGCTTGCCTTGAGTGCTTCAAGTAGTGTGTGCCCGTCTGTTGTGTGACT
CTAGACTCGGACCCTCGAGAGACCGATTGATCCCTTGGGTGACGAATTCGGAGTTATTTCGAACGGAACTCACGAAGTTCATCACACACGGGCAGACAACACACTGA

HIV LTR

6,750 6,760 6,770 6,780 6,790 6,800 6,810 6,820 6,830 6,840

CTGGTAACTAGAGATCCCTCAGACCCTTTTAGTCAGTGTGGAAAATCTCTAGCAGTGGCGCCCGAACAGGGACTTGAAAGCGAAAGGGAAACCAGAGGAGCTCTCTC
GACCATTGATCTCTAGGGAGTCTGGGAAAATCAGTCACACCTTTTAGAGATCGTCACCGCGGGCTTGTCCCTGAACTTTCGCTTTCCCTTTGGTCTCCTCGAGAGAG

HIV LTR

6,850 6,860 6,870 6,880 6,890 6,900 6,910 6,920 6,930 6,940 6,950

bArr2-HA-TEV219 pCDH-CuO-MCS-EF1α-CymR-T2A-Bleo3 SparQ (10907 bp)

GACGCAGGACTCGGCTTGCTGAAGCGCGCACGGCAAGAGGCGAGGGGCGGCGACTGGTGAGTACGCCAAAAATTTTGACTAGCGGAGGCTAGAAGGAGAGAGATGGG
CTGCGTCCTGAGCCGAACGACTTCGCGCGTGCCGTTCTCCGCTCCCCGCCGCTGACCACTCATGCGGTTTTTAAAACTGATCGCCTCCGATCTTCCTCTCTCTACCC

Psi

6,960 6,970 6,980 6,990 7,000 7,010 7,020 7,030 7,040 7,050 7,060

NruI XmnI

TGCGAGAGCGTCAGTATTAAGCGGGGGAGAATTAGATCGCGATGGGAAAAAATTCGGTTAAGGCCAGGGGGAAAGAAAAAATATAAATTAAAACATATAGTATGGGC
ACGCTCTCGCAGTCATAATTCGCCCCCTCTTAATCTAGCGCTACCCTTTTTTAAGCCAATTCCGGTCCCCCTTTCTTTTTTATATTTAATTTTGTATATCATACCCG

Gag

7,070 7,080 7,090 7,100 7,110 7,120 7,130 7,140 7,150 7,160

AAGCAGGGAGCTAGAACGATTCGCAGTTAATCCTGGCCTGTTAGAAACATCAGAAGGCTGTAGACAAATACTGGGACAGCTACAACCATCCCTTCAGACAGGATCAG
TTCGTCCCTCGATCTTGCTAAGCGTCAATTAGGACCGGACAATCTTTGTAGTCTTCCGACATCTGTTTATGACCCTGTCGATGTTGGTAGGGAAGTCTGTCCTAGTC

Gag

7,170 7,180 7,190 7,200 7,210 7,220 7,230 7,240 7,250 7,260 7,270

BspQI
SapI

AAGAACTTAGATCATTATATAATACAGTAGCAACCCTCTATTGTGTGCATCAAAGGATAGAGATAAAAGACACCAAGGAAGCTTTAGACAAGATAGAGGAAGAGCAA
TTCTTGAATCTAGTAATATATTATGTCATCGTTGGGAGATAACACACGTAGTTTCCTATCTCTATTTTCTGTGGTTCCTTCGAAATCTGTTCTATCTCCTTCTCGTT

Gag

7,280 7,290 7,300 7,310 7,320 7,330 7,340 7,350 7,360 7,370 7,380

MfeI

AACAAAAGTAAGACCACCGCACAGCAAGCGGCCACTGATCTTCAGACCTGGAGGAGGAGATATGAGGGACAATTGGAGAAGTGAATTATATAAATATAAAGTAGTAA
TTGTTTTCATTCTGGTGGCGTGTCGTTCGCCGGTGACTAGAAGTCTGGACCTCCTCCTCTATACTCCCTGTTAACCTCTTCACTTAATATATTTATATTTCATCATT

Gag Env

7,390 7,400 7,410 7,420 7,430 7,440 7,450 7,460 7,470 7,480 7,490

AAATTGAACCATTAGGAGTAGCACCCACCAAGGCAAAGAGAAGAGTGGTGCAGAGAGAAAAAAGAGCAGTGGGAATAGGAGCTTTGTTCCTTGGGTTCTTGGGAGCA
TTTAACTTGGTAATCCTCATCGTGGGTGGTTCCGTTTCTCTTCTCACCACGTCTCTCTTTTTTCTCGTCACCCTTATCCTCGAAACAAGGAACCCAAGAACCCTCGT

RRE
Env

7,500 7,510 7,520 7,530 7,540 7,550 7,560 7,570 7,580 7,590

BbvCI

GCAGGAAGCACTATGGGCGCAGCGTCAATGACGCTGACGGTACAGGCCAGACAATTATTGTCTGGTATAGTGCAGCAGCAGAACAATTTGCTGAGGGCTATTGAGGC
CGTCCTTCGTGATACCCGCGTCGCAGTTACTGCGACTGCCATGTCCGGTCTGTTAATAACAGACCATATCACGTCGTCGTCTTGTTAAACGACTCCCGATAACTCCG

RRE
Env

7,600 7,610 7,620 7,630 7,640 7,650 7,660 7,670 7,680 7,690 7,700

bArr2-HA-TEV219 pCDH-CuO-MCS-EF1α-CymR-T2A-Bleo3 SparQ (10907 bp)

BstAPI

GCAACAGCATCTGTTGCAACTCACAGTCTGGGGCATCAAGCAGCTCCAGGCAAGAATCCTGGCTGTGGAAAGATACCTAAAGGATCAACAGCTCCTGGGGATTTGGG
CGTTGTCGTAGACAACGTTGAGTGTCAGACCCCGTAGTTCGTCGAGGTCCGTTCTTAGGACCGACACCTTTCTATGGATTTCCTAGTTGTCGAGGACCCCTAAACCC

RRE
Env

7,710 7,720 7,730 7,740 7,750 7,760 7,770 7,780 7,790 7,800 7,810

GTTGCTCTGGAAAACTCATTTGCACCACTGCTGTGCCTTGGAATGCTAGTTGGAGTAATAAATCTCTGGAACAGATTTGGAATCACACGACCTGGATGGAGTGGGAC
CAACGAGACCTTTTGAGTAAACGTGGTGACGACACGGAACCTTACGATCAACCTCATTATTTAGAGACCTTGTCTAAACCTTAGTGTGCTGGACCTACCTCACCCTG

RRE
Env

7,820 7,830 7,840 7,850 7,860 7,870 7,880 7,890 7,900 7,910

AGAGAAATTAACAATTACACAAGCTTAATACACTCCTTAATTGAAGAATCGCAAACCAGCAAGAAAAGAATGAACAAGAATTATTGGAATTAGATAAATGGGCAAGT
TCTCTTTAATTGTTAATGTGTTCGAATTATGTGAGGAATTAACTTCTTAGCGTTTGGTCGTTCTTTTCTTACTTGTTCTTAATAACCTTAATCTATTTACCCGTTCA

Env

7,920 7,930 7,940 7,950 7,960 7,970 7,980 7,990 8,000 8,010 8,020

TTGTGGAATTGGTTTAACATAACAAATTGGCTGTGGTATATAAAATTATTCATAATGATAGTAGGAGGCTTGGTAGGTTTAAGAATAGTTTTTGCTGTACTTTCTAT
AACACCTTAACCAAATTGTATTGTTTAACCGACACCATATATTTTAATAAGTATTACTATCATCCTCCGAACCATCCAAATTCTTATCAAAAACGACATGAAAGATA

Env

8,030 8,040 8,050 8,060 8,070 8,080 8,090 8,100 8,110 8,120 8,130

PpuMI
KflI

AGTGAATAGAGTTAGGCAGGGATATTCACCATTATCGTTTCAGACCCACCTCCCAACCCCGAGGGGACCCGACAGGCCCGAAGGAATAGAAGAAGAAGGTGGAGAGA
TCACTTATCTCAATCCGTCCCTATAAGTGGTAATAGCAAAGTCTGGGTGGAGGGTTGGGGCTCCCCTGGGCTGTCCGGGCTTCCTTATCTTCTTCTTCCACCTCTCT

Env

8,140 8,150 8,160 8,170 8,180 8,190 8,200 8,210 8,220 8,230

HpaI

GAGACAGAGACAGATCCATTCGATTAGTGAACGGATCTCGACGGTATCGGTTAACTTTTAAAAGAAAAGGGGGGATTGGGGGGTACAGTGCAGGGGAAAGAATAGTA
CTCTGTCTCTGTCTAGGTAAGCTAATCACTTGCCTAGAGCTGCCATAGCCAATTGAAAATTTTCTTTTCCCCCCTAACCCCCCATGTCACGTCCCCTTTCTTATCAT

Env
cPPT

8,240 8,250 8,260 8,270 8,280 8,290 8,300 8,310 8,320 8,330 8,340

BspDI
ClaI SpeI

GACATAATAGCAACAGACATACAAACTAAAGAATTACAAAAACAAATTACAAAATTCAAAATTTTATCGATAGACTAGTTATTAATAGTAATCAATTACGGGGTCAT
CTGTATTATCGTTGTCTGTATGTTTGATTTCTTAATGTTTTTGTTTAATGTTTTAAGTTTTAAAATAGCTATCTGATCAATAATTATCATTAGTTAATGCCCCAGTA

cPPT
CMV + CUO

8,350 8,360 8,370 8,380 8,390 8,400 8,410 8,420 8,430 8,440 8,450

```
TAGTTCATAGCCCATATATGGAGTTCCGCGTTACATAACTTACGGTAAATGGCCCGCCTGGCTGACCGCCCAACGACCCCCGCCCATTGACGTCAATAATGACGTAT
ATCAAGTATCGGGTATATACCTCAAGGCGCAATGTATTGAATGCCATTTACCGGGCGGACCGACTGGCGGGTTGCTGGGGGCGGGTAACTGCAGTTATTACTGCATA
CMV + CUO
8,460 8,470 8,480 8,490 8,500 8,510 8,520 8,530 8,540 8,550 8,560

NdeI
GTTCCCATAGTAACGCCAATAGGGACTTTCCATTGACGTCAATGGGTGGAGTATTTACGGTAAACTGCCCACTTGGCAGTACATCAAGTGTATCATATGCCAAGTCC
CAAGGGTATCATTGCGGTTATCCCTGAAAGGTAACTGCAGTTACCCACCTCATAAATGCCATTTGACGGGTGAACCGTCATGTAGTTCACATAGTATACGGTTCAGG
CMV + CUO
8,570 8,580 8,590 8,600 8,610 8,620 8,630 8,640 8,650 8,660

SnaBI
BsaAI
GCCCCCTATTGACGTCAATGACGGTAAATGGCCCGCCTGGCATTATGCCCAGTACATGACCTTACGGGACTTTCCTACTTGGCAGTACATCTACGTATTAGTCATCG
CGGGGGATAACTGCAGTTACTGCCATTTACCGGGCGGACCGTAATACGGGTCATGTACTGGAATGCCCTGAAAGGATGAACCGTCATGTAGATGCATAATCAGTAGC
CMV + CUO
8,670 8,680 8,690 8,700 8,710 8,720 8,730 8,740 8,750 8,760 8,770

CspCI
CTATTACCATGGTGATGCGGTTTTGGCAGTACACCAATGGGCGTGGATAGCGGTTTGACTCACGGGGATTTCCAAGTCTCCACCCCATTGACGTCAATGGGAGTTTG
GATAATGGTACCACTACGCCAAAACCGTCATGTGGTTACCCGCACCTATCGCCAAACTGAGTGCCCCTAAAGGTTCAGAGGTGGGGTAACTGCAGTTACCCTCAAAC
CMV + CUO
8,780 8,790 8,800 8,810 8,820 8,830 8,840 8,850 8,860 8,870 8,880

TTTTGGCACCAAAATCAACGGGACTTTCCAAAATGTCGTAATACCCCGCCCCGTTGACGCAAATGGGCAAGCTTGCCGGGTCGAGGTAGGCGTGTACGGTGGGAGG
AAAACCGTGGTTTTAGTTGCCCTGAAAGGTTTTACAGCATTATGGGGCGGGGCAACTGCGTTTACCCGTTCGAACGGCCCAGCTCCATCCGCACATGCCACCCTCC
CMV + CUO    CMV + CUO
8,890 8,900 8,910 8,920 8,930 8,940 8,950 8,960 8,970 8,980

AscI
CCTATATAAGCAACCGGTATAATCAAACAGACCAGATTGTCTGTTTGTTACCGGTGTTTAGTGAACCGGGCGCGCCTCATATCGCCTGGAGACGCCATCCACGCTGT
GGATATATTCGTTGGCCATATTAGTTTGTCTGGTCTAACAGACAAACAATGGCCACAAATCACTTGGCCCGCGCGGAGTATAGCGGACCTCTGCGGTAGGTGCGACA
CMV + CUO
8,990 9,000 9,010 9,020 9,030 9,040 9,050 9,060 9,070 9,080 9,090

TTTGACCTCCATAGAAGACACCGGGACCGATCCAGCCTCCGCGGTCACTCTCTTCCGCATCGCTGTCTGCGAGGGCCAGCTGTTGGGCTCGCGGTTGAGGACAAACT
AAACTGGAGGTATCTTCTGTGGCCCTGGCTAGGTCGGAGGCGCCAGTGAGAGAAGGCGTAGCGACAGACGCTCCCGGTCGACAACCCGAGCGCCAACTCCTGTTTGA
CMV + CUO
9,100 9,110 9,120 9,130 9,140 9,150 9,160 9,170 9,180 9,190 9,200
```

bArr2-HA-TEV219 pCDH-CuO-MCS-EF1α-CymR-T2A-Bleo3 SparQ (10907 bp)

PpuMI

CTTCGCGGTCTTTCCAGTACTCTTGGATCGGAAACCCGTCGGCCTCCGAACGGTACTCCGCCACCGAGGGACCTGAGCGAGTCCGCATCGACCGGATCGGAAAACCT
GAAGCGCCAGAAAGGTCATGAGAACCTAGCCTTTGGGCAGCCGGAGGCTTGCCATGAGGCGGTGGCTCCCTGGACTCGCTCAGGCGTAGCTGGCCTAGCCTTTTGGA

CMV + CUO | CMV + CUO

9,210 9,220 9,230 9,240 9,250 9,260 9,270 9,280 9,290 9,300

BlpI

CTCGAGAAAGGCGTCTAACCAGTCACAGTCGCAAGGTAGGCTGAGCACCGTGGCGGGCGGCAGCGGGTGGCGGTCGGGGTTGTTTCTGGCGGAGGTGCTGCTGATGA
GAGCTCTTTCCGCAGATTGGTCAGTGTCAGCGTTCCATCCGACTCGTGGCACCGCCCGCCGTCGCCCACCGCCAGCCCCAACAAAGACCGCCTCCACGACGACTACT

CMV + CUO

9,310 9,320 9,330 9,340 9,350 9,360 9,370 9,380 9,390 9,400 9,410

TGTAATTAAAGTAGGCGGTCTTGAGACGGCGGATGGTCGAGGTGAGGTGTGGCAGGCTTGAGATCCAGCTGTTGGGGTGAGTACTCCCTCTCAAAAGCGGGCATTAC
ACATTAATTTCATCCGCCAGAACTCTGCCGCCTACCAGCTCCACTCCACACCGTCCGAACTCTAGGTCGACAACCCCACTCATGAGGGAGAGTTTTCGCCCGTAATG

CMV + CUO | CMV + CUO

9,420 9,430 9,440 9,450 9,460 9,470 9,480 9,490 9,500 9,510 9,520

MscI

TTCTGCGCTAAGATTGTCAGTTTCCAAAAACGAGGAGGATTTGATATTCACCTGGCCCGATCTGGCCATACACTTGAGTGACAATGACATCCACTTTGCCTTTCTCT
AAGACGCGATTCTAACAGTCAAAGGTTTTTGCTCCTCCTAAACTATAAGTGGACCGGGCTAGACCGGTATGTGAACTCACTGTTACTGTAGGTGAAACGGAAAGAGA

CMV + CUO

9,530 9,540 9,550 9,560 9,570 9,580 9,590 9,600 9,610 9,620 9,630

SmaI
BmtI TspMI
XbaI NheI XmaI

CCACAGGTGTCCACTCCCAGGTCCAAGTTTGGTCTAGAGCTAGCGCCACCATGGGGGAGAAACCCGGGACCAGGGTCTTCAAGAAGTCGAGCCCTAACTGCAAGCTC
GGTGTCCACAGGTGAGGGTCCAGGTTCAAACCAGATCTCGATCGCGGTGGTACCCCCTCTTTGGGCCCTGGTCCCAGAAGTTCTTCAGCTCGGGATTGACGTTCGAG

M G E K P G T R V F K K S S P N C K L

CMV + CUO | bArr2

9,640 9,650 9,660 9,670 9,680 9,690 9,700 9,710 9,720 9,730

ACCGTGTACTTGGGCAAGCGGGACTTCGTAGATCACCTGGACAAAGTGGACCCTGTAGATGGCGTGGTGCTTGTGGACCCTGACTACCTGAAGGACCGCAAAGTGTT
TGGCACATGAACCCGTTCGCCCTGAAGCATCTAGTGGACCTGTTTCACCTGGGACATCTACCGCACCACGAACACCTGGGACTGATGGACTTCCTGGCGTTTCACAA

T V Y L G K R D F V D H L D K V D P V D G V V L V D P D Y L K D R K V F

bArr2

9,740 9,750 9,760 9,770 9,780 9,790 9,800 9,810 9,820 9,830 9,840

bArr2-HA-TEV219 pCDH-CuO-MCS-EF1α-CymR-T2A-Bleo3 SparQ (10907 bp)

PaqCI
BspMI
BfuAI

TGTGACCCTCACCTGCGCCTTCCGCTATGGCCGTGAAGACCTGGATGTGCTGGGCTTGTCCTTCCGCAAAGACCTGTTCATCGCCACCTACCAGGCCTTCCCCCCGG
ACACTGGGAGTGGACGCGGAAGGCGATACCGGCACTTCTGGACCTACACGACCCGAACAGGAAGGCGTTTCTGGACAAGTAGCGGTGGATGGTCCGGAAGGGGGGCC
V T L T C A F R Y G R E D L D V L G L S F R K D L F I A T Y Q A F P P

bArr2

9,850 9,860 9,870 9,880 9,890 9,900 9,910 9,920 9,930 9,940 9,950

SbfI BbvCI SphI

TGCCCAACCCACCCCGGCCCCCCACCCGCCTGCAGGACCGGCTGCTGAGGAAGCTGGGCCAGCATGCCCACCCCTTCTTCTTCACCATACCCCAGAATCTTCCATGC
ACGGGTTGGGTGGGGCCGGGGGGTGGGCGGACGTCCTGGCCGACGACTCCTTCGACCCGGTCGTACGGGTGGGGAAGAAGAAGTGGTATGGGGTCTTAGAAGGTACG
V P N P P R P P T R L Q D R L L R K L G Q H A H P F F F T I P Q N L P C

bArr2

9,960 9,970 9,980 9,990 10,000 10,010 10,020 10,030 10,040 10,050

TCCGTCACACTGCAGCCAGGCCCAGAGGATACAGGAAAGGCCTGCGGCGTAGACTTTGAGATTCGAGCCTTCTGTGCTAAATCACTAGAAGAGAAAAGCCACAAAAG
AGGCAGTGTGACGTCGGTCCGGGTCTCCTATGTCCTTTCCGGACGCCGCATCTGAAACTCTAAGCTCGGAAGACACGATTTAGTGATCTTCTCTTTTCGGTGTTTTC
S V T L Q P G P E D T G K A C G V D F E I R A F C A K S L E E K S H K R

bArr2

10,060 10,070 10,080 10,090 10,100 10,110 10,120 10,130 10,140 10,150 10,160

GAACTCTGTGCGGCTGGTGATCCGAAAGGTGCAGTTCGCCCCGGAGAAACCCGGCCCCCAGCCTTCAGCCGAAACCACACGCCACTTCCTCATGTCTGACCGGTCCC
CTTGAGACACGCCGACCACTAGGCTTTCCACGTCAAGCGGGGCCTCTTTGGGCCGGGGGTCGGAAGTCGGCTTTGGTGTGCGGTGAAGGAGTACAGACTGGCCAGGG
N S V R L V I R K V Q F A P E K P G P Q P S A E T T R H F L M S D R S

bArr2

10,170 10,180 10,190 10,200 10,210 10,220 10,230 10,240 10,250 10,260 10,270

PspXI

TGCACCTCGAGGCTTCCCTGGACAAGGAGCTGTACTACCATGGGGAGCCCCTCAATGTAAATGTCCACGTCACCAACAACTCCACCAAGACCGTCAAGAAGATCAAA
ACGTGGAGCTCCGAAGGGACCTGTTCCTCGACATGATGGTACCCCTCGGGGAGTTACATTTACAGGTGCAGTGGTTGTTGAGGTGGTTCTGGCAGTTCTTCTAGTTT
L H L E A S L D K E L Y Y H G E P L N V N V H V T N N S T K T V K K I K

bArr2

10,280 10,290 10,300 10,310 10,320 10,330 10,340 10,350 10,360 10,370

SexAI

GTCTCTGTGAGACAGTACGCCGACATCTGCCTCTTCAGCACCGCCCAGTACAAGTGTCCTGTGGCTCAACTCGAACAAGATGACCAGGTATCTCCCAGCTCCACATT
CAGAGACACTCTGTCATGCGGCTGTAGACGGAGAAGTCGTGGCGGGTCATGTTCACAGGACACCGAGTTGAGCTTGTTCTACTGGTCCATAGAGGGTCGAGGTGTAA
V S V R Q Y A D I C L F S T A Q Y K C P V A Q L E Q D D Q V S P S S T F

bArr2

10,390 10,400 10,410 10,420 10,430 10,440 10,450 10,460 10,470 10,480

bArr2-HA-TEV219 pCDH-CuO-MCS-EF1α-CymR-T2A-Bleo3 SparQ (10907 bp)

BsrGI BlpI

```
CTGTAAGGTGTACACCATAACCCCACTGCTCAGCGACAACCGGGAGAAGCGGGGTCTCGCCCTGGATGGGAAACTCAAGCACGAGGACACCAACCTGGCTTCCAGCA
GACATTCCACATGTGGTATTGGGGTGACGAGTCGCTGTTGGCCCTCTTCGCCCCAGAGCGGGACCTACCCTTTGAGTTCGTGCTCCTGTGGTTGGACCGAAGGTCGT
 C  K  V  Y  T  I  T  P  L  L  S  D  N  R  E  K  R  G  L  A  L  D  G  K  L  K  H  E  D  T  N  L  A  S  S
```

bArr2

10,490 10,500 10,510 10,520 10,530 10,540 10,550 10,560 10,570 10,580 10,590

```
CCATCGTGAAGGAGGGTGCCAACAAGGAGGTGCTGGGAATCCTGGTGTCCTACAGGGTCAAGGTGAAGCTGGTGGTGTCTCGAGGCGGGGATGTCTCTGTGGAGCTG
GGTAGCACTTCCTCCCACGGTTGTTCCTCCACGACCCTTAGGACCACAGGATGTCCCAGTTCCACTTCGACCACCACAGAGCTCCGCCCCTACAGAGACACCTCGAC
T  I  V  K  E  G  A  N  K  E  V  L  G  I  L  V  S  Y  R  V  K  V  K  L  V  V  S  R  G  G  D  V  S  V  E  L
```

bArr2

10,600 10,610 10,620 10,630 10,640 10,650 10,660 10,670 10,680 10,690 10,700

```
CCTTTTGTTCTTATGCACCCCAAGCCCCACGACCACATCCCCCTCCCCAGACCCCAGTCAGCCGCTCCGGAGACACAGATGTCCCTGTGGACACCAACCTCATTGAATT
GGAAAACAAGAATACGTGGGGTTCGGGGTGCTGGTGTAGGGGGAGGGGTCTGGGGTCAGTCGGCGAGGCCTCTGTCTACAGGGACACCTGTGGTTGGAGTAACTTAA
P  F  V  L  M  H  P  K  P  H  D  H  I  P  L  P  R  P  Q  S  A  A  P  E  T  D  V  P  V  D  T  N  L  I  E  F
```

bArr2

10,710 10,720 10,730 10,740 10,750 10,760 10,770 10,780 10,790 10,800

BclI

```
TGATACCAACTATGCCACAGATGATGACATTGTGTTTGAGGACTTTGCCCGGCTTCGGCTGAAGGGGATGAAGGATGACGACTATGATGATCAACTCTGC
ACTATGGTTGATACGGTGTCTACTACTGTAACACAAACTCCTGAAACGGGCCGAAGCCGACTTCCCCTACTTCCTACTGCTGATACTACTAGTTGAGACG
 D  T  N  Y  A  T  D  D  D  I  V  F  E  D  F  A  R  L  R  L  K  G  M  K  D  D  D  Y  D  D  Q  L  C
```

bArr2

10,810 10,820 10,830 10,840 10,850 10,860 10,870 10,880 10,890 10,900

(from 1-749 bp)

Fyve-HA-TEV219 pCDH-CuO-MCS-EF1α-CymR-T2A-Bleo3...

SwaI NotI AsiSI MfeI

ATTTAAATCGGATCCGCGGCCGCGGATCTGCGATCGCTCCGGTGCCCGTCAGTGGGCAGAGCGCACATCGCCCACAGTCCCCGAGAAGTTGGGGGGAGGGGTCGGCA
TAAATTTAGCCTAGGCGCCGGCGCCTAGACGCTAGCGAGGCCACGGGCAGTCACCCGTCTCGCGTGTAGCGGGTGTCAGGGGCTCTTCAACCCCCCTCCCCAGCCGT

EF1 alpha

10 20 30 40 50 60 70 80 90 100

ATTGAACGGGTGCCTAGAGAAGGTGGCGCGGGGTAAACTGGGAAAGTGATGTCGTGTACTGGCTCCGCCTTTTTCCCGAGGGTGGGGGAGAACCGTATATAAGTGCA
TAACTTGCCCACGGATCTCTTCCACCGCGCCCCATTTGACCCTTTCACTACAGCACATGACCGAGGCGGAAAAAGGGCTCCCACCCCCTCTTGGCATATATTCACGT

EF1 alpha

110 120 130 140 150 160 170 180 190 200 210

Bsu36I

GTAGTCGCCGTGAACGTTCTTTTTCGCAACGGGTTTGCCGCCAGAACACAGCTGAAGCTTCGAGGGGCTCGCATCTCTCCTTCACGCGCCCGCCGCCCTACCTGAGG
CATCAGCGGCACTTGCAAGAAAAAGCGTTGCCCAAACGGCGGTCTTGTGTCGACTTCGAAGCTCCCCGAGCGTAGAGAGGAAGTGCGCGGGCGGCGGGATGGACTCC

EF1 alpha

220 230 240 250 260 270 280 290 300 310 320

CspCI

CCGCCATCCACGCCGGTTGAGTCGCGTTCTGCCGCCTCCCGCCTGTGGTGCCTCCTGAACTGCGTCCGCCGTCTAGGTAAGTTTAAAGCTCAGGTCGAGACCGGGCC
GGCGGTAGGTGCGGCCAACTCAGCGCAAGACGGCGGAGGGCGGACACCACGGAGGACTTGACGCAGGCGGCAGATCCATTCAAATTTCGAGTCCAGCTCTGGCCCGG

EF1 alpha

330 340 350 360 370 380 390 400 410 420

TTTGTCCGGCGCTCCCTTGGAGCCTACCTAGACTCAGCCGGCTCTCCACGCTTTGCCTGACCCTGCTTGCTCAACTCTACGTCTTTGTTTCGTTTTCTGTTCTGCGC
AAACAGGCCGCGAGGGAACCTCGGATGGATCTGAGTCGGCCGAGAGGTGCGAAACGGACTGGGACGAACGAGTTGAGATGCAGAAACAAAGCAAAAGACAAGACGCG

EF1 alpha

430 440 450 460 470 480 490 500 510 520 530

CGTTACAGATCCAAGCTGTGACCGGCGCCTACTCTAGAGCCGCCATGGCTTACCCATACGATGTTCCAGATTACGCTAGTTTGGTGAGGCAGCAGAGACCGATGACA
GCAATGTCTAGGTTCGACACTGGCCGCGGATGAGATCTCGGCGGTACCGAATGGGTATGCTACAAGGTCTAATGCGATCAAACCACTCCGTCGTCTCTGGCTACTGT

M A Y P Y D V P D Y A S L V R Q Q R P M T

EF1 alpha HA

540 550 560 570 580 590 600 610 620 630 640

BclI

TCCACTTTGCCTTTCTCTCCACAGGTGTCCACTCCCAGGTCCAAGTTTGGAAGATCTACCATGCCAAAGAGACCCAGACCCGTGATCATGAGTCCAAAGAGAAGAAC
AGGTGAAACGGAAAGAGAGGTGTCCACAGGTGAGGGTCCAGGTTCAAACCTTCTAGATGGTACGGTTTCTCTGGGTCTGGGCACTAGTACTCAGGTTTCTCTTCTTG

S T L P F S P Q V S T P R S K F G R S T M P K R P R P V I M S P K R R T

CymR

650 660 670 680 690 700 710 720 730 740

Fyve-HA-TEV219 pCDH-CuO-MCS-EF1α-CymR-T2A-Bleo3 SparQ (10070 bp)

PaqCI BspMI BfuAI (~851); PasI (~775); PasI (~799); BspEI (~834)

```
ACAGGCAGAGCGCGCAATGGAGACCCAGGGCAAGTTGATTGCAGCGGCCCTGGGGGTTTTACGGGAAAAAGGTTACGCGGGATTCCGGATCGCAGATGTGCCCGGTG
TGTCCGTCTCGCGCGTTACCTCTGGGTCCCGTTCAACTAACGTCGCCGGGACCCCCAAAATGCCCTTTTTCCAATGCGCCCTAAGGCCTAGCGTCTACACGGGCCAC
 Q  A  E  R  A  M  E  T  Q  G  K  L  I  A  A  A  L  G  V  L  R  E  K  G  Y  A  G  F  R  I  A  D  V  P  G
```

CymR

750 760 770 780 790 800 810 820 830 840 850

PstI; PaeR7I XhoI TliI

```
CTGCAGGTGTCTCGAGAGGAGCGCAGAGCCATCATTTCCCGACAAAGCTTGAGCTTCTGCTTGCCACTTTTGAATGGCTTTACGAACAGATCACCGAACGCAGTCGG
GACGTCCACAGAGCTCTCCTCGCGTCTCGGTAGTAAAGGGCTGTTTCGAACTCGAAGACGAACGGTGAAAACTTACCGAAATGCTTGTCTAGTGGCTTGCGTCAGCC
 A  A  G  V  S  R  G  A  Q  S  H  H  F  P  T  K  L  E  L  L  L  A  T  F  E  W  L  Y  E  Q  I  T  E  R  S  R
```

CymR

860 870 880 890 900 910 920 930 940 950 960

```
GCTCGATTAGCGAAATTGAAGCCAGAGGATGACGTCATCCAGCAAATGCTGGACGACGCCGCCGAATTTTTCCTCGACGATGACTTCTCTATCAGCCTTGATTTGAT
CGAGCTAATCGCTTTAACTTCGGTCTCCTACTGCAGTAGGTCGTTTACGACCTGCTGCGGCGGCTTAAAAAGGAGCTGCTACTGAAGAGATAGTCGGAACTAAACTA
 A  R  L  A  K  L  K  P  E  D  D  V  I  Q  Q  M  L  D  D  A  A  E  F  F  L  D  D  D  F  S  I  S  L  D  L  I
```

CymR

970 980 990 1,000 1,010 1,020 1,030 1,040 1,050 1,060 1,070

```
TGTGGCTGCCGACCGGGATCCAGCGTTACGCGAGGGTATTCAGCGCACGGTAGAGAGGAATCGGTTTGTCGTCGAGGATATGTGGCTTGGTGTTCTGGTGAGCCGTG
ACACCGACGGCTGGCCCTAGGTCGCAATGCGCTCCCATAAGTCGCGTGCCATCTCTCCTTAGCCAAACAGCAGCTCCTATACACCGAACCACAAGACCACTCGGCAC
 V  A  A  D  R  D  P  A  L  R  E  G  I  Q  R  T  V  E  R  N  R  F  V  V  E  D  M  W  L  G  V  L  V  S  R
```

CymR

1,080 1,090 1,100 1,110 1,120 1,130 1,140 1,150 1,160 1,170

EcoRV

```
GTCTTTCGCGTGATGATGCAGAAGATATCCTTTGGTTGATATTCAATTCGGTGCGTGGGCTTGCTGTTCGTAGCCTATGGCAGAAGGACAAAGAACGCTTTGAGCGT
CAGAAAGCGCACTACTACGTCTTCTATAGGAAACCAACTATAAGTTAAGCCACGCACCCGAACGACAAGCATCGGATACCGTCTTCCTGTTTCTTGCGAAACTCGCA
 G L  S  R  D  D  A  E  D  I  L  W  L  I  F  N  S  V  R  G  L  A  V  R  S  L  W  Q  K  D  K  E  R  F  E  R
```

CymR

1,180 1,190 1,200 1,210 1,220 1,230 1,240 1,250 1,260 1,270 1,280

```
GTCAGGAACTCGACACTCGAAATTGCGCGAGAGCGGTACGCGAAATTCAAGCGCGCGGCCAAACCCTCTGGCCGGTCTCTCTCTGTTCGAGAGGGCAGAGGAAGTCT
CAGTCCTTGAGCTGTGAGCTTTAACGCGCTCTCGCCATGCGCTTTAAGTTCGCGCGCCGGTTTGGGAGACCGGCCAGAGAGAGACAAGCTCTCCCGTCTCCTTCAGA
 V  R  N  S  T  L  E  I  A  R  E  R  Y  A  K  F  K  R  A  A  K  P  S  G
```

CymR   T2A

1,290 1,300 1,310 1,320 1,330 1,340 1,350 1,360 1,370 1,380 1,390

Fyve-HA-TEV219 pCDH-CuO-MCS-EF1α-CymR-T2A-Bleo3 SparQ (10070 bp)

BmgBI EcoRI MscI MauBI

```
TCTAACATGCGGTGACGTGGAGGAGAATCCCGGCCCTGAATTCATGGCCAAGTTGACCAGTGCCGTTCCGGTGCTCACCGCGCGCGACGTCGCCGGAGCGGTCGAGT
AGATTGTACGCCACTGCACCTCCTCTTAGGGCCGGGACTTAAGTACCGGTTCAACTGGTCACGGCAAGGCCACGAGTGGCGCGCGCTGCAGCGGCCTCGCCAGCTCA
```

M A K L T S A V P V L T A R D V A G A V E

T2A bleo

1,400 1,410 1,420 1,430 1,440 1,450 1,460 1,470 1,480 1,490

SmaI TspMI XmaI SgrAI BmgBI SexAI

```
TCTGGACCGACCGGCTCGGGTTCTCCCGGGACTTCGTGGAGGACGACTTCGCCGGTGTGGTCCGGGACGACGTGACCCTGTTCATCAGCGCGGTCCAGGACCAGGTG
AGACCTGGCTGGCCGAGCCCAAGAGGGCCCTGAAGCACCTCCTGCTGAAGCGGCCACACCAGGCCCTGCTGCACTGGGACAAGTAGTCGCGCCAGGTCCTGGTCCAC
```

F W T D R L G F S R D F V E D D F A G V V R D D V T L F I S A V Q D Q V

bleo

1,500 1,510 1,520 1,530 1,540 1,550 1,560 1,570 1,580 1,590 1,600

FseI

```
GTGCCGGACAACACCCTGGCCTGGGTGTGGGTGCGCGGCCTGGACGAGCTGTACGCCGAGTGGTCGGAGGTCGTGTCCACGAACTTCCGGGACGCCTCCGGGCCGGC
CACGGCCTGTTGTGGGACCGGACCCACACCCACGCGCCGGACCTGCTCGACATGCGGCTCACCAGCCTCCAGCACAGGTGCTTGAAGGCCCTGCGGAGGCCCGGCCG
```

V P D N T L A W V W V R G L D E L Y A E W S E V V S T N F R D A S G P A

bleo

1,610 1,620 1,630 1,640 1,650 1,660 1,670 1,680 1,690 1,700 1,710

SalI Tth111I DraIII PflFI

```
CATGACCGAGATCGGCGAGCAGCCGTGGGGGCGGGAGTTCGCCCTGCGCGACCCGGCCGGCAACTGCGTGCACTTCGTGGCCGAGGAGCAGGACTGAGTCGACTCGA
GTACTGGCTCTAGCCGCTCGTCGGCACCCCCGCCCTCAAGCGGGACGCGCTGGGCCGGCCGTTGACGCACGTGAAGCACCGGCTCCTCGTCCTGACTCAGCTGAGCT
```

M T E I G E Q P W G R E F A L R D P A G N C V H F V A E E Q D *

bleo

1,720 1,730 1,740 1,750 1,760 1,770 1,780 1,790 1,800 1,810

```
CAATCAACCTCTGGATTACAAAATTTGTGAAAGATTGACTGGTATTCTTAACTATGTTGCTCCTTTTACGCTATGTGGATACGCTGCTTTAATGCCTTTGTATCATG
GTTAGTTGGAGACCTAATGTTTTAAACACTTTCTAACTGACCATAAGAATTGATACAACGAGGAAAATGCGATACACCTATGCGACGAAATTACGGAAACATAGTAC
```

WPRE

1,820 1,830 1,840 1,850 1,860 1,870 1,880 1,890 1,900 1,910 1,920

```
CTATTGCTTCCCGTATGGCTTTCATTTTCTCCTCCTTGTATAAATCCTGGTTGCTGTCTCTTTATGAGGAGTTGTGGCCCGTTGTCAGGCAACGTGGCGTGGTGTGC
GATAACGAAGGGCATACCGAAAGTAAAAGAGGAGGAACATATTTAGGACCAACGACAGAGAAATACTCCTCAACACCGGGCAACAGTCCGTTGCACCGCACCACACG
```

WPRE

1,930 1,940 1,950 1,960 1,970 1,980 1,990 2,000 2,010 2,020 2,030

Fyve-HA-TEV219 pCDH-CuO-MCS-EF1α-CymR-T2A-Bleo3 SparQ (10070 bp)

ACTGTGTTTGCTGACGCAACCCCCACTGGTTGGGGCATTGCCACCACCTGTCAGCTCCTTTCCGGGACTTTCGCTTTCCCCCTCCCTATTGCCACGGCGGAACTCAT
TGACACAAACGACTGCGTTGGGGGTGACCAACCCCGTAACGGTGGTGGACAGTCGAGGAAAGGCCCTGAAAGCGAAAGGGGGAGGGATAACGGTGCCGCCTTGAGTA

WPRE

2,040 2,050 2,060 2,070 2,080 2,090 2,100 2,110 2,120 2,130 2,140

CGCCGCCTGCCTTGCCCGCTGCTGGACAGGGGCTCGGCTGTTGGGCACTGACAATTCCGTGGTGTTGTCGGGGAAATCATCGTCCTTTCCTTGGCTGCTCGCCTGTG
GCGGCGGACGGAACGGGCGACGACCTGTCCCCGAGCCGACAACCCGTGACTGTTAAGGCACCACAACAGCCCCTTTAGTAGCAGGAAAGGAACCGACGAGCGGACAC

WPRE

2,150 2,160 2,170 2,180 2,190 2,200 2,210 2,220 2,230 2,240

TTGCCACCTGGATTCTGCGCGGGACGTCCTTCTGCTACGTCCCTTCGGCCCTCAATCCAGCGGACCTTCCTTCCCGCGGCCTGCTGCCGGCTCTGCGGCCTCTTCCG
AACGGTGGACCTAAGACGCGCCCTGCAGGAAGACGATGCAGGGAAGCCGGGAGTTAGGTCGCCTGGAAGGAAGGGCGCCGGACGACGGCCGAGACGCCGGAGAAGGC

WPRE

2,250 2,260 2,270 2,280 2,290 2,300 2,310 2,320 2,330 2,340 2,350

KpnI
Acc65I

CGTCTTCGCCTTCGCCCTCAGACGAGTCGGATCTCCCTTTGGGCCGCCTCCCCGCCTGGTACCTTTAAGACCAATGACTTACAAGGCAGCTGTAGATCTTAGCCACT
GCAGAAGCGGAAGCGGGAGTCTGCTCAGCCTAGAGGGAAACCCGGCGGAGGGGCGGACCATGGAAATTCTGGTTACTGAATGTTCCGTCGACATCTAGAATCGGTGA

WPRE

2,360 2,370 2,380 2,390 2,400 2,410 2,420 2,430 2,440 2,450 2,460

TTTTAAAAGAAAAGGGGGGACTGGAAGGGCTAATTCACTCCCAACGAAGATAAGATCTGCTTTTTGCTTGTACTGGGTCTCTCTGGTTAGACCAGATCTGAGCCTGG
AAAATTTTCTTTTCCCCCCTGACCTTCCCGATTAAGTGAGGGTTGCTTCTATTCTAGACGAAAAACGAACATGACCCAGAGAGACCAATCTGGTCTAGACTCGGACC

3’LTR

2,470 2,480 2,490 2,500 2,510 2,520 2,530 2,540 2,550 2,560

GAGCTCTCTGGCTAACTAGGGAACCCACTGCTTAAGCCTCAATAAAGCTTGCCTTGAGTGCTTCAAGTAGTGTGTGCCCGTCTGTTGTGTGACTCTGGTAACTAGAG
CTCGAGAGACCGATTGATCCCTTGGGTGACGAATTCGGAGTTATTTCGAACGGAACTCACGAAGTTCATCACACACGGGCAGACAACACACTGAGACCATTGATCTC

3’LTR

2,570 2,580 2,590 2,600 2,610 2,620 2,630 2,640 2,650 2,660 2,670

ATCCCTCAGACCCTTTTAGTCAGTGTGGAAAATCTCTAGCAGTAGTAGTTCATGTCATCTTATTATTCAGTATTTATAACTTGCAAAGAAATGAATATCAGAGAGTG
TAGGGAGTCTGGGAAAATCAGTCACACCTTTTAGAGATCGTCATCATCAAGTACAGTAGAATAATAAGTCATAAATATTGAACGTTTCTTTACTTATAGTCTCTCAC

3’LTR

2,680 2,690 2,700 2,710 2,720 2,730 2,740 2,750 2,760 2,770 2,780

AGAGGAACTTGTTTATTGCAGCTTATAATGGTTACAAATAAAGCAATAGCATCACAAATTTCACAAATAAAGCATTTTTTTCACTGCATTCTAGTTGTGGTTTGTCC
TCTCCTTGAACAAATAACGTCGAATATTACCAATGTTTATTTCGTTATCGTAGTGTTTAAAGTGTTTATTTCGTAAAAAAAGTGACGTAAGATCAACACCAAACAGG

SV40 Polyadenylation signal

2,790 2,800 2,810 2,820 2,830 2,840 2,850 2,860 2,870 2,880

Fyve-HA-TEV219 pCDH-CuO-MCS-EF1α-CymR-T2A-Bleo3 SparQ (10070 bp)

AAACTCATCAATGTATCTTATCATGTCTGGCTCTAGCTATCCCGCCCCTAACTCCGCCCATCCCGCCCCTAACTCCGCCCAGTTCCGCCCATTCTCCGCCCCATGGC
TTTGAGTAGTTACATAGAATAGTACAGACCGAGATCGATAGGGCGGGGATTGAGGCGGGTAGGGCGGGGATTGAGGCGGGTCAAGGCGGGTAAGAGGCGGGGTACCG
SV40 Polyade...tion signal   SV40 Ori
2,890 2,900 2,910 2,920 2,930 2,940 2,950 2,960 2,970 2,980 2,990

SfiI   StuI
TGACTAATTTTTTTTATTTATGCAGAGGCCGAGGCCGCCTCGGCCTCTGAGCTATTCCAGAAGTAGTGAGGAGGCTTTTTTGGAGGCCTAGACTTTTGCAGAGACCA
ACTGATTAAAAAAAATAAATACGTCTCCGGCTCCGGCGGAGCCGGAGACTCGATAAGGTCTTCATCACTCCTCCGAAAAAACCTCCGGATCTGAAAACGTCTCTGGT
SV40 Ori
3,000 3,010 3,020 3,030 3,040 3,050 3,060 3,070 3,080 3,090 3,100

AATTCGTAATCATGTCATAGCTGTTTCCTGTGTGAAATTGTTATCCGCTCACAATTCCACACAACATACGAGCCGGAAGCATAAAGTGTAAAGCCTGGGGTGCCTAA
TTAAGCATTAGTACAGTATCGACAAAGGACACACTTTAACAATAGGCGAGTGTTAAGGTGTGTTGTATGCTCGGCCTTCGTATTTCACATTTCGGACCCCACGGATT
3,110 3,120 3,130 3,140 3,150 3,160 3,170 3,180 3,190 3,200 3,210

TGAGTGAGCTAACTCACATTAATTGCGTTGCGCTCACTGCCCGCTTTCCAGTCGGGAAACCTGTCGTGCCAGCTGCATTAATGAATCGGCCAACGCGCGGGGAGAGG
ACTCACTCGATTGAGTGTAATTAACGCAACGCGAGTGACGGGCGAAAGGTCAGCCCTTTGGACAGCACGGTCGACGTAATTACTTAGCCGGTTGCGCGCCCCTCTCC
3,220 3,230 3,240 3,250 3,260 3,270 3,280 3,290 3,300 3,310

BspQI
SapI
CGGTTTGCGTATTGGGCGCTCTTCCGCTTCCTCGCTCACTGACTCGCTGCGCTCGGTCGTTCGGCTGCGGCGAGCGGTATCAGCTCACTCAAAGGCGGTAATACGGT
GCCAAACGCATAACCCGCGAGAAGGCGAAGGAGCGAGTGACTGAGCGACGCGAGCCAGCAAGCCGACGCCGCTCGCCATAGTCGAGTGAGTTTCCGCCATTATGCCA
3,320 3,330 3,340 3,350 3,360 3,370 3,380 3,390 3,400 3,410 3,420

AflIII
PciI
TATCCACAGAATCAGGGGATAACGCAGGAAAGAACATGTGAGCAAAAGGCCAGCAAAAGGCCAGGAACCGTAAAAAGGCCGCGTTGCTGGCGTTTTTCCATAGGCTC
ATAGGTGTCTTAGTCCCCTATTGCGTCCTTTCTTGTACACTCGTTTTCCGGTCGTTTTCCGGTCCTTGGCATTTTTCCGGCGCAACGACCGCAAAAAGGTATCCGAG
pUC ori
3,430 3,440 3,450 3,460 3,470 3,480 3,490 3,500 3,510 3,520 3,530

CGCCCCCCTGACGAGCATCACAAAAATCGACGCTCAAGTCAGAGGTGGCGAAACCCGACAGGACTATAAAGATACCAGGCGTTTCCCCCTGGAAGCTCCCTCGTGCG
GCGGGGGGACTGCTCGTAGTGTTTTTAGCTGCGAGTTCAGTCTCCACCGCTTTGGGCTGTCCTGATATTTCTATGGTCCGCAAAGGGGGACCTTCGAGGGAGCACGC
pUC ori
3,540 3,550 3,560 3,570 3,580 3,590 3,600 3,610 3,620 3,630

CTCTCCTGTTCCGACCCTGCCGCTTACCGGATACCTGTCCGCCTTTCTCCCTTCGGGAAGCGTGGCGCTTTCTCATAGCTCACGCTGTAGGTATCTCAGTTCGGTGT
GAGAGGACAAGGCTGGGACGGCGAATGGCCTATGGACAGGCGGAAAGAGGGAAGCCCTTCGCACCGCGAAAGAGTATCGAGTGCGACATCCATAGAGTCAAGCCACA
pUC ori
3,640 3,650 3,660 3,670 3,680 3,690 3,700 3,710 3,720 3,730 3,740

Fyve-HA-TEV219 pCDH-CuO-MCS-EF1α-CymR-T2A-Bleo3 SparQ (10070 bp)

BseYI

AGGTCGTTCGCTCCAAGCTGGGCTGTGTGCACGAACCCCCCGTTCAGCCCGACCGCTGCGCCTTATCCGGTAACTATCGTCTTGAGTCCAACCCGGTAAGACACGAC
TCCAGCAAGCGAGGTTCGACCCGACACACGTGCTTGGGGGGCAAGTCGGGCTGGCGACGCGGAATAGGCCATTGATAGCAGAACTCAGGTTGGGCCATTCTGTGCTG

pUC ori

3,750 3,760 3,770 3,780 3,790 3,800 3,810 3,820 3,830 3,840 3,850

TTATCGCCACTGGCAGCAGCCACTGGTAACAGGATTAGCAGAGCGAGGTATGTAGGCGGTGCTACAGAGTTCTTGAAGTGGTGGCCTAACTACGGCTACACTAGAAG
AATAGCGGTGACCGTCGTCGGTGACCATTGTCCTAATCGTCTCGCTCCATACATCCGCCACGATGTCTCAAGAACTTCACCACCGGATTGATGCCGATGTGATCTTC

pUC ori

3,860 3,870 3,880 3,890 3,900 3,910 3,920 3,930 3,940 3,950

GACAGTATTTGGTATCTGCGCTCTGCTGAAGCCAGTTACCTTCGGAAAAAGAGTTGGTAGCTCTTGATCCGGCAAACAAACCACCGCTGGTAGCGGTGGTTTTTTTG
CTGTCATAAACCATAGACGCGAGACGACTTCGGTCAATGGAAGCCTTTTTCTCAACCATCGAGAACTAGGCCGTTTGTTTGGTGGCGACCATCGCCACCAAAAAAAC

pUC ori

3,960 3,970 3,980 3,990 4,000 4,010 4,020 4,030 4,040 4,050 4,060

TTTGCAAGCAGCAGATTACGCGCAGAAAAAAAGGATCTCAAGAAGATCCTTTGATCTTTTCTACGGGGTCTGACGCTCAGTGGAACGAAAACTCACGTTAAGGGATT
AAACGTTCGTCGTCTAATGCGCGTCTTTTTTTCCTAGAGTTCTTCTAGGAAACTAGAAAAGATGCCCCAGACTGCGAGTCACCTTGCTTTTGAGTGCAATTCCCTAA

pUC ori

4,070 4,080 4,090 4,100 4,110 4,120 4,130 4,140 4,150 4,160 4,170

TTGGTCATGAGATTATCAAAAAGGATCTTCACCTAGATCCTTTTAAATTAAAAATGAAGTTTTAAATCAATCTAAAGTATATATGAGTAAACTTGGTCTGACAGTTA
AACCAGTACTCTAATAGTTTTTCCTAGAAGTGGATCTAGGAAAATTTAATTTTTACTTCAAAATTTAGTTAGATTTCATATATACTCATTTGAACCAGACTGTCAAT

4,180 4,190 4,200 4,210 4,220 4,230 4,240 4,250 4,260 4,270 4,280

AhdI

CCAATGCTTAATCAGTGAGGCACCTATCTCAGCGATCTGTCTATTTCGTTCATCCATAGTTGCCTGACTCCCCGTCGTGTAGATAACTACGATACGGGAGGGCTTAC
GGTTACGAATTAGTCACTCCGTGGATAGAGTCGCTAGACAGATAAAGCAAGTAGGTATCAACGGACTGAGGGGCAGCACATCTATTGATGCTATGCCCTCCCGAATG

AmpR

4,290 4,300 4,310 4,320 4,330 4,340 4,350 4,360 4,370 4,380

CATCTGGCCCCAGTGCTGCAATGATACCGCGAGACCCACGCTCACCGGCTCCAGATTTATCAGCAATAAACCAGCCAGCCGGAAGGGCCGAGCGCAGAAGTGGTCCT
GTAGACCGGGGTCACGACGTTACTATGGCGCTCTGGGTGCGAGTGGCCGAGGTCTAAATAGTCGTTATTTGGTCGGTCGGCCTTCCCGGCTCGCGTCTTCACCAGGA

AmpR

4,390 4,400 4,410 4,420 4,430 4,440 4,450 4,460 4,470 4,480 4,490

FspI

GCAACTTTATCCGCCTCCATCCAGTCTATTAATTGTTGCCGGGAAGCTAGAGTAAGTAGTTCGCCAGTTAATAGTTTGCGCAACGTTGTTGCCATTGCTACAGGCAT
CGTTGAAATAGGCGGAGGTAGGTCAGATAATTAACAACGGCCCTTCGATCTCATTCATCAAGCGGTCAATTATCAAACGCGTTGCAACAACGGTAACGATGTCCGTA

AmpR

4,500 4,510 4,520 4,530 4,540 4,550 4,560 4,570 4,580 4,590 4,600

Fyve-HA-TEV219 pCDH-CuO-MCS-EF1α-CymR-T2A-Bleo3 SparQ (10070 bp)

CGTGGTGTCACGCTCGTCGTTTGGTATGGCTTCATTCAGCTCCGGTTCCCAACGATCAAGGCGAGTTACATGATCCCCCATGTTGTGCAAAAAAGCGGTTAGCTCCT
GCACCACAGTGCGAGCAGCAAACCATACCGAAGTAAGTCGAGGCCAAGGGTTGCTAGTTCCGCTCAATGTACTAGGGGGTACAACACGTTTTTTCGCCAATCGAGGA

AmpR

4,610 4,620 4,630 4,640 4,650 4,660 4,670 4,680 4,690 4,700

TCGGTCCTCCGATCGTTGTCAGAAGTAAGTTGGCCGCAGTGTTATCACTCATGGTTATGGCAGCACTGCATAATTCTCTTACTGTCATGCCATCCGTAAGATGCTTT
AGCCAGGAGGCTAGCAACAGTCTTCATTCAACCGGCGTCACAATAGTGAGTACCAATACCGTCGTGACGTATTAAGAGAATGACAGTACGGTAGGCATTCTACGAAA

AmpR

4,710 4,720 4,730 4,740 4,750 4,760 4,770 4,780 4,790 4,800 4,810

TCTGTGACTGGTGAGTACTCAACCAAGTCATTCTGAGAATAGTGTATGCGGCGACCGAGTTGCTCTTGCCCGGCGTCAATACGGGATAATACCGCGCCACATAGCAG
AGACACTGACCACTCATGAGTTGGTTCAGTAAGACTCTTATCACATACGCCGCTGGCTCAACGAGAACGGGCCGCAGTTATGCCCTATTATGGCGCGGTGTATCGTC

AmpR

4,820 4,830 4,840 4,850 4,860 4,870 4,880 4,890 4,900 4,910 4,920

XmnI

AACTTTAAAAGTGCTCATCATTGGAAAACGTTCTTCGGGGCGAAAACTCTCAAGGATCTTACCGCTGTTGAGATCCAGTTCGATGTAACCCACTCGTGCACCCAACT
TTGAAATTTTCACGAGTAGTAACCTTTTGCAAGAAGCCCCGCTTTTGAGAGTTCCTAGAATGGCGACAACTCTAGGTCAAGCTACATTGGGTGAGCACGTGGGTTGA

AmpR

4,930 4,940 4,950 4,960 4,970 4,980 4,990 5,000 5,010 5,020

GATCTTCAGCATCTTTTACTTTCACCAGCGTTTCTGGGTGAGCAAAAACAGGAAGGCAAAATGCCGCAAAAAAGGGAATAAGGGCGACACGGAAATGTTGAATACTC
CTAGAAGTCGTAGAAAATGAAAGTGGTCGCAAAGACCCACTCGTTTTTGTCCTTCCGTTTTACGGCGTTTTTTCCCTTATTCCCGCTGTGCCTTTACAACTTATGAG

AmpR

5,030 5,040 5,050 5,060 5,070 5,080 5,090 5,100 5,110 5,120 5,130

SspI

ATACTCTTCCTTTTTCAATATTATTGAAGCATTTATCAGGGTTATTGTCTCATGAGCGGATACATATTTGAATGTATTTAGAAAAATAAACAAATAGGGGTTCCGCG
TATGAGAAGGAAAAAGTTATAATAACTTCGTAAATAGTCCCAATAACAGAGTACTCGCCTATGTATAAACTTACATAAATCTTTTTATTTGTTTATCCCCAAGGCGC

5,140 5,150 5,160 5,170 5,180 5,190 5,200 5,210 5,220 5,230 5,240

CACATTTCCCCGAAAAGTGCCACCTGACGTCTAAGAAACCATTATTATCATGACATTAACCTATAAAAATAGGCGTATCACGAGGCCCTTTCGTCTCGCGCGTTTCG
GTGTAAAGGGGCTTTTCACGGTGGACTGCAGATTCTTTGGTAATAATAGTACTGTAATTGGATATTTTTATCCGCATAGTGCTCCGGGAAAGCAGAGCGCGCAAAGC

5,250 5,260 5,270 5,280 5,290 5,300 5,310 5,320 5,330 5,340 5,350

GTGATGACGGTGAAAACCTCTGACACATGCAGCTCCCGGAGACGGTCACAGCTTGTCTGTAAGCGGATGCCGGGAGCAGACAAGCCCGTCAGGGCGCGTCAGCGGGT
CACTACTGCCACTTTTGGAGACTGTGTACGTCGAGGGCCTCTGCCAGTGTCGAACAGACATTCGCCTACGGCCCTCGTCTGTTCGGGCAGTCCCGCGCAGTCGCCCA

5,360 5,370 5,380 5,390 5,400 5,410 5,420 5,430 5,440 5,450

BstAPI
NdeI

GTTGGCGGGTGTCGGGGCTGGCTTAACTATGCGGCATCAGAGCAGATTGTACTGAGAGTGCACCATATGCGGTGTGAAATACCGCACAGATGCGTAAGGAGAAAATA
CAACCGCCCACAGCCCCGACCGAATTGATACGCCGTAGTCTCGTCTAACATGACTCTCACGTGGTATACGCCACACTTTATGGCGTGTCTACGCATTCCTCTTTTAT

5,460 5,470 5,480 5,490 5,500 5,510 5,520 5,530 5,540 5,550 5,560

FspI

CCGCATCAGGCGCCATTCGCCATTCAGGCTGCGCAACTGTTGGGAAGGGCGATCGGTGCGGGCCTCTTCGCTATTACGCCAGCTGGCGAAAGGGGGATGTGCTGCAA
GGCGTAGTCCGCGGTAAGCGGTAAGTCCGACGCGTTGACAACCCTTCCCGCTAGCCACGCCCGGAGAAGCGATAATGCGGTCGACCGCTTTCCCCCTACACGACGTT

5,570 5,580 5,590 5,600 5,610 5,620 5,630 5,640 5,650 5,660 5,670

AflIII
MluI

GGCGATTAAGTTGGGTAACGCCAGGGTTTTCCCAGTCACGACGTTGTAAAACGACGGCCAGTGCCAAGCTGACGCGTGTAGTCTTATGCAATACTCTTGTAGTCTTG
CCGCTAATTCAACCCATTGCGGTCCCAAAAGGGTCAGTGCTGCAACATTTTGCTGCCGGTCACGGTTCGACTGCGCACATCAGAATACGTTATGAGAACATCAGAAC

RSV

5,680 5,690 5,700 5,710 5,720 5,730 5,740 5,750 5,760 5,770

SphI BaeI

CAACATGGTAACGATGAGTTAGCAACATGCCTTACAAGGAGAGAAAAAGCACCGTGCATGCCGATTGGTGGAAGTAAGGTGGTACGATCGTGCCTTATTAGGAAGGC
GTTGTACCATTGCTACTCAATCGTTGTACGGAATGTTCCTCTCTTTTTCGTGGCACGTACGGCTAACCACCTTCATTCCACCATGCTAGCACGGAATAATCCTTCCG

RSV

5,780 5,790 5,800 5,810 5,820 5,830 5,840 5,850 5,860 5,870 5,880

AACAGACGGGTCTGACATGGATTGGACGAACCACTGAATTGCCGCATTGCAGAGATATTGTATTTAAGTGCCTAGCTCGATACATAAACGGGTCTCTCTGGTTAGAC
TTGTCTGCCCAGACTGTACCTAACCTGCTTGGTGACTTAACGGCGTAACGTCTCTATAACATAAATTCACGGATCGAGCTATGTATTTGCCCAGAGAGACCAATCTG

RSV HIV LTR

5,890 5,900 5,910 5,920 5,930 5,940 5,950 5,960 5,970 5,980 5,990

CAGATCTGAGCCTGGGAGCTCTCTGGCTAACTAGGGAACCCACTGCTTAAGCCTCAATAAAGCTTGCCTTGAGTGCTTCAAGTAGTGTGTGCCCGTCTGTTGTGTGA
GTCTAGACTCGGACCCTCGAGAGACCGATTGATCCCTTGGGTGACGAATTCGGAGTTATTTCGAACGGAACTCACGAAGTTCATCACACACGGGCAGACAACACACT

HIV LTR

6,000 6,010 6,020 6,030 6,040 6,050 6,060 6,070 6,080 6,090

CTCTGGTAACTAGAGATCCCTCAGACCCTTTTAGTCAGTGTGGAAAATCTCTAGCAGTGGCGCCCGAACAGGGACTTGAAAGCGAAAGGGAAACCAGAGGAGCTCTC
GAGACCATTGATCTCTAGGGAGTCTGGGAAAATCAGTCACACCTTTTAGAGATCGTCACCGCGGGCTTGTCCCTGAACTTTCGCTTTCCCTTTGGTCTCCTCGAGAG

HIV LTR

6,100 6,110 6,120 6,130 6,140 6,150 6,160 6,170 6,180 6,190 6,200

TCGACGCAGGACTCGGCTTGCTGAAGCGCGCACGGCAAGAGGCGAGGGGCGGCGACTGGTGAGTACGCCAAAAATTTTGACTAGCGGAGGCTAGAAGGAGAGAGATG
AGCTGCGTCCTGAGCCGAACGACTTCGCGCGTGCCGTTCTCCGCTCCCCGCCGCTGACCACTCATGCGGTTTTTAAAACTGATCGCCTCCGATCTTCCTCTCTCTAC

Psi

6,210 6,220 6,230 6,240 6,250 6,260 6,270 6,280 6,290 6,300 6,310

Fyve-HA-TEV219 pCDH-CuO-MCS-EF1α-CymR-T2A-Bleo3 SparQ (10070 bp)

NruI XmnI

GGTGCGAGAGCGTCAGTATTAAGCGGGGGAGAATTAGATCGCGATGGGAAAAAATTCGGTTAAGGCCAGGGGGAAAGAAAAAATATAAATTAAAACATATAGTATGG
CCACGCTCTCGCAGTCATAATTCGCCCCCTCTTAATCTAGCGCTACCCTTTTTTAAGCCAATTCCGGTCCCCCTTTCTTTTTTATATTTAATTTTGTATATCATACC

Gag

6,320 6,330 6,340 6,350 6,360 6,370 6,380 6,390 6,400 6,410 6,420

GCAAGCAGGGAGCTAGAACGATTCGCAGTTAATCCTGGCCTGTTAGAAACATCAGAAGGCTGTAGACAAATACTGGGACAGCTACAACCATCCCTTCAGACAGGATC
CGTTCGTCCCTCGATCTTGCTAAGCGTCAATTAGGACCGGACAATCTTTGTAGTCTTCCGACATCTGTTTATGACCCTGTCGATGTTGGTAGGGAAGTCTGTCCTAG

Gag

6,430 6,440 6,450 6,460 6,470 6,480 6,490 6,500 6,510 6,520

BspQI
SapI

AGAAGAACTTAGATCATTATATAATACAGTAGCAACCCTCTATTGTGTGCATCAAAGGATAGAGATAAAAGACACCAAGGAAGCTTTAGACAAGATAGAGGAAGAGC
TCTTCTTGAATCTAGTAATATATTATGTCATCGTTGGGAGATAACACACGTAGTTTCCTATCTCTATTTTCTGTGGTTCCTTCGAAATCTGTTCTATCTCCTTCTCG

Gag

6,530 6,540 6,550 6,560 6,570 6,580 6,590 6,600 6,610 6,620 6,630

MfeI

AAAACAAAAGTAAGACCACCGCACAGCAAGCGGCCACTGATCTTCAGACCTGGAGGAGGAGATATGAGGGACAATTGGAGAAGTGAATTATATAAATATAAAGTAGT
TTTTGTTTTCATTCTGGTGGCGTGTCGTTCGCCGGTGACTAGAAGTCTGGACCTCCTCCTCTATACTCCCTGTTAACCTCTTCACTTAATATATTTATATTTCATCA

Gag Env

6,640 6,650 6,660 6,670 6,680 6,690 6,700 6,710 6,720 6,730 6,740

AAAAATTGAACCATTAGGAGTAGCACCCACCAAGGCAAAGAGAAGAGTGGTGCAGAGAGAAAAAAGAGCAGTGGGAATAGGAGCTTTGTTCCTTGGGTTCTTGGGAG
TTTTTAACTTGGTAATCCTCATCGTGGGTGGTTCCGTTTCTCTTCTCACCACGTCTCTCTTTTTTCTCGTCACCCTTATCCTCGAAACAAGGAACCCAAGAACCCTC

RRE
Env

6,750 6,760 6,770 6,780 6,790 6,800 6,810 6,820 6,830 6,840

BbvCI

CAGCAGGAAGCACTATGGGCGCAGCGTCAATGACGCTGACGGTACAGGCCAGACAATTATTGTCTGGTATAGTGCAGCAGCAGAACAATTTGCTGAGGGCTATTGAG
GTCGTCCTTCGTGATACCCGCGTCGCAGTTACTGCGACTGCCATGTCCGGTCTGTTAATAACAGACCATATCACGTCGTCGTCTTGTTAAACGACTCCCGATAACTC

RRE
Env

6,850 6,860 6,870 6,880 6,890 6,900 6,910 6,920 6,930 6,940 6,950

BstAPI

GCGCAACAGCATCTGTTGCAACTCACAGTCTGGGGCATCAAGCAGCTCCAGGCAAGAATCCTGGCTGTGGAAAGATACCTAAAGGATCAACAGCTCCTGGGGATTTG
CGCGTTGTCGTAGACAACGTTGAGTGTCAGACCCCGTAGTTCGTCGAGGTCCGTTCTTAGGACCGACACCTTTCTATGGATTTCCTAGTTGTCGAGGACCCCTAAAC

RRE
Env

6,960 6,970 6,980 6,990 7,000 7,010 7,020 7,030 7,040 7,050 7,060

Fyve-HA-TEV219 pCDH-CuO-MCS-EF1α-CymR-T2A-Bleo3 SparQ (10070 bp)

AleI

GGGTTGCTCTGGAAAACTCATTTGCACCACTGCTGTGCCTTGGAATGCTAGTTGGAGTAATAAATCTCTGGAACAGATTTGGAATCACACGACCTGGATGGAGTGGG
CCCAACGAGACCTTTTGAGTAAACGTGGTGACGACACGGAACCTTACGATCAACCTCATTATTTAGAGACCTTGTCTAAACCTTAGTGTGCTGGACCTACCTCACCC

RRE
Env

7,070 7,080 7,090 7,100 7,110 7,120 7,130 7,140 7,150 7,160

ACAGAGAAATTAACAATTACACAAGCTTAATACACTCCTTAATTGAAGAATCGCAAACCAGCAAGAAAAGAATGAACAAGAATTATTGGAATTAGATAAATGGGCAA
TGTCTCTTTAATTGTTAATGTGTTCGAATTATGTGAGGAATTAACTTCTTAGCGTTTGGTCGTTCTTTTCTTACTTGTTCTTAATAACCTTAATCTATTTACCCGTT

Env

7,170 7,180 7,190 7,200 7,210 7,220 7,230 7,240 7,250 7,260 7,270

GTTTGTGGAATTGGTTTAACATAACAAATTGGCTGTGGTATATAAAATTATTCATAATGATAGTAGGAGGCTTGGTAGGTTTAAGAATAGTTTTTGCTGTACTTTCT
CAAACACCTTAACCAAATTGTATTGTTTAACCGACACCATATATTTTAATAAGTATTACTATCATCCTCCGAACCATCCAAATTCTTATCAAAAACGACATGAAAGA

Env

7,280 7,290 7,300 7,310 7,320 7,330 7,340 7,350 7,360 7,370 7,380

PpuMI
KflI

ATAGTGAATAGAGTTAGGCAGGGATATTCACCATTATCGTTTCAGACCCACCTCCCAACCCCGAGGGGACCCGACAGGCCCGAAGGAATAGAAGAAGAAGGTGGAGA
TATCACTTATCTCAATCCGTCCCTATAAGTGGTAATAGCAAAGTCTGGGTGGAGGGTTGGGGCTCCCCTGGGCTGTCCGGGCTTCCTTATCTTCTTCTTCCACCTCT

Env

7,390 7,400 7,410 7,420 7,430 7,440 7,450 7,460 7,470 7,480 7,490

HpaI

GAGAGACAGAGACAGATCCATTCGATTAGTGAACGGATCTCGACGGTATCGGTTAACTTTTAAAAGAAAAGGGGGGATTGGGGGGTACAGTGCAGGGGAAAGAATAG
CTCTCTGTCTCTGTCTAGGTAAGCTAATCACTTGCCTAGAGCTGCCATAGCCAATTGAAAATTTTCTTTTCCCCCCTAACCCCCCATGTCACGTCCCCTTTCTTATC

Env
cPPT

7,500 7,510 7,520 7,530 7,540 7,550 7,560 7,570 7,580 7,590

BspDI
ClaI
SpeI

TAGACATAATAGCAACAGACATACAAACTAAAGAATTACAAAAACAAATTACAAAATTCAAAATTTTATCGATAGACTAGTTATTAATAGTAATCAATTACGGGGTC
ATCTGTATTATCGTTGTCTGTATGTTTGATTTCTTAATGTTTTTGTTTAATGTTTTAAGTTTTAAAATAGCTATCTGATCAATAATTATCATTAGTTAATGCCCCAG

cPPT
CMV + CUO

7,600 7,610 7,620 7,630 7,640 7,650 7,660 7,670 7,680 7,690 7,700

ATTAGTTCATAGCCCATATATGGAGTTCCGCGTTACATAACTTACGGTAAATGGCCCGCCTGGCTGACCGCCCAACGACCCCCGCCCATTGACGTCAATAATGACGT
TAATCAAGTATCGGGTATATACCTCAAGGCGCAATGTATTGAATGCCATTTACCGGGCGGACCGACTGGCGGGTTGCTGGGGGCGGGTAACTGCAGTTATTACTGCA

CMV + CUO

7,710 7,720 7,730 7,740 7,750 7,760 7,770 7,780 7,790 7,800 7,810

NdeI

ATGTTCCCATAGTAACGCCAATAGGGACTTTCCATTGACGTCAATGGGTGGAGTATTTACGGTAAACTGCCCACTTGGCAGTACATCAAGTGTATCATATGCCAAGT
TACAAGGGTATCATTGCGGTTATCCCTGAAAGGTAACTGCAGTTACCCACCTCATAAATGCCATTTGACGGGTGAACCGTCATGTAGTTCACATAGTATACGGTTCA

CMV + CUO

7,820 7,830 7,840 7,850 7,860 7,870 7,880 7,890 7,900 7,910

SnaBI
BsaAI

CCGCCCCCTATTGACGTCAATGACGGTAAATGGCCCGCCTGGCATTATGCCCAGTACATGACCTTACGGGACTTTCCTACTTGGCAGTACATCTACGTATTAGTCAT
GGCGGGGGATAACTGCAGTTACTGCCATTTACCGGGCGGACCGTAATACGGGTCATGTACTGGAATGCCCTGAAAGGATGAACCGTCATGTAGATGCATAATCAGTA

CMV + CUO

7,920 7,930 7,940 7,950 7,960 7,970 7,980 7,990 8,000 8,010 8,020

CspCI

CGCTATTACCATGGTGATGCGGTTTTGGCAGTACACCAATGGGCGTGGATAGCGGTTTGACTCACGGGGATTTCCAAGTCTCCACCCCATTGACGTCAATGGGAGTT
GCGATAATGGTACCACTACGCCAAAACCGTCATGTGGTTACCCGCACCTATCGCCAAACTGAGTGCCCCTAAAGGTTCAGAGGTGGGGTAACTGCAGTTACCCTCAA

CMV + CUO

8,030 8,040 8,050 8,060 8,070 8,080 8,090 8,100 8,110 8,120 8,130

TGTTTTGGCACCAAAATCAACGGGACTTTCCAAAATGTCGTAATACCCCCGCCCCGTTGACGCAAATGGGCAAGCTTGCCGGGTCGAGGTAGGCGTGTACGGTGGGA
ACAAAACCGTGGTTTTAGTTGCCCTGAAAGGTTTTACAGCATTATGGGGGCGGGGCAACTGCGTTTACCCGTTCGAACGGCCCAGCTCCATCCGCACATGCCACCCT

CMV + CUO | CMV + CUO

8,140 8,150 8,160 8,170 8,180 8,190 8,200 8,210 8,220 8,230

StuI AgeI AgeI AscI

GGCCTATATAAGCAACCGGTATAATCAAACAGACCAGATTGTCTGTTTGTTACCGGTGTTTAGTGAACCGGGCGCGCCTCATATCGCCTGGAGACGCCATCCACGCT
CCGGATATATTCGTTGGCCATATTAGTTTGTCTGGTCTAACAGACAAACAATGGCCACAAATCACTTGGCCCGCGCGGAGTATAGCGGACCTCTGCGGTAGGTGCGA

CMV + CUO

8,240 8,250 8,260 8,270 8,280 8,290 8,300 8,310 8,320 8,330 8,340

GTTTTGACCTCCATAGAAGACACCGGGACCGATCCAGCCTCCGCGGTCACTCTCTTCCGCATCGCTGTCTGCGAGGGCCAGCTGTTGGGCTCGCGGTTGAGGACAAA
CAAAACTGGAGGTATCTTCTGTGGCCCTGGCTAGGTCGGAGGCGCCAGTGAGAGAAGGCGTAGCGACAGACGCTCCCGGTCGACAACCCGAGCGCCAACTCCTGTTT

CMV + CUO

8,350 8,360 8,370 8,380 8,390 8,400 8,410 8,420 8,430 8,440 8,450

PpuMI

CTCTTCGCGGTCTTTCCAGTACTCTTGGATCGGAAACCCGTCGGCCTCCGAACGGTACTCCGCCACCGAGGGACCTGAGCGAGTCCGCATCGACCGGATCGGAAAAC
GAGAAGCGCCAGAAAGGTCATGAGAACCTAGCCTTTGGGCAGCCGGAGGCTTGCCATGAGGCGGTGGCTCCCTGGACTCGCTCAGGCGTAGCTGGCCTAGCCTTTTG

CMV + CUO | CMV + CUO

8,460 8,470 8,480 8,490 8,500 8,510 8,520 8,530 8,540 8,550 8,560

Fyve-HA-TEV219 pCDH-CuO-MCS-EF1α-CymR-T2A-Bleo3 SparQ (10070 bp)

PaeR7I
XhoI
TliI
BlpI

CTCTCGAGAAAGGCGTCTAACCAGTCACAGTCGCAAGGTAGGCTGAGCACCGTGGCGGGCGGCAGCGGGTGGCGGTCGGGGTTGTTTCTGGCGGAGGTGCTGCTGAT
GAGAGCTCTTTCCGCAGATTGGTCAGTGTCAGCGTTCCATCCGACTCGTGGCACCGCCCGCCGTCGCCCACCGCCAGCCCCAACAAAGACCGCCTCCACGACGACTA

CMV + CUO

8,570 8,580 8,590 8,600 8,610 8,620 8,630 8,640 8,650 8,660

GATGTAATTAAAGTAGGCGGTCTTGAGACGGCGGATGGTCGAGGTGAGGTGTGGCAGGCTTGAGATCCAGCTGTTGGGGTGAGTACTCCCTCTCAAAAGCGGGCATT
CTACATTAATTTCATCCGCCAGAACTCTGCCGCCTACCAGCTCCACTCCACACCGTCCGAACTCTAGGTCGACAACCCCACTCATGAGGGAGAGTTTTCGCCCGTAA

CMV + CUO
CMV + CUO

8,670 8,680 8,690 8,700 8,710 8,720 8,730 8,740 8,750 8,760 8,770

MscI
AleI

ACTTCTGCGCTAAGATTGTCAGTTTCCAAAAACGAGGAGGATTTGATATTCACCTGGCCCGATCTGGCCATACACTTGAGTGACAATGACATCCACTTTGCCTTTCT
TGAAGACGCGATTCTAACAGTCAAAGGTTTTTGCTCCTCCTAAACTATAAGTGGACCGGGCTAGACCGGTATGTGAACTCACTGTTACTGTAGGTGAAACGGAAAGA

CMV + CUO

8,780 8,790 8,800 8,810 8,820 8,830 8,840 8,850 8,860 8,870 8,880

BmtI
NheI
BarI

CTCCACAGGTGTCCACTCCCAGGTCCAAGTTTGGTCTAGAGCTAGCGCCACCATGggtggatctagtttcgtaactgcaaatgaagattctgtacctgaaaacactt
GAGGTGTCCACAGGTGAGGGTCCAGGTTCAAACCAGATCTCGATCGCGGTGGTACccacctagatcaaagcattgacgtttacttctaagacatggacttttgtgaa

M G G S S F V T A N E D S V P E N T

CMV + CUO
FYVE

8,890 8,900 8,910 8,920 8,930 8,940 8,950 8,960 8,970 8,980

gcaaagaaggcttggttttgggccagaaacagcctacttgggttcctgattcagaagctccaaactgtatgaactgccaagtcaaatttacttttaccaaacggcga
cgtttcttccgaaccaaaacccggtctttgtcggatgaacccaaggactaagtcttcgaggtttgacatacttgacggttcagtttaaatgaaaatggtttgccgct

C K E G L V L G Q K Q P T W V P D S E A P N C M N C Q V K F T F T K R R

Minimal FYVE
FYVE

8,990 9,000 9,010 9,020 9,030 9,040 9,050 9,060 9,070 9,080 9,090

caccattgccgagcatgtgggaaagtatttgtggtgtctgttgtaataggaagtgtaaactgcaatatctagaaaaggaagcaagagtatgtgtagtctgctatga
gtggtaacggctcgtacaccctttcataaacaccacagacaacattatccttcacatttgacgttatagatcttttccttcgttctcatacacatcagacgatact

H H C R A C G K V F C G V C C N R K C K L Q Y L E K E A R V C V V C Y E

Minimal FYVE
FYVE

9,100 9,110 9,120 9,130 9,140 9,150 9,160 9,170 9,180 9,190 9,200

Fyve-HA-TEV219 pCDH-CuO-MCS-EF1α-CymR-T2A-Bleo3 SparQ (10070 bp)

Bsu36I
AloI

aactattagtaaagctcaggcatttgaaaggatgatgagtccaactggttctaatcttaagtctaatcattctgatgaatgtactactgtccagcctcctcaggaga

ttgataatcatttcgagtccgtaaactttcctactactcaggttgaccaagattagaattcagattagtaagactacttacatgatgacaggtcggaggagtcctct

T I S K A Q A F E R M M S P T G S N L K S N H S D E C T T V Q P P Q E

Mi...E

FYVE

9,210 9,220 9,230 9,240 9,250 9,260 9,270 9,280 9,290 9,300

XcmI
BspEI

accaaacatccagtgGATCCGGTAGTGGTGGTGGGTCTGGGGGAGGGTCCGGAGGCGGCTCTGGTGGTGGGTCATACCCTTACGACGTACCTGATTACGCGGGCTCA

tggtttgtaggtcacCTAGGCCATCACCACCACCCAGACCCCCTCCCAGGCCTCCGCCGAGACCACCACCCAGTATGGGAATGCTGCATGGACTAATGCGCCCGAGT

N Q T S S G S G S G G G S G G G S G G G S G G G S Y P Y D V P D Y A G S

Linker

FYVE

HA-Tag

9,310 9,320 9,330 9,340 9,350 9,360 9,370 9,380 9,390 9,400 9,410

ApaI
PspOMI

TCTCTGTTCAAGGGCCCAAGAGACTATAATCCTATCTCATCAACGATCTGCCATCTCACAAACGAATCCGACGGACACACGACCTCTCTTTACGGGATTGGTTTCGG

AGAGACAAGTTCCCGGGTTCTCTGATATTAGGATAGAGTAGTTGCTAGACGGTAGAGTGTTTGCTTAGGCTGCCTGTGTGCTGGAGAGAAATGCCCTAACCAAAGCC

S L F K G P R D Y N P I S S T I C H L T N E S D G H T T S L Y G I G F G

Tev 219

9,420 9,430 9,440 9,450 9,460 9,470 9,480 9,490 9,500 9,510 9,520

XcmI

CCCTTTTATCATAACTAATAAACATCTTTTCCGCCGGAACAACGGAACTTTGCTTGTCCAGTCACTTCATGGCGTATTTAAAGTGAAGAATACTACTACTTTGCAAC

GGGAAAATAGTATTGATTATTTGTAGAAAAGGCGGCCTTGTTGCCTTGAAACGAACAGGTCAGTGAAGTACCGCATAAATTTCACTTCTTATGATGATGAAACGTTG

P F I I T N K H L F R R N N G T L L V Q S L H G V F K V K N T T T L Q

Tev 219

9,530 9,540 9,550 9,560 9,570 9,580 9,590 9,600 9,610 9,620 9,630

AACATCTCATTGACGGCCGGGACATGATTATTATAAGAATGCCGAAGGATTTTCCACCTTTCCCCCAAAAACTTAAGTTTCGGGAGCCACAACGAGAGGAACGAATA

TTGTAGAGTAACTGCCGGCCCTGTACTAATAATATTCTTACGGCTTCCTAAAAGGTGGAAAGGGGGTTTTTGAATTCAAAGCCCTCGGTGTTGCTCTCCTTGCTTAT

Q H L I D G R D M I I I R M P K D F P P F P Q K L K F R E P Q R E E R I

Tev 219

9,640 9,650 9,660 9,670 9,680 9,690 9,700 9,710 9,720 9,730

Fyve-HA-TEV219 pCDH-CuO-MCS-EF1α-CymR-T2A-Bleo3 SparQ (10070 bp)

BsrGI

TGCCTGGTAACAACAAATTTTCAAACTAAATCTATGTCTTCAATGGTGAGTGACACATCTTGTACATTTCCCTCTAGTGACGGGATTTTCTGGAAGCATTGGATACA
ACGGACCATTGTTGTTTAAAAGTTTGATTTAGATACAGAAGTTACCACTCACTGTGTAGAACATGTAAAGGGAGATCACTGCCCTAAAAGACCTTCGTAACCTATGT
C L V T T N F Q T K S M S S M V S D T S C T F P S S D G I F W K H W I Q

Tev 219

9,740 9,750 9,760 9,770 9,780 9,790 9,800 9,810 9,820 9,830 9,840

GACTAAGGACGGGCAGTGCGGATCACCACTCGTGTCCACACGCGATGGATTCATTGTCGGGATTCATAGTGCTTCTAACTTCACCAACACCAATAATTATTTTACTT
CTGATTCCTGCCCGTCACGCCTAGTGGTGAGCACAGGTGTGCGCTACCTAAGTAACAGCCCTAAGTATCACGAAGATTGAAGTGGTTGTGGTTATTAATAAAATGAA
T K D G Q C G S P L V S T R D G F I V G I H S A S N F T N T N N Y F T

Tev 219

9,850 9,860 9,870 9,880 9,890 9,900 9,910 9,920 9,930 9,940 9,950

Tth111I
AloI PflFI

CTGTGCCAAAAAACTTCATGGAGCTGCTTACTAGCCAAGAAGCCCAACAGTGGGTGAGTGGGTGGCGCCTGAACGCCGACTCCGTCTTGTGGGGGGGTCACAAGGTC
GACACGGTTTTTTGAAGTACCTCGACGAATGATCGGTTCTTCGGGTTGTCACCCACTCACCCACCGCGGACTTGCGGCTGAGGCAGAACACCCCCCCAGTGTTCCAG
S V P K N F M E L L T S Q E A Q Q W V S G W R L N A D S V L W G G H K V

Tev 219

9,960 9,970 9,980 9,990 10,000 10,010 10,020 10,030 10,040 10,050

TTTATGGTATAA
AAATACCATATT
F M V *

Tev 219

10,060 10,070
