# Supplementary material for: Profiling of basal and ligand-dependent GPCR activities by means of a polyvalent cell-based high-throughput platform
Source: Nat Commun. 2023 Jul 5;14:3684. doi: 10.1038/s41467-023-39132-x (PMC10322906; doi:10.1038/s41467-023-39132-x)
Supplement: Supplementary file 2 — Reporting Summary [file 41467_2023_39132_MOESM2_ESM.pdf]

nature portfolio

Corresponding author(s): Patrick Giguère

Last updated by author(s): May 10, 2023

Reporting Summary

Nature Portfolio wishes to improve the reproducibility of the work that we publish. This form provides structure for consistency and transparency in reporting. For further information on Nature Portfolio policies, see our Editorial Policies and the Editorial Policy Checklist.

Statistics

For all statistical analyses, confirm that the following items are present in the figure legend, table legend, main text, or Methods section.

n/a	Confirmed	
☐	☒	The exact sample size (*n*) for each experimental group/condition, given as a discrete number and unit of measurement
☐	☒	A statement on whether measurements were taken from distinct samples or whether the same sample was measured repeatedly
☐	☒	The statistical test(s) used AND whether they are one- or two-sided<br>*Only common tests should be described solely by name; describe more complex techniques in the Methods section.*
☒	☐	A description of all covariates tested
☐	☒	A description of any assumptions or corrections, such as tests of normality and adjustment for multiple comparisons
☐	☒	A full description of the statistical parameters including central tendency (e.g. means) or other basic estimates (e.g. regression coefficient) AND variation (e.g. standard deviation) or associated estimates of uncertainty (e.g. confidence intervals)
☐	☒	For null hypothesis testing, the test statistic (e.g. *F*, *t*, *r*) with confidence intervals, effect sizes, degrees of freedom and *P* value noted<br>*Give P values as exact values whenever suitable.*
☒	☐	For Bayesian analysis, information on the choice of priors and Markov chain Monte Carlo settings
☒	☐	For hierarchical and complex designs, identification of the appropriate level for tests and full reporting of outcomes
☐	☒	Estimates of effect sizes (e.g. Cohen's *d*, Pearson's *r*), indicating how they were calculated

Our web collection on statistics for biologists contains articles on many of the points above.

Software and code

Policy information about availability of computer code

Data collection	Luminescence readings were collected using BioTek Gen5 software v3.11 from the Synergy Neo2 microplate reader (see https://www.agilent.com/en/product/microplate-instrumentation/microplate-instrumentation-control-analysis-software/imager-reader-control-analysis-software/biotek-gen5-software-for-detection-1623227).
Data analysis	All data was analyzed using GraphPad Prism version 9.5.1

For manuscripts utilizing custom algorithms or software that are central to the research but not yet described in published literature, software must be made available to editors and reviewers. We strongly encourage code deposition in a community repository (e.g. GitHub). See the Nature Portfolio guidelines for submitting code & software for further information.

Data

Policy information about availability of data

All manuscripts must include a data availability statement. This statement should provide the following information, where applicable:

- Accession codes, unique identifiers, or web links for publicly available datasets
- A description of any restrictions on data availability
- For clinical datasets or third party data, please ensure that the statement adheres to our policy

The following Data Availability statement was included in the manuscript:

All data generated or analyzed during this study, including data underlying Figures 1-8 and all Supplementary Figures, are provided as a Source Data file, accessible at the Figshare repository.
Human Protein Atlas (HPA) RNA consensus tissue gene data (version 21.0 and Ensembl version 103.38.) used for the production of Figure 8 was accessed at https://www.proteinatlas.org/about/.
EMTA data compared in Supplementary Table 1, including Emax (in % of vehicle response) and absolute pEC50 values, was downloaded from https://cdn.elifesciences.org/articles/74101/elife-74101-supp2-v2.xlsx.

Research involving human participants, their data, or biological material

Policy information about studies with human participants or human data. See also policy information about sex, gender (identity/presentation), and sexual orientation and race, ethnicity and racism.

Reporting on sex and gender	No human participants were involved, thus reporting on sex and gender is not relevant to our study.
Reporting on race, ethnicity, or other socially relevant groupings	No human participants were involved, thus reporting on race, ethnicity, or other socially relevant groupings is not relevant to our study.
Population characteristics	No human participants were involved, thus reporting on population characteristics is not relevant to our study.
Recruitment	No human participants were involved, thus reporting on recruitment is not relevant to our study.
Ethics oversight	No human participants were involved, thus ethics approval for study protocols involving humans is not relevant to our study.

Note that full information on the approval of the study protocol must also be provided in the manuscript.

Field-specific reporting

Please select the one below that is the best fit for your research. If you are not sure, read the appropriate sections before making your selection.

☒ Life sciences ☐ Behavioural & social sciences ☐ Ecological, evolutionary & environmental sciences

For a reference copy of the document with all sections, see nature.com/documents/nr-reporting-summary-flat.pdf

Life sciences study design

All studies must disclose on these points even when the disclosure is negative.

Sample size

No sample-size calculations were performed. Indicated sample sizes (n=6-8 for main Figures 1-7; n=2-12 for Supplementary Figures) were chosen based on the previous published work of experienced researchers in the field who have developed cell-based platforms of similar design; these include but are not limited to the original PRESTO-Tango (n=3-4; PMID: 25895059), modified versions of Tango such as the piggyBac-TANGO (n=3; PMID: 31122241), and other systems of high-throughput nature such as EMTA (n=2-12; PMID: 35302493). Other examples of these high-throughput assays are also summarized in the review PMID: 32653805, all of which have similar sample sizes to those chosen in our study.

Data exclusions

Certain outlier values, which arose due to a couple of wells exhibiting significant cell detachment or cell aggregation/clumping, OR due to luminescence signal bleed-through between adjacent wells (when using white 384-well plates), were detected by checking microplates under the microscope prior to adding luciferase detection reagent. These outlier wells were noted and subsequently excluded during data processing.

Replication

To verify reproducibility of the findings, main Figures were replicated as n=6 or n=8, specifically in 2 biological replicates with 3 or 4 technical replicates each; all findings presented in the main Figures were successfully replicated, with replicated figures provided in the Supplementary Information file.

As for the Supplementary Figures, Supplementary Figures 1-2 were replicated as n=12 (3 technical replicates from 4 biological samples). All other Supplementary Figures represent n=2-4 (2-4 technical replicates from one biological sample). Given the sheer amount of data generated in this study, time and resources did not permit additional biological replications for the majority of the Supplementary Figures, but successful reproducibility of the technical replicates can be assessed using the Goodness of Fit parameters reported by GraphPad Prism, specifically for results with R squared values >0.8 (for cases where no arrestin recruitment or internalization is detected at a receptor i.e. flat curve, these will be recorded as "unstable" or naturally will have low R squares values given the lack of curve fitting observed); the Goodness of Fit parameters for all Supplementary Figures are included for users in the Source Data file.

Randomization

GPCR-ome HTS screens: Homogeneous cell mastermixes were plated in multiple 384-well plates, and were randomly assigned to previously-prepared GPCR-Tango DNA plates for transfection (e.g., DNA plate 1, plate 2, etc.).

Dose-response curves: Following plating of the cell mastermixes in the designated vessels (6-well dishes, 384 well plates), cell samples were randomly assigned their transfection conditions. Transfection conditions were assigned arbitrary numeric designations, and cell samples were randomly numbered (e.g., CHRM5-Tango = 1; MC4R-Tango = 2, etc.).

Blinding

Investigators could not be completely blinded throughout the entirety of the data collection, as certain experiments require stimulation of cell

Blinding	samples with receptor-specific agonists/antagonists/inverse agonists, thus investigators need to refer back to the transfection conditions (i.e. receptor identities) to determine the appropriate drugs to use.<br><br>For all other experiments, we tried to implement blinding to the best of our abilities through a couple of methods: 1) assigning shorthand numerical designations to transfection conditions at the beginning of the experiment, and keeping these throughout the entirety of the experiment until data analysis (such that investigators do not know which conditions they are handling during data collection); 2) having more than one investigator perform a portion of the data collection of any given experiment (e.g., one investigator plates cells, assigns numerical designations for transfection conditions and transfects cells, and a different investigator performs the remainder of the experiment)

Reporting for specific materials, systems and methods

We require information from authors about some types of materials, experimental systems and methods used in many studies. Here, indicate whether each material, system or method listed is relevant to your study. If you are not sure if a list item applies to your research, read the appropriate section before selecting a response.

Materials & experimental systems

n/a	Involved in the study	
☐	☒	Antibodies
☐	☒	Eukaryotic cell lines
☒	☐	Palaeontology and archaeology
☒	☐	Animals and other organisms
☒	☐	Clinical data
☒	☐	Dual use research of concern
☒	☐	Plants

Methods

n/a	Involved in the study	
☒	☐	ChIP-seq
☒	☐	Flow cytometry
☒	☐	MRI-based neuroimaging

Antibodies

Antibodies used	Monoclonal ANTI-FLAG® M2-Peroxidase (HRP) antibody, MilliporeSigma, Cat. #A8592, Clone M2
Validation	The antibody used was chosen based on the published methods of the original PRESTO-Tango (see PMID: 25895059, under Methods, subsection " Immunofluorescence")

Eukaryotic cell lines

Policy information about cell lines and Sex and Gender in Research

Cell line source(s)	HEK293T cells: ATCC, Cat# CRL-3216; HTL and HTLA cells : A gift from Dr. Richard Axel, Columbia University; Tango Trio cells (HTTL, HTTL-B1, HTTL-B2, HTTL-F): generated herein.
Authentication	Cells were not authenticated.
Mycoplasma contamination	Mycoplasma contamination was tested by PCR; if tested positive, cells were treated with 2-week treatment with Plasmocure™ (InvivoGen) and re-tested by PCR. All cell lines tested negative for mycoplasma before experiments.
Commonly misidentified lines (See ICLAC register)	No misidentified cell lines were used in this study based on the current ICLAC register.
